# FASTOP—Fast Editing Toolkit for Top Expression Sites in Yeast

**DOI:** 10.1021/acs.jafc.6c06735

**Published:** 2026-10-21

**Authors:** Madhushruti Borah, Nicolas Gautron, Vincent Courdavault, Gita Naseri

**Affiliations:** † Institut für Biologie, 9373 Humboldt-Universität zu Berlin, Philippstrasse 13, 10115 Berlin, Germany; ‡ EA2106 Biomolécules et Biotechnologies Végétales, Université de Tours, 31 avenue Monge, 37200 Tours, France; § Max Planck Unit for the Science of Pathogens, Charitéplatz 1, 10117 Berlin, Germany

**Keywords:** CRISPR/Cas9, genome engineering, hot-spot, naringenin, Saccharomyces cerevisiae, synthetic biology

## Abstract

Budding yeast *Saccharomyces cerevisiae* is a widely used chassis for producing food- and agriculture-related compounds, but construction of multienzyme pathways often requires repeated genomic integration. We developed FASTOP, a genome-editing toolkit for rapid engineering of eight transcriptionally active genomic hot-spot loci. FASTOP combines selectable markers, extended homology arms, and Cas9/gRNA-assisted integration, and enables PCR-free release of donor cassettes by restriction digestion to reduce sequence errors introduced during amplification. After characterizing the sites, we integrated a five-gene naringenin biosynthetic pathway into the hot spots as a proof of concept. Our results demonstrated that, upon expressing the five essential genes in simple shake-flask culture, naringenin production reached 505.7 mg/L, 6-fold higher than an episomal control expressing the same pathway genes and 4.5-fold higher than the previously reported strain using additional precursor engineering and controlled bioreactor cultivation. FASTOP therefore provides a practical platform for the rapid assembly of multienzyme pathways in yeast for stable heterologous gene expression.

## Graphical abstract

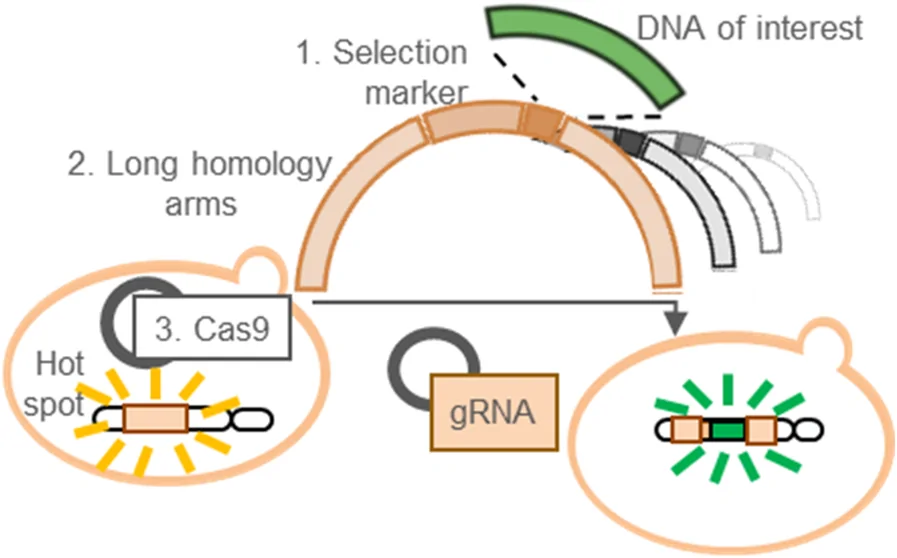

## Introduction

*Saccharomyces cerevisiae* is an industrial chassis for synthetic biology, which allows the production of value-added compounds and biofuels.^1–3^ However, a major limitation for efficient production of heterologous metabolites in microbial hosts is the low transcription of key pathway genes,^4–6^ which often results in constrained metabolic flux and decreases strain engineering progress.^7^ Overcoming transcriptional bottlenecks remains a central challenge in pathway optimization in a microbial cell factory.^4^ The biosynthesis of strictosidine, the central precursor to numerous anticancer natural products in the monoterpenoid indole alkaloid (MIA) family, is a compelling example of this difficulty.^5,8^ Reconstituting strictosidine production in yeast relies on the coordinated expression of many heterologous plant enzymes as well as endogenous yeast genes to establish a highly complex plant-derived metabolic network in the microbe.^8^ Any imbalance in expression levels can severely limit pathway performance.^9^ Notably, the remarkable ∼33-fold increase in strictosidine production was achieved by increasing the copy number of genes encoding rate-limiting enzymes.^10^ An alternative, potentially more streamlined strategy than introducing multiple gene copies is to integrate pathway genes into genomic loci with intrinsically high transcriptional activity.

Strategic integration into high-expression “hot-spot” sites in the yeast genome can enhance transcript abundance while maintaining genomic stability. Our understanding of chromosomal positional effects on gene expression in *S. cerevisiae* has recently been significantly improved.^11,12^ This has enabled us to implement new approaches to rational, more predictable strain engineering. While CRISPR/Cas9 has accelerated targeted genome editing, to our knowledge, none of the existing toolkits enable PCR-free integration into hot-spot genomic sites with high efficiency. In response, we developed FASTOP, a Flexible, Accelerated, Site-specific editing toolkit for engineering TOP expression sites in the yeast genome. FASTOP implements three key design features: (i) Co-delivery of Cas9 and guide RNAs (gRNAs) to induce site-specific double-strand DNA breaks (DSBs) at any target loci; (ii) Extended homology arms (≥500 bp) to enhance homology-directed repair (HDR) efficiency by native yeast precise DNA repair machinery; and (iii) Yeast selection markers for screening of edited clones, to enable identification of successful integrants without the need for sequencing. Crucially, FASTOP enables PCR-free cassette release using restriction enzymes, eliminating the need for PCR amplification^13^ and thereby reducing the risk of sequence errors.

Eight desirable hot-spot loci, located adjacent to the highest reported expressed genes, were fully characterized for three criteria: (1) CRISPR-mediated GFP integration efficiency; (2) expression level, assessed by GFP fluorescence; and (3) multienzyme pathway performance, evaluated through production of naringenin, a flavonoid known for its antioxidant, anti-inflammatory, and applications in functional foods and nutraceuticals.^14,15^ In summary, FASTOP provides a user-friendly, scalable, and high-performance genome-editing platform for the high-level expression of heterologous genes in yeast.

## Materials and Methods

### General

Plasmids were assembled using NEBuilder HiFi DNA assembly (New England Biolabs, Frankfurt am Main, Germany).^16,17^ All gBlocks, primers, and oligonucleotides were ordered from IDT (Integrated DNA Technologies Inc., Dessau-Rosslau, Germany). PCR amplifications were done using Q5 DNA Polymerase (New England Biolabs, Frankfurt am Main, Germany), or PrimeSTAR GXL DNA Polymerase (Takara Bio, Saint-Germain-en-Laye, France) according to the manufacturer’s recommendations. Amplified DNA parts were gel-purified before further use. All constructs were confirmed by sequencing (GENEWIZ, Germany). Primer and plasmid sequences are given in Supplementary Tables S1 and S2, respectively. All strains generated in this study are listed in Table 1.

**Table 1:** List of Parental *S. cerevisiae* Strains Used in This Study

**Yeast strain**	**Relevant genome**	**Origin**
**YPH500**	MAT a, ura3-52, lys2-801, ade2-101, trp1D63, his3D200, leu2D1	ATCC, #76626
**Pro**_**TEF1**_ **-yEGFP**	YPH500/ura3-52:P_TEF1_-yEGFP-T_CYC1_-his3-ura3	This study
**yMA001**	YPH500/YBR128C(chrII:493424-494478):P_TEF1_-yEGFP-T_CYC1_ -leu2	This study
**yMA002**	YPH500/YDR448W-1(chrIV:1355600-1356683):P_TEF1_-yEGFP-T_CYC1_-trp1	This study
**yMA003**	YPH500/YDR448W-2(chrIV:1355600-1356683):P_TEF1_-yEGFP-T_CYC1_-trp1	This study
**yMA004**	YPH500/YGR240C-1(chrVII:972441-973517):P_TEF1_-yEGFP-T_CYC1_-lys2	This study
**yMA005**	YPH500/YGR240C-2(chrVII:972441-973517):P_TEF1_-yEGFP-T_CYC1_-lys2	This study
**yMA006**	YPH500/YHR142W-1(chrVIII:383398-384450):P_TEF1_-yEGFP-T_CYC1_-natMX	This study
**yMA007**	YPH500/YHR142W-2(chrVIII:383398-384450):P_TEF1_-yEGFP-T_CYC1_-natMX	This study
**yMA008**	YPH500/YDR448W(chrIV:1355600-1356683):P_PGI1_-PhCHI-T_PGI1_-P_FBA1_-AtC4H-L5-AtATR2-T_FBA1_-trp1	This study
**yMA009**	yMA008/YHR142W(chrVIII:383398-384450):P_PGK1_-At4CL-2-T_PGK1_-P_TDH3_-HaCHS-T_TDH3_-natMX	This study
**yFASTOP-NG**	yMA009/YBR128C(chrII:493424-494478):P_TDH1_-AtPAL-2-T_TDH1_-leu2	This study
**yPCEN-NG**	YPH500/pCEN/ARS-P_PGI1_-PhCHI-T_PGI1_-P_FBA1_-AtC4H-L5-AtATR2-T_FBA1_-P_PGK1_-At4CL-2-T_PGK1_-P_TDH3_-HaCHS-T_TDH3_-P_TDH1_-AtPAL-2-T_TDH1_-leu2-trp1-leu2	This study

*Escherichia coli* NEB DH5α (New England Biolabs) was used for the transformation of plasmids. Strains were cultivated at 37 °C in Luria–Bertani (LB) medium with a suitable *E. coli* selection marker. Genetic transformation of plasmids or linearized DNA fragments into competent yeast cells was done using the LiAc/SS carrier DNA/PEG method.^18^ To enable selection for transformed cells, all strains were cultivated at 30 °C in media rich in yeast extract, peptone, dextrose, and adenine (YPDA) or in suitable synthetic complete (SC) media deficient in one or more amino acids. Then, verification of the transformation was performed using colony PCR and/or a microplate reader.

### Design and Construction of CRISPR-Cas9/gRNA Plasmids

The gRNAs for the loci *YBR128C*, *YDR448W*, *YGR240C*, and *YHR142W* were designed using CHOPCHOP v3 ^19^ and Benchling (www.benchling.com). Based on their efficiency and off-target scores, two gRNAs were chosen for each of the above loci. The gRNAs for *H1*, *H2*, *H5*, and *H7*,^11^ along with the eight gRNAs for *YBR128C*, *YDR448W*, *YGR240C*, and *YHR142W*, were synthesized as oligo primers (primers MA01-MA24), as described in Supplementary Table S3. These oligos were annealed and T4-ligated into the BsaI-digested pCRCT backbone, which comprises Cas9, tracrRNA, crRNA, and the URA3 yeast selection marker (Addgene #60621), to construct plasmids pHV001-pHV012. Plasmid pHV002 was modified to pHV013 to change the yeast selection marker to *TRP1* by assembling *TRP1* (primers MA25/MA101, on IDT gBlock) into the pHV002 backbone (primers MA26/MA27). Correct sequence integrity of the constructs was verified by colony PCR and Sanger sequencing.

### Construction of Donor Plasmids

Donors comprising homologous recombination sequences with unique yeast selectable markers were constructed for each locus. Homologous recombination sequences were based on 500 bp flanking the gRNA sequences beyond the protospacer adjacent motif (PAM). For construction of the donor plasmids pIV001-pIV008, 500 bp of left homologous arm flanking toward the 5′ end of gRNAs (primers MA28/MA43, on genomic DNA of *S. cerevisiae* YPH500 strain), yeast selectable markers *HIS3*/*URA3*/*KanMX*/*hphMX6*/*LEU2*/*TRP1*/*LYS2*/*natMX* (primers MA44-MA59, plasmids from lab collection), *MCS* introduced in the primers and 500 bp of right homologous arm flanking toward the 3′ end of gRNAs (primers MA60-MA75, on genomic DNA of *S. cerevisiae* YPH500 strain) were cloned into *Bam*HI-digested PUC19 vector, as described in Supplementary Table S3.

For the construction of the yeast-enhanced green fluorescent protein (yEGFP)-containing donor plasmids pIV009-pIV012, a cassette harboring the *TEF1* promoter, yEGFP, and the *CYC1* terminator (primers MA76/MA77, on IDT gBlock) was amplified, digested with *Nco*I*-BamHI*, and cloned into *Nco*I*-BglII*-digested plasmids pIV005-pIV008 (see also Supplementary Table S4 for corresponding regulatory elements, primers, plasmids, and genomic integration loci). The sequences were verified, digested with *Sal*I*-BamHI*/*Sma*I, and transformed into yeast strain YPH500 along with the respective gRNA plasmids to generate strains yMA001-yMA007. For conventional homologous recombination, the same linearized yEGFP donor fragments containing approximately 500-bp homology arms were also transformed into the YPH500 strain without the corresponding Cas9/gRNA helper plasmids. The transformants were selected on the respective combinations of yeast selection media.

### Flow Cytometry and Data Analysis

Yeast strains carrying *yEGFP* reporter constructs at specific loci were used to assess yEGFP expression at those loci. The set included wild-type (WT) strains, a strain with yEGFP under the constitutive *TEF1* promoter at *the ura3-52* locus, and strains with yeast yEGFP under the constitutive *TEF1* promoter at *YBR128C, YDR448W, YGR240C, and YHR142W loci*. Initial colonies were cultured in selective SC medium, then inoculated into YPDA medium for primary culture and incubated overnight (∼16 h) at 30 °C with agitation at 200 rpm. Secondary cultures were started at an initial OD_600_ of ∼0.1 in YPDA medium and grown for 4–6 h at 30 °C with 200 rpm. Cells were collected by centrifugation, washed with phosphate-buffered saline (PBS), and analyzed by flow cytometry using a Cytek Aurora CS cytometer to measure yEGFP fluorescence, with a minimum of 10,000 cells per sample. FlowJo was used to calculate the geometric mean of yEGFP fluorescence per cell.

### Design and Construction of the Naringenin Pathway

#### *yFASTOP-NG* Strain

For construction of the Naringenin pathway, the coding DNA sequences (CDSs) for genes *AtC4H:L5:AtATR2, PhCHI, HaCHS, At4CL-2, and AtPAL-2*, codon-optimized for *S. cerevisiae*, were used.

The *PGI1 t*erminator (primers MA78/MA79, on genomic DNA of *S. cerevisiae* YPH500 strain), *PhCHI* CDS (primers MA80-MA81, IDT gBlock9), *PGI1* promoter (primers MA82/MA83, on genomic DNA of *S. cerevisiae* YPH500 strain),

*FBA1* promoter (primers MA84/MA85, on genomic DNA of *S. cerevisiae* YPH500 strain), *AtC4H: L5:AtATR2* CDS (primers MA86/MA87, on IDT gBlock), *FBA1* terminator (primers MA88/MA89, on genomic DNA of *S. cerevisiae* YPH500 strain) were cloned into *Xho*I-digested yeast vector pIV006 for integration into the *YDR448W* locus, harboring yeast selection auxotrophic marker TRP1. The generated plasmid was called pIV013.

The *PGK1* terminator (primers MA90/MA91, on genomic DNA of *S. cerevisiae* YPH500 strain), *At4CL-2* CDS (primers MA92/MA93, on IDT gBlock), *PGK1* promoter (primers MA94/MA95, on genomic DNA of *S. cerevisiae* YPH500 strain),

*TDH3* promoter (primers MA96/MA97, on genomic DNA of *S. cerevisiae* YPH500 strain), HaCHS CDS (primers MA98/MA99, on IDT gBlock), *TDH3* terminator (primers MA100/MA101, on genomic DNA of *S. cerevisiae* YPH500 strain), were cloned into *Xho*I-digested yeast vector pIV008 for integration into the *YHR142W* locus, harboring yeast selection marker natMX. The generated plasmid was called pIV014.

The *TDH1* promoter (primers MA102/MA103, on genomic DNA of *S. cerevisiae* YPH500 strain), *AtPAL-2* CDS (primers MA104/MA105, on IDT gBlock), and *TDH1* terminator (primers MA106/MA107, on genomic DNA of *S. cerevisiae* YPH500 strain) were cloned into *Xho*I-digested yeast vector pIV005 for integration into the *YBR128C* locus, harboring yeast selection auxotrophic marker LEU2. The generated plasmid was called pIV015 (see also Supplementary Table S4 for corresponding regulatory elements, primers, plasmids, and genomic integration loci). Genes were integrated into yeast by transforming *Sal*I/*BamH*I-, *Sbf*I/*BamH*I-, and *Xba*I/*BamH*I-digested donor fragments from pIV013–15, respectively. Integration was facilitated by the plasmids HV007, pHV011, and pHV005, which express Cas9 and locus-specific gRNAs (see Results section). Transformants were selected on SD–Ura–Trp or SD–Ura–Leu medium and screened by colony PCR, followed by Sanger sequencing of the PCR amplicons.

#### yCEN-NG Strain

To construct the control strain, the five naringenin genes were assembled into two episomal plasmids. The plasmid pIV013 was modified to replace the 500 bp left homologous arm of the *YDR448W* locus with CEN/ARS (primers MA108/MA109, on IDT gBlock) cloned into the PCR-amplified yeast vector pIV013 (primers MA54/MA110, on pIV013) harboring the yeast selection auxotrophic marker TRP1 for episomal transformation into yeast. The generated plasmid was called pIV016. The plasmid pIV015 was modified to insert the *PGK1* terminator-*At4CL-2* CDS-*PGK1* promoter-*TDH3* promoter-*HaCHS* CDS-*TDH3* terminator (primers MA111/MA112, on pIV014), replace the 500 bp of left homologous arm of *YBR128C* locus with CEN/ARS (primers MA108/MA113, on IDT gBlock) cloned into PCR-amplified yeast vector pIV015 (primers MA52/MA114, MA115/MA110, on pIV015) harboring yeast selection auxotrophic marker LEU2. The generated plasmid was called pIV017. The plasmids pIV016 and pIV017 were episomally transformed into yeast, and transformants were selected on SD–Trp–Leu medium and screened by colony PCR, followed by Sanger sequencing of the PCR amplicons. The generated starin was called yCEN-NG.

### HPLC

Single colonies were inoculated into YPDA medium and grown overnight (∼16 h) at 30 °C at 200 rpm. The cultures were inoculated in 10 mL of YPDA medium to an OD_600_ of ∼0.2 and grown at 30 °C for 16 h. Samples were subjected to metabolite extraction from 10 mL of yeast cultures. The cultures were harvested by centrifugation at 1000*g* for 5 min at −20 °C, washed with 5 mL of prechilled 60% (v/v) methanol (−20 °C), then centrifuged at 1000*g* for 5 min at −20 °C. The cells were resuspended in 1 mL of prechilled 80% (v/v) methanol (−80 °C). Cells were transferred to 2 mL bead-beating tubes containing 0.5 mm beads and disrupted using a homogenizer (8000 rpm; 3 × 30 s cycles with 90 s rest), followed by incubation at −80 °C for 20 min and a second homogenization step (8000 rpm; 2 × 20 s cycles with 90 s rest). Lysates were collected and centrifuged at 1000*g* at −20 °C for 30 s; the supernatant was filtered through a 0.22 μm syringe filter, diluted 10-fold in methanol, and centrifuged at 10,000*g* for 10 min before analysis. Naringenin identity was additionally confirmed by LC-MS, with a retention time of approximately 5.7 min and detection of the [M – H]^−^ ion at *m*/*z* 271.061 by high-resolution MS. For quantification, the extracts were analyzed using an ACQUITY UPLC system (Waters) equipped with a diode array detector (DAD). The system was controlled using Empower 3 software (Waters). Chromatographic separation was performed on an Acquity HSS T3 C18 column (150 × 2.1 mm, 1.8 μm; Waters) at 55 °C with a flow rate of 0.4 mL min^–1^. The mobile phase consisted of water (solvent A) and acetonitrile (solvent B), both containing 0.1% formic acid. 5 μL of diluted extracts were separated using a 10 min linear gradient from 85 to 50% of solvent A. Naringenin was quantified from UV chromatograms at 285 nm using calibration curves generated from a commercial naringenin (Sigma-Aldrich) standard.

### Plate Reader Experiments and Growth Assay

Single colonies were inoculated into YNB complete medium (2% glucose + CSM) and grown overnight (∼16 h) at 30 °C with 200 rpm. The cultures were inoculated into 2 mL of YNB medium to an OD_600_ of ∼0.2 and grown at 30 °C in a microplate reader for the growth assay. OD_600_ was measured for 32 h. For YEGFP intensity measurements, secondary cultures were inoculated into 2 mL of YNB medium to an OD_600_ of ∼0.2 and grown for 6 h at 30 °C with 200 rpm. Cells were collected by centrifugation, washed with phosphate-buffered saline (PBS), and analyzed by a plate reader or for growth assays. The set included wild-type (WT) strains, a strain with yEGFP under the constitutive *TEF1* promoter at *the ura3-52* locus, and strains with yeast yEGFP under the constitutive *TEF1* promoter at *YBR128C, YDR448W, YGR240C, and YHR142W loci*.

### Statistics and Reproducibility

Statistical analyses were performed using Prism 10 (GraphPad Software). Data are presented as mean ± standard deviation (SD), unless otherwise indicated. Biological replicates were treated as independent experimental units, and technical replicates were averaged before statistical testing. Normality was assessed using the Shapiro–Wilk test. For Figure 3a, differences in yEGFP fluorescence between each Y-locus condition and the ura3-52 reference strain were analyzed using one-way ANOVA followed by Dunnett’s T3 multiple-comparisons test. For Figure 3b, differences between gRNA1 and gRNA2 across the corresponding genomic loci were analyzed using two-way ANOVA followed by Tukey’s multiple-comparisons test. Comparisons between two groups were performed using two-tailed unpaired Student’s *t* tests. A *p*-value <0.05 was considered statistically significant. The statistical tests and adjusted *p*-values for individual experiments are provided in the corresponding figure in the Supplementary Data.

## Results

### Design of the FASTOP Toolkit

To enable rapid, multiplexed engineering of transcriptionally active loci in *S. cerevisiae*, we developed FASTOP, a modular CRISPR-based platform for integrating up to eight DNA cassettes into predefined hot-spot genomic sites. The system builds on “Y” loci identified by Wu et al.; *YBR128C* on Chr II (*ATG14*, Autophagy-specific subunit of phosphatidylinositol 3-kinase complex I), *YDR448W* on chrIV (*ADA1*, Transcription coactivator of the ADA and SAGA transcriptional adaptor/histone acetyltransferase complexes), *YGR240C* on ChrVII (*PFK1*, α subunit of heterooctameric phosphofructokinase), *YHR142W* on ChrVIII (*CHS7*, protein of unknown function)^12^ and expands them with four additional intergenic “H” hot-spot loci: *H1* on ChrXV (between *ADH1* and *YOL085W-A*), *H2* on ChrXII (between *PDC1* and *STU2)*, H5 on ChrIII (between *PGK1* and *ADP1)*, H7 on ChrI (between *CDC19* and *CYC3*), characterized by Baek et al.^11^ No more than two of these loci are located on the same chromosome to minimize chromosomal instability.

To engineer the hot-spot loci, the FASTOP toolkit (Figure 1) includes two plasmid series: (i) integration vectors (pIVs) harboring ∼500 bp homology arms flanking the inset(s) of interest and a yeast selection marker, but lacking an origin of replication for yeast, enabling direct transformation of linear donor fragments; and (ii) high-copy 2 μ helper vectors (HVs) which encode constitutively expressed Cas9 and locus-specific gRNAs to improve the integration efficiency at the site of choice. For each “Y” site, two gRNAs were designed (see Materials and Methods), and for the “H” sites, we used the gRNAs designed by Baek et al. in pHV plasmids. Upon cotransformation, Cas9-induced double-strand breaks stimulate homology-directed repair, enabling precise integration of donor cassettes. The plasmid collection includes matched integration/helper pairs for all H- and Y-series loci, and in combination with our previously established pCOM002 and pCOM009 auxotrophic and dominant marker-recycling system^9^ to permit iterative rounds of genome engineering.

**Figure 1:**
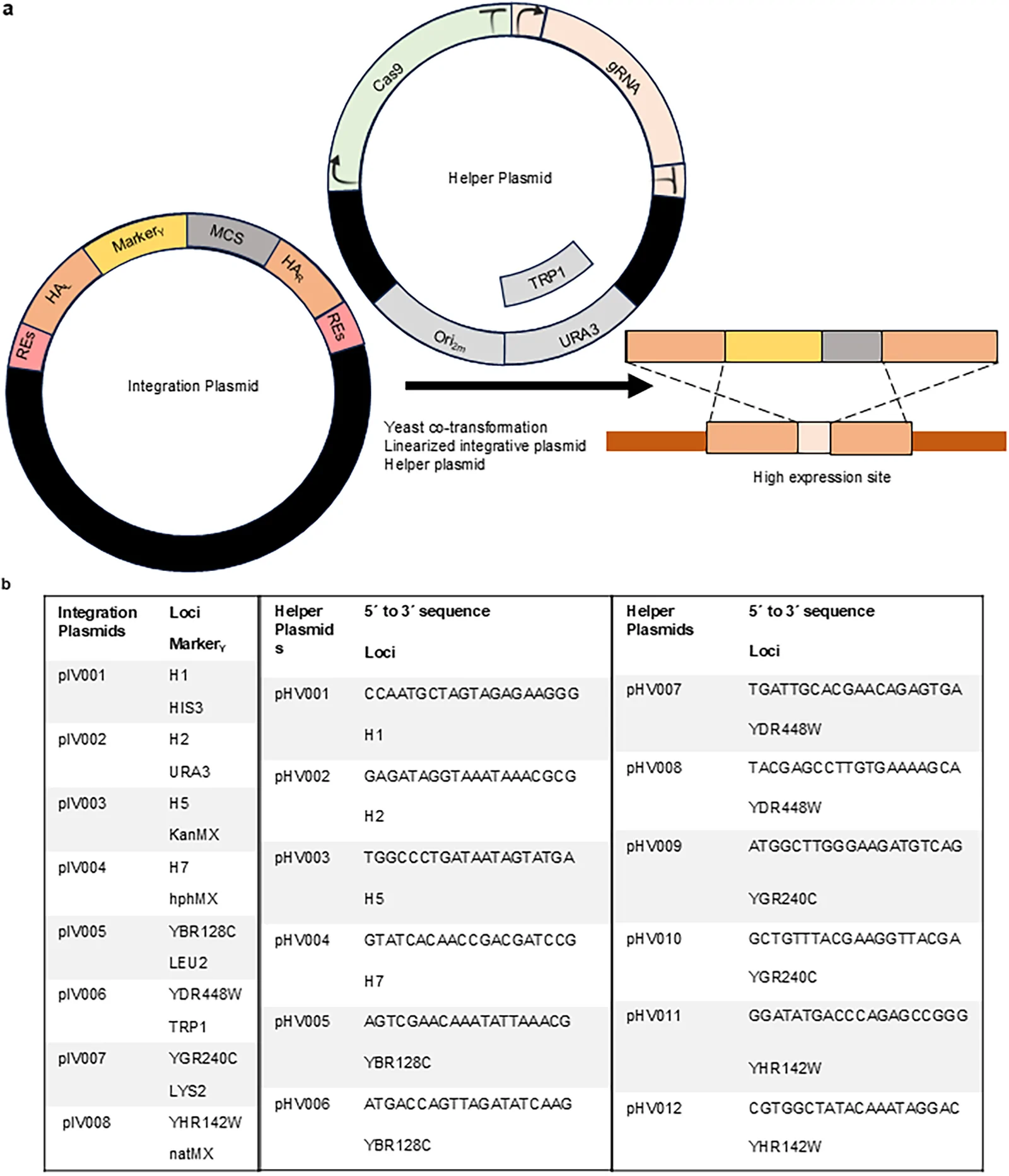
FASTOP toolkit for CRISPR-mediated genomic integration in yeast. **a** Integration vectors contain ∼500 bp left and right homology arms flanking a donor cassette with a selectable marker, enabling targeted insertion into predefined chromosomal loci. Episomal helper vectors express locus-specific gRNAs, controlled by the commonly used constitutive RNA polymerase III promoter SNR52, to achieve stable and efficient guide RNA expression in S. cerevisiae, and constitutively express Cas9 using the *TEF1* promoter. After cotransformation of the linearized donor DNA and the corresponding helper vector into yeast cells, targeted integration occurs using a gRNA per locus, and selection markers allow rapid screening of positive integrants. **b** FASTOP plasmid collection. The integration vector series carries donor cassettes with locus-specific homology arms and selectable markers. The helper vector series encodes the matching guide RNA for each hot-spot. Corresponding vector pairs enable modular and multiplex genome engineering. Abbreviations: REs, multiple restriction enzyme sites; L-HA and R-HA, left and right homology arms; MCS, multiple cloning site; Cas9, CRISPR-associated endonuclease 9; gRNA, guide RNA; Ori_2 μ_, 2-μm origin of replication; pIV, integration vector; pHV, helper vector; HIS3, URA3, LEU2, LYS2, yeast auxotrophic markers; KanMX, hphMX, natMX, dominant markers.

### Assessment of the Integration Efficiency into Hot-Spot Loci Using the FASTOP Toolkit

Baek et al. (2021) previously characterized four H-series loci for integration efficiency using CRISPR/Cas9 editing methodology.^11^ Here, to enable CRISPR-mediated targeting of the Y-series loci, we designed two gRNAs for each site (see Materials and Methods) and cloned them into helper plasmids. A yEGFP expression cassette was cloned into the *MCS* of the integration vectors. Because PCR amplification can introduce sequence errors,^13^ particularly when amplifying long DNA fragments, donor cassettes were generated by restriction enzyme digestion of the integration vectors rather than by PCR amplification. Following cotransformation of linearized donor DNA (upon digestion of integration vectors) and the corresponding helper vector, growing clones on selective media were screened for GFP fluorescence. To determine the integration efficiency, GFP fluorescence of 20 randomly selected colonies per locus was quantified using a plate reader. WT cells were included to determine the basal fluorescence level. We considered colonies with fluorescence signals above the WT threshold to be correct integrants. Because the measured bulk fluorescence can be affected by cell density and partial integration, integration efficiency was confirmed in randomly selected colonies using colony PCR. Across tested Y-series loci, integration efficiencies ranged from 75 to 100% (Figure 2), demonstrating robust CRISPR-assisted cassette integration at these highly transcriptionally active genomic sites. However, at the *YGR240C* locus, we obtained only a few colonies using CRISPR-assisted integration. In contrast, the conventional homologous recombination strategy using the same extended homology arms produced sufficient transformants at this locus. To determine whether this effect was locus-dependent, we also tested homologous recombination at *YBR128C*, *YDR448W*, and *YHR142W*; high integration efficiencies were obtained at *YBR128C* and YDR448W (Supplementary Figure S1), similar to CRISPR-assisted integration. In contrast, no colonies were recovered at *YHR142W* using homologous recombination, whereas CRISPR-assisted integration produced sufficient transformants at this locus. Together, these results demonstrate that the contribution of CRISPR/Cas9 to integration efficiency is locus-dependent and reduced Cas9 accessibility at YGR240C remains a possible explanation for the low recovery, in agreement with previous reports on the effect of chromatin structure, particularly nucleosome occupancy, on Cas9 targeting and cleavage efficiency.^20,21^ Importantly, these locus-dependent outcomes show that the same FASTOP integration vectors can be used with CRISPR/Cas9 assistance or by conventional homologous recombination. As one strategy may perform better than the other depending on the target locus, providing both options increases the flexibility of the toolkit to improve the chance of successful integration.

**Figure 2:**
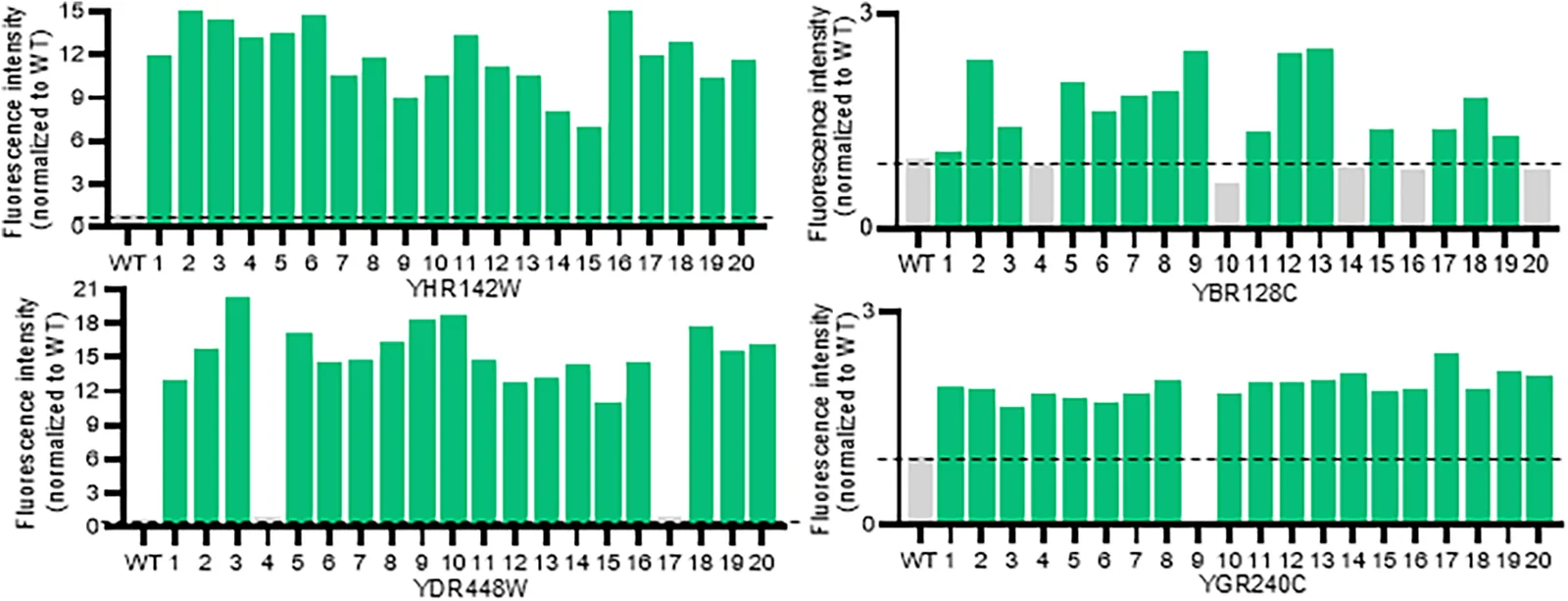
Efficiency of donor integration into hot-spot “Y” loci. For integration into *YHR142W*, *YBR128C*, and *YDR448W*, CRISPR-assisted cassette integration was used, while for *YGR240C*, native homologous recombination was used. To assess the integration efficiency, yEGFP fluorescence intensity was measured by a plate reader in bulk cultures of 20 randomly selected colonies per locus. *YHR142W*, *YBR128C*, *YDR448W*, and *YGR240C* correspond to the Y sites.^12^ The WT yeast strain was used to determine the basal fluorescence level (horizontal baseline). Colonies showing fluorescence above the WT baseline were counted as correct integrants. Gray bars indicate transformants whose fluorescence was not more than that of the wild-type control, whereas green colored bars represent colonies exhibiting fluorescence above background. Abbreviations: WT, Wild-type YPH500; yEGFP, yeast-enhanced green fluorescent protein. Full data are shown in Supplementary Data S1.

### Characterization of Hot-Spot Loci for Gene Expression

Baek et al. (2021) previously characterized “H” sites for high transcriptional output.^11^ Here, to evaluate the transcriptional performance of “Y” genomic hot-spot loci, the fluorescence intensity of a yEGFP expression cassette of verified positive integrants at “Y” loci was quantified at the single-cell level and compared with that at the *ura3-52* locus, which served as a previously characterized reference with high expression. Strain YPH500 was used as a negative control to determine background fluorescence levels (Supplementary Figure S2a). As shown in Figure 3a, the “Y” integration sites using CRISPR-mediated genome editing supported strong GFP expression, with *YBR128C*, *YDR448W*, and *YGR240C* showing fluorescence intensities similar to those observed at *ura3-52*. In contrast, mean fluorescence at *YHR142W* was lower than that of the other “Y” loci and the *ura3-52* reference. Importantly, no significant differences were observed between the two gRNAs tested at each locus (Supplementary Figure S3a), indicating that the observed expression differences were primarily locus-dependent rather than gRNA-dependent. However, single-cell analysis (Figure 3b) revealed greater heterogeneity for the yEGFP cassette integrated into YGR240C via CRISPR genome editing, with approximately 8% GFP-positive cells, and flow cytometry analysis revealed two distinct subpopulations, including a WT-like population and a GFP-positive population (Supplementary Figure S2b). Such locus-dependent differences in reporter expression are consistent with previous reports about the chromosomal position effects in *S. cerevisiae*, highlighting that transcriptional output depends on the genomic context of the integration site,^22,23^ and align with our earlier finding (Figure 2) demonstrating that CRISPR-mediated integration was not successful at this locus, due to likely limited Cas9 accessibility to local chromatin. Using homologous recombination to integrate the yEGFP cassette into *YGR240C* resulted in significantly lower expression levels than CRISPR-mediated genome editing (Figure 3a and Supplementary Figure S3b), but it yielded a hemizygous population (approximately 98% yEGFP-positive cells) (see Figure 3b and Supplementary Figure S3c).

**Figure 3:**
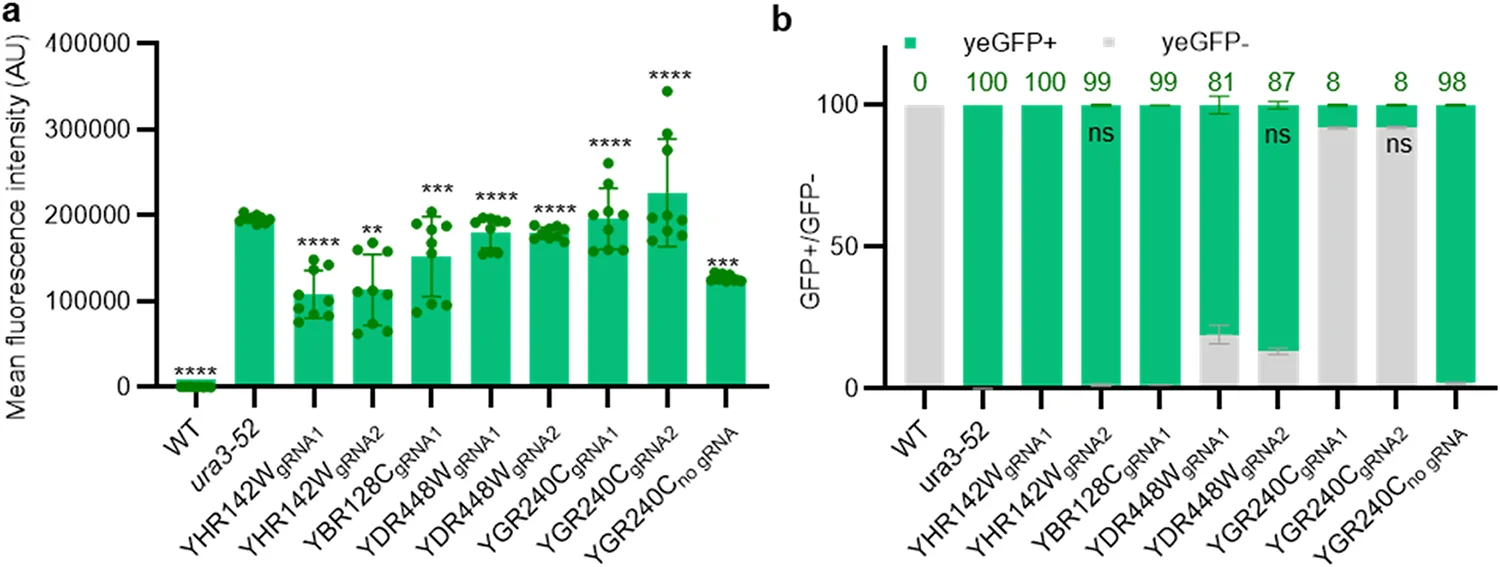
Effect of hot-spot “Y” genomic loci on fluorescent reporter expression in yeast. **a** Transcriptional output of fluorescent protein integrated into the “Y” loci. Cells were transformed with the yEGFP expression cassette for integration into “Y” sites. In the case of CRISPR-based genome editing, for each site, two gRNAs were selected. yEGFP expression in each transformant was measured by flow cytometry, with the *ura3-52* locus selected as a representative of a high-expression site^9^ for comparison. WT YPH500 was used as a negative control. *YBR128C*, *YGR240C*, *YDR448W*, and *YHR142W* correspond to the “Y” integration sites.^12^ Data represent geometric mean fluorescence intensity ± SD from three biological replicates, each with three technical replicates. **b** Percentage of GFP-positive to GFP-negative cells determined by flow cytometry. Data represent the mean number of yEGFP-positive and yEGFP-negative cells ± SD from three biological replicates, as determined by flow cytometry FACS. Green numbers represent the percentage of GFP-positive cells. Abbreviations: FACS, Fluorescence-Activated Cell Sorting; yEGFP, yeast-enhanced green fluorescent protein; gRNA, guide RNA; WT, wild type. Full data and statistical analysis are shown in Supplementary Data S2.

Baek et al. (2021) previously showed that none of the “H” sites selected in this study showed a measurable difference in mean growth rate compared to the WT YPH500 strain.^11^ Here, we examined whether the presence of heterologous DNA in the target “Y” loci could result in any impairment of the growth rate of yeast. The impact of heterologous DNA integration on yeast fitness was examined by measuring the growth rates of each yEGFP-integrated strain in YPDA over 32h, compared to the WT strain and to the commonly used high integration reference site *ura3-52* (Supplementary Figure S4). Integration at *YBR128C* and *YGR240C* resulted in severe growth defects relative to the WT strain, although these strains eventually reached maximum OD_600_ levels comparable to the control. These effects are consistent with the endogenous functions of these loci, as *YGR240C* (*PFK1*) encodes the key enzyme in glycolytic regulation, and *YBR128C* (*ATG14*) encodes a component involved in autophagy and cellular homeostasis. Thus, integration at these loci may impose a fitness burden by perturbing essential cellular processes. In contrast, integration at the *YDR448W* and *YHR142W* loci resulted in modest alterations in growth dynamics, comparable to those observed at *ura3-52*. Together, our results suggest that the hot-spot sites provide practical genomic integration sites for high expression of heterologous genes in yeast. However, our findings emphasize that selecting genomic integration hot-spots must consider not only transcriptional strength but also their influence on cellular fitness (Table 2).

**Table 2:** Practical Comparison of Y-series Genomic Integration Loci Based on Integration Efficiency, Expression Output, Population Homogeneity, and Fitness Cost

Locus	Integration/recovery	GFP expression	Population homogeneity	Fitness	Practical guidance
**YDR448W**	90% by CRISPR; 95% by HR	High, ∼177–179 × 10^3^ AU	Moderate, ∼81–87% GFP+	Modest growth alteration	Best overall choice, particularly when high expression is required
**YHR142W**	Excellent with CRISPR; 20/20 positive; no colonies with HR alone	Lower, ∼100–112 × 10^3^ AU	Very high, ∼99% GFP+	Modest growth alteration	Preferred when homogeneity and fitness are priorities; use CRISPR-assisted integration
**YBR128C**	75% by CRISPR; 100% by HR	High, ∼149 × 10^3^ AU	Very high, ∼98.6% GFP+	Pronounced growth defect	Useful for expression studies, but not preferred when fitness is important
**YGR240C**	Poor colony recovery with CRISPR; HR gives sufficient transformants	Very high with CRISPR (∼207–210 × 10^3^ AU); low with HR (∼128 × 10^3^ AU)	Only ∼8% GFP+ with CRISPR; ∼98% GFP+ with HR	Pronounced growth defect	Lowest-priority site; if required, HR is preferable to CRISPR

### Naringenin Production in Yeast Using the FASTOP Toolkit

Naringenin is synthesized from phenylalanine and Malonyl-CoA via five enzymatic steps (*AtPAL-2, AtC4H:L5:AtATR2*, *At4CL-2, HaCHS, and PhCHI*; Figure 4a).^24^ For a proof-of-concept study, the expression cassettes for yeast codon-optimized AtC4H:L5:AtATR2 and PhCHI, HaCHS, At4CL-2, and AtPAL-2 were integrated into, respectively, the YDR448W, YHR142W, and YBR128C loci to generate strain yFASTOP-NG (Figure 4b; see also the Methods section). Genetic stability of yFASTOP-NG was confirmed after repeated passaging by overlapping diagnostic PCRs covering the complete integration regions. Under simple batch flask culture conditions, after 16 h (mid log-phase, Supplementary Figure S5), the engineered strain produced an intracellular naringenin accumulation of 505.7 mg L^–1^ (Figure 4c, also see Supplementary Figure S6). This titer was approximately 4.5-fold higher than previously reported ∼112.9 mg L^–1^ using additional precursor and pathway copy-number engineering in a controlled batch bioreactor (see Supplementary Table S5 for further details).^25^ In contrast, FASTOP achieved 505.7 mg L^–1^ in simple shake-flask culture without additional host metabolic engineering. We also showed that the FASTOP-integrated strain produced approximately 6-fold more naringenin than the episomal expression system under identical cultivation conditions (Figure 4c), demonstrating the advantage of chromosomal integration at selected hot-spot loci for high-level metabolite production. Importantly, despite strong metabolite production, pathway integration at the selected FASTOP loci resulted in slower growth, and biomass ultimately reached a level comparable to that of the WT strain (Supplementary Figure S5). These results showed that FASTOP-mediated engineering of hot-spot loci can significantly reduce the need for further metabolic engineering strategies such as endogenous pathway rewiring or precursor supply optimization, while still enabling high-level production from a minimal gene set.

**Figure 4:**
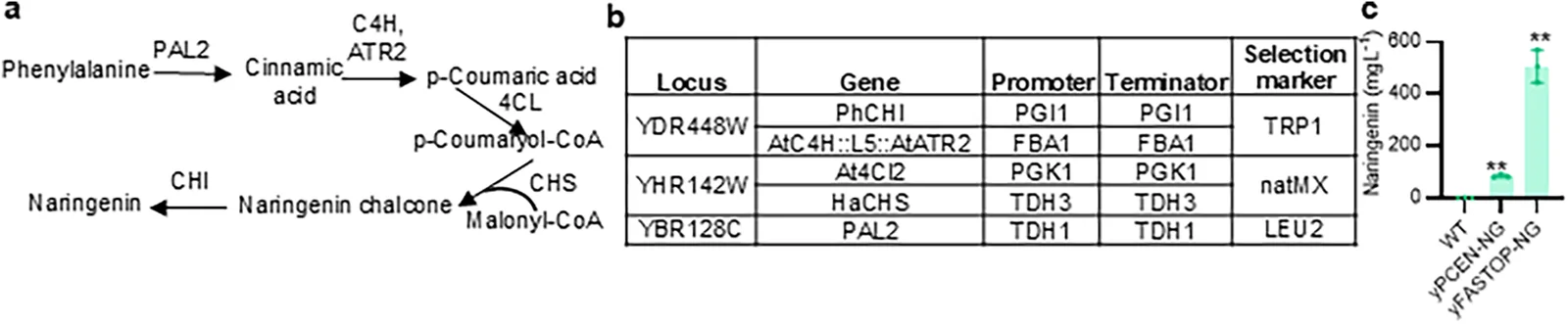
Naringenin production in yeast using the FASTOP toolkit. **a** Schematic illustration of the heterologous naringenin biosynthetic pathway introduced into *Saccharomyces cerevisiae*. Naringenin is synthesized from phenylalanine and malonyl-CoA via five enzymatic steps: AtPAL2, AtC4H:L5:AtATR2, At4CL, HaCHS, and PhCHI. **b** Genomic integration strategy for pathway assembly using FASTOP hot-spot loci. The biosynthetic genes, driven by constitutive promoters, were chromosomally integrated into the *YBR128C*, *YHR142W*, and *YDR448W* loci to generate strain yFASTOP-NG. **c** Naringenin production in strain yFASTOP-NG. Intracellular naringenin accumulation (mg L^–1^) was quantified at the midexponential phase of cells grown in a batch shake-flask. As controls, yeasts WT and yCEN-NG, harboring two episomal plasmids encoding the pathway genes under the control of the same promoters, were used. Data are presented as mean ± SD from three biological replicates. Asterisks indicate a significant difference from the WT (Student’s *t* test; ∗∗*p* < 0.01). Abbreviations: AtPAL2, phenylalanine ammonia lyase from *Arabidopsis thaliana*; C4H, *Arabidopsis thaliana* cinnamate-4-hydroxylase; ATR2, *Arabidopsis thaliana* cytochrome P450 reductase; 4CL, *Arabidopsis thaliana* 2-coumarate: CoA ligase; HaCHS, *Helianthus annuus* chalcone synthase; PhCHI, *Petunia hybrida* Chalcone isomerase; WT, wild-type. Full data sets are provided in Supplementary Data S3.

## Discussion

The development of reliable genome-engineering strategies that enable predictable, high transcriptional output is of high interest for advancing yeast production of food- and agriculture-relevant compounds. This is mainly important as increasing gene copy number, although effective, often introduces instability, recombination risks, and metabolic burden.^26^ Hence, integration into naturally active chromosomal environments represents a more stable and streamlined alternative for enhancing pathway expression. Here, we developed FASTOP, a modular CRISPR-based toolkit for rapid engineering of transcriptionally active genomic loci of yeast.

Over the past decade, several modular genome engineering toolkits have been developed for *S. cerevisiae*. The EasyClone platform^27^ established standardized chromosomal integration at defined genomic loci, while subsequent developments, including EasyClone-MarkerFree,^28^ EasyClone 2.0,^29^ CasEMBLR and CrEdit,^30,31^ incorporated CRISPR/Cas9-mediated markerless genome editing, adding more integration sites and multiplex pathway assembly to expand applicability to industrial strains. More recently, the EasyClone-MarkerFree platform was extended by validating eight additional chromosomal integration sites exhibiting high integration efficiencies and stable transgene expression.^32^ Shaw et al. further developed a modular Golden Gate-compatible CRISPR/Cas9 toolkit comprising ten intergenic genomic integration loci selected based on engineering criteria, including genomic stability, multiplex compatibility, and editing accessibility.^33^ In contrast, FASTOP builds upon previously identified transcriptionally active H- and Y-series loci, thereby focusing on genomic hot-spot sites with high expression potential while integrating them into a standardized modular cloning platform with extended homology arms and optional CRISPR/Cas9-assisted genome editing. A notable feature of FASTOP is the elimination of PCR during donor cassette preparation. PCR amplification is widely used in yeast genome engineering, but can introduce point mutations, rearrangements, or sequence bias, particularly when handling long or repetitive constructs, such as those used in engineering projects that rely on synthetic transcription factors and therefore have multiple copies of core binding sites upstream of pathway genes.^9,34^ In these cases, restriction-based cassette release improves sequence fidelity, which becomes particularly important as pathway size and complexity increase. Although FASTOP’s modular multiple cloning site provides flexibility in restriction enzyme selection, internal restriction sites within the inserted DNA cannot always be avoided. In such cases, users may implement PCR-based donor cassette preparation, or remove internal restriction sites during construct design. In summary, these features were designed to make the FASTOP toolkit usable in laboratories with varying levels of technical expertise for genome editing in yeast.

While previous genome-wide analyses have revealed strong positional effects on transcription in yeast, only a limited subset of loci has been experimentally characterized for engineering applications. By analytically evaluating the integration efficiency and expression capacity of previously unassessed hot-spot sites, this study provides a practical framework for pathway assembly in the *S. cerevisiae* genome. Here, we validated the four Y-series loci as robust genomic environments for high gene expression. The comparable fluorescence output observed between the “Y” loci and *ura3-52*, a commonly used reference locus,^9^ demonstrates that these regions support high transcriptional activity without requiring multicopy integrations. However, our finding also showed that not all transcriptionally active loci are equally suitable for pathway engineering, as integration at *YGR240C* and *YBR128C* was associated with reduced growth.

Population homogeneity also varied among the loci (see Table 2). Because correct integration was confirmed by PCR and the integrated cassettes were present in the genome after repeated passaging, the observed heterogeneity is unlikely to result from cassette loss. However, the present data do not distinguish between heterogeneous editing outcomes, transcriptional silencing, and chromosomal position effects.

Selection of genomic integration sites should be evaluated for both transcriptional output and potential effects on host fitness to achieve optimal production performance, although such trade-offs may be less critical for short-term proof-of-concept or gene expression studies. Overall, YDR448W and YHR142W showed the most balanced performance, whereas YBR128C and YGR240C should be used with particular attention to their associated fitness and integration limitations (see Table 2 for a practical comparison of the Y-series loci).

At *YHR142W*, no transformants were recovered using homologous recombination alone, whereas CRISPR/Cas9-assisted integration produced sufficient transformants. In contrast, at *YGR240C*, conventional homologous recombination resulted in more transformants than CRISPR/Cas9-assisted integration. These results suggest that chromatin accessibility may be an important factor in editing performance, consistent with prior reports that chromatin structure, particularly nucleosome occupancy, can influence Cas9 activity.^20,21^ FASTOP allows the same extended-homology-arm donor constructs to be used with either CRISPR/Cas9 assistance or conventional homologous recombination, providing alternative integration strategies depending on the target locus.

The toolkit provides a set of plasmids for engineering eight host-gene genomic sites. Combined with our previously developed marker recycling systems,^9^ FASTOP-mediated genomically integrated auxotrophic and dominant selection markers can be efficiently inactivated. As a result, it enables the reuse of FASTOP principles for engineering additional hot-spots “H”^11^ and “Y”^12^ for the construction of complex biosynthetic pathways.

Naringenin is a plant-derived flavonoid with well-established antioxidant and anti-inflammatory activities and is used in nutraceuticals and dietary supplements.^14,15^ The successful reconstruction of the naringenin biosynthetic pathway, integrated into three hot-spot sites, resulted in 505.7 mg/L naringenin production, representing an approximately 4.5-fold increase compared with previous reports in a controlled batch bioreactor.^25^ Notably, FASTOP achieved this titer in simple shake-flask culture and without additional host metabolic engineering. These data indicate that transcriptionally active genomic environments can support high-level pathway expression without imposing severe fitness costs, suggesting that hot-spot genome integration can potentially reduce the need for extensive host metabolic rewiring or precursor engineering. Distributing pathway modules across separate loci may also reduce transcriptional interference between regulatory elements.

Despite these advantages, further advancing the toolkit requires characterizing hot-spot loci under diverse cultivation conditions, characterizing the transcriptional strength of various promoters and terminators integrated into hot-spot loci, and expanding the number of hot-spot loci. In summary, FASTOP establishes a practical platform for targeted integration into transcriptionally active regions of the yeast genome. It provides a valuable synthetic biology tool for metabolic engineering applications in the sustainable production of food, nutraceutical, and agriculturally relevant compounds.

## Supplementary Material



## Data Availability

The authors declare that the data supporting the findings of this study are available within the paper and its Supporting Information files. Should any raw data files be needed in another format, they are available from the corresponding author upon reasonable request. The FASTOP plasmids reported in this study are available from Addgene (www.addgene.org) under the following ID numbers. Integration plasmids: pIV001, #256762; pIV002, #256763; pIV003, #256764; pIV004, #256765; pIV005, #256766; pIV006, #256767; pIV007, #256768; pIV008, #256769; pIV0013, #261110; pIV0014, #261111; pIV0015, ##261112; and helper plasmids pHV001, # 261103; pHV002, # 261104; pHV003, # 261105; pHV004, # 261106; pHV005, # 261107; pHV007, #256770; pHV009, # 261108; pHV011, # 261109.

## Notes

Donor integration efficiency, flow cytometry analysis, transcriptional output, growth kinetics, and naringenin analysis (Figures S1–S6); plasmid sequences, primer sequences, expression cassettes with corresponding regulatory elements and primers, and naringenin production comparison table (Tables S1–S5); and underlying numerical data and statistical analyses for the main and supplementary figures (Data S1–S7) (PDF)
