# Supplementary material for: FASTOP—Fast Editing Toolkit for Top Expression Sites in Yeast
Source: J Agric Food Chem. 2026 Oct 21;74(43):34106–15. doi: 10.1021/acs.jafc.6c06735 (PMC13741906; doi:10.1021/acs.jafc.6c06735)
Supplement: Supplementary file 1 [file jf6c06735_si_001.pdf]

FASTOP - Fast editing toolkit for top expression sites in yeast

Madhushruti Borah[1], Nicolas Gautron[2], Vincent Courdavault[2], Gita Naseri[1,3,*]

1. Institut für Biologie, Humboldt-Universität zu Berlin, Philippstrasse 13, 10115 Berlin, Germany

2. EA2106 Biomolécules et Biotechnologies Végétales, Université de Tours, 31 avenue Monge, 37200 Tours, France

3. Max Planck Unit for the Science of Pathogens, Charitéplatz 1, 10117 Berlin, Germany.

*Corresponding author

Gita Naseri, Email: gita.naseri@hu-berlin.de

Supplementary Figures

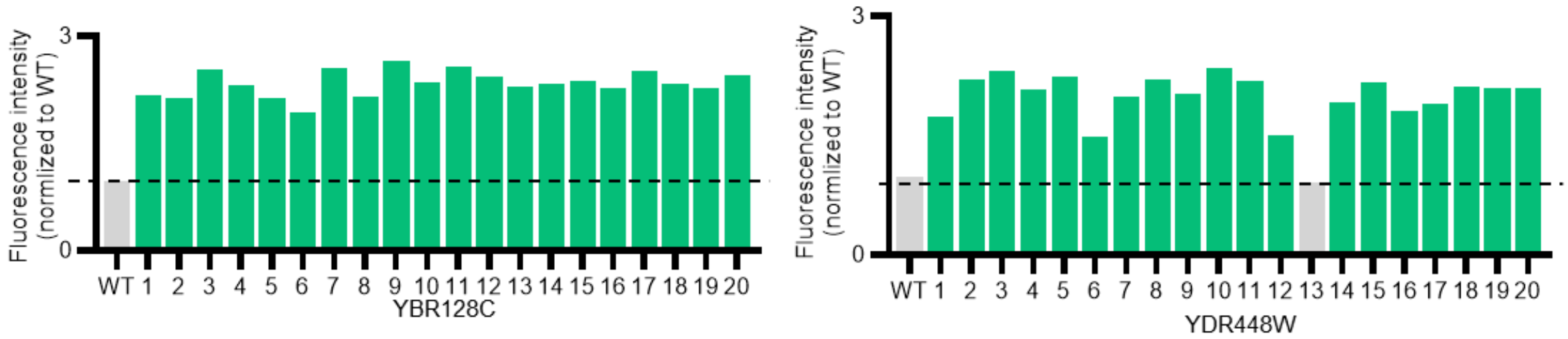

Supplementary Figure S1. Efficiency of donor integration into YBR128C and YDR448W loci in the absence of CRISPR-assisted integration. Using native homologous integration, donor cassettes were integrated. To assess the integration efficiency, yEGFP fluorescence intensity was measured for 20 randomly selected colonies per locus using a fluorescence plate reader. *YBR128C* and *YDR448W* correspond to the Y integration sites[12]. The WT yeast strain was used to determine the basal fluorescence level. Colonies showing fluorescence above the WT baseline were counted as correct integrants. Grey bars indicate transformants whose fluorescence was not more than that of the wild-type control, whereas green colored bars represent colonies exhibiting fluorescence above background. Abbreviations: WT, Wild-type YPH500; yEGFP, yeast-enhanced green fluorescent protein. Full data are shown in **Supplementary Data S4**.

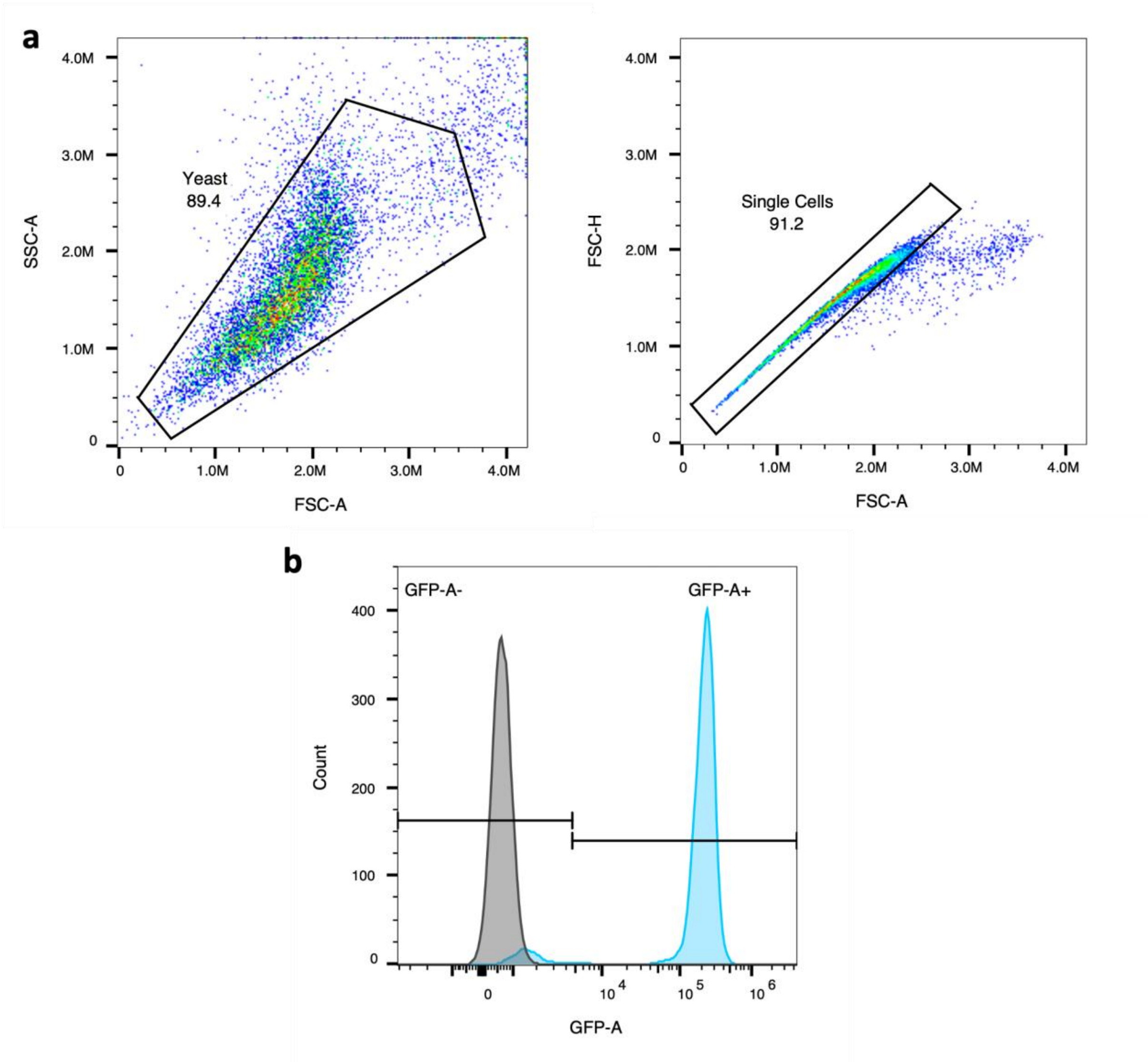

Supplementary Figure S2. Flow cytometry analysis. a Gating strategy. Yeast populations were identified by plotting SSC vs. FSC on a log scale. The gated population (within the outlined area) was used for all subsequent fluorescence measurements. **b** Distribution of fluorescent-positive and fluorescent-negative cells for integration loci. Single colonies grown overnight were inoculated into main cultures for 4–6 h (30°C and 220 rpm). yEGFP fluorescence was measured with a minimum of 10,000 cells per sample. The grey histogram represents the WT, and the blue histogram represents the distribution of positive and negative yEGFP fluorescence. Abbreviations: SSC, side scatter; FSC, forward scatter; WT, wild type; yEGFP, yeast-enhanced green fluorescent protein.

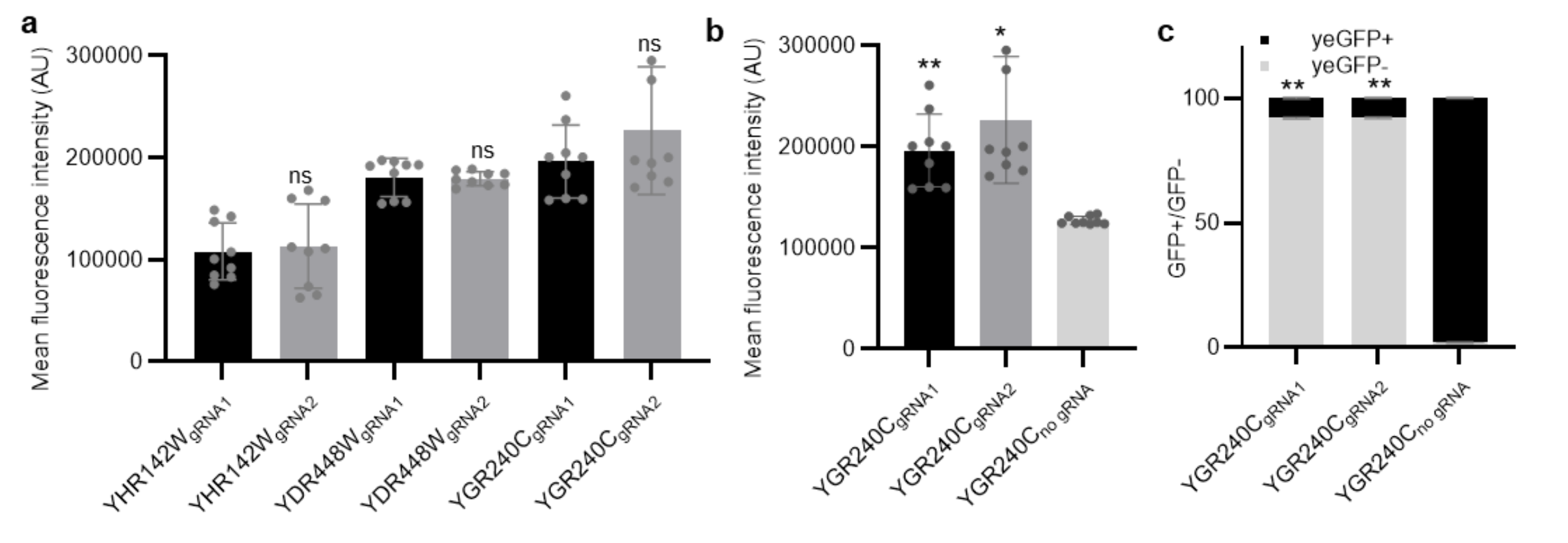

Supplementary Figure S3. Transcriptional output differences for CRISPR-assisted integration into the "Y" loci using different gRNAs. Cells were transformed with the yEGFP expression cassette to be integrated into "Y" sites. For each site, two gRNAs were selected. yEGFP expression was next measured by flow cytometry FACS. *YGR240C*, *YDR448W*, and *YHR142W* correspond to the "Y" integration sites[12]. Data represent geometric mean fluorescence intensity ± SD from three biological replicates, each with three technical replicates. **a** There was no significant difference between the two gRNAs (Student's t-test; ns, non-significant). **b** Asterisks indicate a significant difference in yEGFP expression level from the condition without gRNA. **c** Asterisks indicate a significant difference for yEGFP-positive cells from the condition without gRNA (Student's t-test; $*p < 0.05$; $**p < 0.01$; ns, non-significant). Abbreviations: FACS, Fluorescence-Activated Cell Sorting; yEGFP, yeast-enhanced green fluorescent protein; gRNA, guide RNA; WT, wild type. Full data and statistical anaylsis are shown in **Supplementary Data S5**.

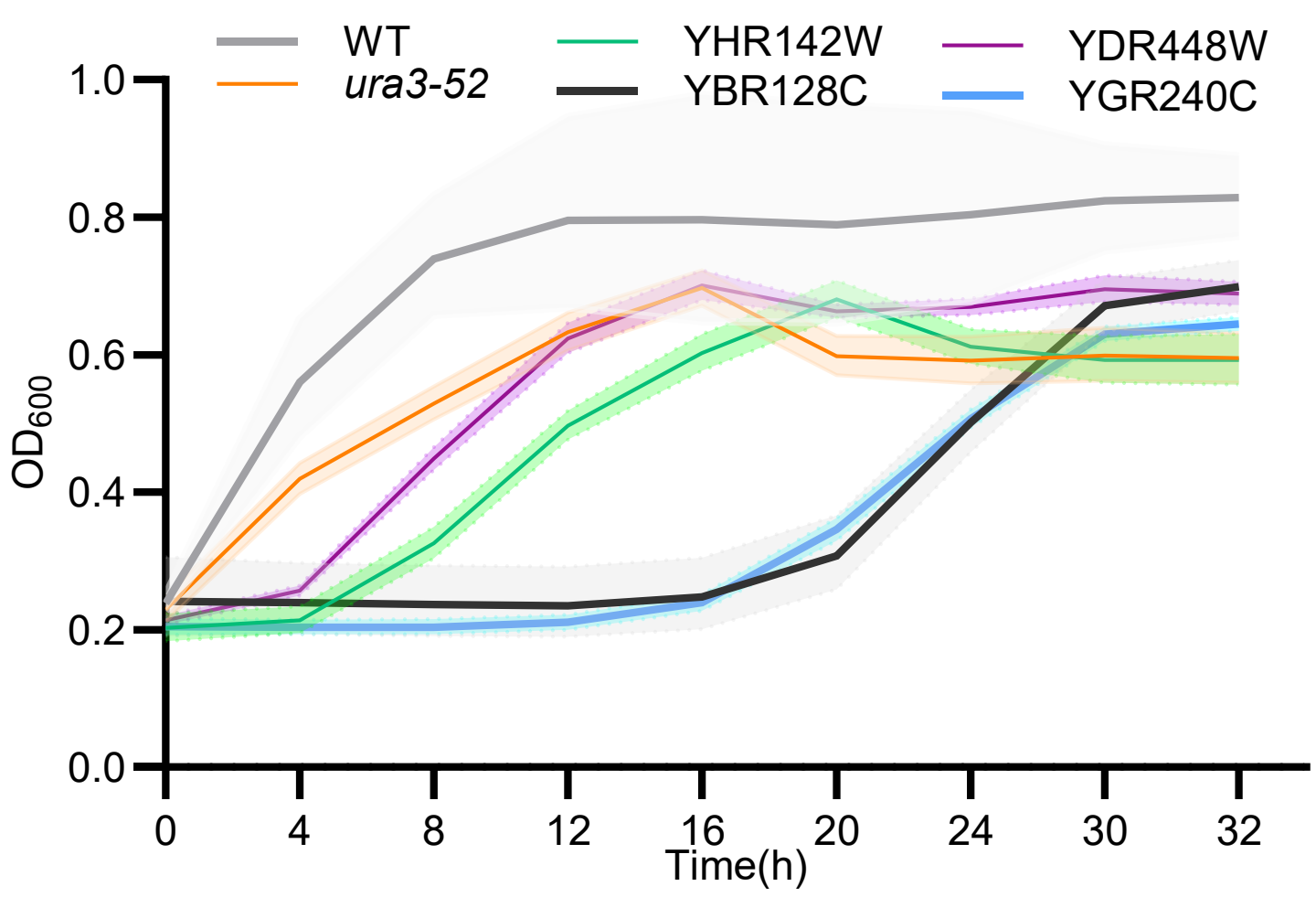

Supplementary Figure S4. Growth kinetics of yeast strains carrying yEGFP reporters integrated at genomic hot-spot loci. OD_{600} was measured over 32 h in YPDA for WT and yEGFP-integrated hot-spot loci strains. *YHR142W*, *YBR128C*, *YDR448W,* and *YGR240C* are hot-spot "Y" loci. As controls, WT and a strain harboring the yEGFP cassette integrated into the *ura3-52* site were used. Measurements were obtained from three independent colonies per strain and are shown as mean ± SD. Abbreviations: yEGFP, yeast enhanced GFP; OD_{600}, optical density at 600 nm; h, hour. Full data are shown in **Supplementary Data S6**.

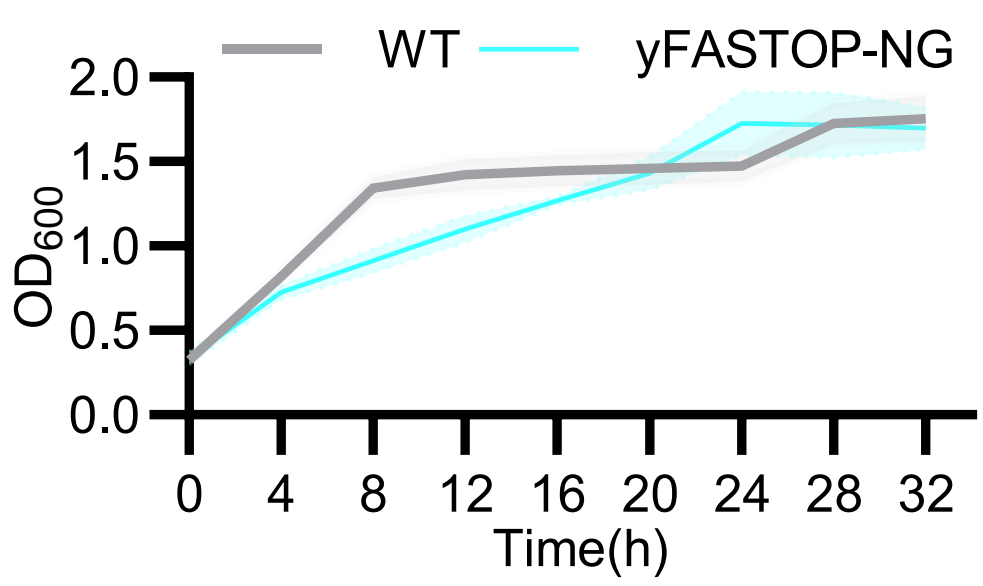

Supplementary Figure S5. Time-course analysis of the growth of the naringenin-producing strain. The engineered strain was cultivated under batch conditions, and OD_{600} was monitored over time. Yeast YPH500 was used as the control WT. Data are presented as mean ± SD from three biological replicates. Asterisks indicate a significant difference from the

WT (Student's t-test; ∗p < 0.05; ∗∗p < 0.01; ns, non-significant). Abbreviations: WT, wild-type; OD_{600}, optical intensity at 600 nm; h, hour. Full datasets are provided in **Supplementary Data S7**.

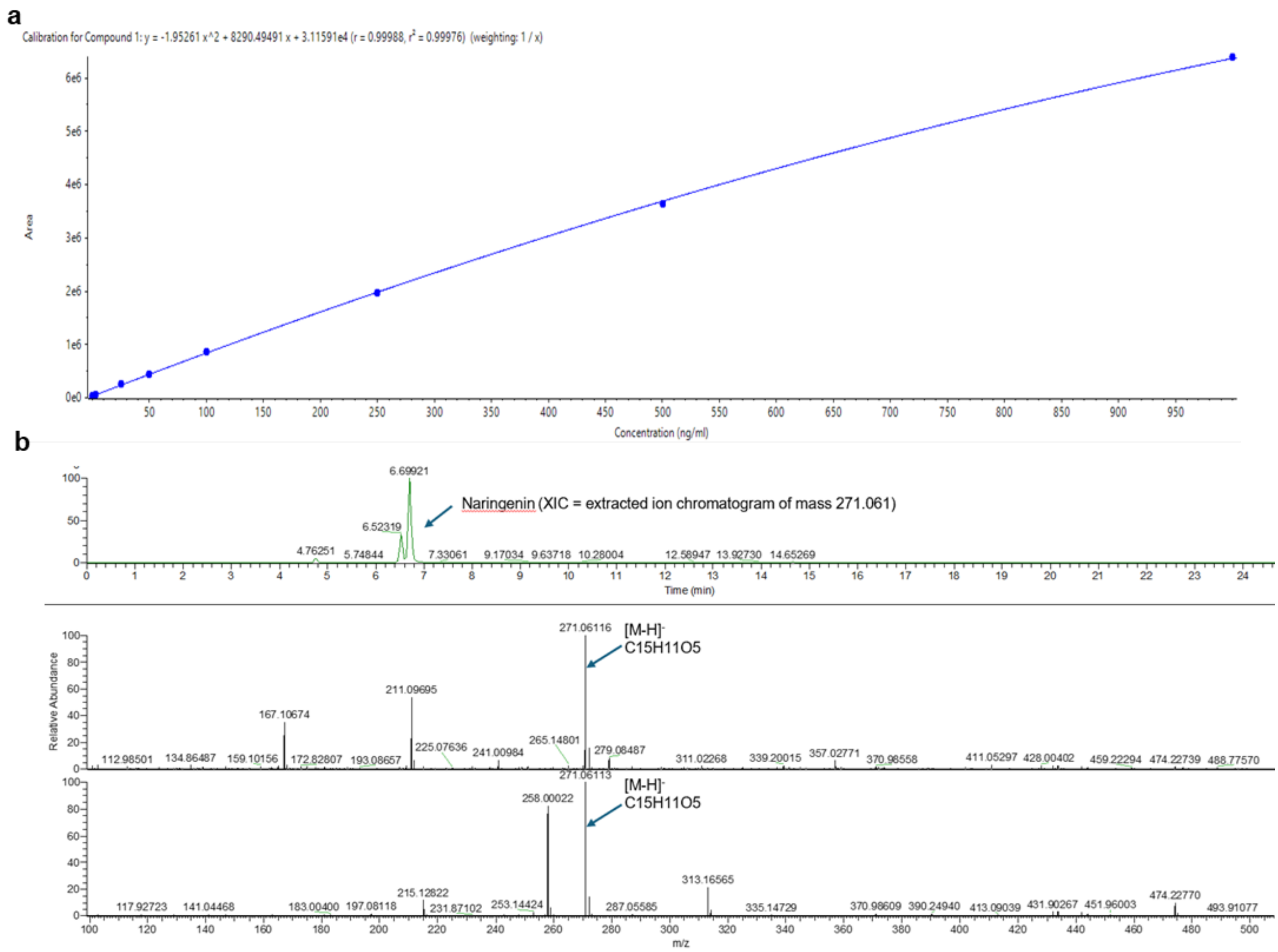

Supplementary Figure S6. Method for naringenin analysis. a Naringenin calibration curve. **b** High-resolution LC-MS confirmation of naringenin. The extracted ion chromatogram in negative electrospray ionization mode showed a peak at m/z 271.061, corresponding to the deprotonated naringenin molecular ion $[M-H]^-$.

Supplementary Data

Supplementary Data S1 Figure 2

Samples	YBR128C	YDR448W	YGR240C	YHR142W
WT	1	1	1	1
1	1,10239421	13,26631024	1,991461971	12,09777789
2	2,40473908	15,96943511	1,948472286	16,88561147
3	1,44588049	20,53008379	1,70481248	14,54528547
4	0,93105128	1,086782704	1,882145658	13,41288308
5	2,07855948	17,31706475	1,819824952	13,68200134
6	1,68242299	14,85083319	1,748668433	14,90626676
7	1,88242431	14,99778097	1,88385566	10,6422102
8	1,96265338	16,56835155	2,078487877	11,91033068
9	2,51345282	18,56944557	NA	9,102468406
10	0,65603988	18,95294933	1,869848683	10,70203729
11	1,4008529	15,05630432	2,031121268	13,55883766
12	2,48301026	12,98882302	2,048117182	11,35384419
13	2,54342521	13,46460955	2,07245232	10,69112942
14	0,88601789	14,51075299	2,173411293	8,267475062
15	1,43394837	11,2683922	1,896976609	7,10623676
16	0,8441121	14,78973393	1,951026563	16,74139734
17	1,43194527	1,062752496	2,457794613	12,08374678
18	1,8700147	18,00927519	1,927509183	13,09303135
19	1,32235622	15,69088303	2,199289288	10,58460497
20	0,87068289	16,39807801	2,142646017	11,79498969

Supplementary Data S2. Figure 3 a

Sample	Repeat 1	Repeat 2	Repeat 3	Repeat 4	Repeat 5	Repeat 6	Repeat 7	Repeat 8	Repeat 9	Average (AU)	STDEV.S
**WT**	548	586	627	570	640	898	102	302	434	567,285714	236,4478197
**ura3-52**	200644	196401	191088	203976	193889	195792	198015	197800	189630	197115	4268,666693
**YHR142W-gRNA1**	107592	92144	83231	137236	142666	148688	75877	84911	100763	112490,571	30187,75045
**YHR142W-gRNA2**	107933	111105	112504	65353	73859	62611	168216	158211	160138	100225,857	37142,45755
**YBR128C-gRNA1**	87265	97001	95415	204585	190543	187811	183661	156062	168187	149468,714	53085,01407
**YDR448W-gRNA1**	185101	193070	197586	156284	154993	157191	196389	193158	191896	177230,571	20120,77228
**YDR448W-gRNA2**	169382	176324	172886	187797	188909	184511	173569	178938	184059	179054	7878,978952
**YGR240C-gRNA1**	200534	183857	200600	237324	204779	260863	159590	158320	160316	206792,429	33395,2958
**YGR240C-gRNA2**	176545	200304	170919	182252	197491	194835	344894	276240	295402	209605,714	60679,87148
**YGR240C-no gRNA**	133699	125240	123957	131278	125370	124640	132341	124542	123427	128075	4167,308084

One-way ANOVA followed by Dunnett's T3 multiple-comparison test

Comparison	Adjusted p value	Significance
**ura3-52 vs WT**	<0.0001	****

**ura3-52 vs YHR142W-gRNA1**	<0.0001	****
**ura3-52 vs YHR142W-gRNA2**	0.0011	**
**ura3-52 vs YBR128C-gRNA1**	0.0003	***
**ura3-52 vs YDR448W-gRNA1**	<0.0001	****
**ura3-52 vs YDR448W-gRNA2**	<0.0001	****
**ura3-52 vs YGR240C-gRNA1**	<0.0001	****
**ura3-52 vs YGR240C-gRNA2**	0.0001	***

Supplementary Data S2.
Figure 3
b

Number of events

Sample	GFP-				GFP+			
	**Repeat 1**	**Repeat 2**	**Repeat 3**	**Average**	**Repeat 1**	**Repeat 2**	**Repeat 3**	**Average**
**WT**	4160	4093	4118	4123,667	0	0	0	0
**ura3-52**	6	6	5	5,666667	4301	4500	4126	4309
**YHR142W-gRNA1**	306	272	239	272,3333	1094	1081	1292	1155,666667
**YHR142W-gRNA2**	212	193	177	194	1286	1214	1313	1271
**YBR128C-gRNA1**	9	7	9	8,333333	4054	4363	4335	4250,666667
**YDR448W-gRNA1**	30	39	44	37,66667	3158	2652	2674	2828
**YDR448W-gRNA2**	42	38	40	40	2911	2890	2838	2879,666667
**YGR240C-gRNA1**	3389	3394	3658	3480,333	311	290	310	303,6666667
**YGR240C-gRNA2**	3918	3964	3703	3861,667	351	327	318	332
**YGR240C-no gRNA**	83	92	115	96,66667	4690	4758	4973	4807

**Relative to 100%**	**GFP-**				**GFP+**			
**Sample**	**Repeat 1**	**Repeat 2**	**Repeat 3**	**Average**	**Repeat 1**	**Repeat 2**	**Repeat 3**	**Average**
**WT**	100	100	100	100	0	0	0	0
**ura3-52**	0,139308103	0,133155792	0,12103607	0,131167	99,86069	99,866844	99,87896393	99,86883335
**YHR142W-gRNA1**	0,221511199	0,160183066	0,20718232	0,196292	99,77849	99,839817	99,79281768	99,8037078
**YHR142W-gRNA2**	0,941028858	1,449275362	1,61883738	1,336381	99,05897	98,550725	98,38116262	98,66361947
**YBR128C-gRNA1**	1,422282425	1,297814208	1,38985407	1,369984	98,57772	98,702186	98,61014593	98,63001643
**YDR448W-gRNA1**	21,85714286	20,10347376	15,610712	19,19044	78,14286	79,896526	84,38928805	80,80955714
**YDR448W-gRNA2**	14,15220294	13,71712864	11,8791946	13,24951	85,8478	86,282871	88,12080537	86,75049126
**YGR240C-gRNA1**	91,59459459	92,12812161	92,1875	91,97007	8,405405	7,8718784	7,8125	8,029927933
**YGR240C-gRNA2**	91,77793394	92,37939874	92,0915195	92,08295	8,222066	7,6206013	7,908480477	7,917049265
**YGR240C-no gRNA**	1,738948251	1,896907216	2,26022013	1,965359	98,26105	98,103093	97,73977987	98,03464147

GFP+/GFP-

**Sample**	**Repeat 1**	**Repeat 2**	**Repeat 3**	**Average (AU)**	**STDEV.S**	**T.TEST (gRNA1/gRNA2)**
**WT**	0	0	0	0	0	NA
**ura3-52**	716,8333333	750	825,2	764,0111	55,52537	NA
**YHR142W-gRNA1**	450,4444444	623,2857143	481,666667	518,4656	92,10943	
**YHR142W-gRNA2**	105,2666667	68	60,7727273	78,01313	23,87729	0,0104149
**YBR128C-gRNA1**	69,30952381	76,05263158	70,95	72,10405	3,516568	
**YDR448W-gRNA1**	3,575163399	3,974264706	5,40585774	4,318429	0,962651	NA
**YDR448W-gRNA2**	6,066037736	6,29015544	7,4180791	6,591424	0,724621	0,0343773
**YGR240C-gRNA1**	0,091767483	0,085444903	0,08474576	0,087319	0,003868	
**YGR240C-gRNA2**	0,089586524	0,082492432	0,08587632	0,085985	0,003548	0,6826349
**YGR240C-no gRNA**	56,5060241	51,7173913	43,2434783	50,48896	6,716067	NA

**Comparison**	**Adjusted p value**	**Significance**

**YHR142W-gRNA1 vs YHR142W-gRNA2**	>0.9999	ns
**YDR448W-gRNA1 vs YDR448W-gRNA2**	>0.9999	ns
**YGR240C-gRNA1 vs YGR240C-gRNA2**	0.9340	ns

Supplementary Data S3
Figure 4
c

mg.L-1	Replicate 1	Replicate 2	Replicate 3	Average (mgL^{-1})	STDEV.S	T.TEST
**WT**	0	0	2,11	0,703333333	1,21820907	
**yPCEN-NG**	78,9	80,9	92	83,93333333	7,057147677	0,001862127
**yFASTOP-NG**	442,83	506,19	568,1	505,7066667	62,6363986	0,00507544

Supplementary Data S4
Supplementary Figure S1

Samples	YBR128C	YDR448W
**WT**	1	1
**1**	2,20764945	1,76883141
**2**	2,16868731	2,22060879
**3**	2,55089007	2,3290755
**4**	2,35028354	2,10832492
**5**	2,16381278	2,25821972
**6**	1,95881959	1,51603576
**7**	2,57351355	2,01931471
**8**	2,1778126	2,2357538
**9**	2,67703157	2,04320682
**10**	2,37725441	2,37097964
**11**	2,59665469	2,21530346
**12**	2,45801534	1,52586191
**13**	2,32182593	0,92695765
**14**	2,37078209	1,94304962
**15**	2,405604	2,18538393
**16**	2,29639352	1,83892452
**17**	2,5386786	1,91526861
**18**	2,35407789	2,1457166
**19**	2,30626084	2,1113389
**20**	2,48215113	2,11588472

Supplementary Data S5.
Supplementary Figure S3.
a

Sample	Repeat 1	Repeat 2	Repeat 3	Repeat 4	Repeat 5	Repeat 6	Repeat 7	Repeat 8	Repeat 9	Average (AU)	STDEV.S	T.TEST (gRNA1/gRNA2)
**YHR142W-gRNA1**	107592	92144	83231	137236	142666	148688	75877	84911	100763	112490,5714	30187,75045	
**YHR142W-gRNA2**	107933	111105	112504	65353	73859	62611	168216	158211	160138	100225,8571	37142,45755	0,511183477

**YDR448W-gRNA1**	185101	193070	197586	156284	154993	157191	196389	193158	191896	177230,5714	20120,77228	NA
**YDR448W-gRNA2**	169382	176324	172886	187797	188909	184511	173569	178938	184059	179054	7878,978952	0,829068967
**YGR240C-gRNA1**	200534	183857	200600	237324	204779	260863	159590	158320	160316	206792,4286	33395,2958	
**YGR240C-gRNA2**	176545	200304	170919	182252	197491	194835	344894	276240	295402	209605,7143	60679,87148	0,916696277

b

**Sample**	**Repeat 1**	**Repeat 2**	**Repeat 3**	**Repeat 4**	**Repeat 5**	**Repeat 6**	**Repeat 7**	**Repeat 8**	**Repeat 9**	**Average (AU)**	**STDEV.S**	**T.TEST (gRNA/no gRNA)**
**YGR240C-gRNA1**	200534	183857	200600	237324	204779	260863	159590	158320	160316	206792,4286	33395,2958	
**YGR240C-gRNA2**	176545	200304	170919	182252	197491	194835	344894	276240	295402	209605,7143	60679,87148	0,000729073
**YGR240C-no gRNA**	133699	125240	123957	131278	125370	124640	132341	124542	123427	128075	4167,308084	0,01193441

c

Number of events

	**GFP-**			**GFP+**		
**Sample**	**Repeat 1**	**Repeat 2**	**Repeat 3**	**Repeat 1**	**Repeat 2**	**Repeat 3**
**YGR240C-gRNA1**	3389	3394	3658	311	290	310
**YGR240C-gRNA2**	3918	3964	3703	351	327	318
**YGR240C-no gRNA**	83	92	115	4690	4758	4973

Relative to 100%

**GFP-**				**GFP+**			
**Repeat 1**	**Repeat 2**	**Repeat 3**	**Average**	**Repeat 1**	**Repeat 2**	**Repeat 3**	**Average**
91,59459459	92,12812161	92,1875	91,97007207	8,405405405	7,871878393	7,8125	8,029927933
91,77793394	92,37939874	92,09151952	92,08295074	8,222066058	7,620601258	7,908480477	7,917049265
1,738948251	1,896907216	2,260220126	1,965358531	98,26105175	98,10309278	97,73977987	98,03464147

GFP+/GFP-

**Repeat 1**	**Repeat 2**	**Repeat 3**	**Average (AU)**	**STDEV.S**	**T.TEST (gRNA/no gRNA)**
0,091767483	0,085444903	0,084745763	0,08731938	0,00387	
0,089586524	0,082492432	0,085876317	0,08598509	0,00355	0,00586656
56,5060241	51,7173913	43,24347826	50,4889646	6,71607	0,005866254

Supplementary Data S6
Supplementary Figure S5

OD_{600}

**Tim**	**WT**	**ura3-52**	**YGR240C**	**YHR142W**	**YBR128C**	**YDR448W**

e (h)																	
**0**	0,2474	0,2308	0,2508	0,2205	0,2172	0,2272	0,1951	0,1884	0,3128	0,2271	0,1977	0,2139	0,2213	0,2069	0,2051	0,2157	0,1921
**4**	0,5033	0,6236	0,4448	0,4136	0,4002	0,2362	0,2075	0,1993	0,3042	0,2254	0,2004	0,2506	0,2647	0,2557	0,2012	0,2155	0,1945
**8**	0,6804	0,8036	0,5567	0,5202	0,5118	0,3533	0,3166	0,3092	0,3007	0,2219	0,1979	0,4307	0,4544	0,4618	0,2005	0,2165	0,1956
**12**	0,7031	0,899	0,6653	0,6208	0,6143	0,5215	0,4892	0,4818	0,2994	0,2203	0,1972	0,6004	0,6284	0,6435	0,2073	0,2236	0,2022
**16**	0,6885	0,9213	0,7274	0,6843	0,6806	0,6344	0,5901	0,5852	0,313	0,2302	0,2103	0,6788	0,7029	0,7221	0,2375	0,2519	0,2288
**20**	0,6851	0,9088	0,6302	0,5733	0,592	0,7118	0,6697	0,6603	0,3736	0,2911	0,2675	0,6524	0,6677	0,6703	0,3516	0,3588	0,3279
**24**	0,7114	0,9071	0,628	0,56	0,5894	0,6383	0,5881	0,6092	0,557	0,4755	0,4762	0,6604	0,6653	0,6827	0,5116	0,5179	0,4925
**30**	0,7721	0,8803	0,6276	0,5568	0,6154	0,628	0,5597	0,5909	0,7159	0,6431	0,6573	0,6816	0,6878	0,7183	0,6357	0,637	0,618
**32**	0,7888	0,871	0,6305	0,5583	0,5975	0,6315	0,5586	0,5887	0,7431	0,6724	0,6838	0,6741	0,6862	0,7073	0,651	0,6509	0,6334

Supplementary Data S7
Supplementary Figure S5

Time (h)	WT					yFASTOP-NG				
**OD_{600}**	**Replicate 1**	**Replicate 2**	**Replicate 3**	**Average**	**STDV**	**Replicate 1**	**Replicate 2**	**Replicate 3**	**Average**	**STDV**
**0**	0,325	0,327	0,32	0,324	0,00360555	0,308600008	0,32589999	0,3811	0,33853333	0,037865066
**4**	0,825	0,827	0,802	0,818	0,01389244	0,67839998	0,7493	0,7568	0,72816666	0,043262042
**8**	1,367	1,348	1,313	1,34266667	0,02739221	0,832099974	0,97009999	0,93970001	0,91396666	0,072509688
**12**	1,461	1,422	1,387	1,42333333	0,03701801	1,067499995	1,04620004	1,19340003	1,10236669	0,079553276
**16**	1,485	1,442	1,407	1,44466667	0,03906832	1,269299984	1,296	1,24430001	1,26986667	0,025854656
**20**	1,504	1,458	1,423	1,46166667	0,04062429	1,345600009	1,41320002	1,53670001	1,43183335	0,096903061
**24**	1,516	1,474	1,439	1,47633333	0,03855299	1,539700031	1,7277	1,91489995	1,72743332	0,187600099
**28**	NA	1,7354	1,71599996	1,72569998	0,0137179	1,535099983	1,69840002	1,91670001	1,71673334	0,191459466
**32**	NA	1,7657	1,74450004	1,75510002	0,01499063	1,58130002	1,67760003	1,83410001	1,69766668	0,127589034

NA = Not applicable
AU = Arbitrary Unit
OD600 = Optical intensity ant 600 nm

Supplementary Table

Table S1. Sequence of plasmids generated in the present study.	
**Plasmid name**	**Plasmid sequence (5' - 3')**
pHV001	TCGCGCGAACCCAACTGCACAGAACAAAAACCTGCAGGAAACGAAGATAAATCATG<br>TCGAAAGCTACATATAAGGAACGTGCTGCTACTCATCCTAGTCCTGTTGCTGCCAA<br>GCTATTTAATATCATGCACGAAAAGCAAACAAACTTGTGTGCTTCATTGGATGTTCG<br>TACCACCAAGGAATTACTGGAGTTAGTTGAAGCATTAGGTCCCAAAATTTGTTTACT<br>AAAAACACATGTGGATATCTTGACTGATTTTTCCATGGAGGGCACAGTTAAGCCGC<br>TAAAGGCATTATCCGCCAAGTACAATTTTTTACTCTTCGAAGACAGAAAATTTGCTG<br>ACATTGGTAATACAGTCAAATTGCAGTACTCTGCGGGTGTATACAGAATAGCAGAA<br>TGGGCAGACATTACGAATGCACACGGTGTGGTGGGCCCAGGTATTGTTAGCGGTT<br>TGAAGCAGGCGGCAGAAGAAGTAACAAAGGAACCTAGAGGCCTTTTGATGTTAGC<br>AGAATTGTCATGCAAGGGCTCCCTATCTACTGGAGAATATACTAAGGGTACTGTTG<br>ACATTGCGAAGAGCGACAAAGATTTTGTTATCGGCTTTATTGCTCAAAGAGACATG<br>GGTGGAAGAGATGAAGGTTACGATTGGTTGATTATGACACCCGGTGTGGGTTTAGA<br>TGACAAGGGAGACGCATTGGGTCAACAGTATAGAACCGTGGATGATGTCGTTTCTA<br>CAGGATCTGACATTATTATTGTTGGAAGAGGACTATTTGCAAAGGGAAGGGATGCT<br>AAGGTAGAGGGTGAACGTTACAGAAAAGCAGGCTGGGAAGCATATTTGAGAAGAT<br>GCGGCCAGCAAAACTAAAAAACTGTATTATAAGTAAATGCATGTATACTAAACTCAC<br>AAATTAGAGCTTCAATTTAATTATATCAGTTATTACCCTGTGCTCGAGTCTTTGAAAA<br>GATAATGTATGATTATGCTTTCACTCATATTTATACAGAAACTTGATGTTTTCTTTCG<br>AGTATATACAAGGTGATTACATGTACGTTTGAAGTACAACTCTAGATTTTGTAGTGC<br>CCTCTTGGGCTAGCGGTAAAGGTGCGCATTTTTTCACACCCTACAATGTTCTGTTC<br>AAAAGATTTTGGTCAAACGCTGTAGAAGTGAAAGTTGGTGCGCATGTTTCGGCGTT<br>CGAAACTTCTCCGCAGTGAAAGATAAATGATCGGGTTTTAgAGCTATGCTGTTTTGA<br>ATGGTCCCAAACCAATGCTAGTAGAGAAGGGAGAGCTATGCTGTTTTGAATGGTCC<br>CAAAACTTTTTTTGTTTTTTATGTCTGCATAGCTTCAAAATGTTTCTACTCCTTTTTTA<br>CTCTTCCAGATTTTCTCGGACTCCGCGCATCGCCGTACCACTTCAAAACACCCAAG<br>CACAGCATACTAAATTTCCCCTCTTTCTTCCTCTAGGGTGTCGTTAATTACCCGTAC<br>TAAAGGTTTGGAAAAGAAAAAAGAGGCCGCCTCGTTTCTTTTTCTTCGTCGAAAAAG<br>GCAATAAAAATTTTTATCACGTTTCTTTTTCTTGAAAATTTTTTTTTTTGATTTTTTTCT<br>CTTTCGATGACCTCCCATTGATATTTAAGTTAATAAACGGTCTTCAATTTCTCAAGTT<br>TCAGTTTCATTTTTCTTGTTCTATTACAACTTTTTTTACTTCTTGCTCATTAGAAAGAA<br>AGCATAGCAATCTAATCTAAGTTTTCTAGATGGATTATAAAGATGACGATGACAAAC<br>CTCCAAAAAAGAAGAGAAAGGTCGATAAGAAATACTCAATAGGCTTAGATATCGGC<br>ACAAATAGCGTCGGATGGGCGGTGATCACTGATGAATATAAGGTTCCGTCTAAAAA<br>GTTCAAGGTTCTGGGAAATACAGACCGCCACAGTATCAAAAAAAATCTTATAGGGG<br>CTCTTTTATTTGACAGTGGAGAGACAGCGGAAGCGACTCGTCTCAAACGGACAGCT<br>CGTAGAAGGTATACACGTCGGAAGAATCGTATTTGTTATCTACAGGAGATTTTTTCA<br>AATGAGATGGCGAAAGTAGATGATAGTTTCTTTCATCGACTTGAAGAGTCTTTTTTG<br>GTGGAAGAAGACAAGAAGCATGAACGTCATCCTATTTTTGGAAATATAGTAGATGA<br>AGTTGCTTATCATGAGAAATATCCAACTATCTATCATCTGCGAAAAAAATTGGTAGA<br>TTCTACTTATAAAGCGGATTTGCGCTTAATCTATTTGGCCTTAGCGCATATGATTAA<br>GTTTCGTGGTCATTTTTTGATTGAGGGAGATTTAAATCCTGATAATAGTGATGTGGA<br>CAAACTATTTATCCAGTTGGTACAAACCTACAATCAATTATTTGAAGAAAACCCTATT<br>AACGCAAGTGGAGTAGATGCTAAAGCGATTCTTTCTGCACGATTGAGTAAATCAAG<br>ACGATTAGAAAATCTCATTGCTCAGCTCCCCGGTGAGAAGAAAAATGGCTTATTTG<br>GGAATCTCATTGCTTTGTCATTGGGTTTGACCCCTAATTTTAAATCAAATTTTGATTT<br>GGCAGAAGATGCTAAATTACAGCTTTCAAAAGATACTTACGATGATGATTTAGATAA<br>TTTATTGGCGCAAATTGGAGATCAATATGCTGATTTGTTTTTGGCAGCTAAGAATTT<br>ATCAGATGCTATTTTACTTTCAGATATCCTAAGAGTAAATACTGAAATAACTAAGGCT<br>CCCCTATCAGCTTCAATGATTAAACGCTACGATGAACATCATCAAGACTTGACTCTT<br>TTAAAAGCTTTAGTTCGACAACAACTTCCAGAAAAGTATAAAGAAATCTTTTTTGATC<br>AATCAAAAAACGGATATGCAGGTTATATTGATGGGGGAGCTAGCCAAGAAGAATTT<br>TATAAATTTATCAAACCAATTTTAGAAAAAATGGATGGTACTGAGGAATTATTGGTGA<br>AACTAAATCGTGAAGATTTGCTGCGCAAGCAACGGACCTTTGACAACGGCTCTATT<br>ACCCATCAAATTCACTTGGGTGAGCTGCATGCTATTTTGAGAAGACAAGAAGACTTT<br>TATCCATTTTTAAAAGACAATCGTGAGAAGATTGAAAAAATCTTGACTTTTCGAATTC<br>CTTATTATGTTGGTCCATTGGCGCGTGGCAATAGTCGTTTTGCATGGATGACTCGG

	AAGTCTGAAGAAACAATTACCCCATGGAATTTTGAAGAAGTTGTCGATAAAGGTGCT<br>TCAGCTCAATCATTTATTGAACGCATGACAAACTTTGATAAAAATCTTCCAAATGAAA<br>AAGTACTACCAAAACATAGTTTGCTTTATGAGTATTTTACGGTTTATAACGAATTGAC<br>AAAGGTCAAATATGTTACTGAAGGAATGCGAAAACCAGCATTTCTTTCAGGTGAACA<br>GAAGAAAGCCATTGTTGATTTACTCTTCAAAACAAATCGAAAAGTAACCGTTAAGCA<br>ATTAAAAGAAGATTATTTCAAAAAAATAGAATGTTTTGATAGTGTTGAAATTTCAGGA<br>GTTGAAGATAGATTTAATGCTTCATTAGGTACCTACCATGATTTGCTAAAAATTATTA<br>AAGATAAAGATTTTTTGGATAATGAAGAAAATGAAGATATCTTAGAGGATATTGTTTT<br>AACATTGACCTTATTTGAAGATAGGGAGATGATTGAGGAAAGACTTAAAACATATGC<br>TCACCTCTTTGATGATAAGGTGATGAAACAGCTTAAACGTCGCCGTTATACTGGTTG<br>GGGACGTTTGTCTCGAAAATTGATTAATGGTATTAGGGATAAGCAATCTGGCAAAA<br>CAATATTAGATTTTTTGAAATCAGATGGTTTTGCCAATCGCAATTTTATGCAGCTGAT<br>CCATGATGATAGTTTGACATTTAAAGAAGACATTCAAAAAGCACAAGTGTCTGGACA<br>AGGCGATAGTTTACATGAACATATTGCAAATTTAGCTGGTAGCCCTGCTATTAAAAA<br>AGGTATTTTACAGACTGTAAAAGTTGTTGATGAATTGGTCAAAGTAATGGGGCGGC<br>ATAAGCCAGAAAATATCGTTATTGAAATGGCACGTGAAAATCAGACAACTCAAAAG<br>GGCCAGAAAAATTCGCGAGAGCGTATGAAACGAATCGAAGAAGGTATCAAAGAATT<br>AGGAAGTCAGATTCTTAAAGAGCATCCTGTTGAAAATACTCAATTGCAAAATGAAAA<br>GCTCTATCTCTATTATCTCCAAAATGGAAGAGACATGTATGTGGACCAAGAATTAGA<br>TATTAATCGTTTAAGTGATTATGATGTCGATCACATTGTTCCACAAAGTTTCCTTAAA<br>GACGATTCAATAGACAATAAGGTCTTAACGCGTTCTGATAAAAATCGTGGTAAATCG<br>GATAACGTTCCAAGTGAAGAAGTAGTCAAAAAGATGAAAAACTATTGGAGACAACTT<br>CTAAACGCCAAGTTAATCACTCAACGTAAGTTTGATAATTTAACGAAAGCTGAACGT<br>GGAGGTTTGAGTGAACTTGATAAAGCTGGTTTTATCAAACGCCAATTGGTTGAAACT<br>CGCCAAATCACTAAGCATGTGGCACAAATTTTGGATAGTCGCATGAATACTAAATAC<br>GATGAAAATGATAAACTTATTCGAGAGGTTAAAGTGATTACCTTAAAATCTAAATTAG<br>TTTCTGACTTCCGAAAAGATTTCCAATTCTATAAAGTACGTGAGATTAACAATTACCA<br>TCATGCCCATGATGCGTATCTAAATGCCGTCGTTGGAACTGCTTTGATTAAGAAATA<br>TCCAAAACTTGAATCGGAGTTTGTCTATGGTGATTATAAAGTTTATGATGTTCGTAA<br>AATGATTGCTAAGTCTGAGCAAGAAATAGGCAAAGCAACCGCAAAATATTTCTTTTA<br>CTCTAATATCATGAACTTCTTCAAAACAGAAATTACACTTGCAAATGGAGAGATTCG<br>CAAACGCCCTCTAATCGAAACTAATGGGGAAACTGGAGAAATTGTCTGGGATAAAG<br>GGCGAGATTTTGCCACAGTGCGCAAAGTATTGTCCATGCCCCAAGTCAATATTGTC<br>AAGAAAACAGAAGTACAGACAGGCGGATTCTCCAAGGAGTCAATTTTACCAAAAAG<br>AAATTCGGACAAGCTTATTGCTCGTAAAAAAGACTGGGATCCAAAAAAATATGGTG<br>GTTTTGATAGTCCAACGGTAGCTTATTCAGTCCTAGTGGTTGCTAAGGTGGAAAAA<br>GGGAAATCGAAGAAGTTAAAATCCGTTAAAGAGTTACTAGGGATCACAATTATGGA<br>AAGAAGTTCCTTTGAAAAAAATCCGATTGACTTTTTAGAAGCTAAAGGATATAAGGA<br>AGTTAAAAAAGACTTAATCATTAAACTACCTAAATATAGTCTTTTTGAGTTAGAAAAC<br>GGTCGTAAACGGATGCTGGCTAGTGCCGGAGAATTACAAAAAGGAAATGAGCTGG<br>CTCTGCCAAGCAAATATGTGAATTTTTTATATTTAGCTAGTCATTATGAAAAGTTGAA<br>GGGTAGTCCAGAAGATAACGAACAAAAACAATTGTTTGTGGAGCAGCATAAGCATT<br>ATTTAGATGAGATTATTGAGCAAATCAGTGAATTTTCTAAGCGTGTTATTTTAGCAGA<br>TGCCAATTTAGATAAAGTTCTTAGTGCATATAACAAACATAGAGACAAACCAATACG<br>TGAACAAGCAGAAAATATTATTCATTTATTTACGTTGACGAATCTTGGAGCTCCCGC<br>TGCTTTTAAATATTTTGATACAACAATTGATCGTAAACGATATACGTCTACAAAAGAA<br>GTTTTAGATGCCACTCTTATCCATCAATCCATCACTGGTCTTTATGAAACACGCATT<br>GATTTGAGTCAGCTAGGAGGTGACCCTCCAAAAAAGAAGAGAAAGGTCTGAGCGG<br>ATCTCTTATGTCTTTACGATTTATAGTTTTCATTATCAAGTATGCCTATATTAGTATAT<br>AGCATCTTTAGATGACAGTGTTCGAAGTTTCACGAATAAAAGATAATATTCTACTTTT<br>TGCTCCCACCGCGTTTGCTAGCACGAGTGAACACCATCCCTCGCCTGTGAGTTGTA<br>CCCATTCCTCTAAACTGTAGACATGGTAGCTTCAGCAGTGTTCGTTATGTACGGCA<br>TCCTCCAACAAACAGTCGGTTATAGTTTGTCCTGCTCCTCTGAATCGAGTCCCTCG<br>ATATTTCTCATACTAGTTCTAGAGATCTGCCAATTGAACATAACATGGTAGTTACATA<br>TACTAGTAATATGGTTCGGCACACATTAAAAGTATAAAAACTATCTGAATTACGAATT<br>ACATATATTGGTCATAAAAATCAATCAATCATCGTGTGTTTTATATGTCTCTTATCTA<br>AGTATAAGAATATCCATAGTTAATATTCACTTACGCTACCTTTTAACCTGTAATCATT<br>GTCAACAGGATATGTTAACGACCCACATTGATAAACGCTAGTATTTCTTTTTCCTCT<br>TCTTATTGGCCGGCTGTCTCTATACTCCCCTATAGTCTGTTTCTTTTCGTTTCGATT<br>GTTTTACGTTTGAGGCCTCGTGGCGCACATGGTACGCTGTGGTGCTCGCGGCTGG<br>GAACGAAACTCTGGGAGCTGCGATTGGCAGGAACCATTCAAAACAGCATAGCAAG

	TTAAAATAAGGCTAGTCCGTTATCAACTTGAAAAAGTGGCACCGAGTCGGTGCTTTT<br>TTTCCATATCCAACTTCCAATTTAATCTTTCTTTTTTAATTTTCACTTATTTGCGATAC<br>AGAAAGACCCTGCAGGCATGCAAGCTTGGCGTAATCATGGTCATAGCTGTTTCCTG<br>TGTGAAATTGTTATCCGCTCACAATTCCACACAACATACGAGCCGGAAGCATAAAG<br>TGTAAAGCCTGGGGTGCCTAATGAGTGAGCTAACTCACATTAATTGCGTTGCGCTC<br>ACTGCCCGCTTTCCAGTCGGGAAACCTGTCGTGCCAGCTGCATTAATGAATCGGC<br>CAACGCGCGGGGAGAGGCGGTTTGCGTATTGGGCGCTCTTCCGCTTCCTCGCTCA<br>CTGACTCGCTGCGCTCGGTCGTTCGGCTGCGGCGAGCGGTATCAGCTCACTCAAA<br>GGCGGTAATACGGTTATCCACAGAATCAGGGGATAACGCAGGAAAGAACATGTGA<br>GCAAAAGGCCAGCAAAAGGCCAGGAACCGTAAAAAGGCCGCGTTGCTGGCGTTTT<br>TCCATAGGCTCCGCCCCCCTGACGAGCATCACAAAAATCGACGCTCAAGTCAGAG<br>GTGGCGAAACCCGACAGGACTATAAAGATACCAGGCGTTTCCCCCTGGAAGCTCC<br>CTCGTGCGCTCTCCTGTTCCGACCCTGCCGCTTACCGGATACCTGTCCGCCTTTCT<br>CCCTTCGGGAAGCGTGGCGCTTTCTCATAGCTCACGCTGTAGGTATCTCAGTTCG<br>GTGTAGGTCGTTCGCTCCAAGCTGGGCTGTGTGCACGAACCCCCCGTTCAGCCCG<br>ACCGCTGCGCCTTATCCGGTAACTATCGTCTTGAGTCCAACCCGGTAAGACACGAC<br>TTATCGCCACTGGCAGCAGCCACTGGTAACAGGATTAGCAGAGCGAGGTATGTAG<br>GCGGTGCTACAGAGTTCTTGAAGTGGTGGCCTAACTACGGCTACACTAGAAGAAC<br>AGTATTTGGTATCTGCGCTCTGCTGAAGCCAGTTACCTTCGGAAAAAGAGTTGGTA<br>GCTCTTGATCCGGCAAACAAACCACCGCTGGTAGCGGTGGTTTTTTTGTTTGCAAG<br>CAGCAGATTACGCGCAGAAAAAAAGGATCTCAAGAAGATCCTTTGATCTTTTCTAC<br>GGGGTCTGACGCTCAGTGGAACGAAAACTCACGTTAAGGGATTTTGGTCATGAGAT<br>TATCAAAAAGGATCTTCACCTAGATCCTTTTAAATTAAAAATGAAGTTTTAAATCAAT<br>CTAAAGTATATATGAGTAAACTTGGTCTGACAGTTACCAATGCTTAATCAGTGAGGC<br>ACCTATCTCAGCGATCTGTCTATTTCGTTCATCCATAGTTGCCTGACTCCCCGTCGT<br>GTAGATAACTACGATACGGGAGGGCTTACCATCTGGCCCCAGTGCTGCAATGATA<br>CCGCGGCTCCCACGCTCACCGGCTCCAGATTTATCAGCAATAAACCAGCCAGCCG<br>GAAGGGCCGAGCGCAGAAGTGGTCCTGCAACTTTATCCGCCTCCATCCAGTCTAT<br>TAATTGTTGCCGGGAAGCTAGAGTAAGTAGTTCGCCAGTTAATAGTTTGCGCAACG<br>TTGTTGCCATTGCTACAGGCATCGTGGTGTCACGCTCGTCGTTTGGTATGGCTTCA<br>TTCAGCTCCGGTTCCCAACGATCAAGGCGAGTTACATGATCCCCCATGTTGTGCAA<br>AAAAGCGGTTAGCTCCTTCGGTCCTCCGATCGTTGTCAGAAGTAAGTTGGCCGCA<br>GTGTTATCACTCATGGTTATGGCAGCACTGCATAATTCTCTTACTGTCATGCCATCC<br>GTAAGATGCTTTTCTGTGACTGGTGAGTACTCAACCAAGTCATTCTGAGAATAGTGT<br>ATGCGGCGACCGAGTTGCTCTTGCCCGGCGTCAATACGGGATAATACCGCGCCAC<br>ATAGCAGAACTTTAAAAGTGCTCATCATTGGAAAACGTTCTTCGGGGCGAAAACTC<br>TCAAGGATCTTACCGCTGTTGAGATCCAGTTCGATGTAACCCACTCGTGCACCCAA<br>CTGATCTTCAGCATCTTTTACTTTCACCAGCGTTTCTGGGTGAGCAAAAACAGGAA<br>GGCAAAATGCCGCAAAAAAGGGAATAAGGGCGACACGGAAATGTTGAATACTCATA<br>CTCTTCCTTTTTCAATATTATTGAAGCATTTATCAGGGTTATTGTCTCATGAGCGGAT<br>ACATATTTGAATGTATTTAGAAAAATAAACAAATAGGGGTTCCGCGCACATTTCCCC<br>GAAAAGTGCCACCTGAACGAAGCATCTGTGCTTCATTTTGTAGAACAAAAATGCAA<br>CGCGAGAGCGCTAATTTTTCAAACAAAGAATCTGAGCTGCATTTTTACAGAACAGAA<br>ATGCAACGCGAAAGCGCTATTTTACCAACGAAGAATCTGTGCTTCATTTTTGTAAAA<br>CAAAAATGCAACGCGAGAGCGCTAATTTTTCAAACAAAGAATCTGAGCTGCATTTTT<br>ACAGAACAGAAATGCAACGCGAGAGCGCTATTTTACCAACAAAGAATCTATACTTCT<br>TTTTTGTTCTACAAAAATGCATCCCGAGAGCGCTATTTTTCTAACAAAGCATCTTAG<br>ATTACTTTTTTTCTCCTTTGTGCGCTCTATAATGCAGTCTCTTGATAACTTTTTGCAC<br>TGTAGGTCCGTTAAGGTTAGAAGAAGGCTACTTTGGTGTCTATTTTCTCTTCCATAA<br>AAAAAGCCTGACTCCACTTCCCGCGTTTACTGATTACTAGCGAAGCTGCGGGTGCA<br>TTTTTTCAAGATAAAGGCATCCCCGATTATATTCTATACCGATGTGGATTGCGCATA<br>CTTTGTGAACAGAAAGTGATAGCGTTGATGATTCTTCATTGGTCAGAAAATTATGAA<br>CGGTTTCTTCTATTTTGTCTCTATATACTACGTATAGGAAATGTTTACATTTTCGTAT<br>TGTTTTCGATTCACTCTATGAATAGTTCTTACTACAATTTTTTTGTCTAAAGAGTAATA<br>CTAGAGATAAACATAAAAAATGTAGAGGTCGAGTTTAGATGCAAGTTCAAGGAGCG<br>AAAGGTGGATGGGTAGGTTATATAGGGATATAGCACAGAGATATATAGCAAAGAGA<br>TACTTTTGAGCAATGTTTGTGGAAGCGGTATTCGCAATATTTTAGTAGCTCGTTACA<br>GTCCGGTGCGTTTTTGGTTTTTTGAAAGTGCGTCTTCAGAGCGCTTTTGGTTTTCAA<br>AAGCGCTCTGAAGTTCCTATACTTTCTAGAGAATAGGAACTTCGGAATAGGAACTTC<br>AAAGCGTTTCCGAAAACGAGCGCTTCCGAAAATGCAACGCGAGCTGCGCACATAC<br>AGCTCACTGTTCACGTCGCACCTATATCTGCGTGTTGCCTGTATATATATATACATG

	AGAAGAACGGCATAGTGCGTGTTTATGCTTAAATGCGTACTTATATGCGTCTATTTA<br>TGTAGGATGAAAGGTAGTCTAGTACCTCCTGTGATATTATCCCATTCCATGCGGGG<br>TATCGTATGCTTCCTTCAGCACTACCCTTTAGCTGTTCTATATGCTGCCACTCCTCA<br>ATTGGATTAGTCTCATCCTTCAATGCTATCATTTCCTTTGATATTGGATCATACTAAG<br>AAACCATTATTATCATGACATTAACCTATAAAAATAGGCGTATCACGAGGCCCTTTC<br>GTC
pHV002	TCGCGCGAACCCAACTGCACAGAACAAAAACCTGCAGGAAACGAAGATAAATCATG<br>TCGAAAGCTACATATAAGGAACGTGCTGCTACTCATCCTAGTCCTGTTGCTGCCAA<br>GCTATTTAATATCATGCACGAAAAGCAAACAAACTTGTGTGCTTCATTGGATGTTCG<br>TACCACCAAGGAATTACTGGAGTTAGTTGAAGCATTAGGTCCCAAAATTTGTTTACT<br>AAAAACACATGTGGATATCTTGACTGATTTTTCCATGGAGGGCACAGTTAAGCCGC<br>TAAAGGCATTATCCGCCAAGTACAATTTTTTACTCTTCGAAGACAGAAAATTTGCTG<br>ACATTGGTAATACAGTCAAATTGCAGTACTCTGCGGGTGTATACAGAATAGCAGAA<br>TGGGCAGACATTACGAATGCACACGGTGTGGTGGGCCCAGGTATTGTTAGCGGTT<br>TGAAGCAGGCGGCAGAAGAAGTAACAAAGGAACCTAGAGGCCTTTTGATGTTAGC<br>AGAATTGTCATGCAAGGGCTCCCTATCTACTGGAGAATATACTAAGGGTACTGTTG<br>ACATTGCGAAGAGCGACAAAGATTTTGTTATCGGCTTTATTGCTCAAAGAGACATG<br>GGTGGAAGAGATGAAGGTTACGATTGGTTGATTATGACACCCGGTGTGGGTTTAGA<br>TGACAAGGGAGACGCATTGGGTCAACAGTATAGAACCGTGGATGATGTCGTTTCTA<br>CAGGATCTGACATTATTATTGTTGGAAGAGGACTATTTGCAAAGGGAAGGGATGCT<br>AAGGTAGAGGGTGAACGTTACAGAAAAGCAGGCTGGGAAGCATATTTGAGAAGAT<br>GCGGCCAGCAAAACTAAAAAACTGTATTATAAGTAAATGCATGTATACTAAACTCAC<br>AAATTAGAGCTTCAATTTAATTATATCAGTTATTACCCTGTGCTCGAGTCTTTGAAAA<br>GATAATGTATGATTATGCTTTCACTCATATTTATACAGAAACTTGATGTTTTCTTTCG<br>AGTATATACAAGGTGATTACATGTACGTTTGAAGTACAACTCTAGATTTTGTAGTGC<br>CCTCTTGGGCTAGCGGTAAAGGTGCGCATTTTTTCACACCCTACAATGTTCTGTTC<br>AAAAGATTTTGGTCAAACGCTGTAGAAGTGAAAGTTGGTGCGCATGTTTCGGCGTT<br>CGAAACTTCTCCGCAGTGAAAGATAAATGATCGGGTTTTAgAGCTATGCTGTTTTGA<br>ATGGTCCCAAAGAGATAGGTAAATAAACGCGAGAGCTATGCTGTTTTGAATGGTCC<br>CAAAACTTTTTTTGTTTTTTATGTCTGCATAGCTTCAAAATGTTTCTACTCCTTTTTTA<br>CTCTTCCAGATTTTCTCGGACTCCGCGCATCGCCGTACCACTTCAAAACACCCAAG<br>CACAGCATACTAAATTTCCCCTCTTTCTTCCTCTAGGGTGTCGTTAATTACCCGTAC<br>TAAAGGTTTGGAAAAGAAAAAAGAGGCCGCCTCGTTTCTTTTTCTTCGTCGAAAAAG<br>GCAATAAAAATTTTTATCACGTTTCTTTTTCTTGAAAATTTTTTTTTTTGATTTTTTTCT<br>CTTTCGATGACCTCCCATTGATATTTAAGTTAATAAACGGTCTTCAATTTCTCAAGTT<br>TCAGTTTCATTTTTCTTGTTCTATTACAACTTTTTTTACTTCTTGCTCATTAGAAAGAA<br>AGCATAGCAATCTAATCTAAGTTTTCTAGATGGATTATAAAGATGACGATGACAAAC<br>CTCCAAAAAAGAAGAGAAAGGTCGATAAGAAATACTCAATAGGCTTAGATATCGGC<br>ACAAATAGCGTCGGATGGGCGGTGATCACTGATGAATATAAGGTTCCGTCTAAAAA<br>GTTCAAGGTTCTGGGAAATACAGACCGCCACAGTATCAAAAAAAATCTTATAGGGG<br>CTCTTTTATTTGACAGTGGAGAGACAGCGGAAGCGACTCGTCTCAAACGGACAGCT<br>CGTAGAAGGTATACACGTCGGAAGAATCGTATTTGTTATCTACAGGAGATTTTTTCA<br>AATGAGATGGCGAAAGTAGATGATAGTTTCTTTCATCGACTTGAAGAGTCTTTTTTG<br>GTGGAAGAAGACAAGAAGCATGAACGTCATCCTATTTTTGGAAATATAGTAGATGA<br>AGTTGCTTATCATGAGAAATATCCAACTATCTATCATCTGCGAAAAAAATTGGTAGA<br>TTCTACTTATAAAGCGGATTTGCGCTTAATCTATTTGGCCTTAGCGCATATGATTAA<br>GTTTCGTGGTCATTTTTTGATTGAGGGAGATTTAAATCCTGATAATAGTGATGTGGA<br>CAAACTATTTATCCAGTTGGTACAAACCTACAATCAATTATTTGAAGAAAACCCTATT<br>AACGCAAGTGGAGTAGATGCTAAAGCGATTCTTTCTGCACGATTGAGTAAATCAAG<br>ACGATTAGAAAATCTCATTGCTCAGCTCCCCGGTGAGAAGAAAAATGGCTTATTTG<br>GGAATCTCATTGCTTTGTCATTGGGTTTGACCCCTAATTTTAAATCAAATTTTGATTT<br>GGCAGAAGATGCTAAATTACAGCTTTCAAAAGATACTTACGATGATGATTTAGATAA<br>TTTATTGGCGCAAATTGGAGATCAATATGCTGATTTGTTTTTGGCAGCTAAGAATTT<br>ATCAGATGCTATTTTACTTTCAGATATCCTAAGAGTAAATACTGAAATAACTAAGGCT<br>CCCCTATCAGCTTCAATGATTAAACGCTACGATGAACATCATCAAGACTTGACTCTT<br>TTAAAAGCTTTAGTTCGACAACAACTTCCAGAAAAGTATAAAGAAATCTTTTTTGATC<br>AATCAAAAAACGGATATGCAGGTTATATTGATGGGGGAGCTAGCCAAGAAGAATTT<br>TATAAATTTATCAAACCAATTTTAGAAAAAATGGATGGTACTGAGGAATTATTGGTGA<br>AACTAAATCGTGAAGATTTGCTGCGCAAGCAACGGACCTTTGACAACGGCTCTATT<br>ACCCATCAAATTCACTTGGGTGAGCTGCATGCTATTTTGAGAAGACAAGAAGACTTT<br>TATCCATTTTTAAAAGACAATCGTGAGAAGATTGAAAAAATCTTGACTTTTCGAATTC

	CTTATTATGTTGGTCCATTGGCGCGTGGCAATAGTCGTTTTGCATGGATGACTCGG<br>AAGTCTGAAGAAACAATTACCCCATGGAATTTTGAAGAAGTTGTCGATAAAGGTGCT<br>TCAGCTCAATCATTTATTGAACGCATGACAAACTTTGATAAAAATCTTCCAAATGAAA<br>AAGTACTACCAAAACATAGTTTGCTTTATGAGTATTTTACGGTTTATAACGAATTGAC<br>AAAGGTCAAATATGTTACTGAAGGAATGCGAAAACCAGCATTTCTTTCAGGTGAACA<br>GAAGAAAGCCATTGTTGATTTACTCTTCAAAACAAATCGAAAAGTAACCGTTAAGCA<br>ATTAAAAGAAGATTATTTCAAAAAAATAGAATGTTTTGATAGTGTTGAAATTTCAGGA<br>GTTGAAGATAGATTTAATGCTTCATTAGGTACCTACCATGATTTGCTAAAAATTATTA<br>AAGATAAAGATTTTTTGGATAATGAAGAAAATGAAGATATCTTAGAGGATATTGTTTT<br>AACATTGACCTTATTTGAAGATAGGGAGATGATTGAGGAAAGACTTAAAACATATGC<br>TCACCTCTTTGATGATAAGGTGATGAAACAGCTTAAACGTCGCCGTTATACTGGTTG<br>GGGACGTTTGTCTCGAAAATTGATTAATGGTATTAGGGATAAGCAATCTGGCAAAA<br>CAATATTAGATTTTTTGAAATCAGATGGTTTTGCCAATCGCAATTTTATGCAGCTGAT<br>CCATGATGATAGTTTGACATTTAAAGAAGACATTCAAAAAGCACAAGTGTCTGGACA<br>AGGCGATAGTTTACATGAACATATTGCAAATTTAGCTGGTAGCCCTGCTATTAAAAA<br>AGGTATTTTACAGACTGTAAAAGTTGTTGATGAATTGGTCAAAGTAATGGGGCGGC<br>ATAAGCCAGAAAATATCGTTATTGAAATGGCACGTGAAAATCAGACAACTCAAAAG<br>GGCCAGAAAAATTCGCGAGAGCGTATGAAACGAATCGAAGAAGGTATCAAAGAATT<br>AGGAAGTCAGATTCTTAAAGAGCATCCTGTTGAAAATACTCAATTGCAAAATGAAAA<br>GCTCTATCTCTATTATCTCCAAAATGGAAGAGACATGTATGTGGACCAAGAATTAGA<br>TATTAATCGTTTAAGTGATTATGATGTCGATCACATTGTTCCACAAAGTTTCCTTAAA<br>GACGATTCAATAGACAATAAGGTCTTAACGCGTTCTGATAAAAATCGTGGTAAATCG<br>GATAACGTTCCAAGTGAAGAAGTAGTCAAAAAGATGAAAAACTATTGGAGACAACTT<br>CTAAACGCCAAGTTAATCACTCAACGTAAGTTTGATAATTTAACGAAAGCTGAACGT<br>GGAGGTTTGAGTGAACTTGATAAAGCTGGTTTTATCAAACGCCAATTGGTTGAAACT<br>CGCCAAATCACTAAGCATGTGGCACAAATTTTGGATAGTCGCATGAATACTAAATAC<br>GATGAAAATGATAAACTTATTCGAGAGGTTAAAGTGATTACCTTAAAATCTAAATTAG<br>TTTCTGACTTCCGAAAAGATTTCCAATTCTATAAAGTACGTGAGATTAACAATTACCA<br>TCATGCCCATGATGCGTATCTAAATGCCGTCGTTGGAACTGCTTTGATTAAGAAATA<br>TCCAAAACTTGAATCGGAGTTTGTCTATGGTGATTATAAAGTTTATGATGTTCGTAA<br>AATGATTGCTAAGTCTGAGCAAGAAATAGGCAAAGCAACCGCAAAATATTTCTTTTA<br>CTCTAATATCATGAACTTCTTCAAAACAGAAATTACACTTGCAAATGGAGAGATTCG<br>CAAACGCCCTCTAATCGAAACTAATGGGGAAACTGGAGAAATTGTCTGGGATAAAG<br>GGCGAGATTTTGCCACAGTGCGCAAAGTATTGTCCATGCCCCAAGTCAATATTGTC<br>AAGAAAACAGAAGTACAGACAGGCGGATTCTCCAAGGAGTCAATTTTACCAAAAAG<br>AAATTCGGACAAGCTTATTGCTCGTAAAAAAGACTGGGATCCAAAAAAATATGGTG<br>GTTTTGATAGTCCAACGGTAGCTTATTCAGTCCTAGTGGTTGCTAAGGTGGAAAAA<br>GGGAAATCGAAGAAGTTAAAATCCGTTAAAGAGTTACTAGGGATCACAATTATGGA<br>AAGAAGTTCCTTTGAAAAAAATCCGATTGACTTTTTAGAAGCTAAAGGATATAAGGA<br>AGTTAAAAAAGACTTAATCATTAAACTACCTAAATATAGTCTTTTTGAGTTAGAAAAC<br>GGTCGTAAACGGATGCTGGCTAGTGCCGGAGAATTACAAAAAGGAAATGAGCTGG<br>CTCTGCCAAGCAAATATGTGAATTTTTTATATTTAGCTAGTCATTATGAAAAGTTGAA<br>GGGTAGTCCAGAAGATAACGAACAAAAACAATTGTTTGTGGAGCAGCATAAGCATT<br>ATTTAGATGAGATTATTGAGCAAATCAGTGAATTTTCTAAGCGTGTTATTTTAGCAGA<br>TGCCAATTTAGATAAAGTTCTTAGTGCATATAACAAACATAGAGACAAACCAATACG<br>TGAACAAGCAGAAAATATTATTCATTTATTTACGTTGACGAATCTTGGAGCTCCCGC<br>TGCTTTTAAATATTTTGATACAACAATTGATCGTAAACGATATACGTCTACAAAAGAA<br>GTTTTAGATGCCACTCTTATCCATCAATCCATCACTGGTCTTTATGAAACACGCATT<br>GATTTGAGTCAGCTAGGAGGTGACCCTCCAAAAAAGAAGAGAAAGGTCTGAGCGG<br>ATCTCTTATGTCTTTACGATTTATAGTTTTCATTATCAAGTATGCCTATATTAGTATAT<br>AGCATCTTTAGATGACAGTGTTCGAAGTTTCACGAATAAAAGATAATATTCTACTTTT<br>TGCTCCCACCGCGTTTGCTAGCACGAGTGAACACCATCCCTCGCCTGTGAGTTGTA<br>CCCATTCCTCTAAACTGTAGACATGGTAGCTTCAGCAGTGTTCGTTATGTACGGCA<br>TCCTCCAACAAACAGTCGGTTATAGTTTGTCCTGCTCCTCTGAATCGAGTCCCTCG<br>ATATTTCTCATACTAGTTCTAGAGATCTGCCAATTGAACATAACATGGTAGTTACATA<br>TACTAGTAATATGGTTCGGCACACATTAAAAGTATAAAAACTATCTGAATTACGAATT<br>ACATATATTGGTCATAAAAATCAATCAATCATCGTGTGTTTTATATGTCTCTTATCTA<br>AGTATAAGAATATCCATAGTTAATATTCACTTACGCTACCTTTTAACCTGTAATCATT<br>GTCAACAGGATATGTTAACGACCCACATTGATAAACGCTAGTATTTCTTTTTCCTCT<br>TCTTATTGGCCGGCTGTCTCTATACTCCCCTATAGTCTGTTTCTTTTCGTTTCGATT<br>GTTTTACGTTTGAGGCCTCGTGGCGCACATGGTACGCTGTGGTGCTCGCGGCTGG

	GAACGAAACTCTGGGAGCTGCGATTGGCAGGAACCATTCAAAACAGCATAGCAAG<br>TTAAAATAAGGCTAGTCCGTTATCAACTTGAAAAAGTGGCACCGAGTCGGTGCTTTT<br>TTTCCATATCCAACTTCCAATTTAATCTTTCTTTTTTAATTTTCACTTATTTGCGATAC<br>AGAAAGACCCTGCAGGCATGCAAGCTTGGCGTAATCATGGTCATAGCTGTTTCCTG<br>TGTGAAATTGTTATCCGCTCACAATTCCACACAACATACGAGCCGGAAGCATAAAG<br>TGTAAAGCCTGGGGTGCCTAATGAGTGAGCTAACTCACATTAATTGCGTTGCGCTC<br>ACTGCCCGCTTTCCAGTCGGGAAACCTGTCGTGCCAGCTGCATTAATGAATCGGC<br>CAACGCGCGGGGAGAGGCGGTTTGCGTATTGGGCGCTCTTCCGCTTCCTCGCTCA<br>CTGACTCGCTGCGCTCGGTCGTTCGGCTGCGGCGAGCGGTATCAGCTCACTCAAA<br>GGCGGTAATACGGTTATCCACAGAATCAGGGGATAACGCAGGAAAGAACATGTGA<br>GCAAAAGGCCAGCAAAAGGCCAGGAACCGTAAAAAGGCCGCGTTGCTGGCGTTTT<br>TCCATAGGCTCCGCCCCCCTGACGAGCATCACAAAAATCGACGCTCAAGTCAGAG<br>GTGGCGAAACCCGACAGGACTATAAAGATACCAGGCGTTTCCCCCTGGAAGCTCC<br>CTCGTGCGCTCTCCTGTTCCGACCCTGCCGCTTACCGGATACCTGTCCGCCTTTCT<br>CCCTTCGGGAAGCGTGGCGCTTTCTCATAGCTCACGCTGTAGGTATCTCAGTTCG<br>GTGTAGGTCGTTCGCTCCAAGCTGGGCTGTGTGCACGAACCCCCCGTTCAGCCCG<br>ACCGCTGCGCCTTATCCGGTAACTATCGTCTTGAGTCCAACCCGGTAAGACACGAC<br>TTATCGCCACTGGCAGCAGCCACTGGTAACAGGATTAGCAGAGCGAGGTATGTAG<br>GCGGTGCTACAGAGTTCTTGAAGTGGTGGCCTAACTACGGCTACACTAGAAGAAC<br>AGTATTTGGTATCTGCGCTCTGCTGAAGCCAGTTACCTTCGGAAAAAGAGTTGGTA<br>GCTCTTGATCCGGCAAACAAACCACCGCTGGTAGCGGTGGTTTTTTTGTTTGCAAG<br>CAGCAGATTACGCGCAGAAAAAAAGGATCTCAAGAAGATCCTTTGATCTTTTCTAC<br>GGGGTCTGACGCTCAGTGGAACGAAAACTCACGTTAAGGGATTTTGGTCATGAGAT<br>TATCAAAAAGGATCTTCACCTAGATCCTTTTAAATTAAAAATGAAGTTTTAAATCAAT<br>CTAAAGTATATATGAGTAAACTTGGTCTGACAGTTACCAATGCTTAATCAGTGAGGC<br>ACCTATCTCAGCGATCTGTCTATTTCGTTCATCCATAGTTGCCTGACTCCCCGTCGT<br>GTAGATAACTACGATACGGGAGGGCTTACCATCTGGCCCCAGTGCTGCAATGATA<br>CCGCGGCTCCCACGCTCACCGGCTCCAGATTTATCAGCAATAAACCAGCCAGCCG<br>GAAGGGCCGAGCGCAGAAGTGGTCCTGCAACTTTATCCGCCTCCATCCAGTCTAT<br>TAATTGTTGCCGGGAAGCTAGAGTAAGTAGTTCGCCAGTTAATAGTTTGCGCAACG<br>TTGTTGCCATTGCTACAGGCATCGTGGTGTCACGCTCGTCGTTTGGTATGGCTTCA<br>TTCAGCTCCGGTTCCCAACGATCAAGGCGAGTTACATGATCCCCCATGTTGTGCAA<br>AAAAGCGGTTAGCTCCTTCGGTCCTCCGATCGTTGTCAGAAGTAAGTTGGCCGCA<br>GTGTTATCACTCATGGTTATGGCAGCACTGCATAATTCTCTTACTGTCATGCCATCC<br>GTAAGATGCTTTTCTGTGACTGGTGAGTACTCAACCAAGTCATTCTGAGAATAGTGT<br>ATGCGGCGACCGAGTTGCTCTTGCCCGGCGTCAATACGGGATAATACCGCGCCAC<br>ATAGCAGAACTTTAAAAGTGCTCATCATTGGAAAACGTTCTTCGGGGCGAAAACTC<br>TCAAGGATCTTACCGCTGTTGAGATCCAGTTCGATGTAACCCACTCGTGCACCCAA<br>CTGATCTTCAGCATCTTTTACTTTCACCAGCGTTTCTGGGTGAGCAAAAACAGGAA<br>GGCAAAATGCCGCAAAAAAGGGAATAAGGGCGACACGGAAATGTTGAATACTCATA<br>CTCTTCCTTTTTCAATATTATTGAAGCATTTATCAGGGTTATTGTCTCATGAGCGGAT<br>ACATATTTGAATGTATTTAGAAAAATAAACAAATAGGGGTTCCGCGCACATTTCCCC<br>GAAAAGTGCCACCTGAACGAAGCATCTGTGCTTCATTTTGTAGAACAAAAATGCAA<br>CGCGAGAGCGCTAATTTTTCAAACAAAGAATCTGAGCTGCATTTTTACAGAACAGAA<br>ATGCAACGCGAAAGCGCTATTTTACCAACGAAGAATCTGTGCTTCATTTTTGTAAAA<br>CAAAAATGCAACGCGAGAGCGCTAATTTTTCAAACAAAGAATCTGAGCTGCATTTTT<br>ACAGAACAGAAATGCAACGCGAGAGCGCTATTTTACCAACAAAGAATCTATACTTCT<br>TTTTTGTTCTACAAAAATGCATCCCGAGAGCGCTATTTTTCTAACAAAGCATCTTAG<br>ATTACTTTTTTTCTCCTTTGTGCGCTCTATAATGCAGTCTCTTGATAACTTTTTGCAC<br>TGTAGGTCCGTTAAGGTTAGAAGAAGGCTACTTTGGTGTCTATTTTCTCTTCCATAA<br>AAAAAGCCTGACTCCACTTCCCGCGTTTACTGATTACTAGCGAAGCTGCGGGTGCA<br>TTTTTTCAAGATAAAGGCATCCCCGATTATATTCTATACCGATGTGGATTGCGCATA<br>CTTTGTGAACAGAAAGTGATAGCGTTGATGATTCTTCATTGGTCAGAAAATTATGAA<br>CGGTTTCTTCTATTTTGTCTCTATATACTACGTATAGGAAATGTTTACATTTTCGTAT<br>TGTTTTCGATTCACTCTATGAATAGTTCTTACTACAATTTTTTTGTCTAAAGAGTAATA<br>CTAGAGATAAACATAAAAAATGTAGAGGTCGAGTTTAGATGCAAGTTCAAGGAGCG<br>AAAGGTGGATGGGTAGGTTATATAGGGATATAGCACAGAGATATATAGCAAAGAGA<br>TACTTTTGAGCAATGTTTGTGGAAGCGGTATTCGCAATATTTTAGTAGCTCGTTACA<br>GTCCGGTGCGTTTTTGGTTTTTTGAAAGTGCGTCTTCAGAGCGCTTTTGGTTTTCAA<br>AAGCGCTCTGAAGTTCCTATACTTTCTAGAGAATAGGAACTTCGGAATAGGAACTTC<br>AAAGCGTTTCCGAAAACGAGCGCTTCCGAAAATGCAACGCGAGCTGCGCACATAC

	AGCTCACTGTTCACGTCGCACCTATATCTGCGTGTTGCCTGTATATATATATACATG<br>AGAAGAACGGCATAGTGCGTGTTTATGCTTAAATGCGTACTTATATGCGTCTATTTA<br>TGTAGGATGAAAGGTAGTCTAGTACCTCCTGTGATATTATCCCATTCCATGCGGGG<br>TATCGTATGCTTCCTTCAGCACTACCCTTTAGCTGTTCTATATGCTGCCACTCCTCA<br>ATTGGATTAGTCTCATCCTTCAATGCTATCATTTCCTTTGATATTGGATCATACTAAG<br>AAACCATTATTATCATGACATTAACCTATAAAAATAGGCGTATCACGAGGCCCTTTC<br>GTC
pHV003	TCGCGCGAACCCAACTGCACAGAACAAAAACCTGCAGGAAACGAAGATAAATCATG<br>TCGAAAGCTACATATAAGGAACGTGCTGCTACTCATCCTAGTCCTGTTGCTGCCAA<br>GCTATTTAATATCATGCACGAAAAGCAAACAAACTTGTGTGCTTCATTGGATGTTCG<br>TACCACCAAGGAATTACTGGAGTTAGTTGAAGCATTAGGTCCCAAAATTTGTTTACT<br>AAAAACACATGTGGATATCTTGACTGATTTTTCCATGGAGGGCACAGTTAAGCCGC<br>TAAAGGCATTATCCGCCAAGTACAATTTTTTACTCTTCGAAGACAGAAAATTTGCTG<br>ACATTGGTAATACAGTCAAATTGCAGTACTCTGCGGGTGTATACAGAATAGCAGAA<br>TGGGCAGACATTACGAATGCACACGGTGTGGTGGGCCCAGGTATTGTTAGCGGTT<br>TGAAGCAGGCGGCAGAAGAAGTAACAAAGGAACCTAGAGGCCTTTTGATGTTAGC<br>AGAATTGTCATGCAAGGGCTCCCTATCTACTGGAGAATATACTAAGGGTACTGTTG<br>ACATTGCGAAGAGCGACAAAGATTTTGTTATCGGCTTTATTGCTCAAAGAGACATG<br>GGTGGAAGAGATGAAGGTTACGATTGGTTGATTATGACACCCGGTGTGGGTTTAGA<br>TGACAAGGGAGACGCATTGGGTCAACAGTATAGAACCGTGGATGATGTCGTTTCTA<br>CAGGATCTGACATTATTATTGTTGGAAGAGGACTATTTGCAAAGGGAAGGGATGCT<br>AAGGTAGAGGGTGAACGTTACAGAAAAGCAGGCTGGGAAGCATATTTGAGAAGAT<br>GCGGCCAGCAAAACTAAAAAACTGTATTATAAGTAAATGCATGTATACTAAACTCAC<br>AAATTAGAGCTTCAATTTAATTATATCAGTTATTACCCTGTGCTCGAGTCTTTGAAAA<br>GATAATGTATGATTATGCTTTCACTCATATTTATACAGAAACTTGATGTTTTCTTTCG<br>AGTATATACAAGGTGATTACATGTACGTTTGAAGTACAACTCTAGATTTTGTAGTGC<br>CCTCTTGGGCTAGCGGTAAAGGTGCGCATTTTTTCACACCCTACAATGTTCTGTTC<br>AAAAGATTTTGGTCAAACGCTGTAGAAGTGAAAGTTGGTGCGCATGTTTCGGCGTT<br>CGAAACTTCTCCGCAGTGAAAGATAAATGATCGGGTTTTAgAGCTATGCTGTTTTGA<br>ATGGTCCCAAATGGCCCTGATAATAGTATGAAGAGCTATGCTGTTTTGAATGGTCC<br>CAAAACTTTTTTTGTTTTTTATGTCTGCATAGCTTCAAAATGTTTCTACTCCTTTTTTA<br>CTCTTCCAGATTTTCTCGGACTCCGCGCATCGCCGTACCACTTCAAAACACCCAAG<br>CACAGCATACTAAATTTCCCCTCTTTCTTCCTCTAGGGTGTCGTTAATTACCCGTAC<br>TAAAGGTTTGGAAAAGAAAAAAGAGGCCGCCTCGTTTCTTTTTCTTCGTCGAAAAAG<br>GCAATAAAAATTTTTATCACGTTTCTTTTTCTTGAAAATTTTTTTTTTTGATTTTTTTCT<br>CTTTCGATGACCTCCCATTGATATTTAAGTTAATAAACGGTCTTCAATTTCTCAAGTT<br>TCAGTTTCATTTTTCTTGTTCTATTACAACTTTTTTTACTTCTTGCTCATTAGAAAGAA<br>AGCATAGCAATCTAATCTAAGTTTTCTAGATGGATTATAAAGATGACGATGACAAAC<br>CTCCAAAAAAGAAGAGAAAGGTCGATAAGAAATACTCAATAGGCTTAGATATCGGC<br>ACAAATAGCGTCGGATGGGCGGTGATCACTGATGAATATAAGGTTCCGTCTAAAAA<br>GTTCAAGGTTCTGGGAAATACAGACCGCCACAGTATCAAAAAAAATCTTATAGGGG<br>CTCTTTTATTTGACAGTGGAGAGACAGCGGAAGCGACTCGTCTCAAACGGACAGCT<br>CGTAGAAGGTATACACGTCGGAAGAATCGTATTTGTTATCTACAGGAGATTTTTTCA<br>AATGAGATGGCGAAAGTAGATGATAGTTTCTTTCATCGACTTGAAGAGTCTTTTTTG<br>GTGGAAGAAGACAAGAAGCATGAACGTCATCCTATTTTTGGAAATATAGTAGATGA<br>AGTTGCTTATCATGAGAAATATCCAACTATCTATCATCTGCGAAAAAAATTGGTAGA<br>TTCTACTTATAAAGCGGATTTGCGCTTAATCTATTTGGCCTTAGCGCATATGATTAA<br>GTTTCGTGGTCATTTTTTGATTGAGGGAGATTTAAATCCTGATAATAGTGATGTGGA<br>CAAACTATTTATCCAGTTGGTACAAACCTACAATCAATTATTTGAAGAAAACCCTATT<br>AACGCAAGTGGAGTAGATGCTAAAGCGATTCTTTCTGCACGATTGAGTAAATCAAG<br>ACGATTAGAAAATCTCATTGCTCAGCTCCCCGGTGAGAAGAAAAATGGCTTATTTG<br>GGAATCTCATTGCTTTGTCATTGGGTTTGACCCCTAATTTTAAATCAAATTTTGATTT<br>GGCAGAAGATGCTAAATTACAGCTTTCAAAAGATACTTACGATGATGATTTAGATAA<br>TTTATTGGCGCAAATTGGAGATCAATATGCTGATTTGTTTTTGGCAGCTAAGAATTT<br>ATCAGATGCTATTTTACTTTCAGATATCCTAAGAGTAAATACTGAAATAACTAAGGCT<br>CCCCTATCAGCTTCAATGATTAAACGCTACGATGAACATCATCAAGACTTGACTCTT<br>TTAAAAGCTTTAGTTCGACAACAACTTCCAGAAAAGTATAAAGAAATCTTTTTTGATC<br>AATCAAAAAACGGATATGCAGGTTATATTGATGGGGGAGCTAGCCAAGAAGAATTT<br>TATAAATTTATCAAACCAATTTTAGAAAAAATGGATGGTACTGAGGAATTATTGGTGA<br>AACTAAATCGTGAAGATTTGCTGCGCAAGCAACGGACCTTTGACAACGGCTCTATT<br>ACCCATCAAATTCACTTGGGTGAGCTGCATGCTATTTTGAGAAGACAAGAAGACTTT

	TATCCATTTTTAAAAGACAATCGTGAGAAGATTGAAAAAATCTTGACTTTTCGAATTC<br>CTTATTATGTTGGTCCATTGGCGCGTGGCAATAGTCGTTTTGCATGGATGACTCGG<br>AAGTCTGAAGAAACAATTACCCCATGGAATTTTGAAGAAGTTGTCGATAAAGGTGCT<br>TCAGCTCAATCATTTATTGAACGCATGACAAACTTTGATAAAAATCTTCCAAATGAAA<br>AAGTACTACCAAAACATAGTTTGCTTTATGAGTATTTTACGGTTTATAACGAATTGAC<br>AAAGGTCAAATATGTTACTGAAGGAATGCGAAAACCAGCATTTCTTTCAGGTGAACA<br>GAAGAAAGCCATTGTTGATTTACTCTTCAAAACAAATCGAAAAGTAACCGTTAAGCA<br>ATTAAAAGAAGATTATTTCAAAAAAATAGAATGTTTTGATAGTGTTGAAATTTCAGGA<br>GTTGAAGATAGATTTAATGCTTCATTAGGTACCTACCATGATTTGCTAAAAATTATTA<br>AAGATAAAGATTTTTTGGATAATGAAGAAAATGAAGATATCTTAGAGGATATTGTTTT<br>AACATTGACCTTATTTGAAGATAGGGAGATGATTGAGGAAAGACTTAAAACATATGC<br>TCACCTCTTTGATGATAAGGTGATGAAACAGCTTAAACGTCGCCGTTATACTGGTTG<br>GGGACGTTTGTCTCGAAAATTGATTAATGGTATTAGGGATAAGCAATCTGGCAAAA<br>CAATATTAGATTTTTTGAAATCAGATGGTTTTGCCAATCGCAATTTTATGCAGCTGAT<br>CCATGATGATAGTTTGACATTTAAAGAAGACATTCAAAAAGCACAAGTGTCTGGACA<br>AGGCGATAGTTTACATGAACATATTGCAAATTTAGCTGGTAGCCCTGCTATTAAAAA<br>AGGTATTTTACAGACTGTAAAAGTTGTTGATGAATTGGTCAAAGTAATGGGGCGGC<br>ATAAGCCAGAAAATATCGTTATTGAAATGGCACGTGAAAATCAGACAACTCAAAAG<br>GGCCAGAAAAATTCGCGAGAGCGTATGAAACGAATCGAAGAAGGTATCAAAGAATT<br>AGGAAGTCAGATTCTTAAAGAGCATCCTGTTGAAAATACTCAATTGCAAAATGAAAA<br>GCTCTATCTCTATTATCTCCAAAATGGAAGAGACATGTATGTGGACCAAGAATTAGA<br>TATTAATCGTTTAAGTGATTATGATGTCGATCACATTGTTCCACAAAGTTTCCTTAAA<br>GACGATTCAATAGACAATAAGGTCTTAACGCGTTCTGATAAAAATCGTGGTAAATCG<br>GATAACGTTCCAAGTGAAGAAGTAGTCAAAAAGATGAAAAACTATTGGAGACAACTT<br>CTAAACGCCAAGTTAATCACTCAACGTAAGTTTGATAATTTAACGAAAGCTGAACGT<br>GGAGGTTTGAGTGAACTTGATAAAGCTGGTTTTATCAAACGCCAATTGGTTGAAACT<br>CGCCAAATCACTAAGCATGTGGCACAAATTTTGGATAGTCGCATGAATACTAAATAC<br>GATGAAAATGATAAACTTATTCGAGAGGTTAAAGTGATTACCTTAAAATCTAAATTAG<br>TTTCTGACTTCCGAAAAGATTTCCAATTCTATAAAGTACGTGAGATTAACAATTACCA<br>TCATGCCCATGATGCGTATCTAAATGCCGTCGTTGGAACTGCTTTGATTAAGAAATA<br>TCCAAAACTTGAATCGGAGTTTGTCTATGGTGATTATAAAGTTTATGATGTTCGTAA<br>AATGATTGCTAAGTCTGAGCAAGAAATAGGCAAAGCAACCGCAAAATATTTCTTTTA<br>CTCTAATATCATGAACTTCTTCAAAACAGAAATTACACTTGCAAATGGAGAGATTCG<br>CAAACGCCCTCTAATCGAAACTAATGGGGAAACTGGAGAAATTGTCTGGGATAAAG<br>GGCGAGATTTTGCCACAGTGCGCAAAGTATTGTCCATGCCCCAAGTCAATATTGTC<br>AAGAAAACAGAAGTACAGACAGGCGGATTCTCCAAGGAGTCAATTTTACCAAAAAG<br>AAATTCGGACAAGCTTATTGCTCGTAAAAAAGACTGGGATCCAAAAAAATATGGTG<br>GTTTTGATAGTCCAACGGTAGCTTATTCAGTCCTAGTGGTTGCTAAGGTGGAAAAA<br>GGGAAATCGAAGAAGTTAAAATCCGTTAAAGAGTTACTAGGGATCACAATTATGGA<br>AAGAAGTTCCTTTGAAAAAAATCCGATTGACTTTTTAGAAGCTAAAGGATATAAGGA<br>AGTTAAAAAAGACTTAATCATTAAACTACCTAAATATAGTCTTTTTGAGTTAGAAAAC<br>GGTCGTAAACGGATGCTGGCTAGTGCCGGAGAATTACAAAAAGGAAATGAGCTGG<br>CTCTGCCAAGCAAATATGTGAATTTTTTATATTTAGCTAGTCATTATGAAAAGTTGAA<br>GGGTAGTCCAGAAGATAACGAACAAAAACAATTGTTTGTGGAGCAGCATAAGCATT<br>ATTTAGATGAGATTATTGAGCAAATCAGTGAATTTTCTAAGCGTGTTATTTTAGCAGA<br>TGCCAATTTAGATAAAGTTCTTAGTGCATATAACAAACATAGAGACAAACCAATACG<br>TGAACAAGCAGAAAATATTATTCATTTATTTACGTTGACGAATCTTGGAGCTCCCGC<br>TGCTTTTAAATATTTTGATACAACAATTGATCGTAAACGATATACGTCTACAAAAGAA<br>GTTTTAGATGCCACTCTTATCCATCAATCCATCACTGGTCTTTATGAAACACGCATT<br>GATTTGAGTCAGCTAGGAGGTGACCCTCCAAAAAAGAAGAGAAAGGTCTGAGCGG<br>ATCTCTTATGTCTTTACGATTTATAGTTTTCATTATCAAGTATGCCTATATTAGTATAT<br>AGCATCTTTAGATGACAGTGTTCGAAGTTTCACGAATAAAAGATAATATTCTACTTTT<br>TGCTCCCACCGCGTTTGCTAGCACGAGTGAACACCATCCCTCGCCTGTGAGTTGTA<br>CCCATTCCTCTAAACTGTAGACATGGTAGCTTCAGCAGTGTTCGTTATGTACGGCA<br>TCCTCCAACAAACAGTCGGTTATAGTTTGTCCTGCTCCTCTGAATCGAGTCCCTCG<br>ATATTTCTCATACTAGTTCTAGAGATCTGCCAATTGAACATAACATGGTAGTTACATA<br>TACTAGTAATATGGTTCGGCACACATTAAAAGTATAAAAACTATCTGAATTACGAATT<br>ACATATATTGGTCATAAAAATCAATCAATCATCGTGTGTTTTATATGTCTCTTATCTA<br>AGTATAAGAATATCCATAGTTAATATTCACTTACGCTACCTTTTAACCTGTAATCATT<br>GTCAACAGGATATGTTAACGACCCACATTGATAAACGCTAGTATTTCTTTTTCCTCT<br>TCTTATTGGCCGGCTGTCTCTATACTCCCCTATAGTCTGTTTCTTTTCGTTTCGATT

GTTTTACGTTTGAGGCCTCGTGGCGCACATGGTACGCTGTGGTGCTCGCGGCTGG
GAACGAAACTCTGGGAGCTGCGATTGGCAGGAACCATTCAAAACAGCATAGCAAG
TTAAAATAAGGCTAGTCCGTTATCAACTTGAAAAAGTGGCACCGAGTCGGTGCTTTT
TTTCCATATCCAACTTCCAATTTAATCTTTCTTTTTTAATTTTCACTTATTTGCGATAC
AGAAAGACCCTGCAGGCATGCAAGCTTGGCGTAATCATGGTCATAGCTGTTTCCTG
TGTGAAATTGTTATCCGCTCACAATTCCACACAACATACGAGCCGGAAGCATAAAG
TGTAAAGCCTGGGGTGCCTAATGAGTGAGCTAACTCACATTAATTGCGTTGCGCTC
ACTGCCCGCTTTCCAGTCGGGAAACCTGTCGTGCCAGCTGCATTAATGAATCGGC
CAACGCGCGGGGAGAGGCGGTTTGCGTATTGGGCGCTCTTCCGCTTCCTCGCTCA
CTGACTCGCTGCGCTCGGTCGTTCGGCTGCGGCGAGCGGTATCAGCTCACTCAAA
GGCGGTAATACGGTTATCCACAGAATCAGGGGATAACGCAGGAAAGAACATGTGA
GCAAAAGGCCAGCAAAAGGCCAGGAACCGTAAAAAGGCCGCGTTGCTGGCGTTTT
TCCATAGGCTCCGCCCCCCTGACGAGCATCACAAAAATCGACGCTCAAGTCAGAG
GTGGCGAAACCCGACAGGACTATAAAGATACCAGGCGTTTCCCCCTGGAAGCTCC
CTCGTGCGCTCTCCTGTTCCGACCCTGCCGCTTACCGGATACCTGTCCGCCTTTCT
CCCTTCGGGAAGCGTGGCGCTTTCTCATAGCTCACGCTGTAGGTATCTCAGTTCG
GTGTAGGTCGTTCGCTCCAAGCTGGGCTGTGTGCACGAACCCCCCGTTCAGCCCG
ACCGCTGCGCCTTATCCGGTAACTATCGTCTTGAGTCCAACCCGGTAAGACACGAC
TTATCGCCACTGGCAGCAGCCACTGGTAACAGGATTAGCAGAGCGAGGTATGTAG
GCGGTGCTACAGAGTTCTTGAAGTGGTGGCCTAACTACGGCTACACTAGAAGAAC
AGTATTTGGTATCTGCGCTCTGCTGAAGCCAGTTACCTTCGGAAAAAGAGTTGGTA
GCTCTTGATCCGGCAAACAAACCACCGCTGGTAGCGGTGGTTTTTTTGTTTGCAAG
CAGCAGATTACGCGCAGAAAAAAAGGATCTCAAGAAGATCCTTTGATCTTTTCTAC
GGGGTCTGACGCTCAGTGGAACGAAAACTCACGTTAAGGGATTTTGGTCATGAGAT
TATCAAAAAGGATCTTCACCTAGATCCTTTTAAATTAAAAATGAAGTTTTAAATCAAT
CTAAAGTATATATGAGTAAACTTGGTCTGACAGTTACCAATGCTTAATCAGTGAGGC
ACCTATCTCAGCGATCTGTCTATTTCGTTCATCCATAGTTGCCTGACTCCCCGTCGT
GTAGATAACTACGATACGGGAGGGCTTACCATCTGGCCCCAGTGCTGCAATGATA
CCGCGGCTCCCACGCTCACCGGCTCCAGATTTATCAGCAATAAACCAGCCAGCCG
GAAGGGCCGAGCGCAGAAGTGGTCCTGCAACTTTATCCGCCTCCATCCAGTCTAT
TAATTGTTGCCGGGAAGCTAGAGTAAGTAGTTCGCCAGTTAATAGTTTGCGCAACG
TTGTTGCCATTGCTACAGGCATCGTGGTGTCACGCTCGTCGTTTGGTATGGCTTCA
TTCAGCTCCGGTTCCCAACGATCAAGGCGAGTTACATGATCCCCCATGTTGTGCAA
AAAAGCGGTTAGCTCCTTCGGTCCTCCGATCGTTGTCAGAAGTAAGTTGGCCGCA
GTGTTATCACTCATGGTTATGGCAGCACTGCATAATTCTCTTACTGTCATGCCATCC
GTAAGATGCTTTTCTGTGACTGGTGAGTACTCAACCAAGTCATTCTGAGAATAGTGT
ATGCGGCGACCGAGTTGCTCTTGCCCGGCGTCAATACGGGATAATACCGCGCCAC
ATAGCAGAACTTTAAAAGTGCTCATCATTGGAAAACGTTCTTCGGGGCGAAAACTC
TCAAGGATCTTACCGCTGTTGAGATCCAGTTCGATGTAACCCACTCGTGCACCCAA
CTGATCTTCAGCATCTTTTACTTTCACCAGCGTTTCTGGGTGAGCAAAAACAGGAA
GGCAAAATGCCGCAAAAAAGGGAATAAGGGCGACACGGAAATGTTGAATACTCATA
CTCTTCCTTTTTCAATATTATTGAAGCATTTATCAGGGTTATTGTCTCATGAGCGGAT
ACATATTTGAATGTATTTAGAAAAATAAACAAATAGGGGTTCCGCGCACATTTCCCC
GAAAAGTGCCACCTGAACGAAGCATCTGTGCTTCATTTTGTAGAACAAAAATGCAA
CGCGAGAGCGCTAATTTTTCAAACAAAGAATCTGAGCTGCATTTTTACAGAACAGAA
ATGCAACGCGAAAGCGCTATTTTACCAACGAAGAATCTGTGCTTCATTTTTGTAAAA
CAAAAATGCAACGCGAGAGCGCTAATTTTTCAAACAAAGAATCTGAGCTGCATTTTT
ACAGAACAGAAATGCAACGCGAGAGCGCTATTTTACCAACAAAGAATCTATACTTCT
TTTTTGTTCTACAAAAATGCATCCCGAGAGCGCTATTTTTCTAACAAAGCATCTTAG
ATTACTTTTTTTCTCCTTTGTGCGCTCTATAATGCAGTCTCTTGATAACTTTTTGCAC
TGTAGGTCCGTTAAGGTTAGAAGAAGGCTACTTTGGTGTCTATTTTCTCTTCCATAA
AAAAAGCCTGACTCCACTTCCCGCGTTTACTGATTACTAGCGAAGCTGCGGGTGCA
TTTTTTCAAGATAAAGGCATCCCCGATTATATTCTATACCGATGTGGATTGCGCATA
CTTTGTGAACAGAAAGTGATAGCGTTGATGATTCTTCATTGGTCAGAAAATTATGAA
CGGTTTCTTCTATTTTGTCTCTATATACTACGTATAGGAAATGTTTACATTTTCGTAT
TGTTTTCGATTCACTCTATGAATAGTTCTTACTACAATTTTTTTGTCTAAAGAGTAATA
CTAGAGATAAACATAAAAAATGTAGAGGTCGAGTTTAGATGCAAGTTCAAGGAGCG
AAAGGTGGATGGGTAGGTTATATAGGGATATAGCACAGAGATATATAGCAAAGAGA
TACTTTTGAGCAATGTTTGTGGAAGCGGTATTCGCAATATTTTAGTAGCTCGTTACA
GTCCGGTGCGTTTTTGGTTTTTTGAAAGTGCGTCTTCAGAGCGCTTTTGGTTTTCAA
AAGCGCTCTGAAGTTCCTATACTTTCTAGAGAATAGGAACTTCGGAATAGGAACTTC

	AAAGCGTTTCCGAAAACGAGCGCTTCCGAAAATGCAACGCGAGCTGCGCACATAC<br>AGCTCACTGTTCACGTCGCACCTATATCTGCGTGTTGCCTGTATATATATATACATG<br>AGAAGAACGGCATAGTGCGTGTTTATGCTTAAATGCGTACTTATATGCGTCTATTTA<br>TGTAGGATGAAAGGTAGTCTAGTACCTCCTGTGATATTATCCCATTCCATGCGGGG<br>TATCGTATGCTTCCTTCAGCACTACCCTTTAGCTGTTCTATATGCTGCCACTCCTCA<br>ATTGGATTAGTCTCATCCTTCAATGCTATCATTTCCTTTGATATTGGATCATACTAAG<br>AAACCATTATTATCATGACATTAACCTATAAAAATAGGCGTATCACGAGGCCCTTTC<br>GTC
pHV004	TCGCGCGAACCCAACTGCACAGAACAAAAACCTGCAGGAAACGAAGATAAATCATG<br>TCGAAAGCTACATATAAGGAACGTGCTGCTACTCATCCTAGTCCTGTTGCTGCCAA<br>GCTATTTAATATCATGCACGAAAAGCAAACAAACTTGTGTGCTTCATTGGATGTTCG<br>TACCACCAAGGAATTACTGGAGTTAGTTGAAGCATTAGGTCCCAAAATTTGTTTACT<br>AAAAACACATGTGGATATCTTGACTGATTTTTCCATGGAGGGCACAGTTAAGCCGC<br>TAAAGGCATTATCCGCCAAGTACAATTTTTTACTCTTCGAAGACAGAAAATTTGCTG<br>ACATTGGTAATACAGTCAAATTGCAGTACTCTGCGGGTGTATACAGAATAGCAGAA<br>TGGGCAGACATTACGAATGCACACGGTGTGGTGGGCCCAGGTATTGTTAGCGGTT<br>TGAAGCAGGCGGCAGAAGAAGTAACAAAGGAACCTAGAGGCCTTTTGATGTTAGC<br>AGAATTGTCATGCAAGGGCTCCCTATCTACTGGAGAATATACTAAGGGTACTGTTG<br>ACATTGCGAAGAGCGACAAAGATTTTGTTATCGGCTTTATTGCTCAAAGAGACATG<br>GGTGGAAGAGATGAAGGTTACGATTGGTTGATTATGACACCCGGTGTGGGTTTAGA<br>TGACAAGGGAGACGCATTGGGTCAACAGTATAGAACCGTGGATGATGTCGTTTCTA<br>CAGGATCTGACATTATTATTGTTGGAAGAGGACTATTTGCAAAGGGAAGGGATGCT<br>AAGGTAGAGGGTGAACGTTACAGAAAAGCAGGCTGGGAAGCATATTTGAGAAGAT<br>GCGGCCAGCAAAACTAAAAAACTGTATTATAAGTAAATGCATGTATACTAAACTCAC<br>AAATTAGAGCTTCAATTTAATTATATCAGTTATTACCCTGTGCTCGAGTCTTTGAAAA<br>GATAATGTATGATTATGCTTTCACTCATATTTATACAGAAACTTGATGTTTTCTTTCG<br>AGTATATACAAGGTGATTACATGTACGTTTGAAGTACAACTCTAGATTTTGTAGTGC<br>CCTCTTGGGCTAGCGGTAAAGGTGCGCATTTTTTCACACCCTACAATGTTCTGTTC<br>AAAAGATTTTGGTCAAACGCTGTAGAAGTGAAAGTTGGTGCGCATGTTTCGGCGTT<br>CGAAACTTCTCCGCAGTGAAAGATAAATGATCGGGTTTTAgAGCTATGCTGTTTTGA<br>ATGGTCCCAAAGTATCACAACCGACGATCCGAGAGCTATGCTGTTTTGAATGGTCC<br>CAAAACTTTTTTTGTTTTTTATGTCTGCATAGCTTCAAAATGTTTCTACTCCTTTTTTA<br>CTCTTCCAGATTTTCTCGGACTCCGCGCATCGCCGTACCACTTCAAAACACCCAAG<br>CACAGCATACTAAATTTCCCCTCTTTCTTCCTCTAGGGTGTCGTTAATTACCCGTAC<br>TAAAGGTTTGGAAAAGAAAAAAGAGGCCGCCTCGTTTCTTTTTCTTCGTCGAAAAAG<br>GCAATAAAAATTTTTATCACGTTTCTTTTTCTTGAAAATTTTTTTTTTTGATTTTTTTCT<br>CTTTCGATGACCTCCCATTGATATTTAAGTTAATAAACGGTCTTCAATTTCTCAAGTT<br>TCAGTTTCATTTTTCTTGTTCTATTACAACTTTTTTTACTTCTTGCTCATTAGAAAGAA<br>AGCATAGCAATCTAATCTAAGTTTTCTAGATGGATTATAAAGATGACGATGACAAAC<br>CTCCAAAAAAGAAGAGAAAGGTCGATAAGAAATACTCAATAGGCTTAGATATCGGC<br>ACAAATAGCGTCGGATGGGCGGTGATCACTGATGAATATAAGGTTCCGTCTAAAAA<br>GTTCAAGGTTCTGGGAAATACAGACCGCCACAGTATCAAAAAAAATCTTATAGGGG<br>CTCTTTTATTTGACAGTGGAGAGACAGCGGAAGCGACTCGTCTCAAACGGACAGCT<br>CGTAGAAGGTATACACGTCGGAAGAATCGTATTTGTTATCTACAGGAGATTTTTTCA<br>AATGAGATGGCGAAAGTAGATGATAGTTTCTTTCATCGACTTGAAGAGTCTTTTTTG<br>GTGGAAGAAGACAAGAAGCATGAACGTCATCCTATTTTTGGAAATATAGTAGATGA<br>AGTTGCTTATCATGAGAAATATCCAACTATCTATCATCTGCGAAAAAAATTGGTAGA<br>TTCTACTTATAAAGCGGATTTGCGCTTAATCTATTTGGCCTTAGCGCATATGATTAA<br>GTTTCGTGGTCATTTTTTGATTGAGGGAGATTTAAATCCTGATAATAGTGATGTGGA<br>CAAACTATTTATCCAGTTGGTACAAACCTACAATCAATTATTTGAAGAAAACCCTATT<br>AACGCAAGTGGAGTAGATGCTAAAGCGATTCTTTCTGCACGATTGAGTAAATCAAG<br>ACGATTAGAAAATCTCATTGCTCAGCTCCCCGGTGAGAAGAAAAATGGCTTATTTG<br>GGAATCTCATTGCTTTGTCATTGGGTTTGACCCCTAATTTTAAATCAAATTTTGATTT<br>GGCAGAAGATGCTAAATTACAGCTTTCAAAAGATACTTACGATGATGATTTAGATAA<br>TTTATTGGCGCAAATTGGAGATCAATATGCTGATTTGTTTTTGGCAGCTAAGAATTT<br>ATCAGATGCTATTTTACTTTCAGATATCCTAAGAGTAAATACTGAAATAACTAAGGCT<br>CCCCTATCAGCTTCAATGATTAAACGCTACGATGAACATCATCAAGACTTGACTCTT<br>TTAAAAGCTTTAGTTCGACAACAACTTCCAGAAAAGTATAAAGAAATCTTTTTTGATC<br>AATCAAAAAACGGATATGCAGGTTATATTGATGGGGGAGCTAGCCAAGAAGAATTT<br>TATAAATTTATCAAACCAATTTTAGAAAAAATGGATGGTACTGAGGAATTATTGGTGA<br>AACTAAATCGTGAAGATTTGCTGCGCAAGCAACGGACCTTTGACAACGGCTCTATT

	ACCCATCAAATTCACTTGGGTGAGCTGCATGCTATTTTGAGAAGACAAGAAGACTTT<br>TATCCATTTTTAAAAGACAATCGTGAGAAGATTGAAAAAATCTTGACTTTTCGAATTC<br>CTTATTATGTTGGTCCATTGGCGCGTGGCAATAGTCGTTTTGCATGGATGACTCGG<br>AAGTCTGAAGAAACAATTACCCCATGGAATTTTGAAGAAGTTGTCGATAAAGGTGCT<br>TCAGCTCAATCATTTATTGAACGCATGACAAACTTTGATAAAAATCTTCCAAATGAAA<br>AAGTACTACCAAAACATAGTTTGCTTTATGAGTATTTTACGGTTTATAACGAATTGAC<br>AAAGGTCAAATATGTTACTGAAGGAATGCGAAAACCAGCATTTCTTTCAGGTGAACA<br>GAAGAAAGCCATTGTTGATTTACTCTTCAAAACAAATCGAAAAGTAACCGTTAAGCA<br>ATTAAAAGAAGATTATTTCAAAAAAATAGAATGTTTTGATAGTGTTGAAATTTCAGGA<br>GTTGAAGATAGATTTAATGCTTCATTAGGTACCTACCATGATTTGCTAAAAATTATTA<br>AAGATAAAGATTTTTTGGATAATGAAGAAAATGAAGATATCTTAGAGGATATTGTTTT<br>AACATTGACCTTATTTGAAGATAGGGAGATGATTGAGGAAAGACTTAAAACATATGC<br>TCACCTCTTTGATGATAAGGTGATGAAACAGCTTAAACGTCGCCGTTATACTGGTTG<br>GGGACGTTTGTCTCGAAAATTGATTAATGGTATTAGGGATAAGCAATCTGGCAAAA<br>CAATATTAGATTTTTTGAAATCAGATGGTTTTGCCAATCGCAATTTTATGCAGCTGAT<br>CCATGATGATAGTTTGACATTTAAAGAAGACATTCAAAAAGCACAAGTGTCTGGACA<br>AGGCGATAGTTTACATGAACATATTGCAAATTTAGCTGGTAGCCCTGCTATTAAAAA<br>AGGTATTTTACAGACTGTAAAAGTTGTTGATGAATTGGTCAAAGTAATGGGGCGGC<br>ATAAGCCAGAAAATATCGTTATTGAAATGGCACGTGAAAATCAGACAACTCAAAAG<br>GGCCAGAAAAATTCGCGAGAGCGTATGAAACGAATCGAAGAAGGTATCAAAGAATT<br>AGGAAGTCAGATTCTTAAAGAGCATCCTGTTGAAAATACTCAATTGCAAAATGAAAA<br>GCTCTATCTCTATTATCTCCAAAATGGAAGAGACATGTATGTGGACCAAGAATTAGA<br>TATTAATCGTTTAAGTGATTATGATGTCGATCACATTGTTCCACAAAGTTTCCTTAAA<br>GACGATTCAATAGACAATAAGGTCTTAACGCGTTCTGATAAAAATCGTGGTAAATCG<br>GATAACGTTCCAAGTGAAGAAGTAGTCAAAAAGATGAAAAACTATTGGAGACAACTT<br>CTAAACGCCAAGTTAATCACTCAACGTAAGTTTGATAATTTAACGAAAGCTGAACGT<br>GGAGGTTTGAGTGAACTTGATAAAGCTGGTTTTATCAAACGCCAATTGGTTGAAACT<br>CGCCAAATCACTAAGCATGTGGCACAAATTTTGGATAGTCGCATGAATACTAAATAC<br>GATGAAAATGATAAACTTATTCGAGAGGTTAAAGTGATTACCTTAAAATCTAAATTAG<br>TTTCTGACTTCCGAAAAGATTTCCAATTCTATAAAGTACGTGAGATTAACAATTACCA<br>TCATGCCCATGATGCGTATCTAAATGCCGTCGTTGGAACTGCTTTGATTAAGAAATA<br>TCCAAAACTTGAATCGGAGTTTGTCTATGGTGATTATAAAGTTTATGATGTTCGTAA<br>AATGATTGCTAAGTCTGAGCAAGAAATAGGCAAAGCAACCGCAAAATATTTCTTTTA<br>CTCTAATATCATGAACTTCTTCAAAACAGAAATTACACTTGCAAATGGAGAGATTCG<br>CAAACGCCCTCTAATCGAAACTAATGGGGAAACTGGAGAAATTGTCTGGGATAAAG<br>GGCGAGATTTTGCCACAGTGCGCAAAGTATTGTCCATGCCCCAAGTCAATATTGTC<br>AAGAAAACAGAAGTACAGACAGGCGGATTCTCCAAGGAGTCAATTTTACCAAAAAG<br>AAATTCGGACAAGCTTATTGCTCGTAAAAAAGACTGGGATCCAAAAAAATATGGTG<br>GTTTTGATAGTCCAACGGTAGCTTATTCAGTCCTAGTGGTTGCTAAGGTGGAAAAA<br>GGGAAATCGAAGAAGTTAAAATCCGTTAAAGAGTTACTAGGGATCACAATTATGGA<br>AAGAAGTTCCTTTGAAAAAAATCCGATTGACTTTTTAGAAGCTAAAGGATATAAGGA<br>AGTTAAAAAAGACTTAATCATTAAACTACCTAAATATAGTCTTTTTGAGTTAGAAAAC<br>GGTCGTAAACGGATGCTGGCTAGTGCCGGAGAATTACAAAAAGGAAATGAGCTGG<br>CTCTGCCAAGCAAATATGTGAATTTTTTATATTTAGCTAGTCATTATGAAAAGTTGAA<br>GGGTAGTCCAGAAGATAACGAACAAAAACAATTGTTTGTGGAGCAGCATAAGCATT<br>ATTTAGATGAGATTATTGAGCAAATCAGTGAATTTTCTAAGCGTGTTATTTTAGCAGA<br>TGCCAATTTAGATAAAGTTCTTAGTGCATATAACAAACATAGAGACAAACCAATACG<br>TGAACAAGCAGAAAATATTATTCATTTATTTACGTTGACGAATCTTGGAGCTCCCGC<br>TGCTTTTAAATATTTTGATACAACAATTGATCGTAAACGATATACGTCTACAAAAGAA<br>GTTTTAGATGCCACTCTTATCCATCAATCCATCACTGGTCTTTATGAAACACGCATT<br>GATTTGAGTCAGCTAGGAGGTGACCCTCCAAAAAAGAAGAGAAAGGTCTGAGCGG<br>ATCTCTTATGTCTTTACGATTTATAGTTTTCATTATCAAGTATGCCTATATTAGTATAT<br>AGCATCTTTAGATGACAGTGTTCGAAGTTTCACGAATAAAAGATAATATTCTACTTTT<br>TGCTCCCACCGCGTTTGCTAGCACGAGTGAACACCATCCCTCGCCTGTGAGTTGTA<br>CCCATTCCTCTAAACTGTAGACATGGTAGCTTCAGCAGTGTTCGTTATGTACGGCA<br>TCCTCCAACAAACAGTCGGTTATAGTTTGTCCTGCTCCTCTGAATCGAGTCCCTCG<br>ATATTTCTCATACTAGTTCTAGAGATCTGCCAATTGAACATAACATGGTAGTTACATA<br>TACTAGTAATATGGTTCGGCACACATTAAAAGTATAAAAACTATCTGAATTACGAATT<br>ACATATATTGGTCATAAAAATCAATCAATCATCGTGTGTTTTATATGTCTCTTATCTA<br>AGTATAAGAATATCCATAGTTAATATTCACTTACGCTACCTTTTAACCTGTAATCATT<br>GTCAACAGGATATGTTAACGACCCACATTGATAAACGCTAGTATTTCTTTTTCCTCT

	TCTTATTGGCCGGCTGTCTCTATACTCCCCTATAGTCTGTTTCTTTTCGTTTCGATT<br>GTTTTACGTTTGAGGCCTCGTGGCGCACATGGTACGCTGTGGTGCTCGCGGCTGG<br>GAACGAAACTCTGGGAGCTGCGATTGGCAGGAACCATTCAAAACAGCATAGCAAG<br>TTAAAATAAGGCTAGTCCGTTATCAACTTGAAAAAGTGGCACCGAGTCGGTGCTTTT<br>TTTCCATATCCAACTTCCAATTTAATCTTTCTTTTTTAATTTTCACTTATTTGCGATAC<br>AGAAAGACCCTGCAGGCATGCAAGCTTGGCGTAATCATGGTCATAGCTGTTTCCTG<br>TGTGAAATTGTTATCCGCTCACAATTCCACACAACATACGAGCCGGAAGCATAAAG<br>TGTAAAGCCTGGGGTGCCTAATGAGTGAGCTAACTCACATTAATTGCGTTGCGCTC<br>ACTGCCCGCTTTCCAGTCGGGAAACCTGTCGTGCCAGCTGCATTAATGAATCGGC<br>CAACGCGCGGGGAGAGGCGGTTTGCGTATTGGGCGCTCTTCCGCTTCCTCGCTCA<br>CTGACTCGCTGCGCTCGGTCGTTCGGCTGCGGCGAGCGGTATCAGCTCACTCAAA<br>GGCGGTAATACGGTTATCCACAGAATCAGGGGATAACGCAGGAAAGAACATGTGA<br>GCAAAAGGCCAGCAAAAGGCCAGGAACCGTAAAAAGGCCGCGTTGCTGGCGTTTT<br>TCCATAGGCTCCGCCCCCCTGACGAGCATCACAAAAATCGACGCTCAAGTCAGAG<br>GTGGCGAAACCCGACAGGACTATAAAGATACCAGGCGTTTCCCCCTGGAAGCTCC<br>CTCGTGCGCTCTCCTGTTCCGACCCTGCCGCTTACCGGATACCTGTCCGCCTTTCT<br>CCCTTCGGGAAGCGTGGCGCTTTCTCATAGCTCACGCTGTAGGTATCTCAGTTCG<br>GTGTAGGTCGTTCGCTCCAAGCTGGGCTGTGTGCACGAACCCCCCGTTCAGCCCG<br>ACCGCTGCGCCTTATCCGGTAACTATCGTCTTGAGTCCAACCCGGTAAGACACGAC<br>TTATCGCCACTGGCAGCAGCCACTGGTAACAGGATTAGCAGAGCGAGGTATGTAG<br>GCGGTGCTACAGAGTTCTTGAAGTGGTGGCCTAACTACGGCTACACTAGAAGAAC<br>AGTATTTGGTATCTGCGCTCTGCTGAAGCCAGTTACCTTCGGAAAAAGAGTTGGTA<br>GCTCTTGATCCGGCAAACAAACCACCGCTGGTAGCGGTGGTTTTTTTGTTTGCAAG<br>CAGCAGATTACGCGCAGAAAAAAAGGATCTCAAGAAGATCCTTTGATCTTTTCTAC<br>GGGGTCTGACGCTCAGTGGAACGAAAACTCACGTTAAGGGATTTTGGTCATGAGAT<br>TATCAAAAAGGATCTTCACCTAGATCCTTTTAAATTAAAAATGAAGTTTTAAATCAAT<br>CTAAAGTATATATGAGTAAACTTGGTCTGACAGTTACCAATGCTTAATCAGTGAGGC<br>ACCTATCTCAGCGATCTGTCTATTTCGTTCATCCATAGTTGCCTGACTCCCCGTCGT<br>GTAGATAACTACGATACGGGAGGGCTTACCATCTGGCCCCAGTGCTGCAATGATA<br>CCGCGGCTCCCACGCTCACCGGCTCCAGATTTATCAGCAATAAACCAGCCAGCCG<br>GAAGGGCCGAGCGCAGAAGTGGTCCTGCAACTTTATCCGCCTCCATCCAGTCTAT<br>TAATTGTTGCCGGGAAGCTAGAGTAAGTAGTTCGCCAGTTAATAGTTTGCGCAACG<br>TTGTTGCCATTGCTACAGGCATCGTGGTGTCACGCTCGTCGTTTGGTATGGCTTCA<br>TTCAGCTCCGGTTCCCAACGATCAAGGCGAGTTACATGATCCCCCATGTTGTGCAA<br>AAAAGCGGTTAGCTCCTTCGGTCCTCCGATCGTTGTCAGAAGTAAGTTGGCCGCA<br>GTGTTATCACTCATGGTTATGGCAGCACTGCATAATTCTCTTACTGTCATGCCATCC<br>GTAAGATGCTTTTCTGTGACTGGTGAGTACTCAACCAAGTCATTCTGAGAATAGTGT<br>ATGCGGCGACCGAGTTGCTCTTGCCCGGCGTCAATACGGGATAATACCGCGCCAC<br>ATAGCAGAACTTTAAAAGTGCTCATCATTGGAAAACGTTCTTCGGGGCGAAAACTC<br>TCAAGGATCTTACCGCTGTTGAGATCCAGTTCGATGTAACCCACTCGTGCACCCAA<br>CTGATCTTCAGCATCTTTTACTTTCACCAGCGTTTCTGGGTGAGCAAAAACAGGAA<br>GGCAAAATGCCGCAAAAAAGGGAATAAGGGCGACACGGAAATGTTGAATACTCATA<br>CTCTTCCTTTTTCAATATTATTGAAGCATTTATCAGGGTTATTGTCTCATGAGCGGAT<br>ACATATTTGAATGTATTTAGAAAAATAAACAAATAGGGGTTCCGCGCACATTTCCCC<br>GAAAAGTGCCACCTGAACGAAGCATCTGTGCTTCATTTTGTAGAACAAAAATGCAA<br>CGCGAGAGCGCTAATTTTTCAAACAAAGAATCTGAGCTGCATTTTTACAGAACAGAA<br>ATGCAACGCGAAAGCGCTATTTTACCAACGAAGAATCTGTGCTTCATTTTTGTAAAA<br>CAAAAATGCAACGCGAGAGCGCTAATTTTTCAAACAAAGAATCTGAGCTGCATTTTT<br>ACAGAACAGAAATGCAACGCGAGAGCGCTATTTTACCAACAAAGAATCTATACTTCT<br>TTTTTGTTCTACAAAAATGCATCCCGAGAGCGCTATTTTTCTAACAAAGCATCTTAG<br>ATTACTTTTTTTCTCCTTTGTGCGCTCTATAATGCAGTCTCTTGATAACTTTTTGCAC<br>TGTAGGTCCGTTAAGGTTAGAAGAAGGCTACTTTGGTGTCTATTTTCTCTTCCATAA<br>AAAAAGCCTGACTCCACTTCCCGCGTTTACTGATTACTAGCGAAGCTGCGGGTGCA<br>TTTTTTCAAGATAAAGGCATCCCCGATTATATTCTATACCGATGTGGATTGCGCATA<br>CTTTGTGAACAGAAAGTGATAGCGTTGATGATTCTTCATTGGTCAGAAAATTATGAA<br>CGGTTTCTTCTATTTTGTCTCTATATACTACGTATAGGAAATGTTTACATTTTCGTAT<br>TGTTTTCGATTCACTCTATGAATAGTTCTTACTACAATTTTTTTGTCTAAAGAGTAATA<br>CTAGAGATAAACATAAAAAATGTAGAGGTCGAGTTTAGATGCAAGTTCAAGGAGCG<br>AAAGGTGGATGGGTAGGTTATATAGGGATATAGCACAGAGATATATAGCAAAGAGA<br>TACTTTTGAGCAATGTTTGTGGAAGCGGTATTCGCAATATTTTAGTAGCTCGTTACA<br>GTCCGGTGCGTTTTTGGTTTTTTGAAAGTGCGTCTTCAGAGCGCTTTTGGTTTTCAA

	AAGCGCTCTGAAGTTCCTATACTTTCTAGAGAATAGGAACTTCGGAATAGGAACTTC<br>AAAGCGTTTCCGAAAACGAGCGCTTCCGAAAATGCAACGCGAGCTGCGCACATAC<br>AGCTCACTGTTCACGTCGCACCTATATCTGCGTGTTGCCTGTATATATATATACATG<br>AGAAGAACGGCATAGTGCGTGTTTATGCTTAAATGCGTACTTATATGCGTCTATTTA<br>TGTAGGATGAAAGGTAGTCTAGTACCTCCTGTGATATTATCCCATTCCATGCGGGG<br>TATCGTATGCTTCCTTCAGCACTACCCTTTAGCTGTTCTATATGCTGCCACTCCTCA<br>ATTGGATTAGTCTCATCCTTCAATGCTATCATTTCCTTTGATATTGGATCATACTAAG<br>AAACCATTATTATCATGACATTAACCTATAAAAATAGGCGTATCACGAGGCCCTTTC<br>GTC
pHV005	TCGCGCGAACCCAACTGCACAGAACAAAAACCTGCAGGAAACGAAGATAAATCATG<br>TCGAAAGCTACATATAAGGAACGTGCTGCTACTCATCCTAGTCCTGTTGCTGCCAA<br>GCTATTTAATATCATGCACGAAAAGCAAACAAACTTGTGTGCTTCATTGGATGTTCG<br>TACCACCAAGGAATTACTGGAGTTAGTTGAAGCATTAGGTCCCAAAATTTGTTTACT<br>AAAAACACATGTGGATATCTTGACTGATTTTTCCATGGAGGGCACAGTTAAGCCGC<br>TAAAGGCATTATCCGCCAAGTACAATTTTTTACTCTTCGAAGACAGAAAATTTGCTG<br>ACATTGGTAATACAGTCAAATTGCAGTACTCTGCGGGTGTATACAGAATAGCAGAA<br>TGGGCAGACATTACGAATGCACACGGTGTGGTGGGCCCAGGTATTGTTAGCGGTT<br>TGAAGCAGGCGGCAGAAGAAGTAACAAAGGAACCTAGAGGCCTTTTGATGTTAGC<br>AGAATTGTCATGCAAGGGCTCCCTATCTACTGGAGAATATACTAAGGGTACTGTTG<br>ACATTGCGAAGAGCGACAAAGATTTTGTTATCGGCTTTATTGCTCAAAGAGACATG<br>GGTGGAAGAGATGAAGGTTACGATTGGTTGATTATGACACCCGGTGTGGGTTTAGA<br>TGACAAGGGAGACGCATTGGGTCAACAGTATAGAACCGTGGATGATGTCGTTTCTA<br>CAGGATCTGACATTATTATTGTTGGAAGAGGACTATTTGCAAAGGGAAGGGATGCT<br>AAGGTAGAGGGTGAACGTTACAGAAAAGCAGGCTGGGAAGCATATTTGAGAAGAT<br>GCGGCCAGCAAAACTAAAAAACTGTATTATAAGTAAATGCATGTATACTAAACTCAC<br>AAATTAGAGCTTCAATTTAATTATATCAGTTATTACCCTGTGCTCGAGTCTTTGAAAA<br>GATAATGTATGATTATGCTTTCACTCATATTTATACAGAAACTTGATGTTTTCTTTCG<br>AGTATATACAAGGTGATTACATGTACGTTTGAAGTACAACTCTAGATTTTGTAGTGC<br>CCTCTTGGGCTAGCGGTAAAGGTGCGCATTTTTTCACACCCTACAATGTTCTGTTC<br>AAAAGATTTTGGTCAAACGCTGTAGAAGTGAAAGTTGGTGCGCATGTTTCGGCGTT<br>CGAAACTTCTCCGCAGTGAAAGATAAATGATCGGGTTTTAgAGCTATGCTGTTTTGA<br>ATGGTCCCAAAAGTCGAACAAATATTAAACGAGAGCTATGCTGTTTTGAATGGTCC<br>CAAAACTTTTTTTGTTTTTTATGTCTGCATAGCTTCAAAATGTTTCTACTCCTTTTTTA<br>CTCTTCCAGATTTTCTCGGACTCCGCGCATCGCCGTACCACTTCAAAACACCCAAG<br>CACAGCATACTAAATTTCCCCTCTTTCTTCCTCTAGGGTGTCGTTAATTACCCGTAC<br>TAAAGGTTTGGAAAAGAAAAAAGAGGCCGCCTCGTTTCTTTTTCTTCGTCGAAAAAG<br>GCAATAAAAATTTTTATCACGTTTCTTTTTCTTGAAAATTTTTTTTTTTGATTTTTTTCT<br>CTTTCGATGACCTCCCATTGATATTTAAGTTAATAAACGGTCTTCAATTTCTCAAGTT<br>TCAGTTTCATTTTTCTTGTTCTATTACAACTTTTTTTACTTCTTGCTCATTAGAAAGAA<br>AGCATAGCAATCTAATCTAAGTTTTCTAGATGGATTATAAAGATGACGATGACAAAC<br>CTCCAAAAAAGAAGAGAAAGGTCGATAAGAAATACTCAATAGGCTTAGATATCGGC<br>ACAAATAGCGTCGGATGGGCGGTGATCACTGATGAATATAAGGTTCCGTCTAAAAA<br>GTTCAAGGTTCTGGGAAATACAGACCGCCACAGTATCAAAAAAAATCTTATAGGGG<br>CTCTTTTATTTGACAGTGGAGAGACAGCGGAAGCGACTCGTCTCAAACGGACAGCT<br>CGTAGAAGGTATACACGTCGGAAGAATCGTATTTGTTATCTACAGGAGATTTTTTCA<br>AATGAGATGGCGAAAGTAGATGATAGTTTCTTTCATCGACTTGAAGAGTCTTTTTTG<br>GTGGAAGAAGACAAGAAGCATGAACGTCATCCTATTTTTGGAAATATAGTAGATGA<br>AGTTGCTTATCATGAGAAATATCCAACTATCTATCATCTGCGAAAAAAATTGGTAGA<br>TTCTACTTATAAAGCGGATTTGCGCTTAATCTATTTGGCCTTAGCGCATATGATTAA<br>GTTTCGTGGTCATTTTTTGATTGAGGGAGATTTAAATCCTGATAATAGTGATGTGGA<br>CAAACTATTTATCCAGTTGGTACAAACCTACAATCAATTATTTGAAGAAAACCCTATT<br>AACGCAAGTGGAGTAGATGCTAAAGCGATTCTTTCTGCACGATTGAGTAAATCAAG<br>ACGATTAGAAAATCTCATTGCTCAGCTCCCCGGTGAGAAGAAAAATGGCTTATTTG<br>GGAATCTCATTGCTTTGTCATTGGGTTTGACCCCTAATTTTAAATCAAATTTTGATTT<br>GGCAGAAGATGCTAAATTACAGCTTTCAAAAGATACTTACGATGATGATTTAGATAA<br>TTTATTGGCGCAAATTGGAGATCAATATGCTGATTTGTTTTTGGCAGCTAAGAATTT<br>ATCAGATGCTATTTTACTTTCAGATATCCTAAGAGTAAATACTGAAATAACTAAGGCT<br>CCCCTATCAGCTTCAATGATTAAACGCTACGATGAACATCATCAAGACTTGACTCTT<br>TTAAAAGCTTTAGTTCGACAACAACTTCCAGAAAAGTATAAAGAAATCTTTTTTGATC<br>AATCAAAAAACGGATATGCAGGTTATATTGATGGGGGAGCTAGCCAAGAAGAATTT<br>TATAAATTTATCAAACCAATTTTAGAAAAAATGGATGGTACTGAGGAATTATTGGTGA

	AACTAAATCGTGAAGATTTGCTGCGCAAGCAACGGACCTTTGACAACGGCTCTATT<br>ACCCATCAAATTCACTTGGGTGAGCTGCATGCTATTTTGAGAAGACAAGAAGACTTT<br>TATCCATTTTTAAAAGACAATCGTGAGAAGATTGAAAAAATCTTGACTTTTCGAATTC<br>CTTATTATGTTGGTCCATTGGCGCGTGGCAATAGTCGTTTTGCATGGATGACTCGG<br>AAGTCTGAAGAAACAATTACCCCATGGAATTTTGAAGAAGTTGTCGATAAAGGTGCT<br>TCAGCTCAATCATTTATTGAACGCATGACAAACTTTGATAAAAATCTTCCAAATGAAA<br>AAGTACTACCAAAACATAGTTTGCTTTATGAGTATTTTACGGTTTATAACGAATTGAC<br>AAAGGTCAAATATGTTACTGAAGGAATGCGAAAACCAGCATTTCTTTCAGGTGAACA<br>GAAGAAAGCCATTGTTGATTTACTCTTCAAAACAAATCGAAAAGTAACCGTTAAGCA<br>ATTAAAAGAAGATTATTTCAAAAAAATAGAATGTTTTGATAGTGTTGAAATTTCAGGA<br>GTTGAAGATAGATTTAATGCTTCATTAGGTACCTACCATGATTTGCTAAAAATTATTA<br>AAGATAAAGATTTTTTGGATAATGAAGAAAATGAAGATATCTTAGAGGATATTGTTTT<br>AACATTGACCTTATTTGAAGATAGGGAGATGATTGAGGAAAGACTTAAAACATATGC<br>TCACCTCTTTGATGATAAGGTGATGAAACAGCTTAAACGTCGCCGTTATACTGGTTG<br>GGGACGTTTGTCTCGAAAATTGATTAATGGTATTAGGGATAAGCAATCTGGCAAAA<br>CAATATTAGATTTTTTGAAATCAGATGGTTTTGCCAATCGCAATTTTATGCAGCTGAT<br>CCATGATGATAGTTTGACATTTAAAGAAGACATTCAAAAAGCACAAGTGTCTGGACA<br>AGGCGATAGTTTACATGAACATATTGCAAATTTAGCTGGTAGCCCTGCTATTAAAAA<br>AGGTATTTTACAGACTGTAAAAGTTGTTGATGAATTGGTCAAAGTAATGGGGCGGC<br>ATAAGCCAGAAAATATCGTTATTGAAATGGCACGTGAAAATCAGACAACTCAAAAG<br>GGCCAGAAAAATTCGCGAGAGCGTATGAAACGAATCGAAGAAGGTATCAAAGAATT<br>AGGAAGTCAGATTCTTAAAGAGCATCCTGTTGAAAATACTCAATTGCAAAATGAAAA<br>GCTCTATCTCTATTATCTCCAAAATGGAAGAGACATGTATGTGGACCAAGAATTAGA<br>TATTAATCGTTTAAGTGATTATGATGTCGATCACATTGTTCCACAAAGTTTCCTTAAA<br>GACGATTCAATAGACAATAAGGTCTTAACGCGTTCTGATAAAAATCGTGGTAAATCG<br>GATAACGTTCCAAGTGAAGAAGTAGTCAAAAAGATGAAAAACTATTGGAGACAACTT<br>CTAAACGCCAAGTTAATCACTCAACGTAAGTTTGATAATTTAACGAAAGCTGAACGT<br>GGAGGTTTGAGTGAACTTGATAAAGCTGGTTTTATCAAACGCCAATTGGTTGAAACT<br>CGCCAAATCACTAAGCATGTGGCACAAATTTTGGATAGTCGCATGAATACTAAATAC<br>GATGAAAATGATAAACTTATTCGAGAGGTTAAAGTGATTACCTTAAAATCTAAATTAG<br>TTTCTGACTTCCGAAAAGATTTCCAATTCTATAAAGTACGTGAGATTAACAATTACCA<br>TCATGCCCATGATGCGTATCTAAATGCCGTCGTTGGAACTGCTTTGATTAAGAAATA<br>TCCAAAACTTGAATCGGAGTTTGTCTATGGTGATTATAAAGTTTATGATGTTCGTAA<br>AATGATTGCTAAGTCTGAGCAAGAAATAGGCAAAGCAACCGCAAAATATTTCTTTTA<br>CTCTAATATCATGAACTTCTTCAAAACAGAAATTACACTTGCAAATGGAGAGATTCG<br>CAAACGCCCTCTAATCGAAACTAATGGGGAAACTGGAGAAATTGTCTGGGATAAAG<br>GGCGAGATTTTGCCACAGTGCGCAAAGTATTGTCCATGCCCCAAGTCAATATTGTC<br>AAGAAAACAGAAGTACAGACAGGCGGATTCTCCAAGGAGTCAATTTTACCAAAAAG<br>AAATTCGGACAAGCTTATTGCTCGTAAAAAAGACTGGGATCCAAAAAAATATGGTG<br>GTTTTGATAGTCCAACGGTAGCTTATTCAGTCCTAGTGGTTGCTAAGGTGGAAAAA<br>GGGAAATCGAAGAAGTTAAAATCCGTTAAAGAGTTACTAGGGATCACAATTATGGA<br>AAGAAGTTCCTTTGAAAAAAATCCGATTGACTTTTTAGAAGCTAAAGGATATAAGGA<br>AGTTAAAAAAGACTTAATCATTAAACTACCTAAATATAGTCTTTTTGAGTTAGAAAAC<br>GGTCGTAAACGGATGCTGGCTAGTGCCGGAGAATTACAAAAAGGAAATGAGCTGG<br>CTCTGCCAAGCAAATATGTGAATTTTTTATATTTAGCTAGTCATTATGAAAAGTTGAA<br>GGGTAGTCCAGAAGATAACGAACAAAAACAATTGTTTGTGGAGCAGCATAAGCATT<br>ATTTAGATGAGATTATTGAGCAAATCAGTGAATTTTCTAAGCGTGTTATTTTAGCAGA<br>TGCCAATTTAGATAAAGTTCTTAGTGCATATAACAAACATAGAGACAAACCAATACG<br>TGAACAAGCAGAAAATATTATTCATTTATTTACGTTGACGAATCTTGGAGCTCCCGC<br>TGCTTTTAAATATTTTGATACAACAATTGATCGTAAACGATATACGTCTACAAAAGAA<br>GTTTTAGATGCCACTCTTATCCATCAATCCATCACTGGTCTTTATGAAACACGCATT<br>GATTTGAGTCAGCTAGGAGGTGACCCTCCAAAAAAGAAGAGAAAGGTCTGAGCGG<br>ATCTCTTATGTCTTTACGATTTATAGTTTTCATTATCAAGTATGCCTATATTAGTATAT<br>AGCATCTTTAGATGACAGTGTTCGAAGTTTCACGAATAAAAGATAATATTCTACTTTT<br>TGCTCCCACCGCGTTTGCTAGCACGAGTGAACACCATCCCTCGCCTGTGAGTTGTA<br>CCCATTCCTCTAAACTGTAGACATGGTAGCTTCAGCAGTGTTCGTTATGTACGGCA<br>TCCTCCAACAAACAGTCGGTTATAGTTTGTCCTGCTCCTCTGAATCGAGTCCCTCG<br>ATATTTCTCATACTAGTTCTAGAGATCTGCCAATTGAACATAACATGGTAGTTACATA<br>TACTAGTAATATGGTTCGGCACACATTAAAAGTATAAAAACTATCTGAATTACGAATT<br>ACATATATTGGTCATAAAAATCAATCAATCATCGTGTGTTTTATATGTCTCTTATCTA<br>AGTATAAGAATATCCATAGTTAATATTCACTTACGCTACCTTTTAACCTGTAATCATT

	GTCAACAGGATATGTTAACGACCCACATTGATAAACGCTAGTATTTCTTTTTCCTCT<br>TCTTATTGGCCGGCTGTCTCTATACTCCCCTATAGTCTGTTTCTTTTCGTTTCGATT<br>GTTTTACGTTTGAGGCCTCGTGGCGCACATGGTACGCTGTGGTGCTCGCGGCTGG<br>GAACGAAACTCTGGGAGCTGCGATTGGCAGGAACCATTCAAAACAGCATAGCAAG<br>TTAAAATAAGGCTAGTCCGTTATCAACTTGAAAAAGTGGCACCGAGTCGGTGCTTTT<br>TTTCCATATCCAACTTCCAATTTAATCTTTCTTTTTTAATTTTCACTTATTTGCGATAC<br>AGAAAGACCCTGCAGGCATGCAAGCTTGGCGTAATCATGGTCATAGCTGTTTCCTG<br>TGTGAAATTGTTATCCGCTCACAATTCCACACAACATACGAGCCGGAAGCATAAAG<br>TGTAAAGCCTGGGGTGCCTAATGAGTGAGCTAACTCACATTAATTGCGTTGCGCTC<br>ACTGCCCGCTTTCCAGTCGGGAAACCTGTCGTGCCAGCTGCATTAATGAATCGGC<br>CAACGCGCGGGGAGAGGCGGTTTGCGTATTGGGCGCTCTTCCGCTTCCTCGCTCA<br>CTGACTCGCTGCGCTCGGTCGTTCGGCTGCGGCGAGCGGTATCAGCTCACTCAAA<br>GGCGGTAATACGGTTATCCACAGAATCAGGGGATAACGCAGGAAAGAACATGTGA<br>GCAAAAGGCCAGCAAAAGGCCAGGAACCGTAAAAAGGCCGCGTTGCTGGCGTTTT<br>TCCATAGGCTCCGCCCCCCTGACGAGCATCACAAAAATCGACGCTCAAGTCAGAG<br>GTGGCGAAACCCGACAGGACTATAAAGATACCAGGCGTTTCCCCCTGGAAGCTCC<br>CTCGTGCGCTCTCCTGTTCCGACCCTGCCGCTTACCGGATACCTGTCCGCCTTTCT<br>CCCTTCGGGAAGCGTGGCGCTTTCTCATAGCTCACGCTGTAGGTATCTCAGTTCG<br>GTGTAGGTCGTTCGCTCCAAGCTGGGCTGTGTGCACGAACCCCCCGTTCAGCCCG<br>ACCGCTGCGCCTTATCCGGTAACTATCGTCTTGAGTCCAACCCGGTAAGACACGAC<br>TTATCGCCACTGGCAGCAGCCACTGGTAACAGGATTAGCAGAGCGAGGTATGTAG<br>GCGGTGCTACAGAGTTCTTGAAGTGGTGGCCTAACTACGGCTACACTAGAAGAAC<br>AGTATTTGGTATCTGCGCTCTGCTGAAGCCAGTTACCTTCGGAAAAAGAGTTGGTA<br>GCTCTTGATCCGGCAAACAAACCACCGCTGGTAGCGGTGGTTTTTTTGTTTGCAAG<br>CAGCAGATTACGCGCAGAAAAAAAGGATCTCAAGAAGATCCTTTGATCTTTTCTAC<br>GGGGTCTGACGCTCAGTGGAACGAAAACTCACGTTAAGGGATTTTGGTCATGAGAT<br>TATCAAAAAGGATCTTCACCTAGATCCTTTTAAATTAAAAATGAAGTTTTAAATCAAT<br>CTAAAGTATATATGAGTAAACTTGGTCTGACAGTTACCAATGCTTAATCAGTGAGGC<br>ACCTATCTCAGCGATCTGTCTATTTCGTTCATCCATAGTTGCCTGACTCCCCGTCGT<br>GTAGATAACTACGATACGGGAGGGCTTACCATCTGGCCCCAGTGCTGCAATGATA<br>CCGCGGCTCCCACGCTCACCGGCTCCAGATTTATCAGCAATAAACCAGCCAGCCG<br>GAAGGGCCGAGCGCAGAAGTGGTCCTGCAACTTTATCCGCCTCCATCCAGTCTAT<br>TAATTGTTGCCGGGAAGCTAGAGTAAGTAGTTCGCCAGTTAATAGTTTGCGCAACG<br>TTGTTGCCATTGCTACAGGCATCGTGGTGTCACGCTCGTCGTTTGGTATGGCTTCA<br>TTCAGCTCCGGTTCCCAACGATCAAGGCGAGTTACATGATCCCCCATGTTGTGCAA<br>AAAAGCGGTTAGCTCCTTCGGTCCTCCGATCGTTGTCAGAAGTAAGTTGGCCGCA<br>GTGTTATCACTCATGGTTATGGCAGCACTGCATAATTCTCTTACTGTCATGCCATCC<br>GTAAGATGCTTTTCTGTGACTGGTGAGTACTCAACCAAGTCATTCTGAGAATAGTGT<br>ATGCGGCGACCGAGTTGCTCTTGCCCGGCGTCAATACGGGATAATACCGCGCCAC<br>ATAGCAGAACTTTAAAAGTGCTCATCATTGGAAAACGTTCTTCGGGGCGAAAACTC<br>TCAAGGATCTTACCGCTGTTGAGATCCAGTTCGATGTAACCCACTCGTGCACCCAA<br>CTGATCTTCAGCATCTTTTACTTTCACCAGCGTTTCTGGGTGAGCAAAAACAGGAA<br>GGCAAAATGCCGCAAAAAAGGGAATAAGGGCGACACGGAAATGTTGAATACTCATA<br>CTCTTCCTTTTTCAATATTATTGAAGCATTTATCAGGGTTATTGTCTCATGAGCGGAT<br>ACATATTTGAATGTATTTAGAAAAATAAACAAATAGGGGTTCCGCGCACATTTCCCC<br>GAAAAGTGCCACCTGAACGAAGCATCTGTGCTTCATTTTGTAGAACAAAAATGCAA<br>CGCGAGAGCGCTAATTTTTCAAACAAAGAATCTGAGCTGCATTTTTACAGAACAGAA<br>ATGCAACGCGAAAGCGCTATTTTACCAACGAAGAATCTGTGCTTCATTTTTGTAAAA<br>CAAAAATGCAACGCGAGAGCGCTAATTTTTCAAACAAAGAATCTGAGCTGCATTTTT<br>ACAGAACAGAAATGCAACGCGAGAGCGCTATTTTACCAACAAAGAATCTATACTTCT<br>TTTTTGTTCTACAAAAATGCATCCCGAGAGCGCTATTTTTCTAACAAAGCATCTTAG<br>ATTACTTTTTTTCTCCTTTGTGCGCTCTATAATGCAGTCTCTTGATAACTTTTTGCAC<br>TGTAGGTCCGTTAAGGTTAGAAGAAGGCTACTTTGGTGTCTATTTTCTCTTCCATAA<br>AAAAAGCCTGACTCCACTTCCCGCGTTTACTGATTACTAGCGAAGCTGCGGGTGCA<br>TTTTTTCAAGATAAAGGCATCCCCGATTATATTCTATACCGATGTGGATTGCGCATA<br>CTTTGTGAACAGAAAGTGATAGCGTTGATGATTCTTCATTGGTCAGAAAATTATGAA<br>CGGTTTCTTCTATTTTGTCTCTATATACTACGTATAGGAAATGTTTACATTTTCGTAT<br>TGTTTTCGATTCACTCTATGAATAGTTCTTACTACAATTTTTTTGTCTAAAGAGTAATA<br>CTAGAGATAAACATAAAAAATGTAGAGGTCGAGTTTAGATGCAAGTTCAAGGAGCG<br>AAAGGTGGATGGGTAGGTTATATAGGGATATAGCACAGAGATATATAGCAAAGAGA<br>TACTTTTGAGCAATGTTTGTGGAAGCGGTATTCGCAATATTTTAGTAGCTCGTTACA

	GTCCGGTGCGTTTTTGGTTTTTTGAAAGTGCGTCTTCAGAGCGCTTTTGGTTTTCAA<br>AAGCGCTCTGAAGTTCCTATACTTTCTAGAGAATAGGAACTTCGGAATAGGAACTTC<br>AAAGCGTTTCCGAAAACGAGCGCTTCCGAAAATGCAACGCGAGCTGCGCACATAC<br>AGCTCACTGTTCACGTCGCACCTATATCTGCGTGTTGCCTGTATATATATATATACATG<br>AGAAGAACGGCATAGTGCGTGTTTATGCTTAAATGCGTACTTATATGCGTCTATTTA<br>TGTAGGATGAAAGGTAGTCTAGTACCTCCTGTGATATTATCCCATTCCATGCGGGG<br>TATCGTATGCTTCCTTCAGCACTACCCTTTAGCTGTTCTATATGCTGCCACTCCTCA<br>ATTGGATTAGTCTCATCCTTCAATGCTATCATTTCCTTTGATATTGGATCATACTAAG<br>AAACCATTATTATCATGACATTAACCTATAAAAATAGGCGTATCACGAGGCCCTTTC<br>GTC
pHV006	TCGCGCGAACCCAACTGCACAGAACAAAAACCTGCAGGAAACGAAGATAAATCATG<br>TCGAAAGCTACATATAAGGAACGTGCTGCTACTCATCCTAGTCCTGTTGCTGCCAA<br>GCTATTTAATATCATGCACGAAAAGCAAACAAACTTGTGTGCTTCATTGGATGTTCG<br>TACCACCAAGGAATTACTGGAGTTAGTTGAAGCATTAGGTCCCAAAATTTGTTTACT<br>AAAAACACATGTGGATATCTTGACTGATTTTTCCATGGAGGGCACAGTTAAGCCGC<br>TAAAGGCATTATCCGCCAAGTACAATTTTTTACTCTTCGAAGACAGAAAATTTGCTG<br>ACATTGGTAATACAGTCAAATTGCAGTACTCTGCGGGTGTATACAGAATAGCAGAA<br>TGGGCAGACATTACGAATGCACACGGTGTGGTGGGCCCAGGTATTGTTAGCGGTT<br>TGAAGCAGGCGGCAGAAGAAGTAACAAAGGAACCTAGAGGCCTTTTGATGTTAGC<br>AGAATTGTCATGCAAGGGCTCCCTATCTACTGGAGAATATACTAAGGGTACTGTTG<br>ACATTGCGAAGAGCGACAAAGATTTTGTTATCGGCTTTATTGCTCAAAGAGACATG<br>GGTGGAAGAGATGAAGGTTACGATTGGTTGATTATGACACCCGGTGTGGGTTTAGA<br>TGACAAGGGAGACGCATTGGGTCAACAGTATAGAACCGTGGATGATGTCGTTTCTA<br>CAGGATCTGACATTATTATTGTTGGAAGAGGACTATTTGCAAAGGGAAGGGATGCT<br>AAGGTAGAGGGTGAACGTTACAGAAAAGCAGGCTGGGAAGCATATTTGAGAAGAT<br>GCGGCCAGCAAAACTAAAAAACTGTATTATAAGTAAATGCATGTATACTAAACTCAC<br>AAATTAGAGCTTCAATTTAATTATATCAGTTATTACCCTGTGCTCGAGTCTTTGAAAA<br>GATAATGTATGATTATGCTTTCACTCATATTTATACAGAAACTTGATGTTTTCTTTCG<br>AGTATATACAAGGTGATTACATGTACGTTTGAAGTACAACTCTAGATTTTGTAGTGC<br>CCTCTTGGGCTAGCGGTAAAGGTGCGCATTTTTTCACACCCTACAATGTTCTGTTC<br>AAAAGATTTTGGTCAAACGCTGTAGAAGTGAAAGTTGGTGCGCATGTTTCGGCGTT<br>CGAAACTTCTCCGCAGTGAAAGATAAATGATCGGGTTTTAgAGCTATGCTGTTTTGA<br>ATGGTCCCAAAATGACCAGTTAGATATCAAGAGAGCTATGCTGTTTTGAATGGTCC<br>CAAAACTTTTTTTGTTTTTTATGTCTGCATAGCTTCAAAATGTTTCTACTCCTTTTTA<br>CTCTTCCAGATTTTCTCGGACTCCGCGCATCGCCGTACCACTTCAAAACACCCAAG<br>CACAGCATACTAAATTTCCCCTCTTTCTTCCTCTAGGGTGTCGTTAATTACCCGTAC<br>TAAAGGTTTGGAAAAGAAAAAAGAGGCCGCCTCGTTTCTTTTTCTTCGTCGAAAAAG<br>GCAATAAAAATTTTTATCACGTTTCTTTTTCTTGAAAATTTTTTTTTTTGATTTTTTTCT<br>CTTTCGATGACCTCCCATTGATATTTAAGTTAATAAACGGTCTTCAATTTCTCAAGTT<br>TCAGTTTCATTTTTCTTGTTCTATTACAACTTTTTTTACTTCTTGCTCATTAGAAAGAA<br>AGCATAGCAATCTAATCTAAGTTTTCTAGATGGATTATAAAGATGACGATGACAAAC<br>CTCCAAAAAAGAAGAGAAAGGTCGATAAGAAATACTCAATAGGCTTAGATATCGGC<br>ACAAATAGCGTCGGATGGGCGGTGATCACTGATGAATATAAGGTTCCGTCTAAAAA<br>GTTCAAGGTTCTGGGAAATACAGACCGCCACAGTATCAAAAAAAATCTTATAGGGG<br>CTCTTTTATTTGACAGTGGAGAGACAGCGGAAGCGACTCGTCTCAAACGGACAGCT<br>CGTAGAAGGTATACACGTCGGAAGAATCGTATTTGTTATCTACAGGAGATTTTTTCA<br>AATGAGATGGCGAAAGTAGATGATAGTTTCTTTCATCGACTTGAAGAGTCTTTTTTG<br>GTGGAAGAAGACAAGAAGCATGAACGTCATCCTATTTTTGGAAATATAGTAGATGA<br>AGTTGCTTATCATGAGAAATATCCAACTATCTATCATCTGCGAAAAAAATTGGTAGA<br>TTCTACTTATAAAGCGGATTTGCGCTTAATCTATTTGGCCTTAGCGCATATGATTAA<br>GTTTCGTGGTCATTTTTTGATTGAGGGAGATTTAAATCCTGATAATAGTGATGTGGA<br>CAAACTATTTATCCAGTTGGTACAAACCTACAATCAATTATTTGAAGAAAACCCTATT<br>AACGCAAGTGGAGTAGATGCTAAAGCGATTCTTTCTGCACGATTGAGTAAATCAAG<br>ACGATTAGAAAATCTCATTGCTCAGCTCCCCGGTGAGAAGAAAAATGGCTTATTTG<br>GGAATCTCATTGCTTTGTCATTGGGTTTGACCCCTAATTTTAAATCAAATTTTGATTT<br>GGCAGAAGATGCTAAATTACAGCTTTCAAAAGATACTTACGATGATGATTTAGATAA<br>TTTATTGGCGCAAATTGGAGATCAATATGCTGATTTGTTTTTGGCAGCTAAGAATTT<br>ATCAGATGCTATTTTACTTTCAGATATCCTAAGAGTAAATACTGAAATAACTAAGGCT<br>CCCCTATCAGCTTCAATGATTAAACGCTACGATGAACATCATCAAGACTTGACTCTT<br>TTAAAAGCTTTAGTTCGACAACAACTTCCAGAAAAGTATAAAGAAATCTTTTTTGATC<br>AATCAAAAAACGGATATGCAGGTTATATTGATGGGGGAGCTAGCCAAGAAGAATTT

	TATAAATTTATCAAACCAATTTTAGAAAAAATGGATGGTACTGAGGAATTATTGGTGA
	AACTAAATCGTGAAGATTTGCTGCGCAAGCAACGGACCTTTGACAACGGCTCTATT
	ACCCATCAAATTCACTTGGGTGAGCTGCATGCTATTTTGAGAAGACAAGAAGACTTT
	TATCCATTTTTAAAAGACAATCGTGAGAAGATTGAAAAAATCTTGACTTTTCGAATTC
	CTTATTATGTTGGTCCATTGGCGCGTGGCAATAGTCGTTTTGCATGGATGACTCGG
	AAGTCTGAAGAAACAATTACCCCATGGAATTTTGAAGAAGTTGTCGATAAAGGTGCT
	TCAGCTCAATCATTTATTGAACGCATGACAAACTTTGATAAAAATCTTCCAAATGAAA
	AAGTACTACCAAAACATAGTTTGCTTTATGAGTATTTTACGGTTTATAACGAATTGAC
	AAAGGTCAAATATGTTACTGAAGGAATGCGAAAACCAGCATTTCTTTCAGGTGAACA
	GAAGAAAGCCATTGTTGATTTACTCTTCAAAACAAATCGAAAAGTAACCGTTAAGCA
	ATTAAAAGAAGATTATTTCAAAAAAATAGAATGTTTTGATAGTGTTGAAATTTCAGGA
	GTTGAAGATAGATTTAATGCTTCATTAGGTACCTACCATGATTTGCTAAAAATTATTA
	AAGATAAAGATTTTTTGGATAATGAAGAAAATGAAGATATCTTAGAGGATATTGTTTT
	AACATTGACCTTATTTGAAGATAGGGAGATGATTGAGGAAAGACTTAAAACATATGC
	TCACCTCTTTGATGATAAGGTGATGAAACAGCTTAAACGTCGCCGTTATACTGGTTG
	GGGACGTTTGTCTCGAAAATTGATTAATGGTATTAGGGATAAGCAATCTGGCAAAA
	CAATATTAGATTTTTTGAAATCAGATGGTTTTGCCAATCGCAATTTTATGCAGCTGAT
	CCATGATGATAGTTTGACATTTAAAGAAGACATTCAAAAAGCACAAGTGTCTGGACA
	AGGCGATAGTTTACATGAACATATTGCAAATTTAGCTGGTAGCCCTGCTATTAAAAA
	AGGTATTTTACAGACTGTAAAAGTTGTTGATGAATTGGTCAAAGTAATGGGGCGGC
	ATAAGCCAGAAAATATCGTTATTGAAATGGCACGTGAAAATCAGACAACTCAAAAG
	GGCCAGAAAAATTCGCGAGAGCGTATGAAACGAATCGAAGAAGGTATCAAAGAATT
	AGGAAGTCAGATTCTTAAAGAGCATCCTGTTGAAAATACTCAATTGCAAAATGAAAA
	GCTCTATCTCTATTATCTCCAAAATGGAAGAGACATGTATGTGGACCAAGAATTAGA
	TATTAATCGTTTAAGTGATTATGATGTCGATCACATTGTTCCACAAAGTTTCCTTAAA
	GACGATTCAATAGACAATAAGGTCTTAACGCGTTCTGATAAAAATCGTGGTAAATCG
	GATAACGTTCCAAGTGAAGAAGTAGTCAAAAAGATGAAAAACTATTGGAGACAACTT
	CTAAACGCCAAGTTAATCACTCAACGTAAGTTTGATAATTTAACGAAAGCTGAACGT
	GGAGGTTTGAGTGAACTTGATAAAGCTGGTTTTATCAAACGCCAATTGGTTGAAACT
	CGCCAAATCACTAAGCATGTGGCACAAATTTTGGATAGTCGCATGAATACTAAATAC
	GATGAAAATGATAAACTTATTCGAGAGGTTAAAGTGATTACCTTAAAATCTAAATTAG
	TTTCTGACTTCCGAAAAGATTTCCAATTCTATAAAGTACGTGAGATTAACAATTACCA
	TCATGCCCATGATGCGTATCTAAATGCCGTCGTTGGAACTGCTTTGATTAAGAAATA
	TCCAAAACTTGAATCGGAGTTTGTCTATGGTGATTATAAAGTTTATGATGTTCGTAA
	AATGATTGCTAAGTCTGAGCAAGAAATAGGCAAAGCAACCGCAAAATATTTCTTTTA
	CTCTAATATCATGAACTTCTTCAAAACAGAAATTACACTTGCAAATGGAGAGATTCG
	CAAACGCCCTCTAATCGAAACTAATGGGGAAACTGGAGAAATTGTCTGGGATAAAG
	GGCGAGATTTTGCCACAGTGCGCAAAGTATTGTCCATGCCCCAAGTCAATATTGTC
	AAGAAAACAGAAGTACAGACAGGCGGATTCTCCAAGGAGTCAATTTTACCAAAAAG
	AAATTCGGACAAGCTTATTGCTCGTAAAAAAGACTGGGATCCAAAAAAATATGGTG
	GTTTTGATAGTCCAACGGTAGCTTATTCAGTCCTAGTGGTTGCTAAGGTGGAAAAA
	GGGAAATCGAAGAAGTTAAAATCCGTTAAAGAGTTACTAGGGATCACAATTATGGA
	AAGAAGTTCCTTTGAAAAAAATCCGATTGACTTTTTAGAAGCTAAAGGATATAAGGA
	AGTTAAAAAAGACTTAATCATTAAACTACCTAAATATAGTCTTTTTGAGTTAGAAAAC
	GGTCGTAAACGGATGCTGGCTAGTGCCGGAGAATTACAAAAAGGAAATGAGCTGG
	CTCTGCCAAGCAAATATGTGAATTTTTTATATTTAGCTAGTCATTATGAAAAGTTGAA
	GGGTAGTCCAGAAGATAACGAACAAAAACAATTGTTTGTGGAGCAGCATAAGCATT
	ATTTAGATGAGATTATTGAGCAAATCAGTGAATTTTCTAAGCGTGTTATTTTAGCAGA
	TGCCAATTTAGATAAAGTTCTTAGTGCATATAACAAACATAGAGACAAACCAATACG
	TGAACAAGCAGAAAATATTATTCATTTATTTACGTTGACGAATCTTGGAGCTCCCGC
	TGCTTTTAAATATTTTGATACAACAATTGATCGTAAACGATATACGTCTACAAAAGAA
	GTTTTAGATGCCACTCTTATCCATCAATCCATCACTGGTCTTTATGAAACACGCATT
	GATTTGAGTCAGCTAGGAGGTGACCCTCCAAAAAAGAAGAGAAAGGTCTGAGCGG
	ATCTCTTATGTCTTTACGATTTATAGTTTTCATTATCAAGTATGCCTATATTAGTATAT
	AGCATCTTTAGATGACAGTGTTCGAAGTTTCACGAATAAAAGATAATATTCTACTTTT
	TGCTCCCACCGCGTTTGCTAGCACGAGTGAACACCATCCCTCGCCTGTGAGTTGTA
	CCCATTCCTCTAAACTGTAGACATGGTAGCTTCAGCAGTGTTCGTTATGTACGGCA
	TCCTCCAACAAACAGTCGGTTATAGTTTGTCCTGCTCCTCTGAATCGAGTCCCTCG
	ATATTTCTCATACTAGTTCTAGAGATCTGCCAATTGAACATAACATGGTAGTTACATA
	TACTAGTAATATGGTTCGGCACACATTAAAAGTATAAAAACTATCTGAATTACGAATT
	ACATATATTGGTCATAAAAATCAATCAATCATCGTGTGTTTTATATGTCTCTTATCTA

	AGTATAAGAATATCCATAGTTAATATTCACTTACGCTACCTTTTAACCTGTAATCATT<br>GTCAACAGGATATGTTAACGACCCACATTGATAAACGCTAGTATTTCTTTTTCCTCT<br>TCTTATTGGCCGGCTGTCTCTATACTCCCCTATAGTCTGTTTCTTTTCGTTTCGATT<br>GTTTTACGTTTGAGGCCTCGTGGCGCACATGGTACGCTGTGGTGCTCGCGGCTGG<br>GAACGAAACTCTGGGAGCTGCGATTGGCAGGAACCATTCAAAACAGCATAGCAAG<br>TTAAAATAAGGCTAGTCCGTTATCAACTTGAAAAAGTGGCACCGAGTCGGTGCTTTT<br>TTTCCATATCCAACTTCCAATTTAATCTTTCTTTTTTAATTTTCACTTATTTGCGATAC<br>AGAAAGACCCTGCAGGCATGCAAGCTTGGCGTAATCATGGTCATAGCTGTTTCCTG<br>TGTGAAATTGTTATCCGCTCACAATTCCACACAACATACGAGCCGGAAGCATAAAG<br>TGTAAAGCCTGGGGTGCCTAATGAGTGAGCTAACTCACATTAATTGCGTTGCGCTC<br>ACTGCCCGCTTTCCAGTCGGGAAACCTGTCGTGCCAGCTGCATTAATGAATCGGC<br>CAACGCGCGGGGAGAGGCGGTTTGCGTATTGGGCGCTCTTCCGCTTCCTCGCTCA<br>CTGACTCGCTGCGCTCGGTCGTTCGGCTGCGGCGAGCGGTATCAGCTCACTCAAA<br>GGCGGTAATACGGTTATCCACAGAATCAGGGGATAACGCAGGAAAGAACATGTGA<br>GCAAAAGGCCAGCAAAAGGCCAGGAACCGTAAAAAGGCCGCGTTGCTGGCGTTTT<br>TCCATAGGCTCCGCCCCCCTGACGAGCATCACAAAAATCGACGCTCAAGTCAGAG<br>GTGGCGAAACCCGACAGGACTATAAAGATACCAGGCGTTTCCCCCTGGAAGCTCC<br>CTCGTGCGCTCTCCTGTTCCGACCCTGCCGCTTACCGGATACCTGTCCGCCTTTCT<br>CCCTTCGGGAAGCGTGGCGCTTTCTCATAGCTCACGCTGTAGGTATCTCAGTTCG<br>GTGTAGGTCGTTCGCTCCAAGCTGGGCTGTGTGCACGAACCCCCCGTTCAGCCCG<br>ACCGCTGCGCCTTATCCGGTAACTATCGTCTTGAGTCCAACCCGGTAAGACACGAC<br>TTATCGCCACTGGCAGCAGCCACTGGTAACAGGATTAGCAGAGCGAGGTATGTAG<br>GCGGTGCTACAGAGTTCTTGAAGTGGTGGCCTAACTACGGCTACACTAGAAGAAC<br>AGTATTTGGTATCTGCGCTCTGCTGAAGCCAGTTACCTTCGGAAAAAGAGTTGGTA<br>GCTCTTGATCCGGCAAACAAACCACCGCTGGTAGCGGTGGTTTTTTTGTTTGCAAG<br>CAGCAGATTACGCGCAGAAAAAAAGGATCTCAAGAAGATCCTTTGATCTTTTCTAC<br>GGGGTCTGACGCTCAGTGGAACGAAAACTCACGTTAAGGGATTTTGGTCATGAGAT<br>TATCAAAAAGGATCTTCACCTAGATCCTTTTAAATTAAAAATGAAGTTTTAAATCAAT<br>CTAAAGTATATATGAGTAAACTTGGTCTGACAGTTACCAATGCTTAATCAGTGAGGC<br>ACCTATCTCAGCGATCTGTCTATTTCGTTCATCCATAGTTGCCTGACTCCCCGTCGT<br>GTAGATAACTACGATACGGGAGGGCTTACCATCTGGCCCCAGTGCTGCAATGATA<br>CCGCGGCTCCCACGCTCACCGGCTCCAGATTTATCAGCAATAAACCAGCCAGCCG<br>GAAGGGCCGAGCGCAGAAGTGGTCCTGCAACTTTATCCGCCTCCATCCAGTCTAT<br>TAATTGTTGCCGGGAAGCTAGAGTAAGTAGTTCGCCAGTTAATAGTTTGCGCAACG<br>TTGTTGCCATTGCTACAGGCATCGTGGTGTCACGCTCGTCGTTTGGTATGGCTTCA<br>TTCAGCTCCGGTTCCCAACGATCAAGGCGAGTTACATGATCCCCCATGTTGTGCAA<br>AAAAGCGGTTAGCTCCTTCGGTCCTCCGATCGTTGTCAGAAGTAAGTTGGCCGCA<br>GTGTTATCACTCATGGTTATGGCAGCACTGCATAATTCTCTTACTGTCATGCCATCC<br>GTAAGATGCTTTTCTGTGACTGGTGAGTACTCAACCAAGTCATTCTGAGAATAGTGT<br>ATGCGGCGACCGAGTTGCTCTTGCCCGGCGTCAATACGGGATAATACCGCGCCAC<br>ATAGCAGAACTTTAAAAGTGCTCATCATTGGAAAACGTTCTTCGGGGCGAAAACTC<br>TCAAGGATCTTACCGCTGTTGAGATCCAGTTCGATGTAACCCACTCGTGCACCCAA<br>CTGATCTTCAGCATCTTTTACTTTCACCAGCGTTTCTGGGTGAGCAAAAACAGGAA<br>GGCAAAATGCCGCAAAAAAGGGAATAAGGGCGACACGGAAATGTTGAATACTCATA<br>CTCTTCCTTTTTCAATATTATTGAAGCATTTATCAGGGTTATTGTCTCATGAGCGGAT<br>ACATATTTGAATGTATTTAGAAAAATAAACAAATAGGGGTTCCGCGCACATTTCCCC<br>GAAAAGTGCCACCTGAACGAAGCATCTGTGCTTCATTTTGTAGAACAAAAATGCAA<br>CGCGAGAGCGCTAATTTTTCAAACAAAGAATCTGAGCTGCATTTTTACAGAACAGAA<br>ATGCAACGCGAAAGCGCTATTTTACCAACGAAGAATCTGTGCTTCATTTTTGTAAAA<br>CAAAAATGCAACGCGAGAGCGCTAATTTTTCAAACAAAGAATCTGAGCTGCATTTTT<br>ACAGAACAGAAATGCAACGCGAGAGCGCTATTTTACCAACAAAGAATCTATACTTCT<br>TTTTTGTTCTACAAAAATGCATCCCGAGAGCGCTATTTTTCTAACAAAGCATCTTAG<br>ATTACTTTTTTTCTCCTTTGTGCGCTCTATAATGCAGTCTCTTGATAACTTTTTGCAC<br>TGTAGGTCCGTTAAGGTTAGAAGAAGGCTACTTTGGTGTCTATTTTCTCTTCCATAA<br>AAAAAGCCTGACTCCACTTCCCGCGTTTACTGATTACTAGCGAAGCTGCGGGTGCA<br>TTTTTTCAAGATAAAGGCATCCCCGATTATATTCTATACCGATGTGGATTGCGCATA<br>CTTTGTGAACAGAAAGTGATAGCGTTGATGATTCTTCATTGGTCAGAAAATTATGAA<br>CGGTTTCTTCTATTTTGTCTCTATATACTACGTATAGGAAATGTTTACATTTTCGTAT<br>TGTTTTCGATTCACTCTATGAATAGTTCTTACTACAATTTTTTTGTCTAAAGAGTAATA<br>CTAGAGATAAACATAAAAAATGTAGAGGTCGAGTTTAGATGCAAGTTCAAGGAGCG<br>AAAGGTGGATGGGTAGGTTATATAGGGATATAGCACAGAGATATATAGCAAAGAGA

	TACTTTTGAGCAATGTTTGTGGAAGCGGTATTCGCAATATTTTAGTAGCTCGTTACA<br>GTCCGGTGCGTTTTTGGTTTTTTGAAAGTGCGTCTTCAGAGCGCTTTTGGTTTTCAA<br>AAGCGCTCTGAAGTTCCTATACTTTCTAGAGAATAGGAACTTCGGAATAGGAACTTC<br>AAAGCGTTTCCGAAAACGAGCGCTTCCGAAAATGCAACGCGAGCTGCGCACATAC<br>AGCTCACTGTTCACGTCGCACCTATATCTGCGTGTTGCCTGTATATATATATACATG<br>AGAAGAACGGCATAGTGCGTGTTTATGCTTAAATGCGTACTTATATGCGTCTATTTA<br>TGTAGGATGAAAGGTAGTCTAGTACCTCCTGTGATATTATCCCATTCCATGCGGGG<br>TATCGTATGCTTCCTTCAGCACTACCCTTTAGCTGTTCTATATGCTGCCACTCCTCA<br>ATTGGATTAGTCTCATCCTTCAATGCTATCATTTCCTTTGATATTGGATCATACTAAG<br>AAACCATTATTATCATGACATTAACCTATAAAAATAGGCGTATCACGAGGCCCTTTC<br>GTC
pHV007	TCGCGCGAACCCAACTGCACAGAACAAAAACCTGCAGGAAACGAAGATAAATCATG<br>TCGAAAGCTACATATAAGGAACGTGCTGCTACTCATCCTAGTCCTGTTGCTGCCAA<br>GCTATTTAATATCATGCACGAAAAGCAAACAAACTTGTGTGCTTCATTGGATGTTCG<br>TACCACCAAGGAATTACTGGAGTTAGTTGAAGCATTAGGTCCCAAAATTTGTTTACT<br>AAAAACACATGTGGATATCTTGACTGATTTTTCCATGGAGGGCACAGTTAAGCCGC<br>TAAAGGCATTATCCGCCAAGTACAATTTTTTACTCTTCGAAGACAGAAAATTTGCTG<br>ACATTGGTAATACAGTCAAATTGCAGTACTCTGCGGGTGTATACAGAATAGCAGAA<br>TGGGCAGACATTACGAATGCACACGGTGTGGTGGGCCCAGGTATTGTTAGCGGTT<br>TGAAGCAGGCGGCAGAAGAAGTAACAAAGGAACCTAGAGGCCTTTTGATGTTAGC<br>AGAATTGTCATGCAAGGGCTCCCTATCTACTGGAGAATATACTAAGGGTACTGTTG<br>ACATTGCGAAGAGCGACAAAGATTTTGTTATCGGCTTTATTGCTCAAAGAGACATG<br>GGTGGAAGAGATGAAGGTTACGATTGGTTGATTATGACACCCGGTGTGGGTTTAGA<br>TGACAAGGGAGACGCATTGGGTCAACAGTATAGAACCGTGGATGATGTCGTTTCTA<br>CAGGATCTGACATTATTATTGTTGGAAGAGGACTATTTGCAAAGGGAAGGGATGCT<br>AAGGTAGAGGGTGAACGTTACAGAAAAGCAGGCTGGGAAGCATATTTGAGAAGAT<br>GCGGCCAGCAAAACTAAAAAACTGTATTATAAGTAAATGCATGTATACTAAACTCAC<br>AAATTAGAGCTTCAATTTAATTATATCAGTTATTACCCTGTGCTCGAGTCTTTGAAAA<br>GATAATGTATGATTATGCTTTCACTCATATTTATACAGAAACTTGATGTTTTCTTTCG<br>AGTATATACAAGGTGATTACATGTACGTTTGAAGTACAACTCTAGATTTTGTAGTGC<br>CCTCTTGGGCTAGCGGTAAAGGTGCGCATTTTTTCACACCCTACAATGTTCTGTTC<br>AAAAGATTTTGGTCAAACGCTGTAGAAGTGAAAGTTGGTGCGCATGTTTCGGCGTT<br>CGAAACTTCTCCGCAGTGAAAGATAAATGATCGGGTTTTAgAGCTATGCTGTTTTGA<br>ATGGTCCCAAATGATTGCACGAACAGAGTGAAGAGCTATGCTGTTTTGAATGGTCC<br>CAAAACTTTTTTTGTTTTTTATGTCTGCATAGCTTCAAAATGTTTCTACTCCTTTTTTA<br>CTCTTCCAGATTTTCTCGGACTCCGCGCATCGCCGTACCACTTCAAAACACCCAAG<br>CACAGCATACTAAATTTCCCCTCTTTCTTCCTCTAGGGTGTCGTTAATTACCCGTAC<br>TAAAGGTTTGGAAAAGAAAAAAGAGGCCGCCTCGTTTCTTTTTCTTCGTCGAAAAAG<br>GCAATAAAAATTTTTATCACGTTTCTTTTTCTTGAAAATTTTTTTTTTTGATTTTTTTCT<br>CTTTCGATGACCTCCCATTGATATTTAAGTTAATAAACGGTCTTCAATTTCTCAAGTT<br>TCAGTTTCATTTTTCTTGTTCTATTACAACTTTTTTTACTTCTTGCTCATTAGAAAGAA<br>AGCATAGCAATCTAATCTAAGTTTCTAGATGGATTATAAAGATGACGATGACAAAC<br>CTCCAAAAAAGAAGAGAAAGGTCGATAAGAAATACTCAATAGGCTTAGATATCGGC<br>ACAAATAGCGTCGGATGGGCGGTGATCACTGATGAATATAAGGTTCCGTCTAAAAA<br>GTTCAAGGTTCTGGGAAATACAGACCGCCACAGTATCAAAAAAAATCTTATAGGGG<br>CTCTTTTATTTGACAGTGGAGAGACAGCGGAAGCGACTCGTCTCAAACGGACAGCT<br>CGTAGAAGGTATACACGTCGGAAGAATCGTATTTGTTATCTACAGGAGATTTTTTCA<br>AATGAGATGGCGAAAGTAGATGATAGTTTCTTTCATCGACTTGAAGAGTCTTTTTTG<br>GTGGAAGAAGACAAGAAGCATGAACGTCATCCTATTTTTGGAAATATAGTAGATGA<br>AGTTGCTTATCATGAGAAATATCCAACTATCTATCATCTGCGAAAAAAATTGGTAGA<br>TTCTACTTATAAAGCGGATTTGCGCTTAATCTATTTGGCCTTAGCGCATATGATTAA<br>GTTTCGTGGTCATTTTTTGATTGAGGGAGATTTAAATCCTGATAATAGTGATGTGGA<br>CAAACTATTTATCCAGTTGGTACAAACCTACAATCAATTATTTGAAGAAAACCCTATT<br>AACGCAAGTGGAGTAGATGCTAAAGCGATTCTTTCTGCACGATTGAGTAAATCAAG<br>ACGATTAGAAAATCTCATTGCTCAGCTCCCCGGTGAGAAGAAAAATGGCTTATTTG<br>GGAATCTCATTGCTTTGTCATTGGGTTTGACCCCTAATTTTAAATCAAATTTTGATTT<br>GGCAGAAGATGCTAAATTACAGCTTTCAAAAGATACTTACGATGATGATTTAGATAA<br>TTTATTGGCGCAAATTGGAGATCAATATGCTGATTTGTTTTTGGCAGCTAAGAATTT<br>ATCAGATGCTATTTTACTTTCAGATATCCTAAGAGTAAATACTGAAATAACTAAGGCT<br>CCCCTATCAGCTTCAATGATTAAACGCTACGATGAACATCATCAAGACTTGACTCTT<br>TTAAAAGCTTTAGTTCGACAACAACTTCCAGAAAAGTATAAAGAAATCTTTTTTGATC

	AATCAAAAAACGGATATGCAGGTTATATTGATGGGGGAGCTAGCCAAGAAGAATTT
	TATAAATTTATCAAACCAATTTTAGAAAAAATGGATGGTACTGAGGAATTATTGGTGA
	AACTAAATCGTGAAGATTTGCTGCGCAAGCAACGGACCTTTGACAACGGCTCTATT
	ACCCATCAAATTCACTTGGGTGAGCTGCATGCTATTTTGAGAAGACAAGAAGACTTT
	TATCCATTTTTAAAAGACAATCGTGAGAAGATTGAAAAAATCTTGACTTTTCGAATTC
	CTTATTATGTTGGTCCATTGGCGCGTGGCAATAGTCGTTTTGCATGGATGACTCGG
	AAGTCTGAAGAAACAATTACCCCATGGAATTTTGAAGAAGTTGTCGATAAAGGTGCT
	TCAGCTCAATCATTTATTGAACGCATGACAAACTTTGATAAAAATCTTCCAAATGAAA
	AAGTACTACCAAAACATAGTTTGCTTTATGAGTATTTTACGGTTTATAACGAATTGAC
	AAAGGTCAAATATGTTACTGAAGGAATGCGAAAACCAGCATTTCTTTCAGGTGAACA
	GAAGAAAGCCATTGTTGATTTACTCTTCAAAACAAATCGAAAAGTAACCGTTAAGCA
	ATTAAAAGAAGATTATTTCAAAAAAATAGAATGTTTTGATAGTGTTGAAATTTCAGGA
	GTTGAAGATAGATTTAATGCTTCATTAGGTACCTACCATGATTTGCTAAAAATTATTA
	AAGATAAAGATTTTTTGGATAATGAAGAAAATGAAGATATCTTAGAGGATATTGTTTT
	AACATTGACCTTATTTGAAGATAGGGAGATGATTGAGGAAAGACTTAAAACATATGC
	TCACCTCTTTGATGATAAGGTGATGAAACAGCTTAAACGTCGCCGTTATACTGGTTG
	GGGACGTTTGTCTCGAAAATTGATTAATGGTATTAGGGATAAGCAATCTGGCAAAA
	CAATATTAGATTTTTTGAAATCAGATGGTTTTGCCAATCGCAATTTTATGCAGCTGAT
	CCATGATGATAGTTTGACATTTAAAGAAGACATTCAAAAAGCACAAGTGTCTGGACA
	AGGCGATAGTTTACATGAACATATTGCAAATTTAGCTGGTAGCCCTGCTATTAAAAA
	AGGTATTTTACAGACTGTAAAAGTTGTTGATGAATTGGTCAAAGTAATGGGGCGGC
	ATAAGCCAGAAAATATCGTTATTGAAATGGCACGTGAAAATCAGACAACTCAAAAG
	GGCCAGAAAAATTCGCGAGAGCGTATGAAACGAATCGAAGAAGGTATCAAAGAATT
	AGGAAGTCAGATTCTTAAAGAGCATCCTGTTGAAAATACTCAATTGCAAAATGAAAA
	GCTCTATCTCTATTATCTCCAAAATGGAAGAGACATGTATGTGGACCAAGAATTAGA
	TATTAATCGTTTAAGTGATTATGATGTCGATCACATTGTTCCACAAAGTTTCCTTAAA
	GACGATTCAATAGACAATAAGGTCTTAACGCGTTCTGATAAAAATCGTGGTAAATCG
	GATAACGTTCCAAGTGAAGAAGTAGTCAAAAAGATGAAAAACTATTGGAGACAACTT
	CTAAACGCCAAGTTAATCACTCAACGTAAGTTTGATAATTTAACGAAAGCTGAACGT
	GGAGGTTTGAGTGAACTTGATAAAGCTGGTTTTATCAAACGCCAATTGGTTGAAACT
	CGCCAAATCACTAAGCATGTGGCACAAATTTTGGATAGTCGCATGAATACTAAATAC
	GATGAAAATGATAAACTTATTCGAGAGGTTAAAGTGATTACCTTAAAATCTAAATTAG
	TTTCTGACTTCCGAAAAGATTTCCAATTCTATAAAGTACGTGAGATTAACAATTACCA
	TCATGCCCATGATGCGTATCTAAATGCCGTCGTTGGAACTGCTTTGATTAAGAAATA
	TCCAAAACTTGAATCGGAGTTTGTCTATGGTGATTATAAAGTTTATGATGTTCGTAA
	AATGATTGCTAAGTCTGAGCAAGAAATAGGCAAAGCAACCGCAAAATATTTCTTTTA
	CTCTAATATCATGAACTTCTTCAAAACAGAAATTACACTTGCAAATGGAGAGATTCG
	CAAACGCCCTCTAATCGAAACTAATGGGGAAACTGGAGAAATTGTCTGGGATAAAG
	GGCGAGATTTTGCCACAGTGCGCAAAGTATTGTCCATGCCCCAAGTCAATATTGTC
	AAGAAAACAGAAGTACAGACAGGCGGATTCTCCAAGGAGTCAATTTTACCAAAAAG
	AAATTCGGACAAGCTTATTGCTCGTAAAAAAGACTGGGATCCAAAAAAATATGGTG
	GTTTTGATAGTCCAACGGTAGCTTATTCAGTCCTAGTGGTTGCTAAGGTGGAAAAA
	GGGAAATCGAAGAAGTTAAAATCCGTTAAAGAGTTACTAGGGATCACAATTATGGA
	AAGAAGTTCCTTTGAAAAAAATCCGATTGACTTTTTAGAAGCTAAAGGATATAAGGA
	AGTTAAAAAAGACTTAATCATTAAACTACCTAAATATAGTCTTTTTGAGTTAGAAAAC
	GGTCGTAAACGGATGCTGGCTAGTGCCGGAGAATTACAAAAAGGAAATGAGCTGG
	CTCTGCCAAGCAAATATGTGAATTTTTTATATTTAGCTAGTCATTATGAAAAGTTGAA
	GGGTAGTCCAGAAGATAACGAACAAAAACAATTGTTTGTGGAGCAGCATAAGCATT
	ATTTAGATGAGATTATTGAGCAAATCAGTGAATTTTCTAAGCGTGTTATTTTAGCAGA
	TGCCAATTTAGATAAAGTTCTTAGTGCATATAACAAACATAGAGACAAACCAATACG
	TGAACAAGCAGAAAATATTATTCATTTATTTACGTTGACGAATCTTGGAGCTCCCGC
	TGCTTTTAAATATTTTGATACAACAATTGATCGTAAACGATATACGTCTACAAAAGAA
	GTTTTAGATGCCACTCTTATCCATCAATCCATCACTGGTCTTTATGAAACACGCATT
	GATTTGAGTCAGCTAGGAGGTGACCCTCCAAAAAAGAAGAGAAAGGTCTGAGCGG
	ATCTCTTATGTCTTTACGATTTATAGTTTTCATTATCAAGTATGCCTATATTAGTATAT
	AGCATCTTTAGATGACAGTGTTCGAAGTTTCACGAATAAAAGATAATATTCTACTTTT
	TGCTCCCACCGCGTTTGCTAGCACGAGTGAACACCATCCCTCGCCTGTGAGTTGTA
	CCCATTCCTCTAAACTGTAGACATGGTAGCTTCAGCAGTGTTCGTTATGTACGGCA
	TCCTCCAACAAACAGTCGGTTATAGTTTGTCCTGCTCCTCTGAATCGAGTCCCTCG
	ATATTTCTCATACTAGTTCTAGAGATCTGCCAATTGAACATAACATGGTAGTTACATA
	TACTAGTAATATGGTTCGGCACACATTAAAAGTATAAAAACTATCTGAATTACGAATT

	ACATATATTGGTCATAAAAATCAATCAATCATCGTGTGTTTTATATGTCTCTTATCTA<br>AGTATAAGAATATCCATAGTTAATATTCACTTACGCTACCTTTTAACCTGTAATCATT<br>GTCAACAGGATATGTTAACGACCCACATTGATAAACGCTAGTATTTCTTTTTCCTCT<br>TCTTATTGGCCGGCTGTCTCTATACTCCCCTATAGTCTGTTTCTTTTCGTTTCGATT<br>GTTTTACGTTTGAGGCCTCGTGGCGCACATGGTACGCTGTGGTGCTCGCGGCTGG<br>GAACGAAACTCTGGGAGCTGCGATTGGCAGGAACCATTCAAAACAGCATAGCAAG<br>TTAAAATAAGGCTAGTCCGTTATCAACTTGAAAAAGTGGCACCGAGTCGGTGCTTTT<br>TTTCCATATCCAACTTCCAATTTAATCTTTCTTTTTTAATTTTCACTTATTTGCGATAC<br>AGAAAGACCCTGCAGGCATGCAAGCTTGGCGTAATCATGGTCATAGCTGTTTCCTG<br>TGTGAAATTGTTATCCGCTCACAATTCCACACAACATACGAGCCGGAAGCATAAAG<br>TGTAAAGCCTGGGGTGCCTAATGAGTGAGCTAACTCACATTAATTGCGTTGCGCTC<br>ACTGCCCGCTTTCCAGTCGGGAAACCTGTCGTGCCAGCTGCATTAATGAATCGGC<br>CAACGCGCGGGGAGAGGCGGTTTGCGTATTGGGCGCTCTTCCGCTTCCTCGCTCA<br>CTGACTCGCTGCGCTCGGTCGTTCGGCTGCGGCGAGCGGTATCAGCTCACTCAAA<br>GGCGGTAATACGGTTATCCACAGAATCAGGGGATAACGCAGGAAAGAACATGTGA<br>GCAAAAGGCCAGCAAAAGGCCAGGAACCGTAAAAAGGCCGCGTTGCTGGCGTTTT<br>TCCATAGGCTCCGCCCCCCTGACGAGCATCACAAAAATCGACGCTCAAGTCAGAG<br>GTGGCGAAACCCGACAGGACTATAAAGATACCAGGCGTTTCCCCCTGGAAGCTCC<br>CTCGTGCGCTCTCCTGTTCCGACCCTGCCGCTTACCGGATACCTGTCCGCCTTTCT<br>CCCTTCGGGAAGCGTGGCGCTTTCTCATAGCTCACGCTGTAGGTATCTCAGTTCG<br>GTGTAGGTCGTTCGCTCCAAGCTGGGCTGTGTGCACGAACCCCCCGTTCAGCCCG<br>ACCGCTGCGCCTTATCCGGTAACTATCGTCTTGAGTCCAACCCGGTAAGACACGAC<br>TTATCGCCACTGGCAGCAGCCACTGGTAACAGGATTAGCAGAGCGAGGTATGTAG<br>GCGGTGCTACAGAGTTCTTGAAGTGGTGGCCTAACTACGGCTACACTAGAAGAAC<br>AGTATTTGGTATCTGCGCTCTGCTGAAGCCAGTTACCTTCGGAAAAAGAGTTGGTA<br>GCTCTTGATCCGGCAAACAAACCACCGCTGGTAGCGGTGGTTTTTTTGTTTGCAAG<br>CAGCAGATTACGCGCAGAAAAAAAGGATCTCAAGAAGATCCTTTGATCTTTTCTAC<br>GGGGTCTGACGCTCAGTGGAACGAAAACTCACGTTAAGGGATTTTGGTCATGAGAT<br>TATCAAAAAGGATCTTCACCTAGATCCTTTTAAATTAAAAATGAAGTTTTAAATCAAT<br>CTAAAGTATATATGAGTAAACTTGGTCTGACAGTTACCAATGCTTAATCAGTGAGGC<br>ACCTATCTCAGCGATCTGTCTATTTCGTTCATCCATAGTTGCCTGACTCCCCGTCGT<br>GTAGATAACTACGATACGGGAGGGCTTACCATCTGGCCCCAGTGCTGCAATGATA<br>CCGCGGCTCCCACGCTCACCGGCTCCAGATTTATCAGCAATAAACCAGCCAGCCG<br>GAAGGGCCGAGCGCAGAAGTGGTCCTGCAACTTTATCCGCCTCCATCCAGTCTAT<br>TAATTGTTGCCGGGAAGCTAGAGTAAGTAGTTCGCCAGTTAATAGTTTGCGCAACG<br>TTGTTGCCATTGCTACAGGCATCGTGGTGTCACGCTCGTCGTTTGGTATGGCTTCA<br>TTCAGCTCCGGTTCCCAACGATCAAGGCGAGTTACATGATCCCCCATGTTGTGCAA<br>AAAAGCGGTTAGCTCCTTCGGTCCTCCGATCGTTGTCAGAAGTAAGTTGGCCGCA<br>GTGTTATCACTCATGGTTATGGCAGCACTGCATAATTCTCTTACTGTCATGCCATCC<br>GTAAGATGCTTTTCTGTGACTGGTGAGTACTCAACCAAGTCATTCTGAGAATAGTGT<br>ATGCGGCGACCGAGTTGCTCTTGCCCGGCGTCAATACGGGATAATACCGCGCCAC<br>ATAGCAGAACTTTAAAAGTGCTCATCATTGGAAAACGTTCTTCGGGGCGAAAACTC<br>TCAAGGATCTTACCGCTGTTGAGATCCAGTTCGATGTAACCCACTCGTGCACCCAA<br>CTGATCTTCAGCATCTTTTACTTTCACCAGCGTTTCTGGGTGAGCAAAAACAGGAA<br>GGCAAAATGCCGCAAAAAAGGGAATAAGGGCGACACGGAAATGTTGAATACTCATA<br>CTCTTCCTTTTTCAATATTATTGAAGCATTTATCAGGGTTATTGTCTCATGAGCGGAT<br>ACATATTTGAATGTATTTAGAAAAATAAACAAATAGGGGTTCCGCGCACATTTCCCC<br>GAAAAGTGCCACCTGAACGAAGCATCTGTGCTTCATTTTGTAGAACAAAAATGCAA<br>CGCGAGAGCGCTAATTTTTCAAACAAAGAATCTGAGCTGCATTTTTACAGAACAGAA<br>ATGCAACGCGAAAGCGCTATTTTACCAACGAAGAATCTGTGCTTCATTTTTGTAAAA<br>CAAAAATGCAACGCGAGAGCGCTAATTTTTCAAACAAAGAATCTGAGCTGCATTTTT<br>ACAGAACAGAAATGCAACGCGAGAGCGCTATTTTACCAACAAAGAATCTATACTTCT<br>TTTTTGTTCTACAAAAATGCATCCCGAGAGCGCTATTTTTCTAACAAAGCATCTTAG<br>ATTACTTTTTTTCTCCTTTGTGCGCTCTATAATGCAGTCTCTTGATAACTTTTTGCAC<br>TGTAGGTCCGTTAAGGTTAGAAGAAGGCTACTTTGGTGTCTATTTTCTCTTCCATAA<br>AAAAAGCCTGACTCCACTTCCCGCGTTTACTGATTACTAGCGAAGCTGCGGGTGCA<br>TTTTTTCAAGATAAAGGCATCCCCGATTATATTCTATACCGATGTGGATTGCGCATA<br>CTTTGTGAACAGAAAGTGATAGCGTTGATGATTCTTCATTGGTCAGAAAATTATGAA<br>CGGTTTCTTCTATTTTGTCTCTATATACTACGTATAGGAAATGTTTACATTTTCGTAT<br>TGTTTTCGATTCACTCTATGAATAGTTCTTACTACAATTTTTTTGTCTAAAGAGTAATA<br>CTAGAGATAAACATAAAAAATGTAGAGGTCGAGTTTAGATGCAAGTTCAAGGAGCG

	AAAGGTGGATGGGTAGGTTATATAGGGATATAGCACAGAGATATATAGCAAAGAGA<br>TACTTTTGAGCAATGTTTGTGGAAGCGGTATTCGCAATATTTTAGTAGCTCGTTACA<br>GTCCGGTGCGTTTTTGGTTTTTTGAAAGTGCGTCTTCAGAGCGCTTTTGGTTTTCAA<br>AAGCGCTCTGAAGTTCCTATACTTTCTAGAGAATAGGAACTTCGGAATAGGAACTTC<br>AAAGCGTTTCCGAAAACGAGCGCTTCCGAAAATGCAACGCGAGCTGCGCACATAC<br>AGCTCACTGTTCACGTCGCACCTATATCTGCGTGTTGCCTGTATATATATATATACATG<br>AGAAGAACGGCATAGTGCGTGTTTATGCTTAAATGCGTACTTATATGCGTCTATTTA<br>TGTAGGATGAAAGGTAGTCTAGTACCTCCTGTGATATTATCCCATTCCATGCGGGG<br>TATCGTATGCTTCCTTCAGCACTACCCTTTAGCTGTTCTATATGCTGCCACTCCTCA<br>ATTGGATTAGTCTCATCCTTCAATGCTATCATTTCCTTTGATATTGGATCATACTAAG<br>AAACCATTATTATCATGACATTAACCTATAAAAATAGGCGTATCACGAGGCCCTTTC<br>GTC
pHV008	TCGCGCGAACCCAACTGCACAGAACAAAAACCTGCAGGAAACGAAGATAAATCATG<br>TCGAAAGCTACATATAAGGAACGTGCTGCTACTCATCCTAGTCCTGTTGCTGCCAA<br>GCTATTTAATATCATGCACGAAAAGCAAACAAACTTGTGTGCTTCATTGGATGTTCG<br>TACCACCAAGGAATTACTGGAGTTAGTTGAAGCATTAGGTCCCAAAATTTGTTTACT<br>AAAAACACATGTGGATATCTTGACTGATTTTTCCATGGAGGGCACAGTTAAGCCGC<br>TAAAGGCATTATCCGCCAAGTACAATTTTTTACTCTTCGAAGACAGAAAATTTGCTG<br>ACATTGGTAATACAGTCAAATTGCAGTACTCTGCGGGTGTATACAGAATAGCAGAA<br>TGGGCAGACATTACGAATGCACACGGTGTGGTGGGCCCAGGTATTGTTAGCGGTT<br>TGAAGCAGGCGGCAGAAGAAGTAACAAAGGAACCTAGAGGCCTTTTGATGTTAGC<br>AGAATTGTCATGCAAGGGCTCCCTATCTACTGGAGAATATACTAAGGGTACTGTTG<br>ACATTGCGAAGAGCGACAAAGATTTTGTTATCGGCTTTATTGCTCAAAGAGACATG<br>GGTGGAAGAGATGAAGGTTACGATTGGTTGATTATGACACCCGGTGTGGGTTTAGA<br>TGACAAGGGAGACGCATTGGGTCAACAGTATAGAACCGTGGATGATGTCGTTTCTA<br>CAGGATCTGACATTATTATTGTTGGAAGAGGACTATTTGCAAAGGGAAGGGATGCT<br>AAGGTAGAGGGTGAACGTTACAGAAAAGCAGGCTGGGAAGCATATTTGAGAAGAT<br>GCGGCCAGCAAAACTAAAAAACTGTATTATAAGTAAATGCATGTATACTAAACTCAC<br>AAATTAGAGCTTCAATTTAATTATATCAGTTATTACCCTGTGCTCGAGTCTTTGAAAA<br>GATAATGTATGATTATGCTTTCACTCATATTTATACAGAAACTTGATGTTTTCTTTCG<br>AGTATATACAAGGTGATTACATGTACGTTTGAAGTACAACTCTAGATTTTGTAGTGC<br>CCTCTTGGGCTAGCGGTAAAGGTGCGCATTTTTTCACACCCTACAATGTTCTGTTC<br>AAAAGATTTTGGTCAAACGCTGTAGAAGTGAAAGTTGGTGCGCATGTTTCGGCGTT<br>CGAAACTTCTCCGCAGTGAAAGATAAATGATCGGGTTTTAgAGCTATGCTGTTTTGA<br>ATGGTCCCAAATACGAGCCTTGTGAAAAGCAAGAGCTATGCTGTTTTGAATGGTCC<br>CAAAACTTTTTTTGTTTTTTATGTCTGCATAGCTTCAAAATGTTTCTACTCCTTTTTTA<br>CTCTTCCAGATTTTCTCGGACTCCGCGCATCGCCGTACCACTTCAAAACACCCAAG<br>CACAGCATACTAAATTTCCCCTCTTTCTTCCTCTAGGGTGTCGTTAATTACCCGTAC<br>TAAAGGTTTGGAAAAGAAAAAAGAGGCCGCCTCGTTTCTTTTTCTTCGTCGAAAAAG<br>GCAATAAAAATTTTTATCACGTTTCTTTTTCTTGAAAATTTTTTTTTTTGATTTTTTTCT<br>CTTTCGATGACCTCCCATTGATATTTAAGTTAATAAACGGTCTTCAATTTCTCAAGTT<br>TCAGTTTCATTTTTCTTGTTCTATTACAACTTTTTTTACTTCTTGCTCATTAGAAAGAA<br>AGCATAGCAATCTAATCTAAGTTTCTAGATGGATTATAAAGATGACGATGACAAAC<br>CTCCAAAAAAGAAGAGAAAGGTCGATAAGAAATACTCAATAGGCTTAGATATCGGC<br>ACAAATAGCGTCGGATGGGCGGTGATCACTGATGAATATAAGGTTCCGTCTAAAAA<br>GTTCAAGGTTCTGGGAAATACAGACCGCCACAGTATCAAAAAAAATCTTATAGGGG<br>CTCTTTTATTTGACAGTGGAGAGACAGCGGAAGCGACTCGTCTCAAACGGACAGCT<br>CGTAGAAGGTATACACGTCGGAAGAATCGTATTTGTTATCTACAGGAGATTTTTTCA<br>AATGAGATGGCGAAAGTAGATGATAGTTTCTTTCATCGACTTGAAGAGTCTTTTTTG<br>GTGGAAGAAGACAAGAAGCATGAACGTCATCCTATTTTTGGAAATATAGTAGATGA<br>AGTTGCTTATCATGAGAAATATCCAACTATCTATCATCTGCGAAAAAAATTGGTAGA<br>TTCTACTTATAAAGCGGATTTGCGCTTAATCTATTTGGCCTTAGCGCATATGATTAA<br>GTTTCGTGGTCATTTTTTGATTGAGGGAGATTTAAATCCTGATAATAGTGATGTGGA<br>CAAACTATTTATCCAGTTGGTACAAACCTACAATCAATTATTTGAAGAAAACCCTATT<br>AACGCAAGTGGAGTAGATGCTAAAGCGATTCTTTCTGCACGATTGAGTAAATCAAG<br>ACGATTAGAAAATCTCATTGCTCAGCTCCCCGGTGAGAAGAAAAATGGCTTATTTG<br>GGAATCTCATTGCTTTGTCATTGGGTTTGACCCCTAATTTTAAATCAAATTTTGATTT<br>GGCAGAAGATGCTAAATTACAGCTTTCAAAAGATACTTACGATGATGATTTAGATAA<br>TTTATTGGCGCAAATTGGAGATCAATATGCTGATTTGTTTTTGGCAGCTAAGAATTT<br>ATCAGATGCTATTTTACTTTCAGATATCCTAAGAGTAAATACTGAAATAACTAAGGCT<br>CCCCTATCAGCTTCAATGATTAAACGCTACGATGAACATCATCAAGACTTGACTCTT

	TTAAAAGCTTTAGTTCGACAACAACTTCCAGAAAAGTATAAAGAAATCTTTTTTGATC<br>AATCAAAAAACGGATATGCAGGTTATATTGATGGGGGAGCTAGCCAAGAAGAATTT<br>TATAAATTTATCAAACCAATTTTAGAAAAAATGGATGGTACTGAGGAATTATTGGTGA<br>AACTAAATCGTGAAGATTTGCTGCGCAAGCAACGGACCTTTGACAACGGCTCTATT<br>ACCCATCAAATTCACTTGGGTGAGCTGCATGCTATTTTGAGAAGACAAGAAGACTTT<br>TATCCATTTTTAAAAGACAATCGTGAGAAGATTGAAAAAATCTTGACTTTTCGAATTC<br>CTTATTATGTTGGTCCATTGGCGCGTGGCAATAGTCGTTTTGCATGGATGACTCGG<br>AAGTCTGAAGAAACAATTACCCCATGGAATTTTGAAGAAGTTGTCGATAAAGGTGCT<br>TCAGCTCAATCATTTATTGAACGCATGACAAACTTTGATAAAAATCTTCCAAATGAAA<br>AAGTACTACCAAAACATAGTTTGCTTTATGAGTATTTTACGGTTTATAACGAATTGAC<br>AAAGGTCAAATATGTTACTGAAGGAATGCGAAAACCAGCATTTCTTTCAGGTGAACA<br>GAAGAAAGCCATTGTTGATTTACTCTTCAAAACAAATCGAAAAGTAACCGTTAAGCA<br>ATTAAAAGAAGATTATTTCAAAAAAATAGAATGTTTTGATAGTGTTGAAATTTCAGGA<br>GTTGAAGATAGATTTAATGCTTCATTAGGTACCTACCATGATTTGCTAAAAATTATTA<br>AAGATAAAGATTTTTTGGATAATGAAGAAAATGAAGATATCTTAGAGGATATTGTTTT<br>AACATTGACCTTATTTGAAGATAGGGAGATGATTGAGGAAAGACTTAAAACATATGC<br>TCACCTCTTTGATGATAAGGTGATGAAACAGCTTAAACGTCGCCGTTATACTGGTTG<br>GGGACGTTTGTCTCGAAAATTGATTAATGGTATTAGGGATAAGCAATCTGGCAAAA<br>CAATATTAGATTTTTTGAAATCAGATGGTTTTGCCAATCGCAATTTTATGCAGCTGAT<br>CCATGATGATAGTTTGACATTTAAAGAAGACATTCAAAAAGCACAAGTGTCTGGACA<br>AGGCGATAGTTTACATGAACATATTGCAAATTTAGCTGGTAGCCCTGCTATTAAAAA<br>AGGTATTTTACAGACTGTAAAAGTTGTTGATGAATTGGTCAAAGTAATGGGGCGGC<br>ATAAGCCAGAAAATATCGTTATTGAAATGGCACGTGAAAATCAGACAACTCAAAAG<br>GGCCAGAAAAATTCGCGAGAGCGTATGAAACGAATCGAAGAAGGTATCAAAGAATT<br>AGGAAGTCAGATTCTTAAAGAGCATCCTGTTGAAAATACTCAATTGCAAAATGAAAA<br>GCTCTATCTCTATTATCTCCAAAATGGAAGAGACATGTATGTGGACCAAGAATTAGA<br>TATTAATCGTTTAAGTGATTATGATGTCGATCACATTGTTCCACAAAGTTTCCTTAAA<br>GACGATTCAATAGACAATAAGGTCTTAACGCGTTCTGATAAAAATCGTGGTAAATCG<br>GATAACGTTCCAAGTGAAGAAGTAGTCAAAAAGATGAAAAACTATTGGAGACAACTT<br>CTAAACGCCAAGTTAATCACTCAACGTAAGTTTGATAATTTAACGAAAGCTGAACGT<br>GGAGGTTTGAGTGAACTTGATAAAGCTGGTTTTATCAAACGCCAATTGGTTGAAACT<br>CGCCAAATCACTAAGCATGTGGCACAAATTTTGGATAGTCGCATGAATACTAAATAC<br>GATGAAAATGATAAACTTATTCGAGAGGTTAAAGTGATTACCTTAAAATCTAAATTAG<br>TTTCTGACTTCCGAAAAGATTTCCAATTCTATAAAGTACGTGAGATTAACAATTACCA<br>TCATGCCCATGATGCGTATCTAAATGCCGTCGTTGGAACTGCTTTGATTAAGAAATA<br>TCCAAAACTTGAATCGGAGTTTGTCTATGGTGATTATAAAGTTTATGATGTTCGTAA<br>AATGATTGCTAAGTCTGAGCAAGAAATAGGCAAAGCAACCGCAAAATATTTCTTTTA<br>CTCTAATATCATGAACTTCTTCAAAACAGAAATTACACTTGCAAATGGAGAGATTCG<br>CAAACGCCCTCTAATCGAAACTAATGGGGAAACTGGAGAAATTGTCTGGGATAAAG<br>GGCGAGATTTTGCCACAGTGCGCAAAGTATTGTCCATGCCCCAAGTCAATATTGTC<br>AAGAAAACAGAAGTACAGACAGGCGGATTCTCCAAGGAGTCAATTTTACCAAAAAG<br>AAATTCGGACAAGCTTATTGCTCGTAAAAAAGACTGGGATCCAAAAAAATATGGTG<br>GTTTTGATAGTCCAACGGTAGCTTATTCAGTCCTAGTGGTTGCTAAGGTGGAAAAA<br>GGGAAATCGAAGAAGTTAAAATCCGTTAAAGAGTTACTAGGGATCACAATTATGGA<br>AAGAAGTTCCTTTGAAAAAAATCCGATTGACTTTTTAGAAGCTAAAGGATATAAGGA<br>AGTTAAAAAAGACTTAATCATTAAACTACCTAAATATAGTCTTTTTGAGTTAGAAAAC<br>GGTCGTAAACGGATGCTGGCTAGTGCCGGAGAATTACAAAAAGGAAATGAGCTGG<br>CTCTGCCAAGCAAATATGTGAATTTTTTATATTTAGCTAGTCATTATGAAAAGTTGAA<br>GGGTAGTCCAGAAGATAACGAACAAAAACAATTGTTTGTGGAGCAGCATAAGCATT<br>ATTTAGATGAGATTATTGAGCAAATCAGTGAATTTTCTAAGCGTGTTATTTTAGCAGA<br>TGCCAATTTAGATAAAGTTCTTAGTGCATATAACAAACATAGAGACAAACCAATACG<br>TGAACAAGCAGAAAATATTATTCATTTATTTACGTTGACGAATCTTGGAGCTCCCGC<br>TGCTTTTAAATATTTTGATACAACAATTGATCGTAAACGATATACGTCTACAAAAGAA<br>GTTTTAGATGCCACTCTTATCCATCAATCCATCACTGGTCTTTATGAAACACGCATT<br>GATTTGAGTCAGCTAGGAGGTGACCCTCCAAAAAAGAAGAGAAAGGTCTGAGCGG<br>ATCTCTTATGTCTTTACGATTTATAGTTTTCATTATCAAGTATGCCTATATTAGTATAT<br>AGCATCTTTAGATGACAGTGTTCGAAGTTTCACGAATAAAAGATAATATTCTACTTTT<br>TGCTCCCACCGCGTTTGCTAGCACGAGTGAACACCATCCCTCGCCTGTGAGTTGTA<br>CCCATTCCTCTAAACTGTAGACATGGTAGCTTCAGCAGTGTTCGTTATGTACGGCA<br>TCCTCCAACAAACAGTCGGTTATAGTTTGTCCTGCTCCTCTGAATCGAGTCCCTCG<br>ATATTTCTCATACTAGTTCTAGAGATCTGCCAATTGAACATAACATGGTAGTTACATA

	TACTAGTAATATGGTTCGGCACACATTAAAAGTATAAAAACTATCTGAATTACGAATT<br>ACATATATTGGTCATAAAAATCAATCAATCATCGTGTGTTTTATATGTCTCTTATCTA<br>AGTATAAGAATATCCATAGTTAATATTCACTTACGCTACCTTTTAACCTGTAATCATT<br>GTCAACAGGATATGTTAACGACCCACATTGATAAACGCTAGTATTTCTTTTTCCTCT<br>TCTTATTGGCCGGCTGTCTCTATACTCCCCTATAGTCTGTTTCTTTTCGTTTCGATT<br>GTTTTACGTTTGAGGCCTCGTGGCGCACATGGTACGCTGTGGTGCTCGCGGCTGG<br>GAACGAAACTCTGGGAGCTGCGATTGGCAGGAACCATTCAAAACAGCATAGCAAG<br>TTAAAATAAGGCTAGTCCGTTATCAACTTGAAAAAGTGGCACCGAGTCGGTGCTTTT<br>TTTCCATATCCAACTTCCAATTTAATCTTTCTTTTTTAATTTTCACTTATTTGCGATAC<br>AGAAAGACCCTGCAGGCATGCAAGCTTGGCGTAATCATGGTCATAGCTGTTTCCTG<br>TGTGAAATTGTTATCCGCTCACAATTCCACACAACATACGAGCCGGAAGCATAAAG<br>TGTAAAGCCTGGGGTGCCTAATGAGTGAGCTAACTCACATTAATTGCGTTGCGCTC<br>ACTGCCCGCTTTCCAGTCGGGAAACCTGTCGTGCCAGCTGCATTAATGAATCGGC<br>CAACGCGCGGGGAGAGGCGGTTTGCGTATTGGGCGCTCTTCCGCTTCCTCGCTCA<br>CTGACTCGCTGCGCTCGGTCGTTCGGCTGCGGCGAGCGGTATCAGCTCACTCAAA<br>GGCGGTAATACGGTTATCCACAGAATCAGGGGATAACGCAGGAAAGAACATGTGA<br>GCAAAAGGCCAGCAAAAGGCCAGGAACCGTAAAAAGGCCGCGTTGCTGGCGTTTT<br>TCCATAGGCTCCGCCCCCCTGACGAGCATCACAAAAATCGACGCTCAAGTCAGAG<br>GTGGCGAAACCCGACAGGACTATAAAGATACCAGGCGTTTCCCCCTGGAAGCTCC<br>CTCGTGCGCTCTCCTGTTCCGACCCTGCCGCTTACCGGATACCTGTCCGCCTTTCT<br>CCCTTCGGGAAGCGTGGCGCTTTCTCATAGCTCACGCTGTAGGTATCTCAGTTCG<br>GTGTAGGTCGTTCGCTCCAAGCTGGGCTGTGTGCACGAACCCCCCGTTCAGCCCG<br>ACCGCTGCGCCTTATCCGGTAACTATCGTCTTGAGTCCAACCCGGTAAGACACGAC<br>TTATCGCCACTGGCAGCAGCCACTGGTAACAGGATTAGCAGAGCGAGGTATGTAG<br>GCGGTGCTACAGAGTTCTTGAAGTGGTGGCCTAACTACGGCTACACTAGAAGAAC<br>AGTATTTGGTATCTGCGCTCTGCTGAAGCCAGTTACCTTCGGAAAAAGAGTTGGTA<br>GCTCTTGATCCGGCAAACAAACCACCGCTGGTAGCGGTGGTTTTTTTGTTTGCAAG<br>CAGCAGATTACGCGCAGAAAAAAAGGATCTCAAGAAGATCCTTTGATCTTTTCTAC<br>GGGGTCTGACGCTCAGTGGAACGAAAACTCACGTTAAGGGATTTTGGTCATGAGAT<br>TATCAAAAAGGATCTTCACCTAGATCCTTTTAAATTAAAAATGAAGTTTTAAATCAAT<br>CTAAAGTATATATGAGTAAACTTGGTCTGACAGTTACCAATGCTTAATCAGTGAGGC<br>ACCTATCTCAGCGATCTGTCTATTTCGTTCATCCATAGTTGCCTGACTCCCCGTCGT<br>GTAGATAACTACGATACGGGAGGGCTTACCATCTGGCCCCAGTGCTGCAATGATA<br>CCGCGGCTCCCACGCTCACCGGCTCCAGATTTATCAGCAATAAACCAGCCAGCCG<br>GAAGGGCCGAGCGCAGAAGTGGTCCTGCAACTTTATCCGCCTCCATCCAGTCTAT<br>TAATTGTTGCCGGGAAGCTAGAGTAAGTAGTTCGCCAGTTAATAGTTTGCGCAACG<br>TTGTTGCCATTGCTACAGGCATCGTGGTGTCACGCTCGTCGTTTGGTATGGCTTCA<br>TTCAGCTCCGGTTCCCAACGATCAAGGCGAGTTACATGATCCCCCATGTTGTGCAA<br>AAAAGCGGTTAGCTCCTTCGGTCCTCCGATCGTTGTCAGAAGTAAGTTGGCCGCA<br>GTGTTATCACTCATGGTTATGGCAGCACTGCATAATTCTCTTACTGTCATGCCATCC<br>GTAAGATGCTTTTCTGTGACTGGTGAGTACTCAACCAAGTCATTCTGAGAATAGTGT<br>ATGCGGCGACCGAGTTGCTCTTGCCCGGCGTCAATACGGGATAATACCGCGCCAC<br>ATAGCAGAACTTTAAAAGTGCTCATCATTGGAAAACGTTCTTCGGGGCGAAAACTC<br>TCAAGGATCTTACCGCTGTTGAGATCCAGTTCGATGTAACCCACTCGTGCACCCAA<br>CTGATCTTCAGCATCTTTTACTTTCACCAGCGTTTCTGGGTGAGCAAAAACAGGAA<br>GGCAAAATGCCGCAAAAAAGGGAATAAGGGCGACACGGAAATGTTGAATACTCATA<br>CTCTTCCTTTTTCAATATTATTGAAGCATTTATCAGGGTTATTGTCTCATGAGCGGAT<br>ACATATTTGAATGTATTTAGAAAAATAAACAAATAGGGGTTCCGCGCACATTTCCCC<br>GAAAAGTGCCACCTGAACGAAGCATCTGTGCTTCATTTTGTAGAACAAAAATGCAA<br>CGCGAGAGCGCTAATTTTTCAAACAAAGAATCTGAGCTGCATTTTTACAGAACAGAA<br>ATGCAACGCGAAAGCGCTATTTTACCAACGAAGAATCTGTGCTTCATTTTTGTAAAA<br>CAAAAATGCAACGCGAGAGCGCTAATTTTTCAAACAAAGAATCTGAGCTGCATTTTT<br>ACAGAACAGAAATGCAACGCGAGAGCGCTATTTTACCAACAAAGAATCTATACTTCT<br>TTTTTGTTCTACAAAAATGCATCCCGAGAGCGCTATTTTTCTAACAAAGCATCTTAG<br>ATTACTTTTTTTCTCCTTTGTGCGCTCTATAATGCAGTCTCTTGATAACTTTTTGCAC<br>TGTAGGTCCGTTAAGGTTAGAAGAAGGCTACTTTGGTGTCTATTTTCTCTTCCATAA<br>AAAAAGCCTGACTCCACTTCCCGCGTTTACTGATTACTAGCGAAGCTGCGGGTGCA<br>TTTTTTCAAGATAAAGGCATCCCCGATTATATTCTATACCGATGTGGATTGCGCATA<br>CTTTGTGAACAGAAAGTGATAGCGTTGATGATTCTTCATTGGTCAGAAAATTATGAA<br>CGGTTTCTTCTATTTTGTCTCTATATACTACGTATAGGAAATGTTTACATTTTCGTAT<br>TGTTTTCGATTCACTCTATGAATAGTTCTTACTACAATTTTTTTGTCTAAAGAGTAATA

	CTAGAGATAAACATAAAAAATGTAGAGGTCGAGTTTAGATGCAAGTTCAAGGAGCG<br>AAAGGTGGATGGGTAGGTTATATAGGGATATAGCACAGAGATATATAGCAAAGAGA<br>TACTTTTGAGCAATGTTTGTGGAAGCGGTATTCGCAATATTTTAGTAGCTCGTTACA<br>GTCCGGTGCGTTTTTGGTTTTTTGAAAGTGCGTCTTCAGAGCGCTTTTGGTTTTCAA<br>AAGCGCTCTGAAGTTCCTATACTTTCTAGAGAATAGGAACTTCGGAATAGGAACTTC<br>AAAGCGTTTCCGAAAACGAGCGCTTCCGAAAATGCAACGCGAGCTGCGCACATAC<br>AGCTCACTGTTCACGTCGCACCTATATCTGCGTGTTGCCTGTATATATATATACATG<br>AGAAGAACGGCATAGTGCGTGTTTATGCTTAAATGCGTACTTATATGCGTCTATTTA<br>TGTAGGATGAAAGGTAGTCTAGTACCTCCTGTGATATTATCCCATTCCATGCGGGG<br>TATCGTATGCTTCCTTCAGCACTACCCTTTAGCTGTTCTATATGCTGCCACTCCTCA<br>ATTGGATTAGTCTCATCCTTCAATGCTATCATTTCCTTTGATATTGGATCATACTAAG<br>AAACCATTATTATCATGACATTAACCTATAAAAATAGGCGTATCACGAGGCCCTTTC<br>GTC
pHV009	TCGCGCGAACCCAACTGCACAGAACAAAAACCTGCAGGAAACGAAGATAAATCATG<br>TCGAAAGCTACATATAAGGAACGTGCTGCTACTCATCCTAGTCCTGTTGCTGCCAA<br>GCTATTTAATATCATGCACGAAAAGCAAACAAACTTGTGTGCTTCATTGGATGTTCG<br>TACCACCAAGGAATTACTGGAGTTAGTTGAAGCATTAGGTCCCAAAATTTGTTTACT<br>AAAAACACATGTGGATATCTTGACTGATTTTTCCATGGAGGGCACAGTTAAGCCGC<br>TAAAGGCATTATCCGCCAAGTACAATTTTTTACTCTTCGAAGACAGAAAATTTGCTG<br>ACATTGGTAATACAGTCAAATTGCAGTACTCTGCGGGTGTATACAGAATAGCAGAA<br>TGGGCAGACATTACGAATGCACACGGTGTGGTGGGCCCAGGTATTGTTAGCGGTT<br>TGAAGCAGGCGGCAGAAGAAGTAACAAAGGAACCTAGAGGCCTTTTGATGTTAGC<br>AGAATTGTCATGCAAGGGCTCCCTATCTACTGGAGAATATACTAAGGGTACTGTTG<br>ACATTGCGAAGAGCGACAAAGATTTTGTTATCGGCTTTATTGCTCAAAGAGACATG<br>GGTGGAAGAGATGAAGGTTACGATTGGTTGATTATGACACCCGGTGTGGGTTTAGA<br>TGACAAGGGAGACGCATTGGGTCAACAGTATAGAACCGTGGATGATGTCGTTTCTA<br>CAGGATCTGACATTATTATTGTTGGAAGAGGACTATTTGCAAAGGGAAGGGATGCT<br>AAGGTAGAGGGTGAACGTTACAGAAAAGCAGGCTGGGAAGCATATTTGAGAAGAT<br>GCGGCCAGCAAAACTAAAAAACTGTATTATAAGTAAATGCATGTATACTAAACTCAC<br>AAATTAGAGCTTCAATTTAATTATATCAGTTATTACCCTGTGCTCGAGTCTTTGAAAA<br>GATAATGTATGATTATGCTTTCACTCATATTTATACAGAAACTTGATGTTTTCTTTCG<br>AGTATATACAAGGTGATTACATGTACGTTTGAAGTACAACTCTAGATTTTGTAGTGC<br>CCTCTTGGGCTAGCGGTAAAGGTGCGCATTTTTTCACACCCTACAATGTTCTGTTC<br>AAAAGATTTTGGTCAAACGCTGTAGAAGTGAAAGTTGGTGCGCATGTTTCGGCGTT<br>CGAAACTTCTCCGCAGTGAAAGATAAATGATCGGGTTTTAgAGCTATGCTGTTTTGA<br>ATGGTCCCAAAATGGCTTGGGAAGATGTCAGAGAGCTATGCTGTTTTGAATGGTCC<br>CAAAACTTTTTTTGTTTTTTATGTCTGCATAGCTTCAAAATGTTTCTACTCCTTTTTTA<br>CTCTTCCAGATTTTCTCGGACTCCGCGCATCGCCGTACCACTTCAAAACACCCAAG<br>CACAGCATACTAAATTTCCCCTCTTTCTTCCTCTAGGGTGTCGTTAATTACCCGTAC<br>TAAAGGTTTGGAAAAGAAAAAAGAGGCCGCCTCGTTTCTTTTTCTTCGTCGAAAAAG<br>GCAATAAAAATTTTTATCACGTTTCTTTTTCTTGAAAATTTTTTTTTTTGATTTTTTTCT<br>CTTTCGATGACCTCCCATTGATATTTAAGTTAATAAACGGTCTTCAATTTCTCAAGTT<br>TCAGTTTCATTTTTCTTGTTCTATTACAACTTTTTTTACTTCTTGCTCATTAGAAAGAA<br>AGCATAGCAATCTAATCTAAGTTTTCTAGATGGATTATAAAGATGACGATGACAAAC<br>CTCCAAAAAAGAAGAGAAAGGTCGATAAGAAATACTCAATAGGCTTAGATATCGGC<br>ACAAATAGCGTCGGATGGGCGGTGATCACTGATGAATATAAGGTTCCGTCTAAAAA<br>GTTCAAGGTTCTGGGAAATACAGACCGCCACAGTATCAAAAAAAATCTTATAGGGG<br>CTCTTTTATTTGACAGTGGAGAGACAGCGGAAGCGACTCGTCTCAAACGGACAGCT<br>CGTAGAAGGTATACACGTCGGAAGAATCGTATTTGTTATCTACAGGAGATTTTTTCA<br>AATGAGATGGCGAAAGTAGATGATAGTTTCTTTCATCGACTTGAAGAGTCTTTTTTG<br>GTGGAAGAAGACAAGAAGCATGAACGTCATCCTATTTTTGGAAATATAGTAGATGA<br>AGTTGCTTATCATGAGAAATATCCAACTATCTATCATCTGCGAAAAAAATTGGTAGA<br>TTCTACTTATAAAGCGGATTTGCGCTTAATCTATTTGGCCTTAGCGCATATGATTAA<br>GTTTCGTGGTCATTTTTTGATTGAGGGAGATTTAAATCCTGATAATAGTGATGTGGA<br>CAAACTATTTATCCAGTTGGTACAAACCTACAATCAATTATTTGAAGAAAACCCTATT<br>AACGCAAGTGGAGTAGATGCTAAAGCGATTCTTTCTGCACGATTGAGTAAATCAAG<br>ACGATTAGAAAATCTCATTGCTCAGCTCCCCGGTGAGAAGAAAAATGGCTTATTTG<br>GGAATCTCATTGCTTTGTCATTGGGTTTGACCCCTAATTTTAAATCAAATTTTGATTT<br>GGCAGAAGATGCTAAATTACAGCTTTCAAAAGATACTTACGATGATGATTTAGATAA<br>TTTATTGGCGCAAATTGGAGATCAATATGCTGATTTGTTTTTGGCAGCTAAGAATTT<br>ATCAGATGCTATTTTACTTTCAGATATCCTAAGAGTAAATACTGAAATAACTAAGGCT

	CCCCTATCAGCTTCAATGATTAAACGCTACGATGAACATCATCAAGACTTGACTCTT<br>TTAAAAGCTTTAGTTCGACAACAACTTCCAGAAAAGTATAAAGAAATCTTTTTTGATC<br>AATCAAAAAACGGATATGCAGGTTATATTGATGGGGGAGCTAGCCAAGAAGAATTT<br>TATAAATTTATCAAACCAATTTTAGAAAAAATGGATGGTACTGAGGAATTATTGGTGA<br>AACTAAATCGTGAAGATTTGCTGCGCAAGCAACGGACCTTTGACAACGGCTCTATT<br>ACCCATCAAATTCACTTGGGTGAGCTGCATGCTATTTTGAGAAGACAAGAAGACTTT<br>TATCCATTTTTAAAAGACAATCGTGAGAAGATTGAAAAAATCTTGACTTTTCGAATTC<br>CTTATTATGTTGGTCCATTGGCGCGTGGCAATAGTCGTTTTGCATGGATGACTCGG<br>AAGTCTGAAGAAACAATTACCCCATGGAATTTTGAAGAAGTTGTCGATAAAGGTGCT<br>TCAGCTCAATCATTTATTGAACGCATGACAAACTTTGATAAAAATCTTCCAAATGAAA<br>AAGTACTACCAAAACATAGTTTGCTTTATGAGTATTTTACGGTTTATAACGAATTGAC<br>AAAGGTCAAATATGTTACTGAAGGAATGCGAAAACCAGCATTTCTTTCAGGTGAACA<br>GAAGAAAGCCATTGTTGATTTACTCTTCAAAACAAATCGAAAAGTAACCGTTAAGCA<br>ATTAAAAGAAGATTATTTCAAAAAAATAGAATGTTTTGATAGTGTTGAAATTTCAGGA<br>GTTGAAGATAGATTTAATGCTTCATTAGGTACCTACCATGATTTGCTAAAAATTATTA<br>AAGATAAAGATTTTTTGGATAATGAAGAAAATGAAGATATCTTAGAGGATATTGTTTT<br>AACATTGACCTTATTTGAAGATAGGGAGATGATTGAGGAAAGACTTAAAACATATGC<br>TCACCTCTTTGATGATAAGGTGATGAAACAGCTTAAACGTCGCCGTTATACTGGTTG<br>GGGACGTTTGTCTCGAAAATTGATTAATGGTATTAGGGATAAGCAATCTGGCAAAA<br>CAATATTAGATTTTTTGAAATCAGATGGTTTTGCCAATCGCAATTTTATGCAGCTGAT<br>CCATGATGATAGTTTGACATTTAAAGAAGACATTCAAAAAGCACAAGTGTCTGGACA<br>AGGCGATAGTTTACATGAACATATTGCAAATTTAGCTGGTAGCCCTGCTATTAAAAA<br>AGGTATTTTACAGACTGTAAAAGTTGTTGATGAATTGGTCAAAGTAATGGGGCGGC<br>ATAAGCCAGAAAATATCGTTATTGAAATGGCACGTGAAAATCAGACAACTCAAAAG<br>GGCCAGAAAAATTCGCGAGAGCGTATGAAACGAATCGAAGAAGGTATCAAAGAATT<br>AGGAAGTCAGATTCTTAAAGAGCATCCTGTTGAAAATACTCAATTGCAAAATGAAAA<br>GCTCTATCTCTATTATCTCCAAAATGGAAGAGACATGTATGTGGACCAAGAATTAGA<br>TATTAATCGTTTAAGTGATTATGATGTCGATCACATTGTTCCACAAAGTTTCCTTAAA<br>GACGATTCAATAGACAATAAGGTCTTAACGCGTTCTGATAAAAATCGTGGTAAATCG<br>GATAACGTTCCAAGTGAAGAAGTAGTCAAAAAGATGAAAAACTATTGGAGACAACTT<br>CTAAACGCCAAGTTAATCACTCAACGTAAGTTTGATAATTTAACGAAAGCTGAACGT<br>GGAGGTTTGAGTGAACTTGATAAAGCTGGTTTTATCAAACGCCAATTGGTTGAAACT<br>CGCCAAATCACTAAGCATGTGGCACAAATTTTGGATAGTCGCATGAATACTAAATAC<br>GATGAAAATGATAAACTTATTCGAGAGGTTAAAGTGATTACCTTAAAATCTAAATTAG<br>TTTCTGACTTCCGAAAAGATTTCCAATTCTATAAAGTACGTGAGATTAACAATTACCA<br>TCATGCCCATGATGCGTATCTAAATGCCGTCGTTGGAACTGCTTTGATTAAGAAATA<br>TCCAAAACTTGAATCGGAGTTTGTCTATGGTGATTATAAAGTTTATGATGTTCGTAA<br>AATGATTGCTAAGTCTGAGCAAGAAATAGGCAAAGCAACCGCAAAATATTTCTTTTA<br>CTCTAATATCATGAACTTCTTCAAAACAGAAATTACACTTGCAAATGGAGAGATTCG<br>CAAACGCCCTCTAATCGAAACTAATGGGGAAACTGGAGAAATTGTCTGGGATAAAG<br>GGCGAGATTTTGCCACAGTGCGCAAAGTATTGTCCATGCCCCAAGTCAATATTGTC<br>AAGAAAACAGAAGTACAGACAGGCGGATTCTCCAAGGAGTCAATTTTACCAAAAAG<br>AAATTCGGACAAGCTTATTGCTCGTAAAAAAGACTGGGATCCAAAAAAATATGGTG<br>GTTTTGATAGTCCAACGGTAGCTTATTCAGTCCTAGTGGTTGCTAAGGTGGAAAAA<br>GGGAAATCGAAGAAGTTAAAATCCGTTAAAGAGTTACTAGGGATCACAATTATGGA<br>AAGAAGTTCCTTTGAAAAAAATCCGATTGACTTTTTAGAAGCTAAAGGATATAAGGA<br>AGTTAAAAAAGACTTAATCATTAAACTACCTAAATATAGTCTTTTTGAGTTAGAAAAC<br>GGTCGTAAACGGATGCTGGCTAGTGCCGGAGAATTACAAAAAGGAAATGAGCTGG<br>CTCTGCCAAGCAAATATGTGAATTTTTTATATTTAGCTAGTCATTATGAAAAGTTGAA<br>GGGTAGTCCAGAAGATAACGAACAAAAACAATTGTTTGTGGAGCAGCATAAGCATT<br>ATTTAGATGAGATTATTGAGCAAATCAGTGAATTTTCTAAGCGTGTTATTTTAGCAGA<br>TGCCAATTTAGATAAAGTTCTTAGTGCATATAACAAACATAGAGACAAACCAATACG<br>TGAACAAGCAGAAAATATTATTCATTTATTTACGTTGACGAATCTTGGAGCTCCCGC<br>TGCTTTTAAATATTTTGATACAACAATTGATCGTAAACGATATACGTCTACAAAAGAA<br>GTTTTAGATGCCACTCTTATCCATCAATCCATCACTGGTCTTTATGAAACACGCATT<br>GATTTGAGTCAGCTAGGAGGTGACCCTCCAAAAAAGAAGAGAAAGGTCTGAGCGG<br>ATCTCTTATGTCTTTACGATTTATAGTTTTCATTATCAAGTATGCCTATATTAGTATAT<br>AGCATCTTTAGATGACAGTGTTCGAAGTTTCACGAATAAAAGATAATATTCTACTTTT<br>TGCTCCCACCGCGTTTGCTAGCACGAGTGAACACCATCCCTCGCCTGTGAGTTGTA<br>CCCATTCCTCTAAACTGTAGACATGGTAGCTTCAGCAGTGTTCGTTATGTACGGCA<br>TCCTCCAACAAACAGTCGGTTATAGTTTGTCCTGCTCCTCTGAATCGAGTCCCTCG

	ATATTTCTCATACTAGTTCTAGAGATCTGCCAATTGAACATAACATGGTAGTTACATA<br>TACTAGTAATATGGTTCGGCACACATTAAAAGTATAAAAACTATCTGAATTACGAATT<br>ACATATATTGGTCATAAAAATCAATCAATCATCGTGTGTTTTATATGTCTCTTATCTA<br>AGTATAAGAATATCCATAGTTAATATTCACTTACGCTACCTTTTAACCTGTAATCATT<br>GTCAACAGGATATGTTAACGACCCACATTGATAAACGCTAGTATTTCTTTTTCCTCT<br>TCTTATTGGCCGGCTGTCTCTATACTCCCCTATAGTCTGTTTCTTTTCGTTTCGATT<br>GTTTTACGTTTGAGGCCTCGTGGCGCACATGGTACGCTGTGGTGCTCGCGGCTGG<br>GAACGAAACTCTGGGAGCTGCGATTGGCAGGAACCATTCAAAACAGCATAGCAAG<br>TTAAAATAAGGCTAGTCCGTTATCAACTTGAAAAAGTGGCACCGAGTCGGTGCTTTT<br>TTTCCATATCCAACTTCCAATTTAATCTTTCTTTTTTAATTTTCACTTATTTGCGATAC<br>AGAAAGACCCTGCAGGCATGCAAGCTTGGCGTAATCATGGTCATAGCTGTTTCCTG<br>TGTGAAATTGTTATCCGCTCACAATTCCACACAACATACGAGCCGGAAGCATAAAG<br>TGTAAAGCCTGGGGTGCCTAATGAGTGAGCTAACTCACATTAATTGCGTTGCGCTC<br>ACTGCCCGCTTTCCAGTCGGGAAACCTGTCGTGCCAGCTGCATTAATGAATCGGC<br>CAACGCGCGGGGAGAGGCGGTTTGCGTATTGGGCGCTCTTCCGCTTCCTCGCTCA<br>CTGACTCGCTGCGCTCGGTCGTTCGGCTGCGGCGAGCGGTATCAGCTCACTCAAA<br>GGCGGTAATACGGTTATCCACAGAATCAGGGGATAACGCAGGAAAGAACATGTGA<br>GCAAAAGGCCAGCAAAAGGCCAGGAACCGTAAAAAGGCCGCGTTGCTGGCGTTTT<br>TCCATAGGCTCCGCCCCCCTGACGAGCATCACAAAAATCGACGCTCAAGTCAGAG<br>GTGGCGAAACCCGACAGGACTATAAAGATACCAGGCGTTTCCCCCTGGAAGCTCC<br>CTCGTGCGCTCTCCTGTTCCGACCCTGCCGCTTACCGGATACCTGTCCGCCTTTCT<br>CCCTTCGGGAAGCGTGGCGCTTTCTCATAGCTCACGCTGTAGGTATCTCAGTTCG<br>GTGTAGGTCGTTCGCTCCAAGCTGGGCTGTGTGCACGAACCCCCCGTTCAGCCCG<br>ACCGCTGCGCCTTATCCGGTAACTATCGTCTTGAGTCCAACCCGGTAAGACACGAC<br>TTATCGCCACTGGCAGCAGCCACTGGTAACAGGATTAGCAGAGCGAGGTATGTAG<br>GCGGTGCTACAGAGTTCTTGAAGTGGTGGCCTAACTACGGCTACACTAGAAGAAC<br>AGTATTTGGTATCTGCGCTCTGCTGAAGCCAGTTACCTTCGGAAAAAGAGTTGGTA<br>GCTCTTGATCCGGCAAACAAACCACCGCTGGTAGCGGTGGTTTTTTTGTTTGCAAG<br>CAGCAGATTACGCGCAGAAAAAAAGGATCTCAAGAAGATCCTTTGATCTTTTCTAC<br>GGGGTCTGACGCTCAGTGGAACGAAAACTCACGTTAAGGGATTTTGGTCATGAGAT<br>TATCAAAAAGGATCTTCACCTAGATCCTTTTAAATTAAAAATGAAGTTTTAAATCAAT<br>CTAAAGTATATATGAGTAAACTTGGTCTGACAGTTACCAATGCTTAATCAGTGAGGC<br>ACCTATCTCAGCGATCTGTCTATTTCGTTCATCCATAGTTGCCTGACTCCCCGTCGT<br>GTAGATAACTACGATACGGGAGGGCTTACCATCTGGCCCCAGTGCTGCAATGATA<br>CCGCGGCTCCCACGCTCACCGGCTCCAGATTTATCAGCAATAAACCAGCCAGCCG<br>GAAGGGCCGAGCGCAGAAGTGGTCCTGCAACTTTATCCGCCTCCATCCAGTCTAT<br>TAATTGTTGCCGGGAAGCTAGAGTAAGTAGTTCGCCAGTTAATAGTTTGCGCAACG<br>TTGTTGCCATTGCTACAGGCATCGTGGTGTCACGCTCGTCGTTTGGTATGGCTTCA<br>TTCAGCTCCGGTTCCCAACGATCAAGGCGAGTTACATGATCCCCCATGTTGTGCAA<br>AAAAGCGGTTAGCTCCTTCGGTCCTCCGATCGTTGTCAGAAGTAAGTTGGCCGCA<br>GTGTTATCACTCATGGTTATGGCAGCACTGCATAATTCTCTTACTGTCATGCCATCC<br>GTAAGATGCTTTTCTGTGACTGGTGAGTACTCAACCAAGTCATTCTGAGAATAGTGT<br>ATGCGGCGACCGAGTTGCTCTTGCCCGGCGTCAATACGGGATAATACCGCGCCAC<br>ATAGCAGAACTTTAAAAGTGCTCATCATTGGAAAACGTTCTTCGGGGCGAAAACTC<br>TCAAGGATCTTACCGCTGTTGAGATCCAGTTCGATGTAACCCACTCGTGCACCCAA<br>CTGATCTTCAGCATCTTTTACTTTCACCAGCGTTTCTGGGTGAGCAAAAACAGGAA<br>GGCAAAATGCCGCAAAAAAGGGAATAAGGGCGACACGGAAATGTTGAATACTCATA<br>CTCTTCCTTTTTCAATATTATTGAAGCATTTATCAGGGTTATTGTCTCATGAGCGGAT<br>ACATATTTGAATGTATTTAGAAAAATAAACAAATAGGGGTTCCGCGCACATTTCCCC<br>GAAAAGTGCCACCTGAACGAAGCATCTGTGCTTCATTTTGTAGAACAAAAATGCAA<br>CGCGAGAGCGCTAATTTTTCAAACAAAGAATCTGAGCTGCATTTTTACAGAACAGAA<br>ATGCAACGCGAAAGCGCTATTTTACCAACGAAGAATCTGTGCTTCATTTTTGTAAAA<br>CAAAAATGCAACGCGAGAGCGCTAATTTTTCAAACAAAGAATCTGAGCTGCATTTTT<br>ACAGAACAGAAATGCAACGCGAGAGCGCTATTTTACCAACAAAGAATCTATACTTCT<br>TTTTTGTTCTACAAAAATGCATCCCGAGAGCGCTATTTTTCTAACAAAGCATCTTAG<br>ATTACTTTTTTTCTCCTTTGTGCGCTCTATAATGCAGTCTCTTGATAACTTTTTGCAC<br>TGTAGGTCCGTTAAGGTTAGAAGAAGGCTACTTTGGTGTCTATTTTCTCTTCCATAA<br>AAAAAGCCTGACTCCACTTCCCGCGTTTACTGATTACTAGCGAAGCTGCGGGTGCA<br>TTTTTTCAAGATAAAGGCATCCCCGATTATATTCTATACCGATGTGGATTGCGCATA<br>CTTTGTGAACAGAAAGTGATAGCGTTGATGATTCTTCATTGGTCAGAAAATTATGAA<br>CGGTTTCTTCTATTTTGTCTCTATATACTACGTATAGGAAATGTTTACATTTTCGTAT

	TGTTTTCGATTCACTCTATGAATAGTTCTTACTACAATTTTTTTGTCTAAAGAGTAATA<br>CTAGAGATAAACATAAAAAATGTAGAGGTCGAGTTTAGATGCAAGTTCAAGGAGCG<br>AAAGGTGGATGGGTAGGTTATATAGGGATATAGCACAGAGATATATAGCAAAGAGA<br>TACTTTTGAGCAATGTTTGTGGAAGCGGTATTCGCAATATTTTAGTAGCTCGTTACA<br>GTCCGGTGCGTTTTTGGTTTTTTGAAAGTGCGTCTTCAGAGCGCTTTTGGTTTTCAA<br>AAGCGCTCTGAAGTTCCTATACTTTCTAGAGAATAGGAACTTCGGAATAGGAACTTC<br>AAAGCGTTTCCGAAAACGAGCGCTTCCGAAAATGCAACGCGAGCTGCGCACATAC<br>AGCTCACTGTTCACGTCGCACCTATATCTGCGTGTTGCCTGTATATATATATACATG<br>AGAAGAACGGCATAGTGCGTGTTTATGCTTAAATGCGTACTTATATGCGTCTATTTA<br>TGTAGGATGAAAGGTAGTCTAGTACCTCCTGTGATATTATCCCATTCCATGCGGGG<br>TATCGTATGCTTCCTTCAGCACTACCCTTTAGCTGTTCTATATGCTGCCACTCCTCA<br>ATTGGATTAGTCTCATCCTTCAATGCTATCATTTCCTTTGATATTGGATCATACTAAG<br>AAACCATTATTATCATGACATTAACCTATAAAAATAGGCGTATCACGAGGCCCTTTC<br>GTC
pHV010	TCGCGCGAACCCAACTGCACAGAACAAAAACCTGCAGGAAACGAAGATAAATCATG<br>TCGAAAGCTACATATAAGGAACGTGCTGCTACTCATCCTAGTCCTGTTGCTGCCAA<br>GCTATTTAATATCATGCACGAAAAGCAAACAAACTTGTGTGCTTCATTGGATGTTCG<br>TACCACCAAGGAATTACTGGAGTTAGTTGAAGCATTAGGTCCCAAAATTTGTTTACT<br>AAAAACACATGTGGATATCTTGACTGATTTTTCCATGGAGGGCACAGTTAAGCCGC<br>TAAAGGCATTATCCGCCAAGTACAATTTTTTACTCTTCGAAGACAGAAAATTTGCTG<br>ACATTGGTAATACAGTCAAATTGCAGTACTCTGCGGGTGTATACAGAATAGCAGAA<br>TGGGCAGACATTACGAATGCACACGGTGTGGTGGGCCCAGGTATTGTTAGCGGTT<br>TGAAGCAGGCGGCAGAAGAAGTAACAAAGGAACCTAGAGGCCTTTTGATGTTAGC<br>AGAATTGTCATGCAAGGGCTCCCTATCTACTGGAGAATATACTAAGGGTACTGTTG<br>ACATTGCGAAGAGCGACAAAGATTTTGTTATCGGCTTTATTGCTCAAAGAGACATG<br>GGTGGAAGAGATGAAGGTTACGATTGGTTGATTATGACACCCGGTGTGGGTTTAGA<br>TGACAAGGGAGACGCATTGGGTCAACAGTATAGAACCGTGGATGATGTCGTTTCTA<br>CAGGATCTGACATTATTATTGTTGGAAGAGGACTATTTGCAAAGGGAAGGGATGCT<br>AAGGTAGAGGGTGAACGTTACAGAAAAGCAGGCTGGGAAGCATATTTGAGAAGAT<br>GCGGCCAGCAAAACTAAAAAACTGTATTATAAGTAAATGCATGTATACTAAACTCAC<br>AAATTAGAGCTTCAATTTAATTATATCAGTTATTACCCTGTGCTCGAGTCTTTGAAAA<br>GATAATGTATGATTATGCTTTCACTCATATTTATACAGAAACTTGATGTTTTCTTTCG<br>AGTATATACAAGGTGATTACATGTACGTTTGAAGTACAACTCTAGATTTTGTAGTGC<br>CCTCTTGGGCTAGCGGTAAAGGTGCGCATTTTTTCACACCCTACAATGTTCTGTTC<br>AAAAGATTTTGGTCAAACGCTGTAGAAGTGAAAGTTGGTGCGCATGTTTCGGCGTT<br>CGAAACTTCTCCGCAGTGAAAGATAAATGATCGGGTTTTAgAGCTATGCTGTTTTGA<br>ATGGTCCCAAAGCTGTTTACGAAGGTTACGAAGAGCTATGCTGTTTTGAATGGTCC<br>CAAAACTTTTTTTGTTTTTTATGTCTGCATAGCTTCAAAATGTTTCTACTCCTTTTTA<br>CTCTTCCAGATTTTCTCGGACTCCGCGCATCGCCGTACCACTTCAAAACACCCAAG<br>CACAGCATACTAAATTTCCCCTCTTTCTTCCTCTAGGGTGTCGTTAATTACCCGTAC<br>TAAAGGTTTGGAAAAGAAAAAAGAGGCCGCCTCGTTTCTTTTTCTTCGTCGAAAAAG<br>GCAATAAAAATTTTTATCACGTTTCTTTTTCTTGAAAATTTTTTTTTTTGATTTTTTTCT<br>CTTTCGATGACCTCCCATTGATATTTAAGTTAATAAACGGTCTTCAATTTCTCAAGTT<br>TCAGTTTCATTTTTCTTGTTCTATTACAACTTTTTTTACTTCTTGCTCATTAGAAAGAA<br>AGCATAGCAATCTAATCTAAGTTTTCTAGATGGATTATAAAGATGACGATGACAAAC<br>CTCCAAAAAAGAAGAGAAAGGTCGATAAGAAATACTCAATAGGCTTAGATATCGGC<br>ACAAATAGCGTCGGATGGGCGGTGATCACTGATGAATATAAGGTTCCGTCTAAAAA<br>GTTCAAGGTTCTGGGAAATACAGACCGCCACAGTATCAAAAAAAATCTTATAGGGG<br>CTCTTTTATTTGACAGTGGAGAGACAGCGGAAGCGACTCGTCTCAAACGGACAGCT<br>CGTAGAAGGTATACACGTCGGAAGAATCGTATTTGTTATCTACAGGAGATTTTTTCA<br>AATGAGATGGCGAAAGTAGATGATAGTTTCTTTCATCGACTTGAAGAGTCTTTTTTG<br>GTGGAAGAAGACAAGAAGCATGAACGTCATCCTATTTTTGGAAATATAGTAGATGA<br>AGTTGCTTATCATGAGAAATATCCAACTATCTATCATCTGCGAAAAAAATTGGTAGA<br>TTCTACTTATAAAGCGGATTTGCGCTTAATCTATTTGGCCTTAGCGCATATGATTAA<br>GTTTCGTGGTCATTTTTTGATTGAGGGAGATTTAAATCCTGATAATAGTGATGTGGA<br>CAAACTATTTATCCAGTTGGTACAAACCTACAATCAATTATTTGAAGAAAACCCTATT<br>AACGCAAGTGGAGTAGATGCTAAAGCGATTCTTTCTGCACGATTGAGTAAATCAAG<br>ACGATTAGAAAATCTCATTGCTCAGCTCCCCGGTGAGAAGAAAAATGGCTTATTTG<br>GGAATCTCATTGCTTTGTCATTGGGTTTGACCCCTAATTTTAAATCAAATTTTGATTT<br>GGCAGAAGATGCTAAATTACAGCTTTCAAAAGATACTTACGATGATGATTTAGATAA<br>TTTATTGGCGCAAATTGGAGATCAATATGCTGATTTGTTTTTGGCAGCTAAGAATTT

	ATCAGATGCTATTTTACTTTCAGATATCCTAAGAGTAAATACTGAAATAACTAAGGCT<br>CCCCTATCAGCTTCAATGATTAAACGCTACGATGAACATCATCAAGACTTGACTCTT<br>TTAAAAGCTTTAGTTCGACAACAACTTCCAGAAAAGTATAAAGAAATCTTTTTTGATC<br>AATCAAAAAACGGATATGCAGGTTATATTGATGGGGGAGCTAGCCAAGAAGAATTT<br>TATAAATTTATCAAACCAATTTTAGAAAAAATGGATGGTACTGAGGAATTATTGGTGA<br>AACTAAATCGTGAAGATTTGCTGCGCAAGCAACGGACCTTTGACAACGGCTCTATT<br>ACCCATCAAATTCACTTGGGTGAGCTGCATGCTATTTTGAGAAGACAAGAAGACTTT<br>TATCCATTTTTAAAAGACAATCGTGAGAAGATTGAAAAAATCTTGACTTTTCGAATTC<br>CTTATTATGTTGGTCCATTGGCGCGTGGCAATAGTCGTTTTGCATGGATGACTCGG<br>AAGTCTGAAGAAACAATTACCCCATGGAATTTTGAAGAAGTTGTCGATAAAGGTGCT<br>TCAGCTCAATCATTTATTGAACGCATGACAAACTTTGATAAAAATCTTCCAAATGAAA<br>AAGTACTACCAAAACATAGTTTGCTTTATGAGTATTTTACGGTTTATAACGAATTGAC<br>AAAGGTCAAATATGTTACTGAAGGAATGCGAAAACCAGCATTTCTTTCAGGTGAACA<br>GAAGAAAGCCATTGTTGATTTACTCTTCAAAACAAATCGAAAAGTAACCGTTAAGCA<br>ATTAAAAGAAGATTATTTCAAAAAAATAGAATGTTTTGATAGTGTTGAAATTTCAGGA<br>GTTGAAGATAGATTTAATGCTTCATTAGGTACCTACCATGATTTGCTAAAAATTATTA<br>AAGATAAAGATTTTTTGGATAATGAAGAAAATGAAGATATCTTAGAGGATATTGTTTT<br>AACATTGACCTTATTTGAAGATAGGGAGATGATTGAGGAAAGACTTAAAACATATGC<br>TCACCTCTTTGATGATAAGGTGATGAAACAGCTTAAACGTCGCCGTTATACTGGTTG<br>GGGACGTTTGTCTCGAAAATTGATTAATGGTATTAGGGATAAGCAATCTGGCAAAA<br>CAATATTAGATTTTTTGAAATCAGATGGTTTTGCCAATCGCAATTTTATGCAGCTGAT<br>CCATGATGATAGTTTGACATTTAAAGAAGACATTCAAAAAGCACAAGTGTCTGGACA<br>AGGCGATAGTTTACATGAACATATTGCAAATTTAGCTGGTAGCCCTGCTATTAAAAA<br>AGGTATTTTACAGACTGTAAAAGTTGTTGATGAATTGGTCAAAGTAATGGGGCGGC<br>ATAAGCCAGAAAATATCGTTATTGAAATGGCACGTGAAAATCAGACAACTCAAAAG<br>GGCCAGAAAAATTCGCGAGAGCGTATGAAACGAATCGAAGAAGGTATCAAAGAATT<br>AGGAAGTCAGATTCTTAAAGAGCATCCTGTTGAAAATACTCAATTGCAAAATGAAAA<br>GCTCTATCTCTATTATCTCCAAAATGGAAGAGACATGTATGTGGACCAAGAATTAGA<br>TATTAATCGTTTAAGTGATTATGATGTCGATCACATTGTTCCACAAAGTTTCCTTAAA<br>GACGATTCAATAGACAATAAGGTCTTAACGCGTTCTGATAAAAATCGTGGTAAATCG<br>GATAACGTTCCAAGTGAAGAAGTAGTCAAAAAGATGAAAAACTATTGGAGACAACTT<br>CTAAACGCCAAGTTAATCACTCAACGTAAGTTTGATAATTTAACGAAAGCTGAACGT<br>GGAGGTTTGAGTGAACTTGATAAAGCTGGTTTTATCAAACGCCAATTGGTTGAAACT<br>CGCCAAATCACTAAGCATGTGGCACAAATTTTGGATAGTCGCATGAATACTAAATAC<br>GATGAAAATGATAAACTTATTCGAGAGGTTAAAGTGATTACCTTAAAATCTAAATTAG<br>TTTCTGACTTCCGAAAAGATTTCCAATTCTATAAAGTACGTGAGATTAACAATTACCA<br>TCATGCCCATGATGCGTATCTAAATGCCGTCGTTGGAACTGCTTTGATTAAGAAATA<br>TCCAAAACTTGAATCGGAGTTTGTCTATGGTGATTATAAAGTTTATGATGTTCGTAA<br>AATGATTGCTAAGTCTGAGCAAGAAATAGGCAAAGCAACCGCAAAATATTTCTTTTA<br>CTCTAATATCATGAACTTCTTCAAAACAGAAATTACACTTGCAAATGGAGAGATTCG<br>CAAACGCCCTCTAATCGAAACTAATGGGGAAACTGGAGAAATTGTCTGGGATAAAG<br>GGCGAGATTTTGCCACAGTGCGCAAAGTATTGTCCATGCCCCAAGTCAATATTGTC<br>AAGAAAACAGAAGTACAGACAGGCGGATTCTCCAAGGAGTCAATTTTACCAAAAAG<br>AAATTCGGACAAGCTTATTGCTCGTAAAAAAGACTGGGATCCAAAAAAATATGGTG<br>GTTTTGATAGTCCAACGGTAGCTTATTCAGTCCTAGTGGTTGCTAAGGTGGAAAAA<br>GGGAAATCGAAGAAGTTAAAATCCGTTAAAGAGTTACTAGGGATCACAATTATGGA<br>AAGAAGTTCCTTTGAAAAAAATCCGATTGACTTTTTAGAAGCTAAAGGATATAAGGA<br>AGTTAAAAAAGACTTAATCATTAAACTACCTAAATATAGTCTTTTTGAGTTAGAAAAC<br>GGTCGTAAACGGATGCTGGCTAGTGCCGGAGAATTACAAAAAGGAAATGAGCTGG<br>CTCTGCCAAGCAAATATGTGAATTTTTTATATTTAGCTAGTCATTATGAAAAGTTGAA<br>GGGTAGTCCAGAAGATAACGAACAAAAACAATTGTTTGTGGAGCAGCATAAGCATT<br>ATTTAGATGAGATTATTGAGCAAATCAGTGAATTTTCTAAGCGTGTTATTTTAGCAGA<br>TGCCAATTTAGATAAAGTTCTTAGTGCATATAACAAACATAGAGACAAACCAATACG<br>TGAACAAGCAGAAAATATTATTCATTTATTTACGTTGACGAATCTTGGAGCTCCCGC<br>TGCTTTTAAATATTTTGATACAACAATTGATCGTAAACGATATACGTCTACAAAAGAA<br>GTTTTAGATGCCACTCTTATCCATCAATCCATCACTGGTCTTTATGAAACACGCATT<br>GATTTGAGTCAGCTAGGAGGTGACCCTCCAAAAAAGAAGAGAAAGGTCTGAGCGG<br>ATCTCTTATGTCTTTACGATTTATAGTTTTCATTATCAAGTATGCCTATATTAGTATAT<br>AGCATCTTTAGATGACAGTGTTCGAAGTTTCACGAATAAAAGATAATATTCTACTTTT<br>TGCTCCCACCGCGTTTGCTAGCACGAGTGAACACCATCCCTCGCCTGTGAGTTGTA<br>CCCATTCCTCTAAACTGTAGACATGGTAGCTTCAGCAGTGTTCGTTATGTACGGCA

	TCCTCCAACAAACAGTCGGTTATAGTTTGTCCTGCTCCTCTGAATCGAGTCCCTCG<br>ATATTTCTCATACTAGTTCTAGAGATCTGCCAATTGAACATAACATGGTAGTTACATA<br>TACTAGTAATATGGTTCGGCACACATTAAAAGTATAAAAACTATCTGAATTACGAATT<br>ACATATATTGGTCATAAAAATCAATCAATCATCGTGTGTTTTATATGTCTCTTATCTA<br>AGTATAAGAATATCCATAGTTAATATTCACTTACGCTACCTTTTAACCTGTAATCATT<br>GTCAACAGGATATGTTAACGACCCACATTGATAAACGCTAGTATTTCTTTTTCCTCT<br>TCTTATTGGCCGGCTGTCTCTATACTCCCCTATAGTCTGTTTCTTTTCGTTTCGATT<br>GTTTTACGTTTGAGGCCTCGTGGCGCACATGGTACGCTGTGGTGCTCGCGGCTGG<br>GAACGAAACTCTGGGAGCTGCGATTGGCAGGAACCATTCAAAACAGCATAGCAAG<br>TTAAAATAAGGCTAGTCCGTTATCAACTTGAAAAAGTGGCACCGAGTCGGTGCTTTT<br>TTTCCATATCCAACTTCCAATTTAATCTTTCTTTTTTAATTTTCACTTATTTGCGATAC<br>AGAAAGACCCTGCAGGCATGCAAGCTTGGCGTAATCATGGTCATAGCTGTTTCCTG<br>TGTGAAATTGTTATCCGCTCACAATTCCACACAACATACGAGCCGGAAGCATAAAG<br>TGTAAAGCCTGGGGTGCCTAATGAGTGAGCTAACTCACATTAATTGCGTTGCGCTC<br>ACTGCCCGCTTTCCAGTCGGGAAACCTGTCGTGCCAGCTGCATTAATGAATCGGC<br>CAACGCGCGGGGAGAGGCGGTTTGCGTATTGGGCGCTCTTCCGCTTCCTCGCTCA<br>CTGACTCGCTGCGCTCGGTCGTTCGGCTGCGGCGAGCGGTATCAGCTCACTCAAA<br>GGCGGTAATACGGTTATCCACAGAATCAGGGGATAACGCAGGAAAGAACATGTGA<br>GCAAAAGGCCAGCAAAAGGCCAGGAACCGTAAAAAGGCCGCGTTGCTGGCGTTTT<br>TCCATAGGCTCCGCCCCCCTGACGAGCATCACAAAAATCGACGCTCAAGTCAGAG<br>GTGGCGAAACCCGACAGGACTATAAAGATACCAGGCGTTTCCCCCTGGAAGCTCC<br>CTCGTGCGCTCTCCTGTTCCGACCCTGCCGCTTACCGGATACCTGTCCGCCTTTCT<br>CCCTTCGGGAAGCGTGGCGCTTTCTCATAGCTCACGCTGTAGGTATCTCAGTTCG<br>GTGTAGGTCGTTCGCTCCAAGCTGGGCTGTGTGCACGAACCCCCCGTTCAGCCCG<br>ACCGCTGCGCCTTATCCGGTAACTATCGTCTTGAGTCCAACCCGGTAAGACACGAC<br>TTATCGCCACTGGCAGCAGCCACTGGTAACAGGATTAGCAGAGCGAGGTATGTAG<br>GCGGTGCTACAGAGTTCTTGAAGTGGTGGCCTAACTACGGCTACACTAGAAGAAC<br>AGTATTTGGTATCTGCGCTCTGCTGAAGCCAGTTACCTTCGGAAAAAGAGTTGGTA<br>GCTCTTGATCCGGCAAACAAACCACCGCTGGTAGCGGTGGTTTTTTTGTTTGCAAG<br>CAGCAGATTACGCGCAGAAAAAAAGGATCTCAAGAAGATCCTTTGATCTTTTCTAC<br>GGGGTCTGACGCTCAGTGGAACGAAAACTCACGTTAAGGGATTTTGGTCATGAGAT<br>TATCAAAAAGGATCTTCACCTAGATCCTTTTAAATTAAAAATGAAGTTTTAAATCAAT<br>CTAAAGTATATATGAGTAAACTTGGTCTGACAGTTACCAATGCTTAATCAGTGAGGC<br>ACCTATCTCAGCGATCTGTCTATTTCGTTCATCCATAGTTGCCTGACTCCCCGTCGT<br>GTAGATAACTACGATACGGGAGGGCTTACCATCTGGCCCCAGTGCTGCAATGATA<br>CCGCGGCTCCCACGCTCACCGGCTCCAGATTTATCAGCAATAAACCAGCCAGCCG<br>GAAGGGCCGAGCGCAGAAGTGGTCCTGCAACTTTATCCGCCTCCATCCAGTCTAT<br>TAATTGTTGCCGGGAAGCTAGAGTAAGTAGTTCGCCAGTTAATAGTTTGCGCAACG<br>TTGTTGCCATTGCTACAGGCATCGTGGTGTCACGCTCGTCGTTTGGTATGGCTTCA<br>TTCAGCTCCGGTTCCCAACGATCAAGGCGAGTTACATGATCCCCCATGTTGTGCAA<br>AAAAGCGGTTAGCTCCTTCGGTCCTCCGATCGTTGTCAGAAGTAAGTTGGCCGCA<br>GTGTTATCACTCATGGTTATGGCAGCACTGCATAATTCTCTTACTGTCATGCCATCC<br>GTAAGATGCTTTTCTGTGACTGGTGAGTACTCAACCAAGTCATTCTGAGAATAGTGT<br>ATGCGGCGACCGAGTTGCTCTTGCCCGGCGTCAATACGGGATAATACCGCGCCAC<br>ATAGCAGAACTTTAAAAGTGCTCATCATTGGAAAACGTTCTTCGGGGCGAAAACTC<br>TCAAGGATCTTACCGCTGTTGAGATCCAGTTCGATGTAACCCACTCGTGCACCCAA<br>CTGATCTTCAGCATCTTTTACTTTCACCAGCGTTTCTGGGTGAGCAAAAACAGGAA<br>GGCAAAATGCCGCAAAAAAGGGAATAAGGGCGACACGGAAATGTTGAATACTCATA<br>CTCTTCCTTTTTCAATATTATTGAAGCATTTATCAGGGTTATTGTCTCATGAGCGGAT<br>ACATATTTGAATGTATTTAGAAAAATAAACAAATAGGGGTTCCGCGCACATTTCCCC<br>GAAAAGTGCCACCTGAACGAAGCATCTGTGCTTCATTTTGTAGAACAAAAATGCAA<br>CGCGAGAGCGCTAATTTTTCAAACAAAGAATCTGAGCTGCATTTTTACAGAACAGAA<br>ATGCAACGCGAAAGCGCTATTTTACCAACGAAGAATCTGTGCTTCATTTTTGTAAAA<br>CAAAAATGCAACGCGAGAGCGCTAATTTTTCAAACAAAGAATCTGAGCTGCATTTTT<br>ACAGAACAGAAATGCAACGCGAGAGCGCTATTTTACCAACAAAGAATCTATACTTCT<br>TTTTTGTTCTACAAAAATGCATCCCGAGAGCGCTATTTTTCTAACAAAGCATCTTAG<br>ATTACTTTTTTTCTCCTTTGTGCGCTCTATAATGCAGTCTCTTGATAACTTTTTGCAC<br>TGTAGGTCCGTTAAGGTTAGAAGAAGGCTACTTTGGTGTCTATTTTCTCTTCCATAA<br>AAAAAGCCTGACTCCACTTCCCGCGTTTACTGATTACTAGCGAAGCTGCGGGTGCA<br>TTTTTTCAAGATAAAGGCATCCCCGATTATATTCTATACCGATGTGGATTGCGCATA<br>CTTTGTGAACAGAAAGTGATAGCGTTGATGATTCTTCATTGGTCAGAAAATTATGAA

	CGGTTTCTTCTATTTTGTCTCTATATACTACGTATAGGAAATGTTTACATTTTCGTAT<br>TGTTTTCGATTCACTCTATGAATAGTTCTTACTACAATTTTTTTGTCTAAAGAGTAATA<br>CTAGAGATAAACATAAAAAATGTAGAGGTCGAGTTTAGATGCAAGTTCAAGGAGCG<br>AAAGGTGGATGGGTAGGTTATATAGGGATATAGCACAGAGATATATAGCAAAGAGA<br>TACTTTTGAGCAATGTTTGTGGAAGCGGTATTCGCAATATTTTAGTAGCTCGTTACA<br>GTCCGGTGCGTTTTTGGTTTTTTGAAAGTGCGTCTTCAGAGCGCTTTTGGTTTTCAA<br>AAGCGCTCTGAAGTTCCTATACTTTCTAGAGAATAGGAACTTCGGAATAGGAACTTC<br>AAAGCGTTTCCGAAAACGAGCGCTTCCGAAAATGCAACGCGAGCTGCGCACATAC<br>AGCTCACTGTTCACGTCGCACCTATATCTGCGTGTTGCCTGTATATATATATACATG<br>AGAAGAACGGCATAGTGCGTGTTTATGCTTAAATGCGTACTTATATGCGTCTATTTA<br>TGTAGGATGAAAGGTAGTCTAGTACCTCCTGTGATATTATCCCATTCCATGCGGGG<br>TATCGTATGCTTCCTTCAGCACTACCCTTTAGCTGTTCTATATGCTGCCACTCCTCA<br>ATTGGATTAGTCTCATCCTTCAATGCTATCATTTCCTTTGATATTGGATCATACTAAG<br>AAACCATTATTATCATGACATTAACCTATAAAAATAGGCGTATCACGAGGCCCTTTC<br>GTC
pHV011	TCGCGCGAACCCAACTGCACAGAACAAAAACCTGCAGGAAACGAAGATAAATCATG<br>TCGAAAGCTACATATAAGGAACGTGCTGCTACTCATCCTAGTCCTGTTGCTGCCAA<br>GCTATTTAATATCATGCACGAAAAGCAAACAAACTTGTGTGCTTCATTGGATGTTCG<br>TACCACCAAGGAATTACTGGAGTTAGTTGAAGCATTAGGTCCCAAAATTTGTTTACT<br>AAAAACACATGTGGATATCTTGACTGATTTTTCCATGGAGGGCACAGTTAAGCCGC<br>TAAAGGCATTATCCGCCAAGTACAATTTTTTACTCTTCGAAGACAGAAAATTTGCTG<br>ACATTGGTAATACAGTCAAATTGCAGTACTCTGCGGGTGTATACAGAATAGCAGAA<br>TGGGCAGACATTACGAATGCACACGGTGTGGTGGGCCCAGGTATTGTTAGCGGTT<br>TGAAGCAGGCGGCAGAAGAAGTAACAAAGGAACCTAGAGGCCTTTTGATGTTAGC<br>AGAATTGTCATGCAAGGGCTCCCTATCTACTGGAGAATATACTAAGGGTACTGTTG<br>ACATTGCGAAGAGCGACAAAGATTTTGTTATCGGCTTTATTGCTCAAAGAGACATG<br>GGTGGAAGAGATGAAGGTTACGATTGGTTGATTATGACACCCGGTGTGGGTTTAGA<br>TGACAAGGGAGACGCATTGGGTCAACAGTATAGAACCGTGGATGATGTCGTTTCTA<br>CAGGATCTGACATTATTATTGTTGGAAGAGGACTATTTGCAAAGGGAAGGGATGCT<br>AAGGTAGAGGGTGAACGTTACAGAAAAGCAGGCTGGGAAGCATATTTGAGAAGAT<br>GCGGCCAGCAAAACTAAAAAACTGTATTATAAGTAAATGCATGTATACTAAACTCAC<br>AAATTAGAGCTTCAATTTAATTATATCAGTTATTACCCTGTGCTCGAGTCTTTGAAAA<br>GATAATGTATGATTATGCTTTCACTCATATTTATACAGAAACTTGATGTTTTCTTTCG<br>AGTATATACAAGGTGATTACATGTACGTTTGAAGTACAACTCTAGATTTTGTAGTGC<br>CCTCTTGGGCTAGCGGTAAAGGTGCGCATTTTTTCACACCCTACAATGTTCTGTTC<br>AAAAGATTTTGGTCAAACGCTGTAGAAGTGAAAGTTGGTGCGCATGTTTCGGCGTT<br>CGAAACTTCTCCGCAGTGAAAGATAAATGATCGGGTTTTAgAGCTATGCTGTTTTGA<br>ATGGTCCCAAAGGATATGACCCAGAGCCGGGAGAGCTATGCTGTTTTGAATGGTC<br>CCAAAACTTTTTTTGTTTTTTATGTCTGCATAGCTTCAAAATGTTTCTACTCCTTTTTT<br>ACTCTTCCAGATTTTCTCGGACTCCGCGCATCGCCGTACCACTTCAAAACACCCAA<br>GCACAGCATACTAAATTTCCCCTCTTTCTTCCTCTAGGGTGTCGTTAATTACCCGTA<br>CTAAAGGTTTGGAAAAGAAAAAAGAGGCCGCCTCGTTTCTTTTTCTTCGTCGAAAAA<br>GGCAATAAAAATTTTTATCACGTTTCTTTTTCTTGAAAATTTTTTTTTTTGATTTTTTTC<br>TCTTTCGATGACCTCCCATTGATATTTAAGTTAATAAACGGTCTTCAATTTCTCAAGT<br>TTCAGTTTCATTTTTCTTGTTCTATTACAACTTTTTTTACTTCTTGCTCATTAGAAAGA<br>AAGCATAGCAATCTAATCTAAGTTTTCTAGATGGATTATAAAGATGACGATGACAAA<br>CCTCCAAAAAAGAAGAGAAAGGTCGATAAGAAATACTCAATAGGCTTAGATATCGG<br>CACAAATAGCGTCGGATGGGCGGTGATCACTGATGAATATAAGGTTCCGTCTAAAA<br>AGTTCAAGGTTCTGGGAAATACAGACCGCCACAGTATCAAAAAAAATCTTATAGGG<br>GCTCTTTTATTTGACAGTGGAGAGACAGCGGAAGCGACTCGTCTCAAACGGACAG<br>CTCGTAGAAGGTATACACGTCGGAAGAATCGTATTTGTTATCTACAGGAGATTTTTT<br>CAAATGAGATGGCGAAAGTAGATGATAGTTTCTTTCATCGACTTGAAGAGTCTTTTT<br>TGGTGGAAGAAGACAAGAAGCATGAACGTCATCCTATTTTTGGAAATATAGTAGAT<br>GAAGTTGCTTATCATGAGAAATATCCAACTATCTATCATCTGCGAAAAAAATTGGTA<br>GATTCTACTTATAAAGCGGATTTGCGCTTAATCTATTTGGCCTTAGCGCATATGATT<br>AAGTTTCGTGGTCATTTTTTGATTGAGGGAGATTTAAATCCTGATAATAGTGATGTG<br>GACAAACTATTTATCCAGTTGGTACAAACCTACAATCAATTATTTGAAGAAAACCCT<br>ATTAACGCAAGTGGAGTAGATGCTAAAGCGATTCTTTCTGCACGATTGAGTAAATCA<br>AGACGATTAGAAAATCTCATTGCTCAGCTCCCCGGTGAGAAGAAAAATGGCTTATT<br>TGGGAATCTCATTGCTTTGTCATTGGGTTTGACCCCTAATTTTAAATCAAATTTTGAT<br>TTGGCAGAAGATGCTAAATTACAGCTTTCAAAAGATACTTACGATGATGATTTAGAT

	AATTTATTGGCGCAAATTGGAGATCAATATGCTGATTTGTTTTTGGCAGCTAAGAAT<br>TTATCAGATGCTATTTTACTTTCAGATATCCTAAGAGTAAATACTGAAATAACTAAGG<br>CTCCCCTATCAGCTTCAATGATTAAACGCTACGATGAACATCATCAAGACTTGACTC<br>TTTTAAAAGCTTTAGTTCGACAACAACTTCCAGAAAAGTATAAAGAAATCTTTTTTGA<br>TCAATCAAAAAACGGATATGCAGGTTATATTGATGGGGGAGCTAGCCAAGAAGAAT<br>TTTATAAATTTATCAAACCAATTTTAGAAAAAATGGATGGTACTGAGGAATTATTGGT<br>GAAACTAAATCGTGAAGATTTGCTGCGCAAGCAACGGACCTTTGACAACGGCTCTA<br>TTACCCATCAAATTCACTTGGGTGAGCTGCATGCTATTTTGAGAAGACAAGAAGACT<br>TTTATCCATTTTTAAAAGACAATCGTGAGAAGATTGAAAAAATCTTGACTTTTCGAAT<br>TCCTTATTATGTTGGTCCATTGGCGCGTGGCAATAGTCGTTTTGCATGGATGACTC<br>GGAAGTCTGAAGAAACAATTACCCCATGGAATTTTGAAGAAGTTGTCGATAAAGGT<br>GCTTCAGCTCAATCATTTATTGAACGCATGACAAACTTTGATAAAAATCTTCCAAAT<br>GAAAAAGTACTACCAAAACATAGTTTGCTTTATGAGTATTTTACGGTTTATAACGAAT<br>TGACAAAGGTCAAATATGTTACTGAAGGAATGCGAAAACCAGCATTTCTTTCAGGT<br>GAACAGAAGAAAGCCATTGTTGATTTACTCTTCAAAACAAATCGAAAAGTAACCGTT<br>AAGCAATTAAAAGAAGATTATTTCAAAAAAATAGAATGTTTTGATAGTGTTGAAATTT<br>CAGGAGTTGAAGATAGATTTAATGCTTCATTAGGTACCTACCATGATTTGCTAAAAA<br>TTATTAAAGATAAAGATTTTTTGGATAATGAAGAAAATGAAGATATCTTAGAGGATAT<br>TGTTTTAACATTGACCTTATTTGAAGATAGGGAGATGATTGAGGAAAGACTTAAAAC<br>ATATGCTCACCTCTTTGATGATAAGGTGATGAAACAGCTTAAACGTCGCCGTTATAC<br>TGGTTGGGGACGTTTGTCTCGAAAATTGATTAATGGTATTAGGGATAAGCAATCTG<br>GCAAAACAATATTAGATTTTTTGAAATCAGATGGTTTTGCCAATCGCAATTTTATGCA<br>GCTGATCCATGATGATAGTTTGACATTTAAAGAAGACATTCAAAAAGCACAAGTGTC<br>TGGACAAGGCGATAGTTTACATGAACATATTGCAAATTTAGCTGGTAGCCCTGCTAT<br>TAAAAAAGGTATTTTACAGACTGTAAAAGTTGTTGATGAATTGGTCAAAGTAATGGG<br>GCGGCATAAGCCAGAAAATATCGTTATTGAAATGGCACGTGAAAATCAGACAACTC<br>AAAAGGGCCAGAAAAATTCGCGAGAGCGTATGAAACGAATCGAAGAAGGTATCAAA<br>GAATTAGGAAGTCAGATTCTTAAAGAGCATCCTGTTGAAAATACTCAATTGCAAAAT<br>GAAAAGCTCTATCTCTATTATCTCCAAAATGGAAGAGACATGTATGTGGACCAAGAA<br>TTAGATATTAATCGTTTAAGTGATTATGATGTCGATCACATTGTTCCACAAAGTTTCC<br>TTAAAGACGATTCAATAGACAATAAGGTCTTAACGCGTTCTGATAAAAATCGTGGTA<br>AATCGGATAACGTTCCAAGTGAAGAAGTAGTCAAAAAGATGAAAAACTATTGGAGA<br>CAACTTCTAAACGCCAAGTTAATCACTCAACGTAAGTTTGATAATTTAACGAAAGCT<br>GAACGTGGAGGTTTGAGTGAACTTGATAAAGCTGGTTTTATCAAACGCCAATTGGT<br>TGAAACTCGCCAAATCACTAAGCATGTGGCACAAATTTTGGATAGTCGCATGAATA<br>CTAAATACGATGAAAATGATAAACTTATTCGAGAGGTTAAAGTGATTACCTTAAAAT<br>CTAAATTAGTTTCTGACTTCCGAAAAGATTTCCAATTCTATAAAGTACGTGAGATTAA<br>CAATTACCATCATGCCCATGATGCGTATCTAAATGCCGTCGTTGGAACTGCTTTGAT<br>TAAGAAATATCCAAAACTTGAATCGGAGTTTGTCTATGGTGATTATAAAGTTTATGAT<br>GTTCGTAAAATGATTGCTAAGTCTGAGCAAGAAATAGGCAAAGCAACCGCAAAATA<br>TTTCTTTTACTCTAATATCATGAACTTCTTCAAAACAGAAATTACACTTGCAAATGGA<br>GAGATTCGCAAACGCCCTCTAATCGAAACTAATGGGGAAACTGGAGAAATTGTCTG<br>GGATAAAGGGCGAGATTTTGCCACAGTGCGCAAAGTATTGTCCATGCCCCAAGTCA<br>ATATTGTCAAGAAAACAGAAGTACAGACAGGCGGATTCTCCAAGGAGTCAATTTTA<br>CCAAAAAGAAATTCGGACAAGCTTATTGCTCGTAAAAAAGACTGGGATCCAAAAAA<br>ATATGGTGGTTTTGATAGTCCAACGGTAGCTTATTCAGTCCTAGTGGTTGCTAAGGT<br>GGAAAAAGGGAAATCGAAGAAGTTAAAATCCGTTAAAGAGTTACTAGGGATCACAA<br>TTATGGAAAGAAGTTCCTTTGAAAAAAATCCGATTGACTTTTTAGAAGCTAAAGGAT<br>ATAAGGAAGTTAAAAAAGACTTAATCATTAAACTACCTAAATATAGTCTTTTTGAGTT<br>AGAAAACGGTCGTAAACGGATGCTGGCTAGTGCCGGAGAATTACAAAAAGGAAAT<br>GAGCTGGCTCTGCCAAGCAAATATGTGAATTTTTTATATTTAGCTAGTCATTATGAA<br>AAGTTGAAGGGTAGTCCAGAAGATAACGAACAAAAACAATTGTTTGTGGAGCAGCA<br>TAAGCATTATTTAGATGAGATTATTGAGCAAATCAGTGAATTTTCTAAGCGTGTTATT<br>TTAGCAGATGCCAATTTAGATAAAGTTCTTAGTGCATATAACAAACATAGAGACAAA<br>CCAATACGTGAACAAGCAGAAAATATTATTCATTTATTTACGTTGACGAATCTTGGA<br>GCTCCCGCTGCTTTTAAATATTTTGATACAACAATTGATCGTAAACGATATACGTCT<br>ACAAAAGAAGTTTTAGATGCCACTCTTATCCATCAATCCATCACTGGTCTTTATGAA<br>ACACGCATTGATTTGAGTCAGCTAGGAGGTGACCCTCCAAAAAAGAAGAGAAAGGT<br>CTGAGCGGATCTCTTATGTCTTTACGATTTATAGTTTTCATTATCAAGTATGCCTATA<br>TTAGTATATAGCATCTTTAGATGACAGTGTTCGAAGTTTCACGAATAAAAGATAATAT<br>TCTACTTTTTGCTCCCACCGCGTTTGCTAGCACGAGTGAACACCATCCCTCGCCTG

	TGAGTTGTACCCATTCCTCTAAACTGTAGACATGGTAGCTTCAGCAGTGTTCGTTAT<br>GTACGGCATCCTCCAACAAACAGTCGGTTATAGTTTGTCCTGCTCCTCTGAATCGA<br>GTCCCTCGATATTTCTCATACTAGTTCTAGAGATCTGCCAATTGAACATAACATGGT<br>AGTTACATATACTAGTAATATGGTTCGGCACACATTAAAAGTATAAAAACTATCTGAA<br>TTACGAATTACATATATTGGTCATAAAAATCAATCAATCATCGTGTGTTTTATATGTC<br>TCTTATCTAAGTATAAGAATATCCATAGTTAATATTCACTTACGCTACCTTTTAACCT<br>GTAATCATTGTCAACAGGATATGTTAACGACCCACATTGATAAACGCTAGTATTTCT<br>TTTTCCTCTTCTTATTGGCCGGCTGTCTCTATACTCCCCTATAGTCTGTTTCTTTTCG<br>TTTCGATTGTTTTACGTTTGAGGCCTCGTGGCGCACATGGTACGCTGTGGTGCTCG<br>CGGCTGGGAACGAAACTCTGGGAGCTGCGATTGGCAGGAACCATTCAAAACAGCA<br>TAGCAAGTTAAAATAAGGCTAGTCCGTTATCAACTTGAAAAAGTGGCACCGAGTCG<br>GTGCTTTTTTTCCATATCCAACTTCCAATTTAATCTTTCTTTTTTAATTTTCACTTATTT<br>GCGATACAGAAAGACCCTGCAGGCATGCAAGCTTGGCGTAATCATGGTCATAGCT<br>GTTTCCTGTGTGAAATTGTTATCCGCTCACAATTCCACACAACATACGAGCCGGAA<br>GCATAAAGTGTAAAGCCTGGGGTGCCTAATGAGTGAGCTAACTCACATTAATTGCG<br>TTGCGCTCACTGCCCGCTTTCCAGTCGGGAAACCTGTCGTGCCAGCTGCATTAATG<br>AATCGGCCAACGCGCGGGGAGAGGCGGTTTGCGTATTGGGCGCTCTTCCGCTTC<br>CTCGCTCACTGACTCGCTGCGCTCGGTCGTTCGGCTGCGGCGAGCGGTATCAGCT<br>CACTCAAAGGCGGTAATACGGTTATCCACAGAATCAGGGGATAACGCAGGAAAGA<br>ACATGTGAGCAAAAGGCCAGCAAAAGGCCAGGAACCGTAAAAAGGCCGCGTTGCT<br>GGCGTTTTTCCATAGGCTCCGCCCCCCTGACGAGCATCACAAAAATCGACGCTCAA<br>GTCAGAGGTGGCGAAACCCGACAGGACTATAAAGATACCAGGCGTTTCCCCCTGG<br>AAGCTCCCTCGTGCGCTCTCCTGTTCCGACCCTGCCGCTTACCGGATACCTGTCC<br>GCCTTTCTCCCTTCGGGAAGCGTGGCGCTTTCTCATAGCTCACGCTGTAGGTATCT<br>CAGTTCGGTGTAGGTCGTTCGCTCCAAGCTGGGCTGTGTGCACGAACCCCCCGTT<br>CAGCCCGACCGCTGCGCCTTATCCGGTAACTATCGTCTTGAGTCCAACCCGGTAA<br>GACACGACTTATCGCCACTGGCAGCAGCCACTGGTAACAGGATTAGCAGAGCGAG<br>GTATGTAGGCGGTGCTACAGAGTTCTTGAAGTGGTGGCCTAACTACGGCTACACTA<br>GAAGAACAGTATTTGGTATCTGCGCTCTGCTGAAGCCAGTTACCTTCGGAAAAAGA<br>GTTGGTAGCTCTTGATCCGGCAAACAAACCACCGCTGGTAGCGGTGGTTTTTTTGT<br>TTGCAAGCAGCAGATTACGCGCAGAAAAAAAGGATCTCAAGAAGATCCTTTGATCT<br>TTTCTACGGGGTCTGACGCTCAGTGGAACGAAAACTCACGTTAAGGGATTTTGGTC<br>ATGAGATTATCAAAAAGGATCTTCACCTAGATCCTTTTAAATTAAAAATGAAGTTTTA<br>AATCAATCTAAAGTATATATGAGTAAACTTGGTCTGACAGTTACCAATGCTTAATCA<br>GTGAGGCACCTATCTCAGCGATCTGTCTATTTCGTTCATCCATAGTTGCCTGACTC<br>CCCGTCGTGTAGATAACTACGATACGGGAGGGCTTACCATCTGGCCCCAGTGCTG<br>CAATGATACCGCGGCTCCCACGCTCACCGGCTCCAGATTTATCAGCAATAAACCAG<br>CCAGCCGGAAGGGCCGAGCGCAGAAGTGGTCCTGCAACTTTATCCGCCTCCATCC<br>AGTCTATTAATTGTTGCCGGGAAGCTAGAGTAAGTAGTTCGCCAGTTAATAGTTTGC<br>GCAACGTTGTTGCCATTGCTACAGGCATCGTGGTGTCACGCTCGTCGTTTGGTATG<br>GCTTCATTCAGCTCCGGTTCCCAACGATCAAGGCGAGTTACATGATCCCCCATGTT<br>GTGCAAAAAAGCGGTTAGCTCCTTCGGTCCTCCGATCGTTGTCAGAAGTAAGTTGG<br>CCGCAGTGTTATCACTCATGGTTATGGCAGCACTGCATAATTCTCTTACTGTCATGC<br>CATCCGTAAGATGCTTTTCTGTGACTGGTGAGTACTCAACCAAGTCATTCTGAGAAT<br>AGTGTATGCGGCGACCGAGTTGCTCTTGCCCGGCGTCAATACGGGATAATACCGC<br>GCCACATAGCAGAACTTTAAAAGTGCTCATCATTGGAAAACGTTCTTCGGGGCGAA<br>AACTCTCAAGGATCTTACCGCTGTTGAGATCCAGTTCGATGTAACCCACTCGTGCA<br>CCCAACTGATCTTCAGCATCTTTTACTTTCACCAGCGTTTCTGGGTGAGCAAAAACA<br>GGAAGGCAAAATGCCGCAAAAAAGGGAATAAGGGCGACACGGAAATGTTGAATAC<br>TCATACTCTTCCTTTTTCAATATTATTGAAGCATTTATCAGGGTTATTGTCTCATGAG<br>CGGATACATATTTGAATGTATTTAGAAAAATAAACAAATAGGGGTTCCGCGCACATT<br>TCCCCGAAAAGTGCCACCTGAACGAAGCATCTGTGCTTCATTTTGTAGAACAAAAA<br>TGCAACGCGAGAGCGCTAATTTTTCAAACAAAGAATCTGAGCTGCATTTTTACAGAA<br>CAGAAATGCAACGCGAAAGCGCTATTTTACCAACGAAGAATCTGTGCTTCATTTTTG<br>TAAAACAAAAATGCAACGCGAGAGCGCTAATTTTTCAAACAAAGAATCTGAGCTGC<br>ATTTTTACAGAACAGAAATGCAACGCGAGAGCGCTATTTTACCAACAAAGAATCTAT<br>ACTTCTTTTTTGTTCTACAAAAATGCATCCCGAGAGCGCTATTTTTCTAACAAAGCAT<br>CTTAGATTACTTTTTTTCTCCTTTGTGCGCTCTATAATGCAGTCTCTTGATAACTTTT<br>TGCACTGTAGGTCCGTTAAGGTTAGAAGAAGGCTACTTTGGTGTCTATTTTCTCTTC<br>CATAAAAAAAGCCTGACTCCACTTCCCGCGTTTACTGATTACTAGCGAAGCTGCGG<br>GTGCATTTTTTCAAGATAAAGGCATCCCCGATTATATTCTATACCGATGTGGATTGC

	GCATACTTTGTGAACAGAAAGTGATAGCGTTGATGATTCTTCATTGGTCAGAAAATT<br>ATGAACGGTTTCTTCTATTTTGTCTCTATATACTACGTATAGGAAATGTTTACATTTT<br>CGTATTGTTTTCGATTCACTCTATGAATAGTTCTTACTACAATTTTTTTGTCTAAAGA<br>GTAATACTAGAGATAAACATAAAAAATGTAGAGGTCGAGTTTAGATGCAAGTTCAAG<br>GAGCGAAAGGTGGATGGGTAGGTTATATAGGGATATAGCACAGAGATATATAGCAA<br>AGAGATACTTTTGAGCAATGTTTGTGGAAGCGGTATTCGCAATATTTTAGTAGCTCG<br>TTACAGTCCGGTGCGTTTTTGGTTTTTTGAAAGTGCGTCTTCAGAGCGCTTTTGGTT<br>TTCAAAAGCGCTCTGAAGTTCCTATACTTTCTAGAGAATAGGAACTTCGGAATAGGA<br>ACTTCAAAGCGTTTCCGAAAACGAGCGCTTCCGAAAATGCAACGCGAGCTGCGCA<br>CATACAGCTCACTGTTCACGTCGCACCTATATCTGCGTGTTGCCTGTATATATATAT<br>ACATGAGAAGAACGGCATAGTGCGTGTTTATGCTTAAATGCGTACTTATATGCGTCT<br>ATTTATGTAGGATGAAAGGTAGTCTAGTACCTCCTGTGATATTATCCCATTCCATGC<br>GGGGTATCGTATGCTTCCTTCAGCACTACCCTTTAGCTGTTCTATATGCTGCCACTC<br>CTCAATTGGATTAGTCTCATCCTTCAATGCTATCATTTCCTTTGATATTGGATCATAC<br>TAAGAAACCATTATTATCATGACATTAACCTATAAAAATAGGCGTATCACGAGGCCC<br>TTTCGTC
pHV012	TCGCGCGAACCCAACTGCACAGAACAAAAACCTGCAGGAAACGAAGATAAATCATG<br>TCGAAAGCTACATATAAGGAACGTGCTGCTACTCATCCTAGTCCTGTTGCTGCCAA<br>GCTATTTAATATCATGCACGAAAAGCAAACAAACTTGTGTGCTTCATTGGATGTTCG<br>TACCACCAAGGAATTACTGGAGTTAGTTGAAGCATTAGGTCCCAAAATTTGTTTACT<br>AAAAACACATGTGGATATCTTGACTGATTTTTCCATGGAGGGCACAGTTAAGCCGC<br>TAAAGGCATTATCCGCCAAGTACAATTTTTTACTCTTCGAAGACAGAAAATTTGCTG<br>ACATTGGTAATACAGTCAAATTGCAGTACTCTGCGGGTGTATACAGAATAGCAGAA<br>TGGGCAGACATTACGAATGCACACGGTGTGGTGGGCCCAGGTATTGTTAGCGGTT<br>TGAAGCAGGCGGCAGAAGAAGTAACAAAGGAACCTAGAGGCCTTTTGATGTTAGC<br>AGAATTGTCATGCAAGGGCTCCCTATCTACTGGAGAATATACTAAGGGTACTGTTG<br>ACATTGCGAAGAGCGACAAAGATTTTGTTATCGGCTTTATTGCTCAAAGAGACATG<br>GGTGGAAGAGATGAAGGTTACGATTGGTTGATTATGACACCCGGTGTGGGTTTAGA<br>TGACAAGGGAGACGCATTGGGTCAACAGTATAGAACCGTGGATGATGTCGTTTCTA<br>CAGGATCTGACATTATTATTGTTGGAAGAGGACTATTTGCAAAGGGAAGGGATGCT<br>AAGGTAGAGGGTGAACGTTACAGAAAAGCAGGCTGGGAAGCATATTTGAGAAGAT<br>GCGGCCAGCAAAACTAAAAAACTGTATTATAAGTAAATGCATGTATACTAAACTCAC<br>AAATTAGAGCTTCAATTTAATTATATCAGTTATTACCCTGTGCTCGAGTCTTTGAAAA<br>GATAATGTATGATTATGCTTTCACTCATATTTATACAGAAACTTGATGTTTTCTTTCG<br>AGTATATACAAGGTGATTACATGTACGTTTGAAGTACAACTCTAGATTTTGTAGTGC<br>CCTCTTGGGCTAGCGGTAAAGGTGCGCATTTTTTCACACCCTACAATGTTCTGTTC<br>AAAAGATTTTGGTCAAACGCTGTAGAAGTGAAAGTTGGTGCGCATGTTTCGGCGTT<br>CGAAACTTCTCCGCAGTGAAAGATAAATGATCGGGTTTTAgAGCTATGCTGTTTTGA<br>ATGGTCCCAAACGTGGCTATACAAATAGGACAGAGCTATGCTGTTTTGAATGGTCC<br>CAAAACTTTTTTTGTTTTTTATGTCTGCATAGCTTCAAAATGTTTCTACTCCTTTTTTA<br>CTCTTCCAGATTTTCTCGGACTCCGCGCATCGCCGTACCACTTCAAAACACCCAAG<br>CACAGCATACTAAATTTCCCCTCTTTCTTCCTCTAGGGTGTCGTTAATTACCCGTAC<br>TAAAGGTTTGGAAAAGAAAAAAGAGGCCGCCTCGTTTCTTTTTCTTCGTCGAAAAAG<br>GCAATAAAAATTTTTATCACGTTTCTTTTTCTTGAAAATTTTTTTTTTTGATTTTTTTCT<br>CTTTCGATGACCTCCCATTGATATTTAAGTTAATAAACGGTCTTCAATTTCTCAAGTT<br>TCAGTTTCATTTTTCTTGTTCTATTACAACTTTTTTTACTTCTTGCTCATTAGAAAGAA<br>AGCATAGCAATCTAATCTAAGTTTTCTAGATGGATTATAAAGATGACGATGACAAAC<br>CTCCAAAAAAGAAGAGAAAGGTCGATAAGAAATACTCAATAGGCTTAGATATCGGC<br>ACAAATAGCGTCGGATGGGCGGTGATCACTGATGAATATAAGGTTCCGTCTAAAAA<br>GTTCAAGGTTCTGGGAAATACAGACCGCCACAGTATCAAAAAAAATCTTATAGGGG<br>CTCTTTTATTTGACAGTGGAGAGACAGCGGAAGCGACTCGTCTCAAACGGACAGCT<br>CGTAGAAGGTATACACGTCGGAAGAATCGTATTTGTTATCTACAGGAGATTTTTTCA<br>AATGAGATGGCGAAAGTAGATGATAGTTTCTTTCATCGACTTGAAGAGTCTTTTTTG<br>GTGGAAGAAGACAAGAAGCATGAACGTCATCCTATTTTTGGAAATATAGTAGATGA<br>AGTTGCTTATCATGAGAAATATCCAACTATCTATCATCTGCGAAAAAAATTGGTAGA<br>TTCTACTTATAAAGCGGATTTGCGCTTAATCTATTTGGCCTTAGCGCATATGATTAA<br>GTTTCGTGGTCATTTTTTGATTGAGGGAGATTTAAATCCTGATAATAGTGATGTGGA<br>CAAACTATTTATCCAGTTGGTACAAACCTACAATCAATTATTTGAAGAAAACCCTATT<br>AACGCAAGTGGAGTAGATGCTAAAGCGATTCTTTCTGCACGATTGAGTAAATCAAG<br>ACGATTAGAAAATCTCATTGCTCAGCTCCCCGGTGAGAAGAAAAATGGCTTATTTG<br>GGAATCTCATTGCTTTGTCATTGGGTTTGACCCCTAATTTTAAATCAAATTTTGATTT

	GGCAGAAGATGCTAAATTACAGCTTTCAAAAGATACTTACGATGATGATTTAGATAA
	TTTATTGGCGCAAATTGGAGATCAATATGCTGATTTGTTTTTGGCAGCTAAGAATTT
	ATCAGATGCTATTTTACTTTCAGATATCCTAAGAGTAAATACTGAAATAACTAAGGCT
	CCCCTATCAGCTTCAATGATTAAACGCTACGATGAACATCATCAAGACTTGACTCTT
	TTAAAAGCTTTAGTTCGACAACAACTTCCAGAAAAGTATAAAGAAATCTTTTTTGATC
	AATCAAAAAACGGATATGCAGGTTATATTGATGGGGGAGCTAGCCAAGAAGAATTT
	TATAAATTTATCAAACCAATTTTAGAAAAAATGGATGGTACTGAGGAATTATTGGTGA
	AACTAAATCGTGAAGATTTGCTGCGCAAGCAACGGACCTTTGACAACGGCTCTATT
	ACCCATCAAATTCACTTGGGTGAGCTGCATGCTATTTTGAGAAGACAAGAAGACTTT
	TATCCATTTTTAAAAGACAATCGTGAGAAGATTGAAAAAATCTTGACTTTTCGAATTC
	CTTATTATGTTGGTCCATTGGCGCGTGGCAATAGTCGTTTTGCATGGATGACTCGG
	AAGTCTGAAGAAACAATTACCCCATGGAATTTTGAAGAAGTTGTCGATAAAGGTGCT
	TCAGCTCAATCATTTATTGAACGCATGACAAACTTTGATAAAAATCTTCCAAATGAAA
	AAGTACTACCAAAACATAGTTTGCTTTATGAGTATTTTACGGTTTATAACGAATTGAC
	AAAGGTCAAATATGTTACTGAAGGAATGCGAAAACCAGCATTTCTTTCAGGTGAACA
	GAAGAAAGCCATTGTTGATTTACTCTTCAAAACAAATCGAAAAGTAACCGTTAAGCA
	ATTAAAAGAAGATTATTTCAAAAAAATAGAATGTTTTGATAGTGTTGAAATTTCAGGA
	GTTGAAGATAGATTTAATGCTTCATTAGGTACCTACCATGATTTGCTAAAAATTATTA
	AAGATAAAGATTTTTTGGATAATGAAGAAAATGAAGATATCTTAGAGGATATTGTTTT
	AACATTGACCTTATTTGAAGATAGGGAGATGATTGAGGAAAGACTTAAAACATATGC
	TCACCTCTTTGATGATAAGGTGATGAAACAGCTTAAACGTCGCCGTTATACTGGTTG
	GGGACGTTTGTCTCGAAAATTGATTAATGGTATTAGGGATAAGCAATCTGGCAAAA
	CAATATTAGATTTTTTGAAATCAGATGGTTTTGCCAATCGCAATTTTATGCAGCTGAT
	CCATGATGATAGTTTGACATTTAAAGAAGACATTCAAAAAGCACAAGTGTCTGGACA
	AGGCGATAGTTTACATGAACATATTGCAAATTTAGCTGGTAGCCCTGCTATTAAAAA
	AGGTATTTTACAGACTGTAAAAGTTGTTGATGAATTGGTCAAAGTAATGGGGCGGC
	ATAAGCCAGAAAATATCGTTATTGAAATGGCACGTGAAAATCAGACAACTCAAAAG
	GGCCAGAAAAATTCGCGAGAGCGTATGAAACGAATCGAAGAAGGTATCAAAGAATT
	AGGAAGTCAGATTCTTAAAGAGCATCCTGTTGAAAATACTCAATTGCAAAATGAAAA
	GCTCTATCTCTATTATCTCCAAAATGGAAGAGACATGTATGTGGACCAAGAATTAGA
	TATTAATCGTTTAAGTGATTATGATGTCGATCACATTGTTCCACAAAGTTTCCTTAAA
	GACGATTCAATAGACAATAAGGTCTTAACGCGTTCTGATAAAAATCGTGGTAAATCG
	GATAACGTTCCAAGTGAAGAAGTAGTCAAAAAGATGAAAAACTATTGGAGACAACTT
	CTAAACGCCAAGTTAATCACTCAACGTAAGTTTGATAATTTAACGAAAGCTGAACGT
	GGAGGTTTGAGTGAACTTGATAAAGCTGGTTTTATCAAACGCCAATTGGTTGAAACT
	CGCCAAATCACTAAGCATGTGGCACAAATTTTGGATAGTCGCATGAATACTAAATAC
	GATGAAAATGATAAACTTATTCGAGAGGTTAAAGTGATTACCTTAAAATCTAAATTAG
	TTTCTGACTTCCGAAAAGATTTCCAATTCTATAAAGTACGTGAGATTAACAATTACCA
	TCATGCCCATGATGCGTATCTAAATGCCGTCGTTGGAACTGCTTTGATTAAGAAATA
	TCCAAAACTTGAATCGGAGTTTGTCTATGGTGATTATAAAGTTTATGATGTTCGTAA
	AATGATTGCTAAGTCTGAGCAAGAAATAGGCAAAGCAACCGCAAAATATTTCTTTTA
	CTCTAATATCATGAACTTCTTCAAAACAGAAATTACACTTGCAAATGGAGAGATTCG
	CAAACGCCCTCTAATCGAAACTAATGGGGAAACTGGAGAAATTGTCTGGGATAAAG
	GGCGAGATTTTGCCACAGTGCGCAAAGTATTGTCCATGCCCCAAGTCAATATTGTC
	AAGAAAACAGAAGTACAGACAGGCGGATTCTCCAAGGAGTCAATTTTACCAAAAAG
	AAATTCGGACAAGCTTATTGCTCGTAAAAAAGACTGGGATCCAAAAAAATATGGTG
	GTTTTGATAGTCCAACGGTAGCTTATTCAGTCCTAGTGGTTGCTAAGGTGGAAAAA
	GGGAAATCGAAGAAGTTAAAATCCGTTAAAGAGTTACTAGGGATCACAATTATGGA
	AAGAAGTTCCTTTGAAAAAAATCCGATTGACTTTTTAGAAGCTAAAGGATATAAGGA
	AGTTAAAAAAGACTTAATCATTAAACTACCTAAATATAGTCTTTTTGAGTTAGAAAAC
	GGTCGTAAACGGATGCTGGCTAGTGCCGGAGAATTACAAAAAGGAAATGAGCTGG
	CTCTGCCAAGCAAATATGTGAATTTTTTATATTTAGCTAGTCATTATGAAAAGTTGAA
	GGGTAGTCCAGAAGATAACGAACAAAAACAATTGTTTGTGGAGCAGCATAAGCATT
	ATTTAGATGAGATTATTGAGCAAATCAGTGAATTTTCTAAGCGTGTTATTTTAGCAGA
	TGCCAATTTAGATAAAGTTCTTAGTGCATATAACAAACATAGAGACAAACCAATACG
	TGAACAAGCAGAAAATATTATTCATTTATTTACGTTGACGAATCTTGGAGCTCCCGC
	TGCTTTTAAATATTTTGATACAACAATTGATCGTAAACGATATACGTCTACAAAAGAA
	GTTTTAGATGCCACTCTTATCCATCAATCCATCACTGGTCTTTATGAAACACGCATT
	GATTTGAGTCAGCTAGGAGGTGACCCTCCAAAAAAGAAGAGAAAGGTCTGAGCGG
	ATCTCTTATGTCTTTACGATTTATAGTTTTCATTATCAAGTATGCCTATATTAGTATAT
	AGCATCTTTAGATGACAGTGTTCGAAGTTTCACGAATAAAAGATAATATTCTACTTTT

	TGCTCCCACCGCGTTTGCTAGCACGAGTGAACACCATCCCTCGCCTGTGAGTTGTA<br>CCCATTCCTCTAAACTGTAGACATGGTAGCTTCAGCAGTGTTCGTTATGTACGGCA<br>TCCTCCAACAAACAGTCGGTTATAGTTTGTCCTGCTCCTCTGAATCGAGTCCCTCG<br>ATATTTCTCATACTAGTTCTAGAGATCTGCCAATTGAACATAACATGGTAGTTACATA<br>TACTAGTAATATGGTTCGGCACACATTAAAAGTATAAAAACTATCTGAATTACGAATT<br>ACATATATTGGTCATAAAAATCAATCAATCATCGTGTGTTTTATATGTCTCTTATCTA<br>AGTATAAGAATATCCATAGTTAATATTCACTTACGCTACCTTTTAACCTGTAATCATT<br>GTCAACAGGATATGTTAACGACCCACATTGATAAACGCTAGTATTTCTTTTTCCTCT<br>TCTTATTGGCCGGCTGTCTCTATACTCCCCTATAGTCTGTTTCTTTTCGTTTCGATT<br>GTTTTACGTTTGAGGCCTCGTGGCGCACATGGTACGCTGTGGTGCTCGCGGCTGG<br>GAACGAAACTCTGGGAGCTGCGATTGGCAGGAACCATTCAAAACAGCATAGCAAG<br>TTAAAATAAGGCTAGTCCGTTATCAACTTGAAAAAGTGGCACCGAGTCGGTGCTTTT<br>TTTCCATATCCAACTTCCAATTTAATCTTTCTTTTTTAATTTTCACTTATTTGCGATAC<br>AGAAAGACCCTGCAGGCATGCAAGCTTGGCGTAATCATGGTCATAGCTGTTTCCTG<br>TGTGAAATTGTTATCCGCTCACAATTCCACACAACATACGAGCCGGAAGCATAAAG<br>TGTAAAGCCTGGGGTGCCTAATGAGTGAGCTAACTCACATTAATTGCGTTGCGCTC<br>ACTGCCCGCTTTCCAGTCGGGAAACCTGTCGTGCCAGCTGCATTAATGAATCGGC<br>CAACGCGCGGGGAGAGGCGGTTTGCGTATTGGGCGCTCTTCCGCTTCCTCGCTCA<br>CTGACTCGCTGCGCTCGGTCGTTCGGCTGCGGCGAGCGGTATCAGCTCACTCAAA<br>GGCGGTAATACGGTTATCCACAGAATCAGGGGATAACGCAGGAAAGAACATGTGA<br>GCAAAAGGCCAGCAAAAGGCCAGGAACCGTAAAAAGGCCGCGTTGCTGGCGTTTT<br>TCCATAGGCTCCGCCCCCCTGACGAGCATCACAAAAATCGACGCTCAAGTCAGAG<br>GTGGCGAAACCCGACAGGACTATAAAGATACCAGGCGTTTCCCCCTGGAAGCTCC<br>CTCGTGCGCTCTCCTGTTCCGACCCTGCCGCTTACCGGATACCTGTCCGCCTTTCT<br>CCCTTCGGGAAGCGTGGCGCTTTCTCATAGCTCACGCTGTAGGTATCTCAGTTCG<br>GTGTAGGTCGTTCGCTCCAAGCTGGGCTGTGTGCACGAACCCCCCGTTCAGCCCG<br>ACCGCTGCGCCTTATCCGGTAACTATCGTCTTGAGTCCAACCCGGTAAGACACGAC<br>TTATCGCCACTGGCAGCAGCCACTGGTAACAGGATTAGCAGAGCGAGGTATGTAG<br>GCGGTGCTACAGAGTTCTTGAAGTGGTGGCCTAACTACGGCTACACTAGAAGAAC<br>AGTATTTGGTATCTGCGCTCTGCTGAAGCCAGTTACCTTCGGAAAAAGAGTTGGTA<br>GCTCTTGATCCGGCAAACAAACCACCGCTGGTAGCGGTGGTTTTTTTGTTTGCAAG<br>CAGCAGATTACGCGCAGAAAAAAAGGATCTCAAGAAGATCCTTTGATCTTTTCTAC<br>GGGGTCTGACGCTCAGTGGAACGAAAACTCACGTTAAGGGATTTTGGTCATGAGAT<br>TATCAAAAAGGATCTTCACCTAGATCCTTTTAAATTAAAAATGAAGTTTTAAATCAAT<br>CTAAAGTATATATGAGTAAACTTGGTCTGACAGTTACCAATGCTTAATCAGTGAGGC<br>ACCTATCTCAGCGATCTGTCTATTTCGTTCATCCATAGTTGCCTGACTCCCCGTCGT<br>GTAGATAACTACGATACGGGAGGGCTTACCATCTGGCCCCAGTGCTGCAATGATA<br>CCGCGGCTCCCACGCTCACCGGCTCCAGATTTATCAGCAATAAACCAGCCAGCCG<br>GAAGGGCCGAGCGCAGAAGTGGTCCTGCAACTTTATCCGCCTCCATCCAGTCTAT<br>TAATTGTTGCCGGGAAGCTAGAGTAAGTAGTTCGCCAGTTAATAGTTTGCGCAACG<br>TTGTTGCCATTGCTACAGGCATCGTGGTGTCACGCTCGTCGTTTGGTATGGCTTCA<br>TTCAGCTCCGGTTCCCAACGATCAAGGCGAGTTACATGATCCCCCATGTTGTGCAA<br>AAAAGCGGTTAGCTCCTTCGGTCCTCCGATCGTTGTCAGAAGTAAGTTGGCCGCA<br>GTGTTATCACTCATGGTTATGGCAGCACTGCATAATTCTCTTACTGTCATGCCATCC<br>GTAAGATGCTTTTCTGTGACTGGTGAGTACTCAACCAAGTCATTCTGAGAATAGTGT<br>ATGCGGCGACCGAGTTGCTCTTGCCCGGCGTCAATACGGGATAATACCGCGCCAC<br>ATAGCAGAACTTTAAAAGTGCTCATCATTGGAAAACGTTCTTCGGGGCGAAAACTC<br>TCAAGGATCTTACCGCTGTTGAGATCCAGTTCGATGTAACCCACTCGTGCACCCAA<br>CTGATCTTCAGCATCTTTTACTTTCACCAGCGTTTCTGGGTGAGCAAAAACAGGAA<br>GGCAAAATGCCGCAAAAAAGGGAATAAGGGCGACACGGAAATGTTGAATACTCATA<br>CTCTTCCTTTTTCAATATTATTGAAGCATTTATCAGGGTTATTGTCTCATGAGCGGAT<br>ACATATTTGAATGTATTTAGAAAAATAAACAAATAGGGGTTCCGCGCACATTTCCCC<br>GAAAAGTGCCACCTGAACGAAGCATCTGTGCTTCATTTTGTAGAACAAAAATGCAA<br>CGCGAGAGCGCTAATTTTTCAAACAAAGAATCTGAGCTGCATTTTTACAGAACAGAA<br>ATGCAACGCGAAAGCGCTATTTTACCAACGAAGAATCTGTGCTTCATTTTTGTAAAA<br>CAAAAATGCAACGCGAGAGCGCTAATTTTTCAAACAAAGAATCTGAGCTGCATTTTT<br>ACAGAACAGAAATGCAACGCGAGAGCGCTATTTTACCAACAAAGAATCTATACTTCT<br>TTTTTGTTCTACAAAAATGCATCCCGAGAGCGCTATTTTTCTAACAAAGCATCTTAG<br>ATTACTTTTTTTCTCCTTTGTGCGCTCTATAATGCAGTCTCTTGATAACTTTTTGCAC<br>TGTAGGTCCGTTAAGGTTAGAAGAAGGCTACTTTGGTGTCTATTTTCTCTTCCATAA<br>AAAAAGCCTGACTCCACTTCCCGCGTTTACTGATTACTAGCGAAGCTGCGGGTGCA

	TTTTTTCAAGATAAAGGCATCCCCGATTATATTCTATACCGATGTGGATTGCGCATA CTTTGTGAACAGAAAGTGATAGCGTTGATGATTCTTCATTGGTCAGAAAATTATGAA CGGTTTCTTCTATTTTGTCTCTATATACTACGTATAGGAAATGTTTACATTTTCGTAT TGTTTTCGATTCACTCTATGAATAGTTCTTACTACAATTTTTTTGTCTAAAGAGTAATA CTAGAGATAAACATAAAAAATGTAGAGGTCGAGTTTAGATGCAAGTTCAAGGAGCG AAAGGTGGATGGGTAGGTTATATAGGGATATAGCACAGAGATATATAGCAAAGAGA TACTTTTGAGCAATGTTTGTGGAAGCGGTATTCGCAATATTTTAGTAGCTCGTTACA GTCCGGTGCGTTTTTGGTTTTTTGAAAGTGCGTCTTCAGAGCGCTTTTGGTTTTCAA AAGCGCTCTGAAGTTCCTATACTTTCTAGAGAATAGGAACTTCGGAATAGGAACTTC AAAGCGTTTCCGAAAACGAGCGCTTCCGAAAATGCAACGCGAGCTGCGCACATAC AGCTCACTGTTCACGTCGCACCTATATCTGCGTGTTGCCTGTATATATATATACATG AGAAGAACGGCATAGTGCGTGTTTATGCTTAAATGCGTACTTATATGCGTCTATTTA TGTAGGATGAAAGGTAGTCTAGTACCTCCTGTGATATTATCCCATTCCATGCGGGG TATCGTATGCTTCCTTCAGCACTACCCTTTAGCTGTTCTATATGCTGCCACTCCTCA ATTGGATTAGTCTCATCCTTCAATGCTATCATTTCCTTTGATATTGGATCATACTAAG AAACCATTATTATCATGACATTAACCTATAAAAATAGGCGTATCACGAGGCCCTTTC GTC
pHV013	TCGCGCGAACCCAACTGCACAGAACAAAAACCTGCAGGAAACGAAGATAAATCGC TTAACTATGCGGCATCAGAGCAGATTGTACTGAGAGTGCACCATAAACGACATTAC TATATATATAATATAGGAAGCATTTAATAGACAGCATCGTAATATATGTGTACTTTGC AGTTATGACGCCAGATGGCAGTAGTGGAAGATATTCTTTATTGAAAAATAGCTTGTC ACCTTACGTACAATCTTGATCCGGAGCTTTTCTTTTTTTGCCGATTAAGAATTAATTC GGTCGAAAAAAGAAAAGGAGAGGGCCAAGAGGGAGGGCATTGGTGACTATTGAGC ACGTGAGTATACGTGATTAAGCACACAAAGGCAGCTTGGAGTATGTCTGTTATTAAT TTCACAGGTAGTTCTGGTCCATTGGTGAAAGTTTGCGGCTTGCAGAGCACAGAGG CCGCAGAATGTGCTCTAGATTCCGATGCTGACTTGCTGGGTATTATATGTGTGCCC AATAGAAAGAGAACAATTGACCCGGTTATTGCAAGGAAAATTTCAAGTCTTGTAAAA GCATATAAAAATAGTTCAGGCACTCCGAAATACTTGGTTGGCGTGTTTCGTAATCAA CCTAAGGAGGATGTTTTGGCTCTGGTCAATGATTACGGCATTGATATCGTCCAACT GCATGGAGATGAGTCGTGGCAAGAATACCAAGAGTTCCTCGGTTTGCCAGTTATTA AAAGACTCGTATTTCCAAAAGACTGCAACATACTACTCAGTGCAGCTTCACAGAAAC CTCATTCGTTTATTCCCTTGTTTGATTCAGAAGCAGGTGGGACAGGTGAACTTTTGG ATTGGAACTCGATTTCTGACTGGGTTGGAAGGCAAGAGAGCCCCGAAAGCTTACAT TTTATGTTAGCTGGTGGACTGACGCCAGAAAATGTTGGTGATGCGCTTAGATTAAA TGGCGTTATTGGTGTTGATGTAAGCGGAGGTGTGGAGACAAATGGTGTAAAAGACT CTAACAAAATAGCAAATTTCGTCAAAAATGCTAAGAAATAGGTTATTACTGAGTAGT ATTTATTTAAGTATTGTTTGTGCACTTGCCTATGCGGTGTGAAATACCGCACAGATG CGTAAGGAGAAAATACCGCATCAGGAAATTGTAAACGTTAATAAAACTGTATTATAA GTAAATGCATGTATACTAAACTCACAAATTAGAGCTTCAATTTAATTATATCAGTTAT TACCCTGTGCTCGAGTCTTTGAAAAGATAATGTATGATTATGCTTTCACTCATATTTA TACAGAAACTTGATGTTTTCTTTCGAGTATATACAAGGTGATTACATGTACGTTTGAA GTACAACTCTAGATTTTGTAGTGCCCTCTTGGGCTAGCGGTAAAGGTGCGCATTTT TTCACACCCTACAATGTTCTGTTCAAAAGATTTTGGTCAAACGCTGTAGAAGTGAAA GTTGGTGCGCATGTTTCGGCGTTCGAAACTTCTCCGCAGTGAAAGATAAATGATCG GGTTTTAgAGCTATGCTGTTTTGAATGGTCCCAAAGAGATAGGTAAATAAACGCGAG AGCTATGCTGTTTTGAATGGTCCCAAAACTTTTTTTGTTTTTTATGTCTGCATAGCTT CAAAATGTTTCTACTCCTTTTTTACTCTTCCAGATTTTCTCGGACTCCGCGCATCGC CGTACCACTTCAAAACACCCAAGCACAGCATACTAAATTTCCCCTCTTTCTTCCTCT AGGGTGTCGTTAATTACCCGTACTAAAGGTTTGGAAAAGAAAAAAGAGGCCGCCTC GTTTCTTTTTCTTCGTCGAAAAAGGCAATAAAAATTTTTATCACGTTTCTTTTTCTTGA AAATTTTTTTTTTTGATTTTTTTCTCTTTCGATGACCTCCCATTGATATTTAAGTTAAT AAACGGTCTTCAATTTCTCAAGTTTCAGTTTCATTTTTCTTGTTCTATTACAACTTTTT TTACTTCTTGCTCATTAGAAAGAAAGCATAGCAATCTAATCTAAGTTTTCTAGATGGA TTATAAAGATGACGATGACAAACCTCCAAAAAAGAAGAGAAAGGTCGATAAGAAAT ACTCAATAGGCTTAGATATCGGCACAAATAGCGTCGGATGGGCGGTGATCACTGAT GAATATAAGGTTCCGTCTAAAAAGTTCAAGGTTCTGGGAAATACAGACCGCCACAG TATCAAAAAAAAATCTTATAGGGGCTCTTTTATTTGACAGTGGAGAGACAGCGGAAG CGACTCGTCTCAAACGGACAGCTCGTAGAAGGTATACACGTCGGAAGAATCGTATT TGTTATCTACAGGAGATTTTTTCAAATGAGATGGCGAAAGTAGATGATAGTTTCTTT CATCGACTTGAAGAGTCTTTTTTGGTGGAAGAAGACAAGAAGCATGAACGTCATCC TATTTTTGGAAATATAGTAGATGAAGTTGCTTATCATGAGAAATATCCAACTATCTAT

	CATCTGCGAAAAAAATTGGTAGATTCTACTTATAAAGCGGATTTGCGCTTAATCTAT<br>TTGGCCTTAGCGCATATGATTAAGTTTCGTGGTCATTTTTTGATTGAGGGAGATTTA<br>AATCCTGATAATAGTGATGTGGACAAACTATTTATCCAGTTGGTACAAACCTACAAT<br>CAATTATTTGAAGAAAACCCTATTAACGCAAGTGGAGTAGATGCTAAAGCGATTCTT<br>TCTGCACGATTGAGTAAATCAAGACGATTAGAAAATCTCATTGCTCAGCTCCCCGG<br>TGAGAAGAAAAATGGCTTATTTGGGAATCTCATTGCTTTGTCATTGGGTTTGACCCC<br>TAATTTTAAATCAAATTTTGATTTGGCAGAAGATGCTAAATTACAGCTTTCAAAAGAT<br>ACTTACGATGATGATTTAGATAATTTATTGGCGCAAATTGGAGATCAATATGCTGAT<br>TTGTTTTTGGCAGCTAAGAATTTATCAGATGCTATTTTACTTTCAGATATCCTAAGAG<br>TAAATACTGAAATAACTAAGGCTCCCCTATCAGCTTCAATGATTAAACGCTACGATG<br>AACATCATCAAGACTTGACTCTTTTAAAAGCTTTAGTTCGACAACAACTTCCAGAAA<br>AGTATAAAGAAATCTTTTTTGATCAATCAAAAAACGGATATGCAGGTTATATTGATG<br>GGGGAGCTAGCCAAGAAGAATTTTATAAATTTATCAAACCAATTTTAGAAAAAATGG<br>ATGGTACTGAGGAATTATTGGTGAAACTAAATCGTGAAGATTTGCTGCGCAAGCAA<br>CGGACCTTTGACAACGGCTCTATTACCCATCAAATTCACTTGGGTGAGCTGCATGC<br>TATTTTGAGAAGACAAGAAGACTTTTATCCATTTTTAAAAGACAATCGTGAGAAGATT<br>GAAAAAATCTTGACTTTTCGAATTCCTTATTATGTTGGTCCATTGGCGCGTGGCAAT<br>AGTCGTTTTGCATGGATGACTCGGAAGTCTGAAGAAACAATTACCCCATGGAATTTT<br>GAAGAAGTTGTCGATAAAGGTGCTTCAGCTCAATCATTTATTGAACGCATGACAAAC<br>TTTGATAAAAATCTTCCAAATGAAAAAGTACTACCAAAACATAGTTTGCTTTATGAGT<br>ATTTTACGGTTTATAACGAATTGACAAAGGTCAAATATGTTACTGAAGGAATGCGAA<br>AACCAGCATTTCTTTCAGGTGAACAGAAGAAAGCCATTGTTGATTTACTCTTCAAAA<br>CAAATCGAAAAGTAACCGTTAAGCAATTAAAAGAAGATTATTTCAAAAAAATAGAAT<br>GTTTTGATAGTGTTGAAATTTCAGGAGTTGAAGATAGATTTAATGCTTCATTAGGTA<br>CCTACCATGATTTGCTAAAAATTATTAAAGATAAAGATTTTTTGGATAATGAAGAAAA<br>TGAAGATATCTTAGAGGATATTGTTTTAACATTGACCTTATTTGAAGATAGGGAGAT<br>GATTGAGGAAAGACTTAAAACATATGCTCACCTCTTTGATGATAAGGTGATGAAACA<br>GCTTAAACGTCGCCGTTATACTGGTTGGGGACGTTTGTCTCGAAAATTGATTAATG<br>GTATTAGGGATAAGCAATCTGGCAAAACAATATTAGATTTTTTGAAATCAGATGGTT<br>TTGCCAATCGCAATTTTATGCAGCTGATCCATGATGATAGTTTGACATTTAAAGAAG<br>ACATTCAAAAAGCACAAGTGTCTGGACAAGGCGATAGTTTACATGAACATATTGCAA<br>ATTTAGCTGGTAGCCCTGCTATTAAAAAAGGTATTTTACAGACTGTAAAAGTTGTTG<br>ATGAATTGGTCAAAGTAATGGGGCGGCATAAGCCAGAAAATATCGTTATTGAAATG<br>GCACGTGAAAATCAGACAACTCAAAAGGGCCAGAAAAATTCGCGAGAGCGTATGA<br>AACGAATCGAAGAAGGTATCAAAGAATTAGGAAGTCAGATTCTTAAAGAGCATCCT<br>GTTGAAAATACTCAATTGCAAAATGAAAAGCTCTATCTCTATTATCTCCAAAATGGAA<br>GAGACATGTATGTGGACCAAGAATTAGATATTAATCGTTTAAGTGATTATGATGTCG<br>ATCACATTGTTCCACAAAGTTTCCTTAAAGACGATTCAATAGACAATAAGGTCTTAA<br>CGCGTTCTGATAAAAATCGTGGTAAATCGGATAACGTTCCAAGTGAAGAAGTAGTC<br>AAAAAGATGAAAAACTATTGGAGACAACTTCTAAACGCCAAGTTAATCACTCAACGT<br>AAGTTTGATAATTTAACGAAAGCTGAACGTGGAGGTTTGAGTGAACTTGATAAAGCT<br>GGTTTTATCAAACGCCAATTGGTTGAAACTCGCCAAATCACTAAGCATGTGGCACA<br>AATTTTGGATAGTCGCATGAATACTAAATACGATGAAAATGATAAACTTATTCGAGA<br>GGTTAAAGTGATTACCTTAAAATCTAAATTAGTTTCTGACTTCCGAAAAGATTTCCAA<br>TTCTATAAAGTACGTGAGATTAACAATTACCATCATGCCCATGATGCGTATCTAAAT<br>GCCGTCGTTGGAACTGCTTTGATTAAGAAATATCCAAAACTTGAATCGGAGTTTGTC<br>TATGGTGATTATAAAGTTTATGATGTTCGTAAAATGATTGCTAAGTCTGAGCAAGAA<br>ATAGGCAAAGCAACCGCAAAATATTTCTTTTACTCTAATATCATGAACTTCTTCAAAA<br>CAGAAATTACACTTGCAAATGGAGAGATTCGCAAACGCCCTCTAATCGAAACTAAT<br>GGGGAAACTGGAGAAATTGTCTGGGATAAAGGGCGAGATTTTGCCACAGTGCGCA<br>AAGTATTGTCCATGCCCCAAGTCAATATTGTCAAGAAAACAGAAGTACAGACAGGC<br>GGATTCTCCAAGGAGTCAATTTTACCAAAAAGAAATTCGGACAAGCTTATTGCTCGT<br>AAAAAAGACTGGGATCCAAAAAAATATGGTGGTTTTGATAGTCCAACGGTAGCTTAT<br>TCAGTCCTAGTGGTTGCTAAGGTGGAAAAAGGGAAATCGAAGAAGTTAAAATCCGT<br>TAAAGAGTTACTAGGGATCACAATTATGGAAAGAAGTTCCTTTGAAAAAAATCCGAT<br>TGACTTTTTAGAAGCTAAAGGATATAAGGAAGTTAAAAAAGACTTAATCATTAAACTA<br>CCTAAATATAGTCTTTTTGAGTTAGAAAACGGTCGTAAACGGATGCTGGCTAGTGC<br>CGGAGAATTACAAAAAGGAAATGAGCTGGCTCTGCCAAGCAAATATGTGAATTTTTT<br>ATATTTAGCTAGTCATTATGAAAAGTTGAAGGGTAGTCCAGAAGATAACGAACAAAA<br>ACAATTGTTTGTGGAGCAGCATAAGCATTATTTAGATGAGATTATTGAGCAAATCAG<br>TGAATTTTCTAAGCGTGTTATTTTAGCAGATGCCAATTTAGATAAAGTTCTTAGTGCA

TATAACAAACATAGAGACAAACCAATACGTGAACAAGCAGAAAATATTATTCATTTAT
TTACGTTGACGAATCTTGGAGCTCCCGCTGCTTTTAAATATTTTGATACAACAATTG
ATCGTAAACGATATACGTCTACAAAAGAAGTTTTAGATGCCACTCTTATCCATCAAT
CCATCACTGGTCTTTATGAAACACGCATTGATTTGAGTCAGCTAGGAGGTGACCCT
CCAAAAAAGAAGAGAAAGGTCTGAGCGGATCTCTTATGTCTTTACGATTTATAGTTT
TCATTATCAAGTATGCCTATATTAGTATATAGCATCTTTAGATGACAGTGTTCGAAGT
TTCACGAATAAAAGATAATATTCTACTTTTTGCTCCCACCGCGTTTGCTAGCACGAG
TGAACACCATCCCTCGCCTGTGAGTTGTACCCATTCCTCTAAACTGTAGACATGGT
AGCTTCAGCAGTGTTCGTTATGTACGGCATCCTCCAACAAACAGTCGGTTATAGTTT
GTCCTGCTCCTCTGAATCGAGTCCCTCGATATTTCTCATACTAGTTCTAGAGATCTG
CCAATTGAACATAACATGGTAGTTACATATACTAGTAATATGGTTCGGCACACATTA
AAAGTATAAAAACTATCTGAATTACGAATTACATATATTGGTCATAAAAATCAATCAA
TCATCGTGTGTTTTATATGTCTCTTATCTAAGTATAAGAATATCCATAGTTAATATTC
ACTTACGCTACCTTTTAACCTGTAATCATTGTCAACAGGATATGTTAACGACCCACA
TTGATAAACGCTAGTATTTCTTTTTCCTCTTCTTATTGGCCGGCTGTCTCTATACTCC
CCTATAGTCTGTTTCTTTTCGTTTCGATTGTTTTACGTTTGAGGCCTCGTGGCGCAC
ATGGTACGCTGTGGTGCTCGCGGCTGGGAACGAAACTCTGGGAGCTGCGATTGG
CAGGAACCATTCAAAACAGCATAGCAAGTTAAAATAAGGCTAGTCCGTTATCAACTT
GAAAAAGTGGCACCGAGTCGGTGCTTTTTTTCCATATCCAACTTCCAATTTAATCTT
TCTTTTTTAATTTTCACTTATTTGCGATACAGAAAGACCCTGCAGGCATGCAAGCTT
GGCGTAATCATGGTCATAGCTGTTTCCTGTGTGAAATTGTTATCCGCTCACAATTCC
ACACAACATACGAGCCGGAAGCATAAAGTGTAAAGCCTGGGGTGCCTAATGAGTG
AGCTAACTCACATTAATTGCGTTGCGCTCACTGCCCGCTTTCCAGTCGGGAAACCT
GTCGTGCCAGCTGCATTAATGAATCGGCCAACGCGCGGGGAGAGGCGGTTTGCG
TATTGGGCGCTCTTCCGCTTCCTCGCTCACTGACTCGCTGCGCTCGGTCGTTCGG
CTGCGGCGAGCGGTATCAGCTCACTCAAAGGCGGTAATACGGTTATCCACAGAAT
CAGGGGATAACGCAGGAAAGAACATGTGAGCAAAAGGCCAGCAAAAGGCCAGGAA
CCGTAAAAAGGCCGCGTTGCTGGCGTTTTTCCATAGGCTCCGCCCCCCTGACGAG
CATCACAAAAATCGACGCTCAAGTCAGAGGTGGCGAAACCCGACAGGACTATAAA
GATACCAGGCGTTTCCCCCTGGAAGCTCCCTCGTGCGCTCTCCTGTTCCGACCCT
GCCGCTTACCGGATACCTGTCCGCCTTTCTCCCTTCGGGAAGCGTGGCGCTTTCT
CATAGCTCACGCTGTAGGTATCTCAGTTCGGTGTAGGTCGTTCGCTCCAAGCTGG
GCTGTGTGCACGAACCCCCCGTTCAGCCCGACCGCTGCGCCTTATCCGGTAACTA
TCGTCTTGAGTCCAACCCGGTAAGACACGACTTATCGCCACTGGCAGCAGCCACT
GGTAACAGGATTAGCAGAGCGAGGTATGTAGGCGGTGCTACAGAGTTCTTGAAGT
GGTGGCCTAACTACGGCTACACTAGAAGAACAGTATTTGGTATCTGCGCTCTGCTG
AAGCCAGTTACCTTCGGAAAAAGAGTTGGTAGCTCTTGATCCGGCAAACAAACCAC
CGCTGGTAGCGGTGGTTTTTTTGTTTGCAAGCAGCAGATTACGCGCAGAAAAAAAG
GATCTCAAGAAGATCCTTTGATCTTTTCTACGGGGTCTGACGCTCAGTGGAACGAA
AACTCACGTTAAGGGATTTTGGTCATGAGATTATCAAAAAGGATCTTCACCTAGATC
CTTTTAAATTAAAAATGAAGTTTTAAATCAATCTAAAGTATATATGAGTAAACTTGGT
CTGACAGTTACCAATGCTTAATCAGTGAGGCACCTATCTCAGCGATCTGTCTATTTC
GTTCATCCATAGTTGCCTGACTCCCCGTCGTGTAGATAACTACGATACGGGAGGGC
TTACCATCTGGCCCCAGTGCTGCAATGATACCGCGGCTCCCACGCTCACCGGCTC
CAGATTTATCAGCAATAAACCAGCCAGCCGGAAGGGCCGAGCGCAGAAGTGGTCC
TGCAACTTTATCCGCCTCCATCCAGTCTATTAATTGTTGCCGGGAAGCTAGAGTAA
GTAGTTCGCCAGTTAATAGTTTGCGCAACGTTGTTGCCATTGCTACAGGCATCGTG
GTGTCACGCTCGTCGTTTGGTATGGCTTCATTCAGCTCCGGTTCCCAACGATCAAG
GCGAGTTACATGATCCCCCATGTTGTGCAAAAAAGCGGTTAGCTCCTTCGGTCCTC
CGATCGTTGTCAGAAGTAAGTTGGCCGCAGTGTTATCACTCATGGTTATGGCAGCA
CTGCATAATTCTCTTACTGTCATGCCATCCGTAAGATGCTTTTCTGTGACTGGTGAG
TACTCAACCAAGTCATTCTGAGAATAGTGTATGCGGCGACCGAGTTGCTCTTGCCC
GGCGTCAATACGGGATAATACCGCGCCACATAGCAGAACTTTAAAAGTGCTCATCA
TTGGAAAACGTTCTTCGGGGCGAAAACTCTCAAGGATCTTACCGCTGTTGAGATCC
AGTTCGATGTAACCCACTCGTGCACCCAACTGATCTTCAGCATCTTTTACTTTCACC
AGCGTTTCTGGGTGAGCAAAAACAGGAAGGCAAAATGCCGCAAAAAAGGGAATAA
GGGCGACACGGAAATGTTGAATACTCATACTCTTCCTTTTTCAATATTATTGAAGCA
TTTATCAGGGTTATTGTCTCATGAGCGGATACATATTTGAATGTATTTAGAAAAATAA
ACAAATAGGGGTTCCGCGCACATTTCCCCGAAAAGTGCCACCTGAACGAAGCATCT
GTGCTTCATTTTGTAGAACAAAAATGCAACGCGAGAGCGCTAATTTTTCAAACAAAG
AATCTGAGCTGCATTTTTACAGAACAGAAATGCAACGCGAAAGCGCTATTTTACCAA

	CGAAGAATCTGTGCTTCATTTTTGTAAAACAAAAATGCAACGCGAGAGCGCTAATTT<br>TTCAAACAAAGAATCTGAGCTGCATTTTTACAGAACAGAAATGCAACGCGAGAGCG<br>CTATTTTACCAACAAAGAATCTATACTTCTTTTTTGTTCTACAAAAATGCATCCCGAG<br>AGCGCTATTTTTCTAACAAAGCATCTTAGATTACTTTTTTTCTCCTTTGTGCGCTCTA<br>TAATGCAGTCTCTTGATAACTTTTTGCACTGTAGGTCCGTTAAGGTTAGAAGAAGGC<br>TACTTTGGTGTCTATTTTCTCTTCCATAAAAAAAGCCTGACTCCACTTCCCGCGTTT<br>ACTGATTACTAGCGAAGCTGCGGGTGCATTTTTTCAAGATAAAGGCATCCCCGATT<br>ATATTCTATACCGATGTGGATTGCGCATACTTTGTGAACAGAAAGTGATAGCGTTGA<br>TGATTCTTCATTGGTCAGAAAATTATGAACGGTTTCTTCTATTTTGTCTCTATATACT<br>ACGTATAGGAAATGTTTACATTTTCGTATTGTTTTCGATTCACTCTATGAATAGTTCT<br>TACTACAATTTTTTTGTCTAAAGAGTAATACTAGAGATAAACATAAAAAATGTAGAGG<br>TCGAGTTTAGATGCAAGTTCAAGGAGCGAAAGGTGGATGGGTAGGTTATATAGGG<br>ATATAGCACAGAGATATATAGCAAAGAGATACTTTTGAGCAATGTTTGTGGAAGCG<br>GTATTCGCAATATTTTAGTAGCTCGTTACAGTCCGGTGCGTTTTTGGTTTTTTGAAA<br>GTGCGTCTTCAGAGCGCTTTTGGTTTTCAAAAGCGCTCTGAAGTTCCTATACTTTCT<br>AGAGAATAGGAACTTCGGAATAGGAACTTCAAAGCGTTTCCGAAAACGAGCGCTTC<br>CGAAAATGCAACGCGAGCTGCGCACATACAGCTCACTGTTCACGTCGCACCTATAT<br>CTGCGTGTTGCCTGTATATATATATACATGAGAAGAACGGCATAGTGCGTGTTTATG<br>CTTAAATGCGTACTTATATGCGTCTATTTATGTAGGATGAAAGGTAGTCTAGTACCT<br>CCTGTGATATTATCCCATTCCATGCGGGGTATCGTATGCTTCCTTCAGCACTACCCT<br>TTAGCTGTTCTATATGCTGCCACTCCTCAATTGGATTAGTCTCATCCTTCAATGCTA<br>TCATTTCCTTTGATATTGGATCATACTAAGAAACCATTATTATCATGACATTAACCTA<br>TAAAAATAGGCGTATCACGAGGCCCTTTCGTC
pIV001	GAGATACCTACAGCGTGAGCTATGAGAAAGCGCCACGCTTCCCGAAGGGAGAAAG<br>GCGGACAGGTATCCGGTAAGCGGCAGGGTCGGAACAGGAGAGCGCACGAGGGA<br>GCTTCCAGGGGGAAACGCCTGGTATCTTTATAGTCCTGTCGGGTTTCGCCACCTCT<br>GACTTGAGCGTCGATTTTTGTGATGCTCGTCAGGGGGGCGGAGCCTATGGAAAAA<br>CGCCAGCAACGCGGCCTTTTTACGGTTCCTGGCCTTTTGCTGGCCTTTTGCTCACA<br>TGTTCTTTCCTGCGTTATCCCCTGATTCTGTGGATAACCGTATTACCGCCTTTGAGT<br>GAGCTGATACCGCTCGCCGCAGCCGAACGACCGAGCGCAGCGAGTCAGTGAGCG<br>AGGAAGCGGAAGAGCGCCCAATACGCAAACCGCCTCTCCCCGCGCGTTGGCCGA<br>TTCATTAATGCAGCTGGCACGACAGGTTTCCCGACTGGAAAGCGGGCAGTGAGCG<br>CAACGCAATTAATGTGAGTTAGCTCACTCATTAGGCACCCCAGGCTTTACACTTTAT<br>GCTTCCGGCTCGTATGTTGTGTGGAATTGTGAGCGGATAACAATTTCACACAGGAA<br>ACAGCTATGACCATGATTACGCCAAGCTTGCATGCCTGCAGGTCGACTCTAGAtgga<br>acaacgacatctgttggtgctgtctttgtcgttaatttttcctttagtgtcttccatcatttttttgtcattgcggatatggtgagacaa<br>caacgggggagagagaaaagaaaaaaaaagaaaagaagttgcatgcgcctattattacttcaatagatggcaaatgg<br>aaaaagggtagtgaaacttcgatatgatgatggctatcaagtctagggctacagtattagttcgttatgtaccaccatcaatg<br>aggcagtgtaattggtgtagtcttgtttagcccattatgtcttgtctggtatctgttctattgtatatctcccctccgccacctacatgt<br>tagggagaccaacgaaggtattataggaatcccgatgtatgggtttggttgccagaaaagaggaagtccatattgtacacc<br>cggaaacaacaaaaggatatccgaaatattccacggtttagaaaaaaatcggaaaagagcgcggaggggtgttaCC<br>TAGTACACTCTATATTTTTTTATGCCTCGGTAATGATTTTCATTTTTTTTTTTCCACCT<br>AGCGGATGACTCTTTTTTTTTCTTAGCGATTGGCATTATCACATAATGAATTATACAT<br>TATATAAAGTAATGTGATTTCTTCGAAGAATATACTAAAAAATGAGCAGGCAAGATA<br>AACGAAGGCAAAGATGACAGAGCAGAAAGCCCTAGTAAAGCGTATTACAAATGAAA<br>CCAAGATTCAGATTGCGATCTCTTTAAAGGGTGGTCCCCTAGCGATAGAGCACTCG<br>ATCTTCCCAGAAAAAGAGGCAGAAGCAGTAGCAGAACAGGCCACACAATCGCAAG<br>TGATTAACGTCCACACAGGTATAGGGTTTCTGGACCATATGATACATGCTCTGGCC<br>AAGCATTCCGGCTGGTCGCTAATCGTTGAGTGCATTGGTGACTTACACATAGACGA<br>CCATCACACCACTGAAGACTGCGGGATTGCTCTCGGTCAAGCTTTTAAAGAGGCCC<br>TAGGGGCCGTGCGTGGAGTAAAAAGGTTTGGATCAGGATTTGCGCCTTTGGATGA<br>GGCACTTTCCAGAGCGGTGGTAGATCTTTCGAACAGGCCGTACGCAGTTGTCGAA<br>CTTGGTTTGCAAAGGGAGAAAGTAGGAGATCTCTCTTGCGAGATGATCCCGCATTT<br>TCTTGAAAGCTTTGCAGAGGCTAGCAGAATTACCCTCCACGTTGATTGTCTGCGAG<br>GCAAGAATGATCATCACCGTAGTGAGAGTGCGTTCAAGGCTCTTGCGGTTGCCATA<br>AGAGAAGCCACCTCGCCCAATGGTACCAACGATGTTCCCTCCACCAAAGGTGTTCT<br>TATGTAGTGACACCGATTATTTAAAGCTGCAGCATACGATATATATACATGTGTATA<br>TATGTATACCTATGAATGTCAGTAAGTATGTATACGAACAGTATGATACTGAAGATG<br>ACAAGGTAATGCATCATTCTATACGTGTCATTCTGAACGAGGCGCGCTTTCCTTTTT<br>TCTTTTTGCTTTTTCTTTTTTTTTCTCTTGAACTCGACGGATCTATGCGCCATGGGCT<br>AGCCTCGAGAGATCTGCGGCCGCaatatatgtgcataggagaagtgtaaagttcccttccatattgtaacat

	aataaagtgcacacccaaatgaattgaaagcgtactcaaacagacaaccatttccagtgttgtatgtacctgtctatttatact<br>ggtagcaaccctattgctgtttcctcttcaaagtactctagcggttatgcgcgtctcaccttcaaggtcatggtcgctctattgttc<br>gcaccaccggcaaactcgcgtctcgcaagtcttggctcattcttctagtatactcatgttgcaaatgcactcaggttctttcggc<br>aacttaaataatgacaccagttgtcgtggtcgtcatcatcgcaaccccaaccggcattcttattgcttctccaatctcgcccctt<br>agcgcagggtaaaccttggaaaatgcaggcgcaaaaaactccgccgggcacagcctcacgcccagcgttatcgccgg<br>gccggcaagagcgcgggtccgccacagagtcagGGATCCCCGGGTACCGAGCTCGAATTCACT<br>GGCCGTCGTTTTACAACGTCGTGACTGGGAAAACCCTGGCGTTACCCAACTTAATC<br>GCCTTGCAGCACATCCCCCTTTCGCCAGCTGGCGTAATAGCGAAGAGGCCCGCAC<br>CGATCGCCCTTCCCAACAGTTGCGCAGCCTGAATGGCGAATGGCGCCTGATGCGG<br>TATTTTCTCCTTACGCATCTGTGCGGTATTTCACACCGCATATGGTGCACTCTCAGT<br>ACAATCTGCTCTGATGCCGCATAGTTAAGCCAGCCCCGACACCCGCCAACACCCG<br>CTGACGCGCCCTGACGGGCTTGTCTGCTCCCGGCATCCGCTTACAGACAAGCTGT<br>GACCGTCTCCGGGAGCTGCATGTGTCAGAGGTTTTCACCGTCATCACCGAAACGC<br>GCGAGACGAAAGGGCCTCGTGATACGCCTATTTTTATAGGTTAATGTCATGATAAT<br>AATGGTTTCTTAGACGTCAGGTGGCACTTTTCGGGGAAATGTGCGCGGAACCCCTA<br>TTTGTTTATTTTTCTAAATACATTCAAATATGTATCCGCTCATGAGACAATAACCCTG<br>ATAAATGCTTCAATAATATTGAAAAAGGAAGAGTATGAGTATTCAACATTTCCGTGT<br>CGCCCTTATTCCCTTTTTTGCGGCATTTTGCCTTCCTGTTTTTGCTCACCCAGAAAC<br>GCTGGTGAAAGTAAAAGATGCTGAAGATCAGTTGGGTGCACGAGTGGGTTACATC<br>GAACTGGATCTCAACAGCGGTAAGATCCTTGAGAGTTTTCGCCCCGAAGAACGTTT<br>TCCAATGATGAGCACTTTTAAAGTTCTGCTATGTGGCGCGGTATTATCCCGTATTGA<br>CGCCGGGCAAGAGCAACTCGGTCGCCGCATACACTATTCTCAGAATGACTTGGTT<br>GAGTACTCACCAGTCACAGAAAAGCATCTTACGGATGGCATGACAGTAAGAGAATT<br>ATGCAGTGCTGCCATAACCATGAGTGATAACACTGCGGCCAACTTACTTCTGACAA<br>CGATCGGAGGACCGAAGGAGCTAACCGCTTTTTTGCACAACATGGGGGATCATGT<br>AACTCGCCTTGATCGTTGGGAACCGGAGCTGAATGAAGCCATACCAAACGACGAG<br>CGTGACACCACGATGCCTGTAGCAATGGCAACAACGTTGCGCAAACTATTAACTGG<br>CGAACTACTTACTCTAGCTTCCCGGCAACAATTAATAGACTGGATGGAGGCGGATA<br>AAGTTGCAGGACCACTTCTGCGCTCGGCCCTTCCGGCTGGCTGGTTTATTGCTGAT<br>AAATCTGGAGCCGGTGAGCGTGGGTCTCGCGGTATCATTGCAGCACTGGGGCCA<br>GATGGTAAGCCCTCCCGTATCGTAGTTATCTACACGACGGGGAGTCAGGCAACTAT<br>GGATGAACGAAATAGACAGATCGCTGAGATAGGTGCCTCACTGATTAAGCATTGGT<br>AACTGTCAGACCAAGTTTACTCATATATACTTTAGATTGATTTAAAACTTCATTTTTAA<br>TTTAAAAGGATCTAGGTGAAGATCCTTTTTGATAATCTCATGACCAAAATCCCTTAA<br>CGTGAGTTTTCGTTCCACTGAGCGTCAGACCCCGTAGAAAAGATCAAAGGATCTTC<br>TTGAGATCCTTTTTTTCTGCGCGTAATCTGCTGCTTGCAAACAAAAAAACCACCGCT<br>ACCAGCGGTGGTTTGTTTGCCGGATCAAGAGCTACCAACTCTTTTTCCGAAGGTAA<br>CTGGCTTCAGCAGAGCGCAGATACCAAATACTGTTCTTCTAGTGTAGCCGTAGTTA<br>GGCCACCACTTCAAGAACTCTGTAGCACCGCCTACATACCTCGCTCTGCTAATCCT<br>GTTACCAGTGGCTGCTGCCAGTGGCGATAAGTCGTGTCTTACCGGGTTGGACTCA<br>AGACGATAGTTACCGGATAAGGCGCAGCGGTCGGGCTGAACGGGGGGTTCGTGC<br>ACACAGCCCAGCTTGGAGCGAACGACCTACACCGAACT
pIV002	GAGATACCTACAGCGTGAGCTATGAGAAAGCGCCACGCTTCCCGAAGGGAGAAAG<br>GCGGACAGGTATCCGGTAAGCGGCAGGGTCGGAACAGGAGAGCGCACGAGGGA<br>GCTTCCAGGGGGAAACGCCTGGTATCTTTATAGTCCTGTCGGGTTTCGCCACCTCT<br>GACTTGAGCGTCGATTTTTGTGATGCTCGTCAGGGGGGCGGAGCCTATGGAAAAA<br>CGCCAGCAACGCGGCCTTTTTACGGTTCCTGGCCTTTTGCTGGCCTTTTGCTCACA<br>TGTTCTTTCCTGCGTTATCCCCTGATTCTGTGGATAACCGTATTACCGCCTTTGAGT<br>GAGCTGATACCGCTCGCCGCAGCCGAACGACCGAGCGCAGCGAGTCAGTGAGCG<br>AGGAAGCGGAAGAGCGCCCAATACGCAAACCGCCTCTCCCCGCGCGTTGGCCGA<br>TTCATTAATGCAGCTGGCACGACAGGTTTCCCGACTGGAAAGCGGGCAGTGAGCG<br>CAACGCAATTAATGTGAGTTAGCTCACTCATTAGGCACCCCAGGCTTTACACTTTAT<br>GCTTCCGGCTCGTATGTTGTGTGGAATTGTGAGCGGATAACAATTTCACACAGGAA<br>ACAGCTATGACCATGATTACGCCAAGCTTGCATGCCTGCAGGTCGACTCTAGAcaac<br>tatgaaagaagatcaagcgagtccatcggcgacctgccgcaccgcgtaaactctcttaacatacggccgtacagaaaa<br>aacggcactggagtgagcagtgtctccgacgatttggatatcgatttcaacgatagttttgtctcagaagaaagttataaacg<br>tgccgctgcagtgacatctaccctaaaggccagaattgaaaaaatgaaggccaaatcaaggcgggaagggacaacca<br>ggacgtaaagggtagcctccccataacataaactcaataaaatatatagtcttcaacttgaaaaaggaacaagctcatgc<br>aaagaggtggtacccgcacgccgaaatgcatgcaagtaacctattcaaagtaatatctcatacatgtttcatgagggtaac<br>aacatgcgactgggtgagcatatgttccgctgatgtgatgtgcaagataaacaagcaaggcagaaactaacttcttcttcat<br>gtaataaacacaccccGTATCACGAGGCCCTTTCGTCTCGCGCGAACCCAACTGCACAGA

	ACAAAAACCTGCAGGAAACGAAGATAAATCATGTCGAAAGCTACATATAAGGAACG<br>TGCTGCTACTCATCCTAGTCCTGTTGCTGCCAAGCTATTTAATATCATGCACGAAAA<br>GCAAACAAACTTGTGTGCTTCATTGGATGTTCGTACCACCAAGGAATTACTGGAGT<br>TAGTTGAAGCATTAGGTCCCAAAATTTGTTTACTAAAAACACATGTGGATATCTTGA<br>CTGATTTTTCCATGGAGGGCACAGTTAAGCCGCTAAAGGCATTATCCGCCAAGTAC<br>AATTTTTTACTCTTCGAAGACAGAAAATTTGCTGACATTGGTAATACAGTCAAATTGC<br>AGTACTCTGCGGGTGTATACAGAATAGCAGAATGGGCAGACATTACGAATGCACAC<br>GGTGTGGTGGGCCCAGGTATTGTTAGCGGTTTGAAGCAGGCGGCAGAAGAAGTAA<br>CAAAGGAACCTAGAGGCCTTTTGATGTTAGCAGAATTGTCATGCAAGGGCTCCCTA<br>TCTACTGGAGAATATACTAAGGGTACTGTTGACATTGCGAAGAGCGACAAAGATTT<br>TGTTATCGGCTTTATTGCTCAAAGAGACATGGGTGGAAGAGATGAAGGTTACGATT<br>GGTTGATTATGACACCCGGTGTGGGTTTAGATGACAAGGGAGACGCATTGGGTCA<br>ACAGTATAGAACCGTGGATGATGTCGTTTCTACAGGATCTGACATTATTATTGTTGG<br>AAGAGGACTATTTGCAAAGGGAAGGGATGCTAAGGTAGAGGGTGAACGTTACAGA<br>AAAGCAGGCTGGGAAGCATATTTGAGAAGATGCGGCCAGCAAAACTAAAAAACTGT<br>ATTATAAGTAAATGCATGTATACTAAACTCACAAATTAGAGCTTCAATTTAATTATAT<br>CAGTTATTACCCTGTGCTCGCCATGGGCTAGCCTCGAGAGATCTGCGGCCGCtaaac<br>ttcaacaccttatatcataactaatatttcttgagataagcacactgcacccataccttccttaaaaacgtagcttccagtttttg<br>gtggttccggcttccttcccgattccgcccgctaaacgcatatttttgttgcctggtggcatttgcaaaatgcataacctatgcatt<br>taaaagattatgtatgctcttctgacttttcgtgtgatgaggctcgtggaaaaaatgaataatttatgaatttgagaacaattttgt<br>gttgttacggtattttactatggaataatcaatcaattgaggattttatgcaaatatcgtttgaatatttttccgaccctttgagtactt<br>ttcttcataattgcataatattgtccgctgccccttttttctgttagacggtgtcttgatctacttgctatcgttcaacaccaccttattttc<br>taactattttttttttagctcatttgaatcagcttatggtgatggcacattttGGATCCCCGGGTACCGAGCTCGAA<br>TTCACTGGCCGTCGTTTTACAACGTCGTGACTGGGAAAACCCTGGCGTTACCCAAC<br>TTAATCGCCTTGCAGCACATCCCCCTTTCGCCAGCTGGCGTAATAGCGAAGAGGC<br>CCGCACCGATCGCCCTTCCCAACAGTTGCGCAGCCTGAATGGCGAATGGCGCCTG<br>ATGCGGTATTTTCTCCTTACGCATCTGTGCGGTATTTCACACCGCATATGGTGCACT<br>CTCAGTACAATCTGCTCTGATGCCGCATAGTTAAGCCAGCCCCGACACCCGCCAA<br>CACCCGCTGACGCGCCCTGACGGGCTTGTCTGCTCCCGGCATCCGCTTACAGACA<br>AGCTGTGACCGTCTCCGGGAGCTGCATGTGTCAGAGGTTTTCACCGTCATCACCG<br>AAACGCGCGAGACGAAAGGGCCTCGTGATACGCCTATTTTTATAGGTTAATGTCAT<br>GATAATAATGGTTTCTTAGACGTCAGGTGGCACTTTTCGGGGAAATGTGCGCGGAA<br>CCCCTATTTGTTTATTTTTCTAAATACATTCAAATATGTATCCGCTCATGAGACAATA<br>ACCCTGATAAATGCTTCAATAATATTGAAAAAGGAAGAGTATGAGTATTCAACATTT<br>CCGTGTCGCCCTTATTCCCTTTTTTGCGGCATTTTGCCTTCCTGTTTTTGCTCACCC<br>AGAAACGCTGGTGAAAGTAAAAGATGCTGAAGATCAGTTGGGTGCACGAGTGGGT<br>TACATCGAACTGGATCTCAACAGCGGTAAGATCCTTGAGAGTTTTCGCCCCGAAGA<br>ACGTTTTCCAATGATGAGCACTTTTAAAGTTCTGCTATGTGGCGCGGTATTATCCCG<br>TATTGACGCCGGGCAAGAGCAACTCGGTCGCCGCATACACTATTCTCAGAATGACT<br>TGGTTGAGTACTCACCAGTCACAGAAAAGCATCTTACGGATGGCATGACAGTAAGA<br>GAATTATGCAGTGCTGCCATAACCATGAGTGATAACACTGCGGCCAACTTACTTCT<br>GACAACGATCGGAGGACCGAAGGAGCTAACCGCTTTTTTGCACAACATGGGGGAT<br>CATGTAACTCGCCTTGATCGTTGGGAACCGGAGCTGAATGAAGCCATACCAAACGA<br>CGAGCGTGACACCACGATGCCTGTAGCAATGGCAACAACGTTGCGCAAACTATTAA<br>CTGGCGAACTACTTACTCTAGCTTCCCGGCAACAATTAATAGACTGGATGGAGGCG<br>GATAAAGTTGCAGGACCACTTCTGCGCTCGGCCCTTCCGGCTGGCTGGTTTATTG<br>CTGATAAATCTGGAGCCGGTGAGCGTGGGTCTCGCGGTATCATTGCAGCACTGGG<br>GCCAGATGGTAAGCCCTCCCGTATCGTAGTTATCTACACGACGGGGAGTCAGGCA<br>ACTATGGATGAACGAAATAGACAGATCGCTGAGATAGGTGCCTCACTGATTAAGCA<br>TTGGTAACTGTCAGACCAAGTTTACTCATATATACTTTAGATTGATTTAAAACTTCAT<br>TTTTAATTTAAAAGGATCTAGGTGAAGATCCTTTTTGATAATCTCATGACCAAAATCC<br>CTTAACGTGAGTTTTCGTTCCACTGAGCGTCAGACCCCGTAGAAAAGATCAAAGGA<br>TCTTCTTGAGATCCTTTTTTTCTGCGCGTAATCTGCTGCTTGCAAACAAAAAAACCA<br>CCGCTACCAGCGGTGGTTTGTTTGCCGGATCAAGAGCTACCAACTCTTTTTCCGAA<br>GGTAACTGGCTTCAGCAGAGCGCAGATACCAAATACTGTTCTTCTAGTGTAGCCGT<br>AGTTAGGCCACCACTTCAAGAACTCTGTAGCACCGCCTACATACCTCGCTCTGCTA<br>ATCCTGTTACCAGTGGCTGCTGCCAGTGGCGATAAGTCGTGTCTTACCGGGTTGG<br>ACTCAAGACGATAGTTACCGGATAAGGCGCAGCGGTCGGGCTGAACGGGGGGTT<br>CGTGCACACAGCCCAGCTTGGAGCGAACGACCTACACCGAACT
pIV003	GAGATACCTACAGCGTGAGCTATGAGAAAGCGCCACGCTTCCCGAAGGGAGAAAG<br>GCGGACAGGTATCCGGTAAGCGGCAGGGTCGGAACAGGAGAGCGCACGAGGGA

	GCTTCCAGGGGGAAACGCCTGGTATCTTTATAGTCCTGTCGGGTTTCGCCACCTCT<br>GACTTGAGCGTCGATTTTTGTGATGCTCGTCAGGGGGGCGGAGCCTATGGAAAAA<br>CGCCAGCAACGCGGCCTTTTTACGGTTCCTGGCCTTTTGCTGGCCTTTTGCTCACA<br>TGTTCTTTCCTGCGTTATCCCCTGATTCTGTGGATAACCGTATTACCGCCTTTGAGT<br>GAGCTGATACCGCTCGCCGCAGCCGAACGACCGAGCGCAGCGAGTCAGTGAGCG<br>AGGAAGCGGAAGAGCGCCCAATACGCAAACCGCCTCTCCCCGCGCGTTGGCCGA<br>TTCATTAATGCAGCTGGCACGACAGGTTTCCCGACTGGAAAGCGGGCAGTGAGCG<br>CAACGCAATTAATGTGAGTTAGCTCACTCATTAGGCACCCCAGGCTTTACACTTTAT<br>GCTTCCGGCTCGTATGTTGTGTGGAATTGTGAGCGGATAACAATTTCACACAGGAA<br>ACAGCTATGACCATGATTACGCCAAGCTTGCATGCCTGCAGGTCGACTCTAGAcact<br>cacatctaccagtgtacgaattgcattcagaaaactgtttgcattcaaaaataggtagcatacaattaaaacatggcgggc<br>atgtatcattgcccttatcttgtgcagttagacgcgaattttcgaagaagtaccttcaaagaatggggtcttatcttgttttgcaa<br>gtaccactgagcaggataataatagaaatgataatatactatagtagagataacgtcgatgacttcccatactgtaattgcttt<br>tagttgtgtatttttagtgtgcaagtttctgtaaatcgattaattttttttctttcctcttttattaaccttaatttttattttagattcctgactt<br>caactcaagacgcacagatattataacatctgcataataggcatttgcaagaattactcgtgagtaaggaaagagtgagg<br>aactatcgcatacctgcatttaaagatgccgatttgggcgcgaatcctttattttggcttcaccccGACATGGAGGCC<br>CAGAATACCCTCCTTGACAGTCTTGACGTGCGCAGCTCAGGGGCATGATGTGACT<br>GTCGCCCGTACATTTAGCCCATACATCCCCATGTATAATCATTTGCATCCATACATT<br>TTGATGGCCGCACGGCGCGAAGCAAAAATTACGGCTCCTCGCTGCAGACCTGCGA<br>GCAGGGAAACGCTCCCCTCACAGACGCGTTGAATTGTCCCCACGCCGCGCCCCTG<br>TAGAGAAATATAAAAGGTTAGGATTTGCCACTGAGGTTCTTCTTTCATATACTTCCTT<br>TTAAAATCTTGCTAGGATACAGTTCTCACATCACATCCGAACATAAACAACCATGGG<br>TAAGGAAAAGACTCACGTTTCGAGGCCGCGATTAAATTCCAACATGGATGCTGATT<br>TATATGGGTATAAATGGGCTCGCGATAATGTCGGGCAATCAGGTGCGACAATCTAT<br>CGATTGTATGGGAAGCCCGATGCGCCAGAGTTGTTTCTGAAACATGGCAAAGGTA<br>GCGTTGCCAATGATGTTACAGATGAGATGGTCAGACTAAACTGGCTGACGGAATTT<br>ATGCCTCTTCCGACCATCAAGCATTTTATCCGTACTCCTGATGATGCATGGTTACTC<br>ACCACTGCGATCCCCGGCAAAACAGCATTCCAGGTATTAGAAGAATATCCTGATTC<br>AGGTGAAAATATTGTTGATGCGCTGGCAGTGTTCCTGCGCCGGTTGCATTCGATTC<br>CTGTTTGTAATTGTCCTTTTAACAGCGATCGCGTATTTCGTCTCGCTCAGGCGCAAT<br>CACGAATGAATAACGGTTTGGTTGATGCGAGTGATTTTGATGACGAGCGTAATGGC<br>TGGCCTGTTGAACAAGTCTGGAAAGAAATGCATAAGCTTTTGCCATTCTCACCGGA<br>TTCAGTCGTCACTCATGGTGATTTCTCACTTGATAACCTTATTTTTGACGAGGGGAA<br>ATTAATAGGTTGTATTGATGTTGGACGAGTCGGAATCGCAGACCGATACCAGGATC<br>TTGCCATCCTATGGAACTGCCTCGGTGAGTTTTCTCCTTCATTACAGAAACGGCTTT<br>TTCAAAAATATGGTATTGATAATCCTGATATGAATAAATTGCAGTTTCATTTGATGCT<br>CGATGAGTTTTTCTAATCAGTACTGACAATAAAAAGATTCTTGTTTTCAAGAACTTGT<br>CATTTGTATAGTTTTTTTATATTGTAGTTGTTCTATTTTAATCAAATGTTAGCGTGATT<br>TATATTTTTTTTCGCCTCGACATCATCTGCCCAGATGCGAAGTTAAGTGCGCAGAAA<br>GTAATATCATGCGTCAATCGTATGTGAATGCTGGTCGCTATACTGCCATGGGCTAG<br>CCTCGAGAGATCTGCGGCCGCgaaaaggaagtgtttccctccttcttgaattgatgttaccctcataaagca<br>cgtggcctcttatcgagaaagaaattaccgtcgctcgtgatttgtttgcaaaaagaacaaaactgaaaaaacccagacac<br>gctcgacttcctgtcttcctattgattgcagcttccaatttcgtcacacaacaaggtcctagcgacggctcacaggttttgtaac<br>aagcaatcgaaggttctggaatggcgggaaagggtttagtaccacatgctatgatgcccactgtgatctccagagcaaag<br>ttcgttcgatcgtactgttactctctctctttcaaacagaattgtccgaatcgtgtgacaacaacagcctgttctcacacactctttt<br>cttctaaccaaggggggtggtttagtttagtagaacctcgtgaaacttacatttacatatatataaacttgcataaattggtcaatg<br>caagaaatacatatttggtcttttctaatGGATCCCCGGGTACCGAGCTCGAATTCACTGGCCGTC<br>GTTTTACAACGTCGTGACTGGGAAAACCCTGGCGTTACCCAACTTAATCGCCTTGC<br>AGCACATCCCCCTTTCGCCAGCTGGCGTAATAGCGAAGAGGCCCGCACCGATCGC<br>CCTTCCCAACAGTTGCGCAGCCTGAATGGCGAATGGCGCCTGATGCGGTATTTTCT<br>CCTTACGCATCTGTGCGGTATTTCACACCGCATATGGTGCACTCTCAGTACAATCT<br>GCTCTGATGCCGCATAGTTAAGCCAGCCCCGACACCCGCCAACACCCGCTGACGC<br>GCCCTGACGGGCTTGTCTGCTCCCGGCATCCGCTTACAGACAAGCTGTGACCGTC<br>TCCGGGAGCTGCATGTGTCAGAGGTTTTCACCGTCATCACCGAAACGCGCGAGAC<br>GAAAGGGCCTCGTGATACGCCTATTTTTATAGGTTAATGTCATGATAATAATGGTTT<br>CTTAGACGTCAGGTGGCACTTTTCGGGGAAATGTGCGCGGAACCCCTATTTGTTTA<br>TTTTTCTAAATACATTCAAATATGTATCCGCTCATGAGACAATAACCCTGATAAATGC<br>TTCAATAATATTGAAAAAGGAAGAGTATGAGTATTCAACATTTCCGTGTCGCCCTTA<br>TTCCCTTTTTTGCGGCATTTTGCCTTCCTGTTTTTGCTCACCCAGAAACGCTGGTGA<br>AAGTAAAAGATGCTGAAGATCAGTTGGGTGCACGAGTGGGTTACATCGAACTGGAT<br>CTCAACAGCGGTAAGATCCTTGAGAGTTTTCGCCCCGAAGAACGTTTTCCAATGAT

	GAGCACTTTTAAAGTTCTGCTATGTGGCGCGGTATTATCCCGTATTGACGCCGGGC<br>AAGAGCAACTCGGTCGCCGCATACACTATTCTCAGAATGACTTGGTTGAGTACTCA<br>CCAGTCACAGAAAAGCATCTTACGGATGGCATGACAGTAAGAGAATTATGCAGTGC<br>TGCCATAACCATGAGTGATAACACTGCGGCCAACTTACTTCTGACAACGATCGGAG<br>GACCGAAGGAGCTAACCGCTTTTTTGCACAACATGGGGGATCATGTAACTCGCCTT<br>GATCGTTGGGAACCGGAGCTGAATGAAGCCATACCAAACGACGAGCGTGACACCA<br>CGATGCCTGTAGCAATGGCAACAACGTTGCGCAAACTATTAACTGGCGAACTACTT<br>ACTCTAGCTTCCCGGCAACAATTAATAGACTGGATGGAGGCGGATAAAGTTGCAGG<br>ACCACTTCTGCGCTCGGCCCTTCCGGCTGGCTGGTTTATTGCTGATAAATCTGGAG<br>CCGGTGAGCGTGGGTCTCGCGGTATCATTGCAGCACTGGGGCCAGATGGTAAGC<br>CCTCCCGTATCGTAGTTATCTACACGACGGGGAGTCAGGCAACTATGGATGAACG<br>AAATAGACAGATCGCTGAGATAGGTGCCTCACTGATTAAGCATTGGTAACTGTCAG<br>ACCAAGTTTACTCATATATACTTTAGATTGATTTAAAACTTCATTTTTAATTTAAAAGG<br>ATCTAGGTGAAGATCCTTTTTGATAATCTCATGACCAAAATCCCTTAACGTGAGTTT<br>TCGTTCCACTGAGCGTCAGACCCCGTAGAAAAGATCAAAGGATCTTCTTGAGATCC<br>TTTTTTTCTGCGCGTAATCTGCTGCTTGCAAACAAAAAAACCACCGCTACCAGCGG<br>TGGTTTGTTTGCCGGATCAAGAGCTACCAACTCTTTTTCCGAAGGTAACTGGCTTC<br>AGCAGAGCGCAGATACCAAATACTGTTCTTCTAGTGTAGCCGTAGTTAGGCCACCA<br>CTTCAAGAACTCTGTAGCACCGCCTACATACCTCGCTCTGCTAATCCTGTTACCAG<br>TGGCTGCTGCCAGTGGCGATAAGTCGTGTCTTACCGGGTTGGACTCAAGACGATA<br>GTTACCGGATAAGGCGCAGCGGTCGGGCTGAACGGGGGGTTCGTGCACACAGCC<br>CAGCTTGGAGCGAACGACCTACACCGAACT
pIV004	GAGATACCTACAGCGTGAGCTATGAGAAAGCGCCACGCTTCCCGAAGGGAGAAAG<br>GCGGACAGGTATCCGGTAAGCGGCAGGGTCGGAACAGGAGAGCGCACGAGGGA<br>GCTTCCAGGGGGAAACGCCTGGTATCTTTATAGTCCTGTCGGGTTTCGCCACCTCT<br>GACTTGAGCGTCGATTTTTGTGATGCTCGTCAGGGGGGCGGAGCCTATGGAAAAA<br>CGCCAGCAACGCGGCCTTTTTACGGTTCCTGGCCTTTTGCTGGCCTTTTGCTCACA<br>TGTTCTTTCCTGCGTTATCCCCTGATTCTGTGGATAACCGTATTACCGCCTTTGAGT<br>GAGCTGATACCGCTCGCCGCAGCCGAACGACCGAGCGCAGCGAGTCAGTGAGCG<br>AGGAAGCGGAAGAGCGCCCAATACGCAAACCGCCTCTCCCCGCGCGTTGGCCGA<br>TTCATTAATGCAGCTGGCACGACAGGTTTCCCGACTGGAAAGCGGGCAGTGAGCG<br>CAACGCAATTAATGTGAGTTAGCTCACTCATTAGGCACCCCAGGCTTTACACTTTAT<br>GCTTCCGGCTCGTATGTTGTGTGGAATTGTGAGCGGATAACAATTTCACACAGGAA<br>ACAGCTATGACCATGATTACGCCAAGCTTGCATGCCTGCAGGTCGACTCTAGAaaag<br>agggtctgtccagcgaataagaagaaaacctccttttcggcttttgaagataggttgcagttgtctgcgggcacaaaatggg<br>caatttttttaatactttttacgtatgagacaagatttttttcgccaattatatcgcatgaagaataaccagagtttttctccgaacgt<br>taagggagttgaagtaaaaataaagaaaggaccaaatgagaatgggtatgcttggtcttagtcttcgaatcaaattctgctt<br>ccctgttcatggcaacgtcacctcaattatttggaaagggggggttttccgactttatttgagatgacttgagatgtgtgtcaatg<br>ctagtattttggagattaatctcagtacaaaacaatattaaaaagaggtgaattatttttccccccttattttttttttgttagaattga<br>tccaaatgtaaataaacaatcacaaggaaaaaaaaaaaaaaaaaaaaatagccgccatgaccccGACATGGAG<br>GCCCAGAATACCCTCCTTGACAGTCTTGACGTGCGCAGCTCAGGGGCATGATGTG<br>ACTGTCGCCCGTACATTTAGCCCATACATCCCCATGTATAATCATTTGCATCCATAC<br>ATTTTGATGGCCGCACGGCGCGAAGCAAAAATTACGGCTCCTCGCTGCGGACCTG<br>CGAGCAGGGAAACGCTCCCCTCACAGACGCGTTGAATTGTCCCCACGCCGCGCC<br>CCTGTAGAGAAATATAAAAGGTTAGGATTTGCCACTGAGGTTCTTCTTTCATATACT<br>TCCTTTTAAAATCTTGCTAGGATACAGTTCTCACATCACATCCGAACATAAACAACC<br>ATGGGTAAAAAGCCTGAACTCACCGCGACGTCTGTCGAGAAGTTTCTGATCGAAAA<br>GTTCGACAGCGTCTCCGACCTGATGCAGCTCTCGGAGGGCGAAGAATCTCGTGCT<br>TTCAGCTTCGATGTAGGAGGGCGTGGATATGTCCTGCGGGTAAATAGCTGCGCCG<br>ATGGTTTCTACAAAGATCGTTATGTTTATCGGCACTTTGCATCGGCCGCGCTCCCG<br>ATTCCGGAAGTGCTTGACATTGGGGAGTTCAGCGAGAGCCTGACCTATTGCATCTC<br>CCGCCGTGCACAGGGTGTCACGTTGCAAGACCTGCCTGAAACCGAACTGCCCGCT<br>GTTCTGCAGCCGGTCGCGGAGGCCATGGATGCGATCGCTGCGGCCGATCTTAGC<br>CAGACGAGCGGGTTCGGCCCATTCGGACCGCAAGGAATCGGTCAATACACTACAT<br>GGCGTGATTTCATATGCGCGATTGCTGATCCCCATGTGTATCACTGGCAAACTGTG<br>ATGGACGACACCGTCAGTGCGTCCGTCGCGCAGGCTCTCGATGAGCTGATGCTTT<br>GGGCCGAGGACTGCCCCGAAGTCCGGCACCTCGTGCACGCGGATTTCGGCTCCA<br>ACAATGTCCTGACGGACAATGGCCGCATAACAGCGGTCATTGACTGGAGCGAGGC<br>GATGTTCGGGGATTCCCAATACGAGGTCGCCAACATCTTCTTCTGGAGGCCGTGG<br>TTGGCTTGTATGGAGCAGCAGACGCGCTACTTCGAGCGGAGGCATCCGGAGCTTG<br>CAGGATCGCCGCGGCTCCGGGCGTATATGCTCCGCATTGGTCTTGACCAACTCTA

	TCAGAGCTTGGTTGACGGCAATTTCGATGATGCAGCTTGGGCGCAGGGTCGATGC<br>GACGCAATCGTCCGATCCGGAGCCGGGACTGTCGGGCGTACACAAATCGCCCGC<br>AGAAGCGCGGCCGTCTGGACCGATGGCTGTGTAGAAGTACTCGCCGATAGTGGAA<br>ACCGACGCCCCAGCACTCGTCCGAGGGCAAAGGAATAATCAGTACTGACAATAAA<br>AAGATTCTTGTTTTCAAGAACTTGTCATTTGTATAGTTTTTTTATATTGTAGTTGTTCT<br>ATTTTAATCAAATGTTAGCGTGATTTATATTTTTTTTCGCCTCGACATCATCTGCCCA<br>GATGCGAAGTTAAGTGCGCAGAAAGTAATATCATGCGTCAATCGTATGTGAATGCT<br>GGTCGCTATACTGCCATGGGCTAGCCTCGAGAGATCTGCGGCCGCagcgccctggtcaa<br>acttcagaactaaaaaaataataaggaagaaaaaaatagctaatttttccggcagaaagattttcgctacccgaaagttttt<br>ccggcaagctaaatggaaaaaggaaagattattgaaagagaaagaaagaaaaaaaaaaatgtacacccagacatc<br>gggcttccacaatttcggctctattgttttccatctctcgcaacggcgggattcctctatggcgtgtgatgtctgtatctgttactta<br>atccagaaactggcacttgacccaactctgccacgtgggtcgttttgccatcgacagattgggagattttcatagtagaattc<br>agcatgatagctacgtaaatgtgttccgcaccgtcacaaagtgttttctactgttctttcttctttcgttcattcagttgagttgagtg<br>agtgctttgttcaatggatcttagctaaaatgcatatttttctcttggtaaatgaatgcttgtgatgGGATCCCCGGGTA<br>CCGAGCTCGAATTCACTGGCCGTCGTTTTACAACGTCGTGACTGGGAAAACCCTG<br>GCGTTACCCAACTTAATCGCCTTGCAGCACATCCCCCTTTCGCCAGCTGGCGTAAT<br>AGCGAAGAGGCCCGCACCGATCGCCCTTCCCAACAGTTGCGCAGCCTGAATGGC<br>GAATGGCGCCTGATGCGGTATTTTCTCCTTACGCATCTGTGCGGTATTTCACACCG<br>CATATGGTGCACTCTCAGTACAATCTGCTCTGATGCCGCATAGTTAAGCCAGCCCC<br>GACACCCGCCAACACCCGCTGACGCGCCCTGACGGGCTTGTCTGCTCCCGGCAT<br>CCGCTTACAGACAAGCTGTGACCGTCTCCGGGAGCTGCATGTGTCAGAGGTTTTC<br>ACCGTCATCACCGAAACGCGCGAGACGAAAGGGCCTCGTGATACGCCTATTTTTAT<br>AGGTTAATGTCATGATAATAATGGTTTCTTAGACGTCAGGTGGCACTTTTCGGGGA<br>AATGTGCGCGGAACCCCTATTTGTTTATTTTTCTAAATACATTCAAATATGTATCCGC<br>TCATGAGACAATAACCCTGATAAATGCTTCAATAATATTGAAAAAGGAAGAGTATGA<br>GTATTCAACATTTCCGTGTCGCCCTTATTCCCTTTTTTGCGGCATTTTGCCTTCCTG<br>TTTTTGCTCACCCAGAAACGCTGGTGAAAGTAAAAGATGCTGAAGATCAGTTGGGT<br>GCACGAGTGGGTTACATCGAACTGGATCTCAACAGCGGTAAGATCCTTGAGAGTTT<br>TCGCCCCGAAGAACGTTTTCCAATGATGAGCACTTTTAAAGTTCTGCTATGTGGCG<br>CGGTATTATCCCGTATTGACGCCGGGCAAGAGCAACTCGGTCGCCGCATACACTA<br>TTCTCAGAATGACTTGGTTGAGTACTCACCAGTCACAGAAAAGCATCTTACGGATG<br>GCATGACAGTAAGAGAATTATGCAGTGCTGCCATAACCATGAGTGATAACACTGCG<br>GCCAACTTACTTCTGACAACGATCGGAGGACCGAAGGAGCTAACCGCTTTTTTGCA<br>CAACATGGGGGATCATGTAACTCGCCTTGATCGTTGGGAACCGGAGCTGAATGAA<br>GCCATACCAAACGACGAGCGTGACACCACGATGCCTGTAGCAATGGCAACAACGT<br>TGCGCAAACTATTAACTGGCGAACTACTTACTCTAGCTTCCCGGCAACAATTAATAG<br>ACTGGATGGAGGCGGATAAAGTTGCAGGACCACTTCTGCGCTCGGCCCTTCCGGC<br>TGGCTGGTTTATTGCTGATAAATCTGGAGCCGGTGAGCGTGGGTCTCGCGGTATC<br>ATTGCAGCACTGGGGCCAGATGGTAAGCCCTCCCGTATCGTAGTTATCTACACGAC<br>GGGGAGTCAGGCAACTATGGATGAACGAAATAGACAGATCGCTGAGATAGGTGCC<br>TCACTGATTAAGCATTGGTAACTGTCAGACCAAGTTTACTCATATATACTTTAGATTG<br>ATTTAAAACTTCATTTTTAATTTAAAAGGATCTAGGTGAAGATCCTTTTTGATAATCT<br>CATGACCAAAATCCCTTAACGTGAGTTTTCGTTCCACTGAGCGTCAGACCCCGTAG<br>AAAAGATCAAAGGATCTTCTTGAGATCCTTTTTTTCTGCGCGTAATCTGCTGCTTGC<br>AAACAAAAAAACCACCGCTACCAGCGGTGGTTTGTTTGCCGGATCAAGAGCTACCA<br>ACTCTTTTTCCGAAGGTAACTGGCTTCAGCAGAGCGCAGATACCAAATACTGTTCTT<br>CTAGTGTAGCCGTAGTTAGGCCACCACTTCAAGAACTCTGTAGCACCGCCTACATA<br>CCTCGCTCTGCTAATCCTGTTACCAGTGGCTGCTGCCAGTGGCGATAAGTCGTGTC<br>TTACCGGGTTGGACTCAAGACGATAGTTACCGGATAAGGCGCAGCGGTCGGGCTG<br>AACGGGGGGTTCGTGCACACAGCCCAGCTTGGAGCGAACGACCTACACCGAACT
pIV005	GAGATACCTACAGCGTGAGCTATGAGAAAGCGCCACGCTTCCCGAAGGGAGAAAG<br>GCGGACAGGTATCCGGTAAGCGGCAGGGTCGGAACAGGAGAGCGCACGAGGGA<br>GCTTCCAGGGGGAAACGCCTGGTATCTTTATAGTCCTGTCGGGTTTCGCCACCTCT<br>GACTTGAGCGTCGATTTTTGTGATGCTCGTCAGGGGGGCGGAGCCTATGGAAAAA<br>CGCCAGCAACGCGGCCTTTTTACGGTTCCTGGCCTTTTGCTGGCCTTTTGCTCACA<br>TGTTCTTTCCTGCGTTATCCCCTGATTCTGTGGATAACCGTATTACCGCCTTTGAGT<br>GAGCTGATACCGCTCGCCGCAGCCGAACGACCGAGCGCAGCGAGTCAGTGAGCG<br>AGGAAGCGGAAGAGCGCCCAATACGCAAACCGCCTCTCCCCGCGCGTTGGCCGA<br>TTCATTAATGCAGCTGGCACGACAGGTTTCCCGACTGGAAAGCGGGCAGTGAGCG<br>CAACGCAATTAATGTGAGTTAGCTCACTCATTAGGCACCCCAGGCTTTACACTTTAT<br>GCTTCCGGCTCGTATGTTGTGTGGAATTGTGAGCGGATAACAATTTCACACAGGAA

	ACAGCTATGACCATGATTACGCCAAGCTTGCATGCCTGCAGGTCGACTCTAGAgcgc<br>taattacatttgatgaaaggttatcttttaaagccgctaacgatcaagatatggtggattggataataaatttcaagagtggaat<br>tttgataagaaaaaaattaaaggccgaaaatatataagcaatgccaatgatgaatatgtattgacttttttttctttgtactatta<br>aataatacgtgtaattgttttccttacgtctagttaaaaaactatttatttatttgtattttaatgataataagcactttttcctcgctga<br>aaattaagggaaaggaccaaatacaaaagtgattagaaggataacgagtagagaaaaagggaagtaaaagttaaaa<br>actagaatcctagtatgacatgcattgcccaatttgccaccatagagcgcatgtagtgtactgtgcacattgtatcaatacga<br>gcccaagtctgctactgaagctaaaactagatttaattttattaaaggatgagaataaagaacttaacgggaaCAACTG<br>TGGGAATACTCAGGTATCGTAAGATGCAAGAGTTCGAATCTCTTAGCAACCATTATT<br>TTTTTCCTCAACATAACGAGAACACACAGGGGCGCTATCGCACAGAATCAAATTCG<br>ATGACTGGAAATTTTTTGTTAATTTCAGAGGTCGCCTGACGCATATACCTTTTTCAA<br>CTGAAAAATTGGGAGAAAAAGGAAAGGTGAGAGCGCCGGAACCGGCTTTTCATAT<br>AGAATAGAGAAGCGTTCATGACTAAATGCTTGCATCACAATACTTGAAGTTGACAAT<br>ATTATTTAAGGACCTATTGTTTTTTCCAATAGGTGGTTAGCAATCGTCTTACTTTCTA<br>ACTTTTCTTACCTTTTACATTTCAGCAATATATATATATATATATTTCAAGGATATACCAT<br>TCTAATGTCTGCCCCTAAGAAGATCGTCGTTTTGCCAGGTGACCACGTTGGTCAAG<br>AAATCACAGCCGAAGCCATTAAGGTTCTTAAAGCTATTTCTGATGTTCGTTCCAATG<br>TCAAGTTCGATTTCGAAAATCATTTAATTGGTGGTGCTGCTATCGATGCTGCAGGTA<br>TTCCACTTCCAGATGAGGCGCTGGAAGCCTCCAAGAAGGCTGATGCCGTTTTGTTA<br>GGTGCTGTGGGTGGTCCTAAATGGGGTACCGGTAGTGTTAGACCTGAACAAGGTT<br>TACTAAAAATCCGTAAAGAACTTCAATTGTACGCCAACTTAAGACCATGTAACTTTG<br>CATCCGACTCTCTTTTAGACTTATCTCCAATCAAGCCACAATTTGCTAAAGGTACTG<br>ACTTCGTTGTTGTCAGAGAATTAGTGGGAGGTATTTACTTTGGTAAGAGAAAGGAA<br>GACGATGGTGATGGTGTCGCTTGGGATAGTGAACAATACACCGTTCCAGAAGTGC<br>AAAGAATCACAAGAATGGCCGCTTTCATGGCCCTACAACATGAGCCACCATTGCCT<br>ATTTGGTCCTTGGATAAAGCTAATGTTTTGGCCTCTTCAAGATTATGGAGAAAAACT<br>GTGGAGGAAACCATCAAGAACGAATTCCCTACATTGAAGGTTCAACATCAATTGATT<br>GATTCTGCCGCCATGATCCTAGTTAAGAACCCAACCCACCTAAATGGTATTATAATC<br>ACCAGCAACATGTTTGGTGATATCATCTCCGATGAAGCCTCCGTTATCCCAGGTTC<br>CTTGGGTTTGTTGCCATCTGCGTCCTTGGCCTCTTTGCCAGACAAGAACACCGCAT<br>TTGGTTTGTACGAACCATGCCACGGTTCTGCTCCAGATTTGCCAAAGAATAAGGTC<br>AACCCTATCGCCACTATCTTGTCTGCTGCAATGATGTTGAAATTGTCATTGAACTTG<br>CCTGAAGAAGGTAAGGCCATTGAAGATGCAGTTAAAAAGGTTTTGGATGCAGGTAT<br>CAGAACTGGTGATTTAGGTGGTTCCAACAGTACCACCGAAGTCGGTGATGCTGTC<br>GCCGAAGAAGTTAAGAAAATCCTTGCTTAAAAAGATTCTCTTTTTTTATGATATTTGT<br>ACATAAACTTTATAAATGAAATTCATAATAGAAACGACACGAAATTACAAAATGGAAT<br>ATGTTCATAGGGTAGACGAAACTATATACGCAATCTACATACATTTATCAAGAAGGA<br>GAAAAAGGAGGATGTAAAGGAATACAGGTAAGCAAATTGATACTAATGGCTCAACG<br>TGATAAGGAAAAAGAATTGCACTTTAACATTAATATTGACAAGGAGGAGGGCACCA<br>CACAAAAAGTTAGGTGTAACAGAAAATCATGAAACTATGATTCCTAATTTATATATTG<br>GAGGATTTTCTCTAAAAAAAAAAAAAATACAACAAATAAAAAACACTCAATGACCTGA<br>CCATTTGATGGAGTTTAAGTCAATACCTTCTTGAACCATTTCCCATAATGGTGAAAG<br>TTCCCTCAAGAATTTTACTCTGTCAGAAACGGCCTTAACGACGTAGTCGATATGGT<br>GCACTCTCAGTACAATCTGCTCTGATGCCGCATAGTTAAGCCAGCCCCGACACCC<br>GCCAACACCCGCTGACGCGCCCTGACGGGCTTGTCTGCTCCCGGCATCCCCATG<br>GGCTAGCCTCGAGAGATCTGCGGCCGCatggagaaaaagaaagatcccctgatgaatagtctcatg<br>aagttagatgttttacgaatgaaaaagaataataacctgattaggcacaggatagaacagctaaatgagcggatttatagt<br>aaaaggaatcacatcagcgaattgaaagtagaaattgacaattataaatgttataaggtgggtactggtacggacaaatta<br>agagagcaagtggaaattagtgatgcaaaaaataagctggcacaagtatcgaagatttgtgaatcggctagagattaca<br>agctcaatctactaaataattggtttgtgatccagaagctgcaagataatttccaaatacctttcgctatagcttttcagccacta<br>atatctttgaaaaacttccgtattttacctcttgctataacaaacgattccatcaacatcatgtggaagtatattagcttcttctcag<br>acattcttatgattaaacttccctacacaaataaaatctgtgaacaGGATCCCCGGGTACCGAGCTCGAAT<br>TCACTGGCCGTCGTTTTACAACGTCGTGACTGGGAAAACCCTGGCGTTACCCAACT<br>TAATCGCCTTGCAGCACATCCCCCTTTCGCCAGCTGGCGTAATAGCGAAGAGGCC<br>CGCACCGATCGCCCTTCCCAACAGTTGCGCAGCCTGAATGGCGAATGGCGCCTGA<br>TGCGGTATTTTCTCCTTACGCATCTGTGCGGTATTTCACACCGCATATGGTGCACT<br>CTCAGTACAATCTGCTCTGATGCCGCATAGTTAAGCCAGCCCCGACACCCGCCAA<br>CACCCGCTGACGCGCCCTGACGGGCTTGTCTGCTCCCGGCATCCGCTTACAGACA<br>AGCTGTGACCGTCTCCGGGAGCTGCATGTGTCAGAGGTTTTCACCGTCATCACCG<br>AAACGCGCGAGACGAAAGGGCCTCGTGATACGCCTATTTTTATAGGTTAATGTCAT<br>GATAATAATGGTTTCTTAGACGTCAGGTGGCACTTTTCGGGGAAATGTGCGCGGAA<br>CCCCTATTTGTTTATTTTTCTAAATACATTCAAATATGTATCCGCTCATGAGACAATA

	ACCCTGATAAATGCTTCAATAATATTGAAAAAGGAAGAGTATGAGTATTCAACATTT<br>CCGTGTCGCCCTTATTCCCTTTTTTGCGGCATTTTGCCTTCCTGTTTTTGCTCACCC<br>AGAAACGCTGGTGAAAGTAAAAGATGCTGAAGATCAGTTGGGTGCACGAGTGGGT<br>TACATCGAACTGGATCTCAACAGCGGTAAGATCCTTGAGAGTTTTCGCCCCGAAGA<br>ACGTTTTCCAATGATGAGCACTTTTAAAGTTCTGCTATGTGGCGCGGTATTATCCCG<br>TATTGACGCCGGGCAAGAGCAACTCGGTCGCCGCATACACTATTCTCAGAATGACT<br>TGGTTGAGTACTCACCAGTCACAGAAAAGCATCTTACGGATGGCATGACAGTAAGA<br>GAATTATGCAGTGCTGCCATAACCATGAGTGATAACACTGCGGCCAACTTACTTCT<br>GACAACGATCGGAGGACCGAAGGAGCTAACCGCTTTTTTGCACAACATGGGGGAT<br>CATGTAACTCGCCTTGATCGTTGGGAACCGGAGCTGAATGAAGCCATACCAAACGA<br>CGAGCGTGACACCACGATGCCTGTAGCAATGGCAACAACGTTGCGCAAACTATTAA<br>CTGGCGAACTACTTACTCTAGCTTCCCGGCAACAATTAATAGACTGGATGGAGGCG<br>GATAAAGTTGCAGGACCACTTCTGCGCTCGGCCCTTCCGGCTGGCTGGTTTATTG<br>CTGATAAATCTGGAGCCGGTGAGCGTGGGTCTCGCGGTATCATTGCAGCACTGGG<br>GCCAGATGGTAAGCCCTCCCGTATCGTAGTTATCTACACGACGGGGAGTCAGGCA<br>ACTATGGATGAACGAAATAGACAGATCGCTGAGATAGGTGCCTCACTGATTAAGCA<br>TTGGTAACTGTCAGACCAAGTTTACTCATATATACTTTAGATTGATTTAAAACTTCAT<br>TTTTAATTTAAAAGGATCTAGGTGAAGATCCTTTTTGATAATCTCATGACCAAAATCC<br>CTTAACGTGAGTTTTCGTTCCACTGAGCGTCAGACCCCGTAGAAAAGATCAAAGGA<br>TCTTCTTGAGATCCTTTTTTTCTGCGCGTAATCTGCTGCTTGCAAACAAAAAAACCA<br>CCGCTACCAGCGGTGGTTTGTTTGCCGGATCAAGAGCTACCAACTCTTTTTCCGAA<br>GGTAACTGGCTTCAGCAGAGCGCAGATACCAAATACTGTTCTTCTAGTGTAGCCGT<br>AGTTAGGCCACCACTTCAAGAACTCTGTAGCACCGCCTACATACCTCGCTCTGCTA<br>ATCCTGTTACCAGTGGCTGCTGCCAGTGGCGATAAGTCGTGTCTTACCGGGTTGG<br>ACTCAAGACGATAGTTACCGGATAAGGCGCAGCGGTCGGGCTGAACGGGGGGTT<br>CGTGCACACAGCCCAGCTTGGAGCGAACGACCTACACCGAACT
pIV006	GAGATACCTACAGCGTGAGCTATGAGAAAGCGCCACGCTTCCCGAAGGGAGAAAG<br>GCGGACAGGTATCCGGTAAGCGGCAGGGTCGGAACAGGAGAGCGCACGAGGGA<br>GCTTCCAGGGGGAAACGCCTGGTATCTTTATAGTCCTGTCGGGTTTCGCCACCTCT<br>GACTTGAGCGTCGATTTTTGTGATGCTCGTCAGGGGGGCGGAGCCTATGGAAAAA<br>CGCCAGCAACGCGGCCTTTTTACGGTTCCTGGCCTTTTGCTGGCCTTTTGCTCACA<br>TGTTCTTTCCTGCGTTATCCCCTGATTCTGTGGATAACCGTATTACCGCCTTTGAGT<br>GAGCTGATACCGCTCGCCGCAGCCGAACGACCGAGCGCAGCGAGTCAGTGAGCG<br>AGGAAGCGGAAGAGCGCCCAATACGCAAACCGCCTCTCCCCGCGCGTTGGCCGA<br>TTCATTAATGCAGCTGGCACGACAGGTTTCCCGACTGGAAAGCGGGCAGTGAGCG<br>CAACGCAATTAATGTGAGTTAGCTCACTCATTAGGCACCCCAGGCTTTACACTTTAT<br>GCTTCCGGCTCGTATGTTGTGTGGAATTGTGAGCGGATAACAATTTCACACAGGAA<br>ACAGCTATGACCATGATTACGCCAAGCTTGCATGCCTGCAGGTCGACTCTAGAcaac<br>gcaaaataaataattaatatgtaaaatatctgtttacttgaaatttacccttaacagaggtactaacgggaggaaggactgg<br>ccttgctccggtagacctcgatgcctgaaccttctccctcatctcgaaacggtacgacctctgagaaaacgatatcaatcaa<br>tgcgcgtcttccagatgaaattcttcggaatgcccataatacgaaaaactaacgatggaaaaaatactataaatgtatgggt<br>gtaattttaaccgaatgtatggatgttacagggagctttatatatgtagaaaattccagacgttccaaacaaataagtgaaaa<br>tcaacacgattgggaaagtacatgccagagttcgatgtttaggttaatgataaaaataatttttctctaatcaccctccattttcg<br>ataaaatatcagcgtagtctgaaaatatatacattaagcaaaaagaatgtcaaacaagtttcactgtgacgtttgttcagcC<br>GCTTAACTATGCGGCATCAGAGCAGATTGTACTGAGAGTGCACCATAGATCAACGA<br>CATTACTATATATATAATATAGGAAGCATTTAATAGAACAGCATCGTAATATATGTGT<br>ACTTTGCAGTTATGACGCCAGATGGCAGTAGTGGAAGATATTCTTTATTGAAAAATA<br>GCTTGTCACCTTACGTACAATCTTGATCCGGAGCTTTTCTTTTTTTGCCGATTAAGA<br>ATTAATTCGGTCGAAAAAAGAAAAGGAGAGGGCCAAGAGGGAGGGCATTGGTGAC<br>TATTGAGCACGTGAGTATACGTGATTAAGCACACAAAGGCAGCTTGGAGTATGTCT<br>GTTATTAATTTCACAGGTAGTTCTGGTCCATTGGTGAAAGTTTGCGGCTTGCAGAG<br>CACAGAGGCCGCAGAATGTGCTCTAGATTCCGATGCTGACTTGCTGGGTATTATAT<br>GTGTGCCCAATAGAAAGAGAACAATTGACCCGGTTATTGCAAGGAAAATTTCAAGT<br>CTTGTAAAAGCATATAAAAATAGTTCAGGCACTCCGAAATACTTGGTTGGCGTGTTT<br>CGTAATCAACCTAAGGAGGATGTTTTGGCTCTGGTCAATGATTACGGCATTGATAT<br>CGTCCAACTGCATGGAGATGAGTCGTGGCAAGAATACCAAGAGTTCCTCGGTTTG<br>CCAGTTATTAAAAGACTCGTATTTCCAAAAGACTGCAACATACTACTCAGTGCAGCT<br>TCACAGAAACCTCATTCGTTTATTCCCTTGTTTGATTCAGAAGCAGGTGGGACAGG<br>TGAACTTTTGGATTGGAACTCGATTTCTGACTGGGTTGGAAGGCAAGAGAGCCCCG<br>AAAGCTTACATTTTATGTTAGCTGGTGGACTGACGCCAGAAAATGTTGGTGATGCG<br>CTTAGATTAAATGGCGTTATTGGTGTTGATGTAAGCGGAGGTGTGGAGACAAATGG

	TGTAAAAGACTCTAACAAAATAGCAAATTTCGTCAAAAATGCTAAGAAATAGGTTATT<br>ACTGAGTAGTATTTATTTAAGTATTGTTTGTGCACTTGCCGATCTATGCGGTGTGAA<br>ATACCGCACAGATGCGTAAGGAGAAAATACCGCATCAGGAAATTGTAAACGTTAAT<br>CCATGGGCTAGCCTCGAGAGATCTGCGGCCGCtacaggaaagcatcgcccttaccatgattatag<br>gataatagagaccaattcataccccattctctgtcctgattggggtgccgatgaagaattgcagttgattaaaggcgcacag<br>actttagggctcggtaattggcaggatattgctgaccatataggcagcagaggcaaagaagaagttaaggaacattacct<br>aaaatattatctggaaagcaaatactatccaatacctgatattacccaaaatatacatgtcccacaagatgaatttttggaac<br>agcgaaggcatagaatcgagtccttccgggagaggccgctagagcctccaagaaagcccatggcatcggttcctagct<br>gccatgaagtacaggggtttatgccgggcagattagaatttgaaacagaattcgaaaatgaagctgaaggtcccgtaaa<br>ggatatggtttttgaacctgatgaccagcccttagacattgaattgaagtttgccatcttggacaGGATCCCCGGGTA<br>CCGAGCTCGAATTCACTGGCCGTCGTTTTACAACGTCGTGACTGGGAAAACCCTG<br>GCGTTACCCAACTTAATCGCCTTGCAGCACATCCCCCTTTCGCCAGCTGGCGTAAT<br>AGCGAAGAGGCCCGCACCGATCGCCCTTCCCAACAGTTGCGCAGCCTGAATGGC<br>GAATGGCGCCTGATGCGGTATTTTCTCCTTACGCATCTGTGCGGTATTTCACACCG<br>CATATGGTGCACTCTCAGTACAATCTGCTCTGATGCCGCATAGTTAAGCCAGCCCC<br>GACACCCGCCAACACCCGCTGACGCGCCCTGACGGGCTTGTCTGCTCCCGGCAT<br>CCGCTTACAGACAAGCTGTGACCGTCTCCGGGAGCTGCATGTGTCAGAGGTTTTC<br>ACCGTCATCACCGAAACGCGCGAGACGAAAGGGCCTCGTGATACGCCTATTTTTAT<br>AGGTTAATGTCATGATAATAATGGTTTCTTAGACGTCAGGTGGCACTTTTCGGGGA<br>AATGTGCGCGGAACCCCTATTTGTTTATTTTTCTAAATACATTCAAATATGTATCCGC<br>TCATGAGACAATAACCCTGATAAATGCTTCAATAATATTGAAAAAGGAAGAGTATGA<br>GTATTCAACATTTCCGTGTCGCCCTTATTCCCTTTTTTGCGGCATTTTGCCTTCCTG<br>TTTTTGCTCACCCAGAAACGCTGGTGAAAGTAAAAGATGCTGAAGATCAGTTGGGT<br>GCACGAGTGGGTTACATCGAACTGGATCTCAACAGCGGTAAGATCCTTGAGAGTTT<br>TCGCCCCGAAGAACGTTTTCCAATGATGAGCACTTTTAAAGTTCTGCTATGTGGCG<br>CGGTATTATCCCGTATTGACGCCGGGCAAGAGCAACTCGGTCGCCGCATACACTA<br>TTCTCAGAATGACTTGGTTGAGTACTCACCAGTCACAGAAAAGCATCTTACGGATG<br>GCATGACAGTAAGAGAATTATGCAGTGCTGCCATAACCATGAGTGATAACACTGCG<br>GCCAACTTACTTCTGACAACGATCGGAGGACCGAAGGAGCTAACCGCTTTTTTGCA<br>CAACATGGGGGATCATGTAACTCGCCTTGATCGTTGGGAACCGGAGCTGAATGAA<br>GCCATACCAAACGACGAGCGTGACACCACGATGCCTGTAGCAATGGCAACAACGT<br>TGCGCAAACTATTAACTGGCGAACTACTTACTCTAGCTTCCCGGCAACAATTAATAG<br>ACTGGATGGAGGCGGATAAAGTTGCAGGACCACTTCTGCGCTCGGCCCTTCCGGC<br>TGGCTGGTTTATTGCTGATAAATCTGGAGCCGGTGAGCGTGGGTCTCGCGGTATC<br>ATTGCAGCACTGGGGCCAGATGGTAAGCCCTCCCGTATCGTAGTTATCTACACGAC<br>GGGGAGTCAGGCAACTATGGATGAACGAAATAGACAGATCGCTGAGATAGGTGCC<br>TCACTGATTAAGCATTGGTAACTGTCAGACCAAGTTTACTCATATATACTTTAGATTG<br>ATTTAAAACTTCATTTTTAATTTAAAAGGATCTAGGTGAAGATCCTTTTTGATAATCT<br>CATGACCAAAATCCCTTAACGTGAGTTTTCGTTCCACTGAGCGTCAGACCCCGTAG<br>AAAAGATCAAAGGATCTTCTTGAGATCCTTTTTTTCTGCGCGTAATCTGCTGCTTGC<br>AAACAAAAAAACCACCGCTACCAGCGGTGGTTTGTTTGCCGGATCAAGAGCTACCA<br>ACTCTTTTTCCGAAGGTAACTGGCTTCAGCAGAGCGCAGATACCAAATACTGTTCTT<br>CTAGTGTAGCCGTAGTTAGGCCACCACTTCAAGAACTCTGTAGCACCGCCTACATA<br>CCTCGCTCTGCTAATCCTGTTACCAGTGGCTGCTGCCAGTGGCGATAAGTCGTGTC<br>TTACCGGGTTGGACTCAAGACGATAGTTACCGGATAAGGCGCAGCGGTCGGGCTG<br>AACGGGGGGTTCGTGCACACAGCCCAGCTTGGAGCGAACGACCTACACCGAACT
pIV007	GAGATACCTACAGCGTGAGCTATGAGAAAGCGCCACGCTTCCCGAAGGGAGAAAG<br>GCGGACAGGTATCCGGTAAGCGGCAGGGTCGGAACAGGAGAGCGCACGAGGGA<br>GCTTCCAGGGGGAAACGCCTGGTATCTTTATAGTCCTGTCGGGTTTCGCCACCTCT<br>GACTTGAGCGTCGATTTTTGTGATGCTCGTCAGGGGGGCGGAGCCTATGGAAAAA<br>CGCCAGCAACGCGGCCTTTTTACGGTTCCTGGCCTTTTGCTGGCCTTTTGCTCACA<br>TGTTCTTTCCTGCGTTATCCCCTGATTCTGTGGATAACCGTATTACCGCCTTTGAGT<br>GAGCTGATACCGCTCGCCGCAGCCGAACGACCGAGCGCAGCGAGTCAGTGAGCG<br>AGGAAGCGGAAGAGCGCCCAATACGCAAACCGCCTCTCCCCGCGCGTTGGCCGA<br>TTCATTAATGCAGCTGGCACGACAGGTTTCCCGACTGGAAAGCGGGCAGTGAGCG<br>CAACGCAATTAATGTGAGTTAGCTCACTCATTAGGCACCCCAGGCTTTACACTTTAT<br>GCTTCCGGCTCGTATGTTGTGTGGAATTGTGAGCGGATAACAATTTCACACAGGAA<br>ACAGCTATGACCATGATTACGCCAAGCTTGCATGCCTGCAGGTCGACTCTAGAaggtt<br>gatgcttctgggttccgtataccacaacaagaagctactaacaaggctcaaagtcaaggtgctctattaaagattcgtttagt<br>gatgtctgctccaatcgatgaaactttcgacaccaacgaaaccgccacaatcacttatttctctactgatttgaacaagattgt<br>cgagaaattcccaaaacaagccgaaaaattgtccgataccttagtgtttttgaaagatccaatgggcaacaacatcacctt

	ctcaggcttagctaatgcaaccgattccgctccaacttccaaagatgctttcttagaagctacctccgaagacgaaatcatct<br>ctagagcttcttccgatgcttctgacttactaagacaaacattgggctcttctcaaaagaagaagaagattgctgtcatgactt<br>ctggtggtgattctccaggtatgaatgccgctgttcgtgccgttgttcgtacaggtatacatttcggctgtgatgtttttCTAGA<br>GGCATCGCACAGTTTTAGCGAGGAAAACTCTTCAATAGTTTTGCCAGCGGAATTCC<br>ACTTGCAATTACATAAAAAATTCCGGCGGTTTTTCGCGTGTGACTCAATGTCGAAAT<br>ACCTGCCTAATGAACATGAACATCGCCCAAATGTATTTGAAGACCCGCTGGGAGAA<br>GTTCAAGATATATAAGTAACAAGCAGCCAATAGTATAAAAAAAAATCTGAGTTTATTA<br>CCTTTCCTGGAATTTCAGTGAAAAACTGCTAATTATAGAGAGATATCACAGAGTTAC<br>TCACTAATGACTAACGAAAAGGTCTGGATAGAGAAGTTGGATAATCCAACTCTTTCA<br>GTGTTACCACATGACTTTTTACGCCCACAACAAGAACCTTATACGAAACAAGCTACA<br>TATTCGTTACAGCTACCTCAGCTCGATGTGCCTCATGATAGTTTTTCTAACAAATAC<br>GCTGTCGCTTTGAGTGTATGGGCTGCATTGATATATAGAGTAACCGGTGACGATGA<br>TATTGTTCTTTATATTGCGAATAACAAAATCTTAAGATTCAATATTCAACCAACGTGG<br>GTCATTTAATGAGCTGTATTCTACAATTAACAATGAGTTGAACAAGCTCAATTCTATT<br>GAGGCCAATTTTTCCTTTGACGAGCTAGCTGAAAAAATTCAAAGTTGCCAAGATCTG<br>GAAAGGACCCCTCAGTTGTTCCGTTTGGCCTTTTTGGAAAACCAAGATTTCAAATTA<br>GACGAGTTCAAGCATCATTTAGTGGACTTTGCTTTGAATTTGGATACCAGTAATAAT<br>GCGCATGTTTTGAACTTAATTTATAACAGCTTACTGTATTCGAATGAAAGAGTAACC<br>ATTGTTGCGGACCAATTTACTCAATATTTGACTGCTGCGCTAAGCGATCCATCCAAT<br>TGCATAACTAAAATCTCTCTGATCACCGCATCATCCAAGGATAGTTTACCTGATCCA<br>ACTAAGAACTTGGGCTGGTGCGATTTCGTGGGGTGTATTCACGACATTTTCCAGGA<br>CAATGCTGAAGCCTTCCCAGAGAGAACCTGTGTTGTGGAGACTCCAACACTAAATT<br>CCGACAAGTCCCGTTCTTTCACTTATCGCGACATCAACCGCACTTCTAACATAGTTG<br>CCCATTATTTGATTAAAACAGGTATCAAAAGAGGTGATGTAGTGATGATCTATTCTT<br>CTAGGGGTGTGGATTTGATGGTATGTGTGATGGGTGTCTTGAAAGCCGGCGCAAC<br>CTTTTCAGTTATCGACCCTGCATATCCCCCAGCCAGACAAACCATTTACTTAGGTGT<br>TGCTAAACCACGTGGGTTGATTGTTATTAGAGCTGCTGGACAATTGGATCAACTAG<br>TAGAAGATTACATCAATGATGAATTGGAGATTGTTTCAAGAATCAATTCCATCGCTA<br>TTCAAGAAAATGGTACCATTGAAGGTGGCAAATTGGACAATGGCGAGGATGTTTTG<br>GCTCCATATGATCACTACAAAGACACCAGAACAGGTGTTGTAGTTGGACCAGATTC<br>CAACCCAACCCTATCTTTCACATCTGGTTCCGAAGGTATTCCTAAGGGTGTTCTTG<br>GTAGACATTTTTCCTTGGCTTATTATTTCAATTGGATGTCCAAAAGGTTCAACTTAAC<br>AGAAAATGATAAATTCACAATGCTGAGCGGTATTGCACATGATCCAATTCAAAGAGA<br>TATGTTTACACCATTATTTTTAGGTGCCCAATTGTATGTCCCTACTCAAGATGATATT<br>GGTACACCGGGCCGTTTAGCGGAATGGATGAGTAAGTATGGTTGCACAGTTACCC<br>ATTTAACACCTGCCATGGGTCAATTACTTACTGCCCAAGCTACTACACCATTCCCTA<br>AGTTACATCATGCGTTCTTTGTGGGTGACATTTTAACAAAACGTGATTGTCTGAGGT<br>TACAAACCTTGGCAGAAAATTGCCGTATTGTTAATATGTACGGTACCACTGAAACAC<br>AGCGTGCAGTTTCTTATTTCGAAGTTAAATCAAAAAATGACGATCCAAACTTTTTGA<br>AAAAATTGAAAGATGTCATGCCTGCTGGTAAAGGTATGTTGAACGTTCAGCTACTA<br>GTTGTTAACAGGAACGATCGTACTCAAATATGTGGTATTGGCGAAATAGGTGAGAT<br>TTATGTTCGTGCAGGTGGTTTGGCCGAAGGTTATAGAGGATTACCAGAATTGAATA<br>AAGAAAAATTTGTGAACAACTGGTTTGTTGAAAAAGATCACTGGAATTATTTGGATA<br>AGGATAATGGTGAACCTTGGAGACAATTCTGGTTAGGTCCAAGAGATAGATTGTAC<br>AGAACGGGTGATTTAGGTCGTTATCTACCAAACGGTGACTGTGAATGTTGCGGTAG<br>GGCTGATGATCAAGTTAAAATTCGTGGGTTCAGAATCGAATTAGGAGAAATAGATA<br>CGCACATTTCCCAACATCCATTGGTAAGAGAAAACATTACTTTAGTTCGCAAAAATG<br>CCGACAATGAGCCAACATTGATCACATTTATGGTCCCAAGATTTGACAAGCCAGAT<br>GACTTGTCTAAGTTCCAAAGTGATGTTCCAAAGGAGGTTGAAACTGACCCTATAGTT<br>AAGGGCTTAATCGGTTACCATCTTTTATCCAAGGACATCAGGACTTTCTTAAAGAAA<br>AGATTGGCTAGCTATGCTATGCCTTCCTTGATTGTGGTTATGGATAAACTACCATTG<br>AATCCAAATGGTAAAGTTGATAAGCCTAAACTTCAATTCCCAACTCCCAAGCAATTA<br>AATTTGGTAGCTGAAAATACAGTTTCTGAAACTGACGACTCTCAGTTTACCAATGTT<br>GAGCGCGAGGTTAGAGACTTATGGTTAAGTATATTACCTACCAAGCCAGCATCTGT<br>ATCACCAGATGATTCGTTTTTCGATTTAGGTGGTCATTCTATCTTGGCTACCAAAAT<br>GATTTTTACCTTAAAGAAAAAGCTGCAAGTTGATTTACCATTGGGCACAATTTTCAA<br>GTATCCAACGATAAAGGCCTTTGCCGCGGAAATTGACAGAATTAAATCATCGGGTG<br>GATCATCTCAAGGTGAGGTCGTCGAAAATGTCACTGCAAATTATGCGGAAGACGCC<br>AAGAAATTGGTTGAGACGCTACCAAGTTCGTACCCCTCTCGAGAATATTTTGTTGAA<br>CCTAATAGTGCCGAAGGAAAAACAACAATTAATGTGTTTGTTACCGGTGTCACAGG<br>ATTTCTGGGCTCCTACATCCTTGCAGATTTGTTAGGACGTTCTCCAAAGAACTACAG

	TTTCAAAGTGTTTGCCCACGTCAGGGCCAAGGATGAAGAAGCTGCATTTGCAAGAT<br>TACAAAAGGCAGGTATCACCTATGGTACTTGGAACGAAAAATTTGCCTCAAATATTA<br>AAGTTGTATTAGGCGATTTATCTAAAAGCCAATTTGGTCTTTCAGATGAGAAGTGGA<br>TGGATTTGGCAAACACAGTTGATATAATTATCCATAATGGTGCGTTAGTTCACTGGG<br>TTTATCCATATGCCAAATTGAGGGATCCAAATGTTATTTCAACTATCAATGTTATGAG<br>CTTAGCCGCCGTCGGCAAGCCAAAGTTCTTTGACTTTGTTTCCTCCACTTCTACTCT<br>TGACACTGAATACTACTTTAATTTGTCAGATAAACTTGTTAGCGAAGGGAAGCCAGG<br>CATTTTAGAATCAGACGATTTAATGAACTCTGCAAGCGGGCTCACTGGTGGATATG<br>GTCAGTCCAAATGGGCTGCTGAGTACATCATTAGACGTGCAGGTGAAAGGGGCCT<br>ACGTGGGTGTATTGTCAGACCAGGTTACGTAACAGGTGCCTCTGCCAATGGTTCTT<br>CAAACACAGATGATTTCTTATTGAGATTTTTGAAAGGTTCAGTCCAATTAGGTAAGA<br>TTCCAGATATCGAAAATTCCGTGAATATGGTTCCAGTAGATCATGTTGCTCGTGTTG<br>TTGTTGCTACGTCTTTGAATCCTCCCAAAGAAAATGAATTGGCCGTTGCTCAAGTAA<br>CGGGTCACCCAAGAATATTATTCAAAGACTACTTGTATACTTTACACGATTATGGTT<br>ACGATGTCGAAATCGAAAGCTATTCTAAATGGAAGAAATCATTGGAGGCGTCTGTT<br>ATTGACAGGAATGAAGAAAATGCGTTGTATCCTTTGCTACACATGGTCTTAGACAAC<br>TTACCTGAAAGTACCAAAGCTCCGGAACTAGACGATAGGAACGCCGTGGCATCTTT<br>AAAGAAAGACACCGCATGGACAGGTGTTGATTGGTCTAATGGAATAGGTGTTACTC<br>CAGAAGAGGTTGGTATATATATTGCATTTTTAAACAAGGTTGGATTTTTACCTCCAC<br>CAACTCATAATGACAAACTTCCACTGCCAAGTATAGAACTAACTCAAGCGCAAATAA<br>GTCTAGTTGCTTCAGGTGCTGGTGCTCGTGGAAGCTCCGCAGCAGCTTAAGGTTG<br>AGCATTACGTATGATATGTCCATGTACAATAATTAAATATGAATTAGGAGAAAGACT<br>TAGCTTCTTTTCGGGTGATGTCACTTAAAAACTCCGAGAATAATATATAATAAGAGA<br>ATAAAATATTAGTTATTGAATAAGAACTGTAAATCAGgcatgcCCATGGGCTAGCCTCG<br>AGAGATCTGCGGCCGCttggttaagtgaaggtggtactttgattggtactgctcgttctatggaattcagaaagcgt<br>gagggtcgtagacaagctgcaggcaatttaatttcgcaaggtattgacgctttggttgtttgtggtggtgatggttctttaaccg<br>gtgctgatcttttcagacacgaatggccatctttggttgatgaattggttgcagaaggtagattcactaaagaagaagtcgcc<br>ccatacaagaatttgtccattgttggtcttgtcggttccatcgataatgatatgtctggtactgactctaccattggtgcttattctgc<br>tttggaaagaatctgtgaaatggttgactacattgatgccaccgctaaatcccactcccgtgcctttgttgttgaagttatgggt<br>agacattgtggttggttggccttgatggctggtattgctaccggtgccgattacatttttattccagaaagagctgttcctcacgg<br>aaaatggcaggGGATCCCCGGGTACCGAGCTCGAATTCACTGGCCGTCGTTTTACAAC<br>GTCGTGACTGGGAAAACCCTGGCGTTACCCAACTTAATCGCCTTGCAGCACATCCC<br>CCTTTCGCCAGCTGGCGTAATAGCGAAGAGGCCCGCACCGATCGCCCTTCCCAAC<br>AGTTGCGCAGCCTGAATGGCGAATGGCGCCTGATGCGGTATTTTCTCCTTACGCAT<br>CTGTGCGGTATTTCACACCGCATATGGTGCACTCTCAGTACAATCTGCTCTGATGC<br>CGCATAGTTAAGCCAGCCCCGACACCCGCCAACACCCGCTGACGCGCCCTGACG<br>GGCTTGTCTGCTCCCGGCATCCGCTTACAGACAAGCTGTGACCGTCTCCGGGAGC<br>TGCATGTGTCAGAGGTTTTCACCGTCATCACCGAAACGCGCGAGACGAAAGGGCC<br>TCGTGATACGCCTATTTTTATAGGTTAATGTCATGATAATAATGGTTTCTTAGACGTC<br>AGGTGGCACTTTTCGGGGAAATGTGCGCGGAACCCCTATTTGTTTATTTTTCTAAAT<br>ACATTCAAATATGTATCCGCTCATGAGACAATAACCCTGATAAATGCTTCAATAATAT<br>TGAAAAAGGAAGAGTATGAGTATTCAACATTTCCGTGTCGCCCTTATTCCCTTTTTT<br>GCGGCATTTTGCCTTCCTGTTTTTGCTCACCCAGAAACGCTGGTGAAAGTAAAAGA<br>TGCTGAAGATCAGTTGGGTGCACGAGTGGGTTACATCGAACTGGATCTCAACAGC<br>GGTAAGATCCTTGAGAGTTTTCGCCCCGAAGAACGTTTTCCAATGATGAGCACTTT<br>TAAAGTTCTGCTATGTGGCGCGGTATTATCCCGTATTGACGCCGGGCAAGAGCAAC<br>TCGGTCGCCGCATACACTATTCTCAGAATGACTTGGTTGAGTACTCACCAGTCACA<br>GAAAAGCATCTTACGGATGGCATGACAGTAAGAGAATTATGCAGTGCTGCCATAAC<br>CATGAGTGATAACACTGCGGCCAACTTACTTCTGACAACGATCGGAGGACCGAAG<br>GAGCTAACCGCTTTTTTGCACAACATGGGGGATCATGTAACTCGCCTTGATCGTTG<br>GGAACCGGAGCTGAATGAAGCCATACCAAACGACGAGCGTGACACCACGATGCCT<br>GTAGCAATGGCAACAACGTTGCGCAAACTATTAACTGGCGAACTACTTACTCTAGC<br>TTCCCGGCAACAATTAATAGACTGGATGGAGGCGGATAAAGTTGCAGGACCACTTC<br>TGCGCTCGGCCCTTCCGGCTGGCTGGTTTATTGCTGATAAATCTGGAGCCGGTGA<br>GCGTGGGTCTCGCGGTATCATTGCAGCACTGGGGCCAGATGGTAAGCCCTCCCGT<br>ATCGTAGTTATCTACACGACGGGGAGTCAGGCAACTATGGATGAACGAAATAGACA<br>GATCGCTGAGATAGGTGCCTCACTGATTAAGCATTGGTAACTGTCAGACCAAGTTT<br>ACTCATATATACTTTAGATTGATTTAAAACTTCATTTTTAATTTAAAAGGATCTAGGTG<br>AAGATCCTTTTTGATAATCTCATGACCAAAATCCCTTAACGTGAGTTTTCGTTCCACT<br>GAGCGTCAGACCCCGTAGAAAAGATCAAAGGATCTTCTTGAGATCCTTTTTTTCTG<br>CGCGTAATCTGCTGCTTGCAAACAAAAAAACCACCGCTACCAGCGGTGGTTTGTTT

	GCCGGATCAAGAGCTACCAACTCTTTTTCCGAAGGTAACTGGCTTCAGCAGAGCG<br>CAGATACCAAATACTGTTCTTCTAGTGTAGCCGTAGTTAGGCCACCACTTCAAGAA<br>CTCTGTAGCACCGCCTACATACCTCGCTCTGCTAATCCTGTTACCAGTGGCTGCTG<br>CCAGTGGCGATAAGTCGTGTCTTACCGGGTTGGACTCAAGACGATAGTTACCGGA<br>TAAGGCGCAGCGGTCGGGCTGAACGGGGGGTTCGTGCACACAGCCCAGCTTGGA<br>GCGAACGACCTACACCGAACT
pIV008	GAGATACCTACAGCGTGAGCTATGAGAAAGCGCCACGCTTCCCGAAGGGAGAAAG<br>GCGGACAGGTATCCGGTAAGCGGCAGGGTCGGAACAGGAGAGCGCACGAGGGA<br>GCTTCCAGGGGGAAACGCCTGGTATCTTTATAGTCCTGTCGGGTTTCGCCACCTCT<br>GACTTGAGCGTCGATTTTTGTGATGCTCGTCAGGGGGGCGGAGCCTATGGAAAAA<br>CGCCAGCAACGCGGCCTTTTTACGGTTCCTGGCCTTTTGCTGGCCTTTTGCTCACA<br>TGTTCTTTCCTGCGTTATCCCCTGATTCTGTGGATAACCGTATTACCGCCTTTGAGT<br>GAGCTGATACCGCTCGCCGCAGCCGAACGACCGAGCGCAGCGAGTCAGTGAGCG<br>AGGAAGCGGAAGAGCGCCCAATACGCAAACCGCCTCTCCCCGCGCGTTGGCCGA<br>TTCATTAATGCAGCTGGCACGACAGGTTTCCCGACTGGAAAGCGGGCAGTGAGCG<br>CAACGCAATTAATGTGAGTTAGCTCACTCATTAGGCACCCCAGGCTTTACACTTTAT<br>GCTTCCGGCTCGTATGTTGTGTGGAATTGTGAGCGGATAACAATTTCACACAGGAA<br>ACAGCTATGACCATGATTACGCCAAGCTTGCATGCCTGCAGGTCGACTCTAGAaaag<br>attaaaaaaagtcaaagctacactggcatactttgaactgtttttcaaagctgtctccacgtggaaagtttatttcatcaaaac<br>tattatcaaaaattgtggttttgttaatacaaaagctgatatatatataatatggcatttagtgattttgctgccatatgctcaaaga<br>ccccgttgccattatgttcggtaataaagtctaaaacccatctaatactttcgaactcaacaattatacatgattttgatcctttaa<br>atttgaatgtcggtgtactgccacgctgttatgctcggtcgattgatcttgccaatacagtcatctttgatgtcgggaacgcattc<br>ataaatattggtgctctaggtgtcattttaatcatactttataacataagacagaagtatactgctattggcaggtctgaatatctc<br>tactttttccaactaacattgctattgataatatttaccttggtggtagactgtggtgtatctCgacatggaggcccagaataccc<br>tccttgacagtcttgacgtgcgcagctcaggggcatgatgtgactgtcgcccgtacatttagcccatacatccccatgtataat<br>catttgcatccatacattttgatggccgcacggcgcgaagcaaaaattacggctcctcgctgcagacctgcgagcaggga<br>aacgctcccctcacagacgcgttgaattgtccccacgccgcgcccctgtagagaaatataaaaggttaggatttgccactg<br>aggttcttctttcatatacttccttttaaaatcttgctaggatacagttctcacatcacatccgaacataaacaaccatgggtacc<br>actcttgacgacacggcttaccggtaccgcaccagtgtcccgggggacgccgaggccatcgaggcactggatgggtcct<br>tcaccaccgacaccgttttccgcgtcaccAccaccggggacggcttcaccctgcgggaggtgccggtggacccgcccct<br>gaccaaggtgttccccgacgacgaatcggacgacgaatcggacgacggggaggacggcgacccggactcccggac<br>gttcgtcgcgtacggggacgacggcgacctggcgggcttcgtggtcgtctcgtactccggctggaaccgccggctgaccg<br>tcgaggacatcgaggtcgccccggagcaccgggggcacggggtcgggcgcgcgttgatggggctcgcgacggagttc<br>gcccgcgagcggggcgccgggcacctctggctggaggtcaccaacgtcaacgcaccggcgatccacgcgtaccggc<br>ggatggggttcaccctctgcggcctggacaccgccctgtacgacggcaccgcctcggacggcgagcaggcgctctacat<br>gagcatgccctgcccctaatcagtactgacaataaaaagattcttgttttcaagaacttgtcatttgtatagtttttttatattgtagt<br>tgttctattttaatcaaatgttagcgtgatttatattttttttcgcctcgacatcatctgcccagatgcgaagttaagtgcgcagaaa<br>gtaatatcatgcgtcaatcgtatgtgaatgctggtcgctatactgCCATGGGCTAGCCTCGAGAGATCTGC<br>GGCCGCcgggtgcatgttgctgggccttattgataatcgggtttttaggtttcaatttatgggaagatgggactacaaagtc<br>catgctgttggtccgtggaacgtccatgctaggattcatagccaatttttttagcctctattttaaccttcaaagcatggatcaccg<br>accataaagtagcaacaatgaacgcttcagggatgattgtcgtcgtttacataataaacgccattttcttattcgttttcgttattt<br>gtcaattactggtatccctattggtagttcgaaacttatgggtcacaggagctatctttttggggctatttttctttgtagcaggcca<br>ggtattggtttatgccttctctacacaaatttgtgaagggttcaagcactacttagatggcctctttttttggaagcatctgtaatgtg<br>ttcacattaatgatggtttacaagacttgggatatgactaccgacgacgacttggaatttggtgtaagtgttagcGGATCC<br>CCGGGTACCGAGCTCGAATTCACTGGCCGTCGTTTTACAACGTCGTGACTGGGAA<br>AACCCTGGCGTTACCCAACTTAATCGCCTTGCAGCACATCCCCCTTTCGCCAGCTG<br>GCGTAATAGCGAAGAGGCCCGCACCGATCGCCCTTCCCAACAGTTGCGCAGCCTG<br>AATGGCGAATGGCGCCTGATGCGGTATTTTCTCCTTACGCATCTGTGCGGTATTTC<br>ACACCGCATATGGTGCACTCTCAGTACAATCTGCTCTGATGCCGCATAGTTAAGCC<br>AGCCCCGACACCCGCCAACACCCGCTGACGCGCCCTGACGGGCTTGTCTGCTCC<br>CGGCATCCGCTTACAGACAAGCTGTGACCGTCTCCGGGAGCTGCATGTGTCAGAG<br>GTTTTCACCGTCATCACCGAAACGCGCGAGACGAAAGGGCCTCGTGATACGCCTA<br>TTTTTATAGGTTAATGTCATGATAATAATGGTTTCTTAGACGTCAGGTGGCACTTTTC<br>GGGGAAATGTGCGCGGAACCCCTATTTGTTTATTTTTCTAAATACATTCAAATATGT<br>ATCCGCTCATGAGACAATAACCCTGATAAATGCTTCAATAATATTGAAAAAGGAAGA<br>GTATGAGTATTCAACATTTCCGTGTCGCCCTTATTCCCTTTTTTGCGGCATTTTGCC<br>TTCCTGTTTTTGCTCACCCAGAAACGCTGGTGAAAGTAAAAGATGCTGAAGATCAG<br>TTGGGTGCACGAGTGGGTTACATCGAACTGGATCTCAACAGCGGTAAGATCCTTGA<br>GAGTTTTCGCCCCGAAGAACGTTTTCCAATGATGAGCACTTTTAAAGTTCTGCTATG<br>TGGCGCGGTATTATCCCGTATTGACGCCGGGCAAGAGCAACTCGGTCGCCGCATA<br>CACTATTCTCAGAATGACTTGGTTGAGTACTCACCAGTCACAGAAAAGCATCTTACG

	GATGGCATGACAGTAAGAGAATTATGCAGTGCTGCCATAACCATGAGTGATAACAC<br>TGCGGCCAACTTACTTCTGACAACGATCGGAGGACCGAAGGAGCTAACCGCTTTTT<br>TGCACAACATGGGGGATCATGTAACTCGCCTTGATCGTTGGGAACCGGAGCTGAA<br>TGAAGCCATACCAAACGACGAGCGTGACACCACGATGCCTGTAGCAATGGCAACA<br>ACGTTGCGCAAACTATTAACTGGCGAACTACTTACTCTAGCTTCCCGGCAACAATTA<br>ATAGACTGGATGGAGGCGGATAAAGTTGCAGGACCACTTCTGCGCTCGGCCCTTC<br>CGGCTGGCTGGTTTATTGCTGATAAATCTGGAGCCGGTGAGCGTGGGTCTCGCGG<br>TATCATTGCAGCACTGGGGCCAGATGGTAAGCCCTCCCGTATCGTAGTTATCTACA<br>CGACGGGGAGTCAGGCAACTATGGATGAACGAAATAGACAGATCGCTGAGATAGG<br>TGCCTCACTGATTAAGCATTGGTAACTGTCAGACCAAGTTTACTCATATATACTTTA<br>GATTGATTTAAAACTTCATTTTTAATTTAAAAGGATCTAGGTGAAGATCCTTTTTGAT<br>AATCTCATGACCAAAATCCCTTAACGTGAGTTTTCGTTCCACTGAGCGTCAGACCC<br>CGTAGAAAAGATCAAAGGATCTTCTTGAGATCCTTTTTTTCTGCGCGTAATCTGCTG<br>CTTGCAAACAAAAAAACCACCGCTACCAGCGGTGGTTTGTTTGCCGGATCAAGAGC<br>TACCAACTCTTTTTCCGAAGGTAACTGGCTTCAGCAGAGCGCAGATACCAAATACT<br>GTTCTTCTAGTGTAGCCGTAGTTAGGCCACCACTTCAAGAACTCTGTAGCACCGCC<br>TACATACCTCGCTCTGCTAATCCTGTTACCAGTGGCTGCTGCCAGTGGCGATAAGT<br>CGTGTCTTACCGGGTTGGACTCAAGACGATAGTTACCGGATAAGGCGCAGCGGTC<br>GGGCTGAACGGGGGGTTCGTGCACACAGCCCAGCTTGGAGCGAACGACCTACAC<br>CGAACT
pIV009	GAGATACCTACAGCGTGAGCTATGAGAAAGCGCCACGCTTCCCGAAGGGAGAAAG<br>GCGGACAGGTATCCGGTAAGCGGCAGGGTCGGAACAGGAGAGCGCACGAGGGA<br>GCTTCCAGGGGGAAACGCCTGGTATCTTTATAGTCCTGTCGGGTTTCGCCACCTCT<br>GACTTGAGCGTCGATTTTTGTGATGCTCGTCAGGGGGGCGGAGCCTATGGAAAAA<br>CGCCAGCAACGCGGCCTTTTTACGGTTCCTGGCCTTTTGCTGGCCTTTTGCTCACA<br>TGTTCTTTCCTGCGTTATCCCCTGATTCTGTGGATAACCGTATTACCGCCTTTGAGT<br>GAGCTGATACCGCTCGCCGCAGCCGAACGACCGAGCGCAGCGAGTCAGTGAGCG<br>AGGAAGCGGAAGAGCGCCCAATACGCAAACCGCCTCTCCCCGCGCGTTGGCCGA<br>TTCATTAATGCAGCTGGCACGACAGGTTTCCCGACTGGAAAGCGGGCAGTGAGCG<br>CAACGCAATTAATGTGAGTTAGCTCACTCATTAGGCACCCCAGGCTTTACACTTTAT<br>GCTTCCGGCTCGTATGTTGTGTGGAATTGTGAGCGGATAACAATTTCACACAGGAA<br>ACAGCTATGACCATGATTACGCCAAGCTTGCATGCCTGCAGGTCGACTCTAGAgcgc<br>taattacatttgatgaaaggttatcttttaaagccgctaacgatcaagatatggtggattggataataaatttcaagagtggaat<br>tttgataagaaaaaaattaaaggccgaaaatatataagcaatgccaatgatgaatatgtattgactttttttttctttgtactatta<br>aataatacgtgtaattgttttccttacgtctagttaaaaaactatttatttatttgtattttaatgataataagcactttttcctcgctga<br>aaattaagggaaaggaccaaatacaaaagtgattagaaggataacgagtagagaaaaagggaagtaaaagttaaaa<br>actagaatcctagtatgacatgcattgcccaatttgccaccatagagcgcatgtagtgtactgtgcacattgtatcaatacga<br>gcccaagtctgctactgaagctaaaactagatttaattttattaaaggatgagaataaagaacttaacgggaaCAACTG<br>TGGGAATACTCAGGTATCGTAAGATGCAAGAGTTCGAATCTCTTAGCAACCATTATT<br>TTTTTCCTCAACATAACGAGAACACACAGGGGCGCTATCGCACAGAATCAAATTCG<br>ATGACTGGAAATTTTTTGTTAATTTCAGAGGTCGCCTGACGCATATACCTTTTTCAA<br>CTGAAAAATTGGGAGAAAAAGGAAAGGTGAGAGCGCCGGAACCGGCTTTTCATAT<br>AGAATAGAGAAGCGTTCATGACTAAATGCTTGCATCACAATACTTGAAGTTGACAAT<br>ATTATTTAAGGACCTATTGTTTTTTCCAATAGGTGGTTAGCAATCGTCTTACTTTCTA<br>ACTTTTCTTACCTTTTACATTTCAGCAATATATATATATATATATTTCAAGGATATACCAT<br>TCTAATGTCTGCCCCTAAGAAGATCGTCGTTTTGCCAGGTGACCACGTTGGTCAAG<br>AAATCACAGCCGAAGCCATTAAGGTTCTTAAAGCTATTTCTGATGTTCGTTCCAATG<br>TCAAGTTCGATTTCGAAAATCATTTAATTGGTGGTGCTGCTATCGATGCTGCAGGTA<br>TTCCACTTCCAGATGAGGCGCTGGAAGCCTCCAAGAAGGCTGATGCCGTTTTGTTA<br>GGTGCTGTGGGTGGTCCTAAATGGGGTACCGGTAGTGTTAGACCTGAACAAGGTT<br>TACTAAAAATCCGTAAAGAACTTCAATTGTACGCCAACTTAAGACCATGTAACTTTG<br>CATCCGACTCTCTTTTAGACTTATCTCCAATCAAGCCACAATTTGCTAAAGGTACTG<br>ACTTCGTTGTTGTCAGAGAATTAGTGGGAGGTATTTACTTTGGTAAGAGAAAGGAA<br>GACGATGGTGATGGTGTCGCTTGGGATAGTGAACAATACACCGTTCCAGAAGTGC<br>AAAGAATCACAAGAATGGCCGCTTTCATGGCCCTACAACATGAGCCACCATTGCCT<br>ATTTGGTCCTTGGATAAAGCTAATGTTTTGGCCTCTTCAAGATTATGGAGAAAAACT<br>GTGGAGGAAACCATCAAGAACGAATTCCCTACATTGAAGGTTCAACATCAATTGATT<br>GATTCTGCCGCCATGATCCTAGTTAAGAACCCAACCCACCTAAATGGTATTATAATC<br>ACCAGCAACATGTTTGGTGATATCATCTCCGATGAAGCCTCCGTTATCCCAGGTTC<br>CTTGGGTTTGTTGCCATCTGCGTCCTTGGCCTCTTTGCCAGACAAGAACACCGCAT<br>TTGGTTTGTACGAACCATGCCACGGTTCTGCTCCAGATTTGCCAAAGAATAAGGTC

	AACCCTATCGCCACTATCTTGTCTGCTGCAATGATGTTGAAATTGTCATTGAACTTG<br>CCTGAAGAAGGTAAGGCCATTGAAGATGCAGTTAAAAAGGTTTTGGATGCAGGTAT<br>CAGAACTGGTGATTTAGGTGGTTCCAACAGTACCACCGAAGTCGGTGATGCTGTC<br>GCCGAAGAAGTTAAGAAAATCCTTGCTTAAAAAGATTCTCTTTTTTTATGATATTTGT<br>ACATAAACTTTATAAATGAAATTCATAATAGAAACGACACGAAATTACAAAATGGAAT<br>ATGTTCATAGGGTAGACGAAACTATATACGCAATCTACATACATTTATCAAGAAGGA<br>GAAAAAGGAGGATGTAAAGGAATACAGGTAAGCAAATTGATACTAATGGCTCAACG<br>TGATAAGGAAAAAGAATTGCACTTTAACATTAATATTGACAAGGAGGAGGGCACCA<br>CACAAAAAGTTAGGTGTAACAGAAAATCATGAAACTATGATTCCTAATTTATATATTG<br>GAGGATTTTCTCTAAAAAAAAAAAAAATACAACAAATAAAAAACACTCAATGACCTGA<br>CCATTTGATGGAGTTTAAGTCAATACCTTCTTGAACCATTTCCCATAATGGTGAAAG<br>TTCCCTCAAGAATTTTACTCTGTCAGAAACGGCCTTAACGACGTAGTCGATATGGT<br>GCACTCTCAGTACAATCTGCTCTGATGCCGCATAGTTAAGCCAGCCCCGACACCC<br>GCCAACACCCGCTGACGCGCCCTGACGGGCTTGTCTGCTCCCGGCATCCCCATG<br>GGCTAGCCTCGAGAGATCCATAGCTTCAAAATGTTTCTACTCCTTTTTTACTCTTCC<br>AGATTTTCTCGGACTCCGCGCATCGCCGTACCACTTCAAAACACCCAAGCACAGCA<br>TACTAAATTTCCCCTCTTTCTTCCTCTAGGGTGTCGTTAATTACCCGTACTAAAGGT<br>TTGGAAAAGAAAAAAGAGACCGCCTCGTTTCTTTTTCTTCGTCGAAAAAGGCAATAA<br>AAATTTTTATCACGTTTCTTTTTCTTGAAAATTTTTTTTTTGATTTTTTTCTCTTTCGAT<br>GACCTCCCATTGATATTTAAGTTAATAAACGGTCTTCAATTTCTCAAGTTTCAGTTTC<br>ATTTTTCTTGTTCTATTACAACTTTTTTTACTTCTTGCTCATTAGAAAGAAAGCATAGC<br>AATCTAATCTAAGCCAAGCTAGATCTACCGGTCAAAATGTCTAAAGGTGAAGAATTA<br>TTCACTGGTGTTGTCCCAATTTTGGTTGAATTAGATGGTGATGTTAATGGTCACAAA<br>TTTTCTGTCTCCGGTGAAGGTGAAGGTGATGCTACTTACGGTAAATTGACCTTAAAA<br>TTTATTTGTACTACTGGTAAATTGCCAGTTCCATGGCCAACCTTAGTCACTACTTTC<br>GGTTATGGTGTTCAATGTTTTGCGAGATACCCAGATCATATGAAACAACATGACTTT<br>TTCAAGTCTGCCATGCCAGAAGGTTATGTTCAAGAAAGAACTATTTTTTTCAAAGAT<br>GACGGTAACTACAAGACCAGAGCTGAAGTCAAGTTTGAAGGTGATACCTTAGTTAA<br>TAGAATCGAATTAAAAGGTATTGATTTTAAAGAAGATGGTAACATTTTAGGTCACAA<br>ATTGGAATACAACTATAACTCTCACAATGTTTACATCATGGCTGACAAACAAAAGAA<br>TGGTATCAAAGTTAACTTCAAAATTAGACACAACATTGAAGATGGTTCTGTTCAATTA<br>GCTGACCATTATCAACAAAATACTCCAATTGGTGATGGTCCAGTCTTGTTACCAGAC<br>AACCATTACTTATCCACTCAATCTGCCTTATCCAAAGATCCAAACGAAAAGAGAGAC<br>CACATGGTCTTGTTAGAATTTGTTACTGCTGCTGGTATTACCCATGGTATGGATGAA<br>TTGTACAAATAACTCGAGTAAGCTTGGTACCGCGGCTAGCTAAGATCCGCTCTAAC<br>CGAAAAGGAAGGAGTTAGACAACCTGAAGTCTAGGTCCCTATTTATTTTTTTATAGT<br>TATGTTAGTATTAAGAACGTTATTTATATTTCAAATTTTTCTTTTTTTTCTGTACAGAC<br>GCGTGTACGCATGTAACATTATACTGAAAACCTTGCTTGAGAAGGTTTTGGGACGC<br>TCGAAGGCGGCCGCatggagaaaaagaaagatcccctgatgaatagtctcatgaagttagatgttttacgaatg<br>aaaaagaataataacctgattaggcacaggatagaacagctaaatgagcggatttatagtaaaaggaatcacatcagc<br>gaattgaaagtagaaattgacaattataaatgttataaggtgggtactggtacggacaaattaagagagcaagtggaaatt<br>agtgatgcaaaaaataagctggcacaagtatcgaagatttgtgaatcggctagagattacaagctcaatctactaaataat<br>tggtttgtgatccagaagctgcaagataatttccaaatacctttcgctatagcttttcagccactaatatctttgaaaaacttccgt<br>attttacctcttgctataacaaacgattccatcaacatcatgtggaagtatattagcttcttctcagacattcttatgattaaacttc<br>cctacacaaataaaatctgtgaacaGGATCCCCGGGTACCGAGCTCGAATTCACTGGCCGTC<br>GTTTTACAACGTCGTGACTGGGAAAACCCTGGCGTTACCCAACTTAATCGCCTTGC<br>AGCACATCCCCCTTTCGCCAGCTGGCGTAATAGCGAAGAGGCCCGCACCGATCGC<br>CCTTCCCAACAGTTGCGCAGCCTGAATGGCGAATGGCGCCTGATGCGGTATTTTCT<br>CCTTACGCATCTGTGCGGTATTTCACACCGCATATGGTGCACTCTCAGTACAATCT<br>GCTCTGATGCCGCATAGTTAAGCCAGCCCCGACACCCGCCAACACCCGCTGACGC<br>GCCCTGACGGGCTTGTCTGCTCCCGGCATCCGCTTACAGACAAGCTGTGACCGTC<br>TCCGGGAGCTGCATGTGTCAGAGGTTTTCACCGTCATCACCGAAACGCGCGAGAC<br>GAAAGGGCCTCGTGATACGCCTATTTTTATAGGTTAATGTCATGATAATAATGGTTT<br>CTTAGACGTCAGGTGGCACTTTTCGGGGAAATGTGCGCGGAACCCCTATTTGTTTA<br>TTTTTCTAAATACATTCAAATATGTATCCGCTCATGAGACAATAACCCTGATAAATGC<br>TTCAATAATATTGAAAAAGGAAGAGTATGAGTATTCAACATTTCCGTGTCGCCCTTA<br>TTCCCTTTTTTGCGGCATTTTGCCTTCCTGTTTTTGCTCACCCAGAAACGCTGGTGA<br>AAGTAAAAGATGCTGAAGATCAGTTGGGTGCACGAGTGGGTTACATCGAACTGGAT<br>CTCAACAGCGGTAAGATCCTTGAGAGTTTTCGCCCCGAAGAACGTTTTCCAATGAT<br>GAGCACTTTTAAAGTTCTGCTATGTGGCGCGGTATTATCCCGTATTGACGCCGGGC<br>AAGAGCAACTCGGTCGCCGCATACACTATTCTCAGAATGACTTGGTTGAGTACTCA

	CCAGTCACAGAAAAGCATCTTACGGATGGCATGACAGTAAGAGAATTATGCAGTGC TGCCATAACCATGAGTGATAACACTGCGGCCAACTTACTTCTGACAACGATCGGAG GACCGAAGGAGCTAACCGCTTTTTTGCACAACATGGGGGATCATGTAACTCGCCTT GATCGTTGGGAACCGGAGCTGAATGAAGCCATACCAAACGACGAGCGTGACACCA CGATGCCTGTAGCAATGGCAACAACGTTGCGCAAACTATTAACTGGCGAACTACTT ACTCTAGCTTCCCGGCAACAATTAATAGACTGGATGGAGGCGGATAAAGTTGCAGG ACCACTTCTGCGCTCGGCCCTTCCGGCTGGCTGGTTTATTGCTGATAAATCTGGAG CCGGTGAGCGTGGGTCTCGCGGTATCATTGCAGCACTGGGGCCAGATGGTAAGC CCTCCCGTATCGTAGTTATCTACACGACGGGGAGTCAGGCAACTATGGATGAACG AAATAGACAGATCGCTGAGATAGGTGCCTCACTGATTAAGCATTGGTAACTGTCAG ACCAAGTTTACTCATATATACTTTAGATTGATTTAAAACTTCATTTTTAATTTAAAAGG ATCTAGGTGAAGATCCTTTTTGATAATCTCATGACCAAAATCCCTTAACGTGAGTTT TCGTTCCACTGAGCGTCAGACCCCGTAGAAAAGATCAAAGGATCTTCTTGAGATCC TTTTTTTCTGCGCGTAATCTGCTGCTTGCAAACAAAAAAACCACCGCTACCAGCGG TGGTTTGTTTGCCGGATCAAGAGCTACCAACTCTTTTTCCGAAGGTAACTGGCTTC AGCAGAGCGCAGATACCAAATACTGTTCTTCTAGTGTAGCCGTAGTTAGGCCACCA CTTCAAGAACTCTGTAGCACCGCCTACATACCTCGCTCTGCTAATCCTGTTACCAG TGGCTGCTGCCAGTGGCGATAAGTCGTGTCTTACCGGGTTGGACTCAAGACGATA GTTACCGGATAAGGCGCAGCGGTCGGGCTGAACGGGGGGTTCGTGCACACAGCC CAGCTTGGAGCGAACGACCTACACCGAACT
pIV010	GAGATACCTACAGCGTGAGCTATGAGAAAGCGCCACGCTTCCCGAAGGGAGAAAG GCGGACAGGTATCCGGTAAGCGGCAGGGTCGGAACAGGAGAGCGCACGAGGGA GCTTCCAGGGGGAAACGCCTGGTATCTTTATAGTCCTGTCGGGTTTCGCCACCTCT GACTTGAGCGTCGATTTTTGTGATGCTCGTCAGGGGGGCGGAGCCTATGGAAAAA CGCCAGCAACGCGGCCTTTTTACGGTTCCTGGCCTTTTGCTGGCCTTTTGCTCACA TGTTCTTTCCTGCGTTATCCCCTGATTCTGTGGATAACCGTATTACCGCCTTTGAGT GAGCTGATACCGCTCGCCGCAGCCGAACGACCGAGCGCAGCGAGTCAGTGAGCG AGGAAGCGGAAGAGCGCCCAATACGCAAACCGCCTCTCCCCGCGCGTTGGCCGA TTCATTAATGCAGCTGGCACGACAGGTTTCCCGACTGGAAAGCGGGCAGTGAGCG CAACGCAATTAATGTGAGTTAGCTCACTCATTAGGCACCCCAGGCTTTACACTTTAT GCTTCCGGCTCGTATGTTGTGTGGAATTGTGAGCGGATAACAATTTCACACAGGAA ACAGCTATGACCATGATTACGCCAAGCTTGCATGCCTGCAGGTCGACTCTAGAcaac gcaaaataaataattaatatgtaaaatatctgtttacttgaaatttacccttaacagaggtactaacgggaggaaggactgg ccttgctccggtagacctcgatgcctgaaccttctccctcatctcgaaacggtacgacctctgagaaaacgatatcaatcaa tgcgcgtcttccagatgaaattcttcggaatgcccataatacgaaaaactaacgatggaaaaaatactataaatgtatgggt gtaattttaaccgaatgtatggatgttacagggagctttatatatgtagaaaattccagacgttccaaacaaataagtgaaaa tcaacacgattgggaaagtacatgccagagttcgatgtttaggttaatgataaaaataatttttctctaatcaccctccattttcg ataaaatatcagcgtagtctgaaaatatatacattaagcaaaaagaatgtcaaacaagtttcactgtgacgtttgttcagcC GCTTAACTATGCGGCATCAGAGCAGATTGTACTGAGAGTGCACCATAGATCAACGA CATTACTATATATATAATATAGGAAGCATTTAATAGAACAGCATCGTAATATATGTGT ACTTTGCAGTTATGACGCCAGATGGCAGTAGTGGAAGATATTCTTTATTGAAAAATA GCTTGTCACCTTACGTACAATCTTGATCCGGAGCTTTTCTTTTTTTGCCGATTAAGA ATTAATTCGGTCGAAAAAAGAAAAGGAGAGGGCCAAGAGGGAGGGCATTGGTGAC TATTGAGCACGTGAGTATACGTGATTAAGCACACAAAGGCAGCTTGGAGTATGTCT GTTATTAATTTCACAGGTAGTTCTGGTCCATTGGTGAAAGTTTGCGGCTTGCAGAG CACAGAGGCCGCAGAATGTGCTCTAGATTCCGATGCTGACTTGCTGGGTATTATAT GTGTGCCCAATAGAAAGAGAACAATTGACCCGGTTATTGCAAGGAAAATTTCAAGT CTTGTAAAAGCATATAAAAATAGTTCAGGCACTCCGAAATACTTGGTTGGCGTGTTT CGTAATCAACCTAAGGAGGATGTTTTGGCTCTGGTCAATGATTACGGCATTGATAT CGTCCAACTGCATGGAGATGAGTCGTGGCAAGAATACCAAGAGTTCCTCGGTTTG CCAGTTATTAAAAGACTCGTATTTCCAAAAGACTGCAACATACTACTCAGTGCAGCT TCACAGAAACCTCATTCGTTTATTCCCTTGTTTGATTCAGAAGCAGGTGGGACAGG TGAACTTTTGGATTGGAACTCGATTTCTGACTGGGTTGGAAGGCAAGAGAGCCCCG AAAGCTTACATTTTATGTTAGCTGGTGGACTGACGCCAGAAAATGTTGGTGATGCG CTTAGATTAAATGGCGTTATTGGTGTTGATGTAAGCGGAGGTGTGGAGACAAATGG TGTAAAAGACTCTAACAAAATAGCAAATTTCGTCAAAAATGCTAAGAAATAGGTTATT ACTGAGTAGTATTTATTTAAGTATTGTTTGTGCACTTGCCGATCTATGCGGTGTGAA ATACCGCACAGATGCGTAAGGAGAAAATACCGCATCAGGAAATTGTAAACGTTAAT CCATGGGCTAGCCTCGAGAGATCTCATAGCTTCAAAATGTTTCTACTCCTTTTTTAC TCTTCCAGATTTTCTCGGACTCCGCGCATCGCCGTACCACTTCAAAACACCCAAGC ACAGCATACTAAATTTCCCCTCTTTCTTCCTCTAGGGTGTCGTTAATTACCCGTACT

	AAAGGTTTGGAAAAGAAAAAAGAGACCGCCTCGTTTCTTTTTCTTCGTCGAAAAAG<br>GCAATAAAAATTTTTATCACGTTTCTTTTTCTTGAAAATTTTTTTTTTGATTTTTTTCTC<br>TTTCGATGACCTCCCATTGATATTTAAGTTAATAAACGGTCTTCAATTTCTCAAGTTT<br>CAGTTTCATTTTTCTTGTTCTATTACAACTTTTTTTACTTCTTGCTCATTAGAAAGAAA<br>GCATAGCAATCTAATCTAAGCCAAGCTAGATCTACCGGTCAAAATGTCTAAAGGTG<br>AAGAATTATTCACTGGTGTTGTCCCAATTTTGGTTGAATTAGATGGTGATGTTAATG<br>GTCACAAATTTTCTGTCTCCGGTGAAGGTGAAGGTGATGCTACTTACGGTAAATTG<br>ACCTTAAAATTTATTTGTACTACTGGTAAATTGCCAGTTCCATGGCCAACCTTAGTC<br>ACTACTTTCGGTTATGGTGTTCAATGTTTTGCGAGATACCCAGATCATATGAAACAA<br>CATGACTTTTTCAAGTCTGCCATGCCAGAAGGTTATGTTCAAGAAAGAACTATTTTT<br>TTCAAAGATGACGGTAACTACAAGACCAGAGCTGAAGTCAAGTTTGAAGGTGATAC<br>CTTAGTTAATAGAATCGAATTAAAAGGTATTGATTTTAAAGAAGATGGTAACATTTTA<br>GGTCACAAATTGGAATACAACTATAACTCTCACAATGTTTACATCATGGCTGACAAA<br>CAAAAGAATGGTATCAAAGTTAACTTCAAAATTAGACACAACATTGAAGATGGTTCT<br>GTTCAATTAGCTGACCATTATCAACAAAATACTCCAATTGGTGATGGTCCAGTCTTG<br>TTACCAGACAACCATTACTTATCCACTCAATCTGCCTTATCCAAAGATCCAAACGAA<br>AAGAGAGACCACATGGTCTTGTTAGAATTTGTTACTGCTGCTGGTATTACCCATGGT<br>ATGGATGAATTGTACAAATAACTCGAGTAAGCTTGGTACCGCGGCTAGCTAAGATC<br>CGCTCTAACCGAAAAGGAAGGAGTTAGACAACCTGAAGTCTAGGTCCCTATTTATT<br>TTTTTATAGTTATGTTAGTATTAAGAACGTTATTTATATTTCAAATTTTTCTTTTTTTTC<br>TGTACAGACGCGTGTACGCATGTAACATTATACTGAAAACCTTGCTTGAGAAGGTTT<br>TGGGACGCTCGAAGGCGGCCGCtacaggaaagcatcgcccttaccatgattataggataatagagacca<br>attcataccccattctctgtcctgattggggtgccgatgaagaattgcagttgattaaaggcgcacagactttagggctcggta<br>attggcaggatattgctgaccatataggcagcagaggcaaagaagaagttaaggaacattacctaaaatattatctggaa<br>agcaaatactatccaatacctgatattacccaaaatatacatgtcccacaagatgaatttttggaacagcgaaggcataga<br>atcgagtccttccgggagaggccgctagagcctccaagaaagcccatggcatcggttcctagctgccatgaagtacagg<br>ggtttatgccgggcagattagaatttgaaacagaattcgaaaatgaagctgaaggtcccgtaaaggatatggtttttgaacc<br>tgatgaccagcccttagacattgaattgaagtttgccatcttggacaGGATCCCCGGGTACCGAGCTCGAA<br>TTCACTGGCCGTCGTTTTACAACGTCGTGACTGGGAAAACCCTGGCGTTACCCAAC<br>TTAATCGCCTTGCAGCACATCCCCCTTTCGCCAGCTGGCGTAATAGCGAAGAGGC<br>CCGCACCGATCGCCCTTCCCAACAGTTGCGCAGCCTGAATGGCGAATGGCGCCTG<br>ATGCGGTATTTTCTCCTTACGCATCTGTGCGGTATTTCACACCGCATATGGTGCACT<br>CTCAGTACAATCTGCTCTGATGCCGCATAGTTAAGCCAGCCCCGACACCCGCCAA<br>CACCCGCTGACGCGCCCTGACGGGCTTGTCTGCTCCCGGCATCCGCTTACAGACA<br>AGCTGTGACCGTCTCCGGGAGCTGCATGTGTCAGAGGTTTTCACCGTCATCACCG<br>AAACGCGCGAGACGAAAGGGCCTCGTGATACGCCTATTTTTATAGGTTAATGTCAT<br>GATAATAATGGTTTCTTAGACGTCAGGTGGCACTTTTCGGGGAAATGTGCGCGGAA<br>CCCCTATTTGTTTATTTTTCTAAATACATTCAAATATGTATCCGCTCATGAGACAATA<br>ACCCTGATAAATGCTTCAATAATATTGAAAAAGGAAGAGTATGAGTATTCAACATTT<br>CCGTGTCGCCCTTATTCCCTTTTTTGCGGCATTTTGCCTTCCTGTTTTTGCTCACCC<br>AGAAACGCTGGTGAAAGTAAAAGATGCTGAAGATCAGTTGGGTGCACGAGTGGGT<br>TACATCGAACTGGATCTCAACAGCGGTAAGATCCTTGAGAGTTTTCGCCCCGAAGA<br>ACGTTTTCCAATGATGAGCACTTTTAAAGTTCTGCTATGTGGCGCGGTATTATCCCG<br>TATTGACGCCGGGCAAGAGCAACTCGGTCGCCGCATACACTATTCTCAGAATGACT<br>TGGTTGAGTACTCACCAGTCACAGAAAAGCATCTTACGGATGGCATGACAGTAAGA<br>GAATTATGCAGTGCTGCCATAACCATGAGTGATAACACTGCGGCCAACTTACTTCT<br>GACAACGATCGGAGGACCGAAGGAGCTAACCGCTTTTTTGCACAACATGGGGGAT<br>CATGTAACTCGCCTTGATCGTTGGGAACCGGAGCTGAATGAAGCCATACCAAACGA<br>CGAGCGTGACACCACGATGCCTGTAGCAATGGCAACAACGTTGCGCAAACTATTAA<br>CTGGCGAACTACTTACTCTAGCTTCCCGGCAACAATTAATAGACTGGATGGAGGCG<br>GATAAAGTTGCAGGACCACTTCTGCGCTCGGCCCTTCCGGCTGGCTGGTTTATTG<br>CTGATAAATCTGGAGCCGGTGAGCGTGGGTCTCGCGGTATCATTGCAGCACTGGG<br>GCCAGATGGTAAGCCCTCCCGTATCGTAGTTATCTACACGACGGGGAGTCAGGCA<br>ACTATGGATGAACGAAATAGACAGATCGCTGAGATAGGTGCCTCACTGATTAAGCA<br>TTGGTAACTGTCAGACCAAGTTTACTCATATATACTTTAGATTGATTTAAAACTTCAT<br>TTTTAATTTAAAAGGATCTAGGTGAAGATCCTTTTTGATAATCTCATGACCAAAATCC<br>CTTAACGTGAGTTTTCGTTCCACTGAGCGTCAGACCCCGTAGAAAAGATCAAAGGA<br>TCTTCTTGAGATCCTTTTTTTCTGCGCGTAATCTGCTGCTTGCAAACAAAAAAACCA<br>CCGCTACCAGCGGTGGTTTGTTTGCCGGATCAAGAGCTACCAACTCTTTTTCCGAA<br>GGTAACTGGCTTCAGCAGAGCGCAGATACCAAATACTGTTCTTCTAGTGTAGCCGT<br>AGTTAGGCCACCACTTCAAGAACTCTGTAGCACCGCCTACATACCTCGCTCTGCTA

	ATCCTGTTACCAGTGGCTGCTGCCAGTGGCGATAAGTCGTGTCTTACCGGGTTGG<br>ACTCAAGACGATAGTTACCGGATAAGGCGCAGCGGTCGGGCTGAACGGGGGGTT<br>CGTGCACACAGCCCAGCTTGGAGCGAACGACCTACACCGAACT
pIV011	GAGATACCTACAGCGTGAGCTATGAGAAAGCGCCACGCTTCCCGAAGGGAGAAAG<br>GCGGACAGGTATCCGGTAAGCGGCAGGGTCGGAACAGGAGAGCGCACGAGGGA<br>GCTTCCAGGGGGAAACGCCTGGTATCTTTATAGTCCTGTCGGGTTTCGCCACCTCT<br>GACTTGAGCGTCGATTTTTGTGATGCTCGTCAGGGGGGCGGAGCCTATGGAAAAA<br>CGCCAGCAACGCGGCCTTTTTACGGTTCCTGGCCTTTTGCTGGCCTTTTGCTCACA<br>TGTTCTTTCCTGCGTTATCCCCTGATTCTGTGGATAACCGTATTACCGCCTTTGAGT<br>GAGCTGATACCGCTCGCCGCAGCCGAACGACCGAGCGCAGCGAGTCAGTGAGCG<br>AGGAAGCGGAAGAGCGCCCAATACGCAAACCGCCTCTCCCCGCGCGTTGGCCGA<br>TTCATTAATGCAGCTGGCACGACAGGTTTCCCGACTGGAAAGCGGGCAGTGAGCG<br>CAACGCAATTAATGTGAGTTAGCTCACTCATTAGGCACCCCAGGCTTTACACTTTAT<br>GCTTCCGGCTCGTATGTTGTGTGGAATTGTGAGCGGATAACAATTTCACACAGGAA<br>ACAGCTATGACCATGATTACGCCAAGCTTGCATGCCTGCAGGTCGACTCTAGAaggtt<br>gatgcttctgggttccgtataccacaacaagaagctactaacaaggctcaaagtcaaggtgctctattaaagattcgtttagt<br>gatgtctgctccaatcgatgaaactttcgacaccaacgaaaccgccacaatcacttatttctctactgatttgaacaagattgt<br>cgagaaattcccaaaacaagccgaaaaattgtccgataccttagtgtttttgaaagatccaatgggcaacaacatcacctt<br>ctcaggcttagctaatgcaaccgattccgctccaacttccaaagatgctttcttagaagctacctccgaagacgaaatcatct<br>ctagagcttcttccgatgcttctgacttactaagacaaacattgggctcttctcaaaagaagaagaagattgctgtcatgactt<br>ctggtggtgattctccaggtatgaatgccgctgttcgtgccgttgttcgtacaggtatacatttcggctgtgatgtttttCTAGA<br>GGCATCGCACAGTTTTAGCGAGGAAAACTCTTCAATAGTTTTGCCAGCGGAATTCC<br>ACTTGCAATTACATAAAAAATTCCGGCGGTTTTTCGCGTGTGACTCAATGTCGAAAT<br>ACCTGCCTAATGAACATGAACATCGCCCAAATGTATTTGAAGACCCGCTGGGAGAA<br>GTTCAAGATATATAAGTAACAAGCAGCCAATAGTATAAAAAAAAATCTGAGTTTATTA<br>CCTTTCCTGGAATTTCAGTGAAAAACTGCTAATTATAGAGAGATATCACAGAGTTAC<br>TCACTAATGACTAACGAAAAGGTCTGGATAGAGAAGTTGGATAATCCAACTCTTTCA<br>GTGTTACCACATGACTTTTTACGCCCACAACAAGAACCTTATACGAAACAAGCTACA<br>TATTCGTTACAGCTACCTCAGCTCGATGTGCCTCATGATAGTTTTTCTAACAAATAC<br>GCTGTCGCTTTGAGTGTATGGGCTGCATTGATATATAGAGTAACCGGTGACGATGA<br>TATTGTTCTTTATATTGCGAATAACAAAATCTTAAGATTCAATATTCAACCAACGTGG<br>GTCATTTAATGAGCTGTATTCTACAATTAACAATGAGTTGAACAAGCTCAATTCTATT<br>GAGGCCAATTTTTCCTTTGACGAGCTAGCTGAAAAAATTCAAAGTTGCCAAGATCTG<br>GAAAGGACCCCTCAGTTGTTCCGTTTGGCCTTTTTGGAAAACCAAGATTTCAAATTA<br>GACGAGTTCAAGCATCATTTAGTGGACTTTGCTTTGAATTTGGATACCAGTAATAAT<br>GCGCATGTTTTGAACTTAATTTATAACAGCTTACTGTATTCGAATGAAAGAGTAACC<br>ATTGTTGCGGACCAATTTACTCAATATTTGACTGCTGCGCTAAGCGATCCATCCAAT<br>TGCATAACTAAAATCTCTCTGATCACCGCATCATCCAAGGATAGTTTACCTGATCCA<br>ACTAAGAACTTGGGCTGGTGCGATTTCGTGGGGTGTATTCACGACATTTTCCAGGA<br>CAATGCTGAAGCCTTCCCAGAGAGAACCTGTGTTGTGGAGACTCCAACACTAAATT<br>CCGACAAGTCCCGTTCTTTCACTTATCGCGACATCAACCGCACTTCTAACATAGTTG<br>CCCATTATTTGATTAAAACAGGTATCAAAAGAGGTGATGTAGTGATGATCTATTCTT<br>CTAGGGGTGTGGATTTGATGGTATGTGTGATGGGTGTCTTGAAAGCCGGCGCAAC<br>CTTTTCAGTTATCGACCCTGCATATCCCCCAGCCAGACAAACCATTTACTTAGGTGT<br>TGCTAAACCACGTGGGTTGATTGTTATTAGAGCTGCTGGACAATTGGATCAACTAG<br>TAGAAGATTACATCAATGATGAATTGGAGATTGTTTCAAGAATCAATTCCATCGCTA<br>TTCAAGAAAATGGTACCATTGAAGGTGGCAAATTGGACAATGGCGAGGATGTTTTG<br>GCTCCATATGATCACTACAAAGACACCAGAACAGGTGTTGTAGTTGGACCAGATTC<br>CAACCCAACCCTATCTTTCACATCTGGTTCCGAAGGTATTCCTAAGGGTGTTCTTG<br>GTAGACATTTTTCCTTGGCTTATTATTTCAATTGGATGTCCAAAAGGTTCAACTTAAC<br>AGAAAATGATAAATTCACAATGCTGAGCGGTATTGCACATGATCCAATTCAAAGAGA<br>TATGTTTACACCATTATTTTTAGGTGCCCAATTGTATGTCCCTACTCAAGATGATATT<br>GGTACACCGGGCCGTTTAGCGGAATGGATGAGTAAGTATGGTTGCACAGTTACCC<br>ATTTAACACCTGCCATGGGTCAATTACTTACTGCCCAAGCTACTACACCATTCCCTA<br>AGTTACATCATGCGTTCTTTGTGGGTGACATTTTAACAAAACGTGATTGTCTGAGGT<br>TACAAACCTTGGCAGAAAATTGCCGTATTGTTAATATGTACGGTACCACTGAAACAC<br>AGCGTGCAGTTTCTTATTTCGAAGTTAAATCAAAAAATGACGATCCAAACTTTTTGA<br>AAAAATTGAAAGATGTCATGCCTGCTGGTAAAGGTATGTTGAACGTTCAGCTACTA<br>GTTGTTAACAGGAACGATCGTACTCAAATATGTGGTATTGGCGAAATAGGTGAGAT<br>TTATGTTCGTGCAGGTGGTTTGGCCGAAGGTTATAGAGGATTACCAGAATTGAATA<br>AAGAAAAATTTGTGAACAACTGGTTTGTTGAAAAAGATCACTGGAATTATTTGGATA

	AGGATAATGGTGAACCTTGGAGACAATTCTGGTTAGGTCCAAGAGATAGATTGTAC<br>AGAACGGGTGATTTAGGTCGTTATCTACCAAACGGTGACTGTGAATGTTGCGGTAG<br>GGCTGATGATCAAGTTAAAATTCGTGGGTTCAGAATCGAATTAGGAGAAATAGATA<br>CGCACATTTCCCAACATCCATTGGTAAGAGAAAACATTACTTTAGTTCGCAAAAATG<br>CCGACAATGAGCCAACATTGATCACATTTATGGTCCCAAGATTTGACAAGCCAGAT<br>GACTTGTCTAAGTTCCAAAGTGATGTTCCAAAGGAGGTTGAAACTGACCCTATAGTT<br>AAGGGCTTAATCGGTTACCATCTTTTATCCAAGGACATCAGGACTTTCTTAAAGAAA<br>AGATTGGCTAGCTATGCTATGCCTTCCTTGATTGTGGTTATGGATAAACTACCATTG<br>AATCCAAATGGTAAAGTTGATAAGCCTAAACTTCAATTCCCAACTCCCAAGCAATTA<br>AATTTGGTAGCTGAAAATACAGTTTCTGAAACTGACGACTCTCAGTTTACCAATGTT<br>GAGCGCGAGGTTAGAGACTTATGGTTAAGTATATTACCTACCAAGCCAGCATCTGT<br>ATCACCAGATGATTCGTTTTTCGATTTAGGTGGTCATTCTATCTTGGCTACCAAAAT<br>GATTTTTACCTTAAAGAAAAAGCTGCAAGTTGATTTACCATTGGGCACAATTTTCAA<br>GTATCCAACGATAAAGGCCTTTGCCGCGGAAATTGACAGAATTAAATCATCGGGTG<br>GATCATCTCAAGGTGAGGTCGTCGAAAATGTCACTGCAAATTATGCGGAAGACGCC<br>AAGAAATTGGTTGAGACGCTACCAAGTTCGTACCCCTCTCGAGAATATTTTGTTGAA<br>CCTAATAGTGCCGAAGGAAAAACAACAATTAATGTGTTTGTTACCGGTGTCACAGG<br>ATTTCTGGGCTCCTACATCCTTGCAGATTTGTTAGGACGTTCTCCAAAGAACTACAG<br>TTTCAAAGTGTTTGCCCACGTCAGGGCCAAGGATGAAGAAGCTGCATTTGCAAGAT<br>TACAAAAGGCAGGTATCACCTATGGTACTTGGAACGAAAAATTTGCCTCAAATATTA<br>AAGTTGTATTAGGCGATTTATCTAAAAGCCAATTTGGTCTTTCAGATGAGAAGTGGA<br>TGGATTTGGCAAACACAGTTGATATAATTATCCATAATGGTGCGTTAGTTCACTGGG<br>TTTATCCATATGCCAAATTGAGGGATCCAAATGTTATTTCAACTATCAATGTTATGAG<br>CTTAGCCGCCGTCGGCAAGCCAAAGTTCTTTGACTTTGTTTCCTCCACTTCTACTCT<br>TGACACTGAATACTACTTTAATTTGTCAGATAAACTTGTTAGCGAAGGGAAGCCAGG<br>CATTTTAGAATCAGACGATTTAATGAACTCTGCAAGCGGGCTCACTGGTGGATATG<br>GTCAGTCCAAATGGGCTGCTGAGTACATCATTAGACGTGCAGGTGAAAGGGGCCT<br>ACGTGGGTGTATTGTCAGACCAGGTTACGTAACAGGTGCCTCTGCCAATGGTTCTT<br>CAAACACAGATGATTTCTTATTGAGATTTTTGAAAGGTTCAGTCCAATTAGGTAAGA<br>TTCCAGATATCGAAAATTCCGTGAATATGGTTCCAGTAGATCATGTTGCTCGTGTTG<br>TTGTTGCTACGTCTTTGAATCCTCCCAAAGAAAATGAATTGGCCGTTGCTCAAGTAA<br>CGGGTCACCCAAGAATATTATTCAAAGACTACTTGTATACTTTACACGATTATGGTT<br>ACGATGTCGAAATCGAAAGCTATTCTAAATGGAAGAAATCATTGGAGGCGTCTGTT<br>ATTGACAGGAATGAAGAAAATGCGTTGTATCCTTTGCTACACATGGTCTTAGACAAC<br>TTACCTGAAAGTACCAAAGCTCCGGAACTAGACGATAGGAACGCCGTGGCATCTTT<br>AAAGAAAGACACCGCATGGACAGGTGTTGATTGGTCTAATGGAATAGGTGTTACTC<br>CAGAAGAGGTTGGTATATATATTGCATTTTTAAACAAGGTTGGATTTTTACCTCCAC<br>CAACTCATAATGACAAACTTCCACTGCCAAGTATAGAACTAACTCAAGCGCAAATAA<br>GTCTAGTTGCTTCAGGTGCTGGTGCTCGTGGAAGCTCCGCAGCAGCTTAAGGTTG<br>AGCATTACGTATGATATGTCCATGTACAATAATTAAATATGAATTAGGAGAAAGACT<br>TAGCTTCTTTTCGGGTGATGTCACTTAAAAACTCCGAGAATAATATATAATAAGAGA<br>ATAAAATATTAGTTATTGAATAAGAACTGTAAATCAGgcatgcCCATGGGCTAGCCTCG<br>AGAGATCTCATAGCTTCAAAATGTTTCTACTCCTTTTTACTCTTCCAGATTTTCTCG<br>GACTCCGCGCATCGCCGTACCACTTCAAAACACCCAAGCACAGCATACTAAATTTC<br>CCCTCTTTCTTCCTCTAGGGTGTCGTTAATTACCCGTACTAAAGGTTTGGAAAAGAA<br>AAAAGAGACCGCCTCGTTTCTTTTTCTTCGTCGAAAAAGGCAATAAAAATTTTTATC<br>ACGTTTCTTTTTCTTGAAAATTTTTTTTTTGATTTTTTTCTCTTTCGATGACCTCCCAT<br>TGATATTTAAGTTAATAAACGGTCTTCAATTTCTCAAGTTTCAGTTTCATTTTTCTTGT<br>TCTATTACAACTTTTTTTACTTCTTGCTCATTAGAAAGAAAGCATAGCAATCTAATCT<br>AAGCCAAGCTAGATCTACCGGTCAAAATGTCTAAAGGTGAAGAATTATTCACTGGT<br>GTTGTCCCAATTTTGGTTGAATTAGATGGTGATGTTAATGGTCACAAATTTTCTGTC<br>TCCGGTGAAGGTGAAGGTGATGCTACTTACGGTAAATTGACCTTAAAATTTATTTGT<br>ACTACTGGTAAATTGCCAGTTCCATGGCCAACCTTAGTCACTACTTTCGGTTATGGT<br>GTTCAATGTTTTGCGAGATACCCAGATCATATGAAACAACATGACTTTTTCAAGTCT<br>GCCATGCCAGAAGGTTATGTTCAAGAAAGAACTATTTTTTTCAAAGATGACGGTAAC<br>TACAAGACCAGAGCTGAAGTCAAGTTTGAAGGTGATACCTTAGTTAATAGAATCGA<br>ATTAAAAGGTATTGATTTTAAAGAAGATGGTAACATTTTAGGTCACAAATTGGAATAC<br>AACTATAACTCTCACAATGTTTACATCATGGCTGACAAACAAAAGAATGGTATCAAA<br>GTTAACTTCAAAATTAGACACAACATTGAAGATGGTTCTGTTCAATTAGCTGACCAT<br>TATCAACAAAATACTCCAATTGGTGATGGTCCAGTCTTGTTACCAGACAACCATTAC<br>TTATCCACTCAATCTGCCTTATCCAAAGATCCAAACGAAAAGAGAGACCACATGGT

	CTTGTTAGAATTTGTTACTGCTGCTGGTATTACCCATGGTATGGATGAATTGTACAA<br>ATAACTCGAGTAAGCTTGGTACCGCGGCTAGCTAAGATCCGCTCTAACCGAAAAGG<br>AAGGAGTTAGACAACCTGAAGTCTAGGTCCCTATTTATTTTTTTATAGTTATGTTAGT<br>ATTAAGAACGTTATTTATATTTCAAATTTTTCTTTTTTTTCTGTACAGACGCGTGTAC<br>GCATGTAACATTATACTGAAAACCTTGCTTGAGAAGGTTTTGGGACGCTCGAAGGC<br>GGCCGCttggttaagtgaaggtggtactttgattggtactgctcgttctatggaattcagaaagcgtgagggtcgtagaca<br>agctgcaggcaatttaatttcgcaaggtattgacgctttggttgtttgtggtggtgatggttctttaaccggtgctgatcttttcaga<br>cacgaatggccatctttggttgatgaattggttgcagaaggtagattcactaaagaagaagtcgccccatacaagaatttgt<br>ccattgttggtcttgtcggttccatcgataatgatatgtctggtactgactctaccattggtgcttattctgctttggaaagaatctgt<br>gaaatggttgactacattgatgccaccgctaaatcccactcccgtgcctttgttgttgaagttatgggtagacattgtggttggtt<br>ggccttgatggctggtattgctaccggtgccgattacatttttattccagaaagagctgttcctcacggaaaatggcaggGG<br>ATCCCCGGGTACCGAGCTCGAATTCACTGGCCGTCGTTTTACAACGTCGTGACTG<br>GGAAAACCCTGGCGTTACCCAACTTAATCGCCTTGCAGCACATCCCCCTTTCGCCA<br>GCTGGCGTAATAGCGAAGAGGCCCGCACCGATCGCCCTTCCCAACAGTTGCGCAG<br>CCTGAATGGCGAATGGCGCCTGATGCGGTATTTTCTCCTTACGCATCTGTGCGGTA<br>TTTCACACCGCATATGGTGCACTCTCAGTACAATCTGCTCTGATGCCGCATAGTTAA<br>GCCAGCCCCGACACCCGCCAACACCCGCTGACGCGCCCTGACGGGCTTGTCTGC<br>TCCCGGCATCCGCTTACAGACAAGCTGTGACCGTCTCCGGGAGCTGCATGTGTCA<br>GAGGTTTTCACCGTCATCACCGAAACGCGCGAGACGAAAGGGCCTCGTGATACGC<br>CTATTTTTATAGGTTAATGTCATGATAATAATGGTTTCTTAGACGTCAGGTGGCACTT<br>TTCGGGGAAATGTGCGCGGAACCCCTATTTGTTTATTTTTCTAAATACATTCAAATA<br>TGTATCCGCTCATGAGACAATAACCCTGATAAATGCTTCAATAATATTGAAAAAGGA<br>AGAGTATGAGTATTCAACATTTCCGTGTCGCCCTTATTCCCTTTTTTGCGGCATTTT<br>GCCTTCCTGTTTTTGCTCACCCAGAAACGCTGGTGAAAGTAAAAGATGCTGAAGAT<br>CAGTTGGGTGCACGAGTGGGTTACATCGAACTGGATCTCAACAGCGGTAAGATCC<br>TTGAGAGTTTTCGCCCCGAAGAACGTTTTCCAATGATGAGCACTTTTAAAGTTCTGC<br>TATGTGGCGCGGTATTATCCCGTATTGACGCCGGGCAAGAGCAACTCGGTCGCCG<br>CATACACTATTCTCAGAATGACTTGGTTGAGTACTCACCAGTCACAGAAAAGCATCT<br>TACGGATGGCATGACAGTAAGAGAATTATGCAGTGCTGCCATAACCATGAGTGATA<br>ACACTGCGGCCAACTTACTTCTGACAACGATCGGAGGACCGAAGGAGCTAACCGC<br>TTTTTTGCACAACATGGGGGATCATGTAACTCGCCTTGATCGTTGGGAACCGGAGC<br>TGAATGAAGCCATACCAAACGACGAGCGTGACACCACGATGCCTGTAGCAATGGC<br>AACAACGTTGCGCAAACTATTAACTGGCGAACTACTTACTCTAGCTTCCCGGCAAC<br>AATTAATAGACTGGATGGAGGCGGATAAAGTTGCAGGACCACTTCTGCGCTCGGC<br>CCTTCCGGCTGGCTGGTTTATTGCTGATAAATCTGGAGCCGGTGAGCGTGGGTCT<br>CGCGGTATCATTGCAGCACTGGGGCCAGATGGTAAGCCCTCCCGTATCGTAGTTA<br>TCTACACGACGGGGAGTCAGGCAACTATGGATGAACGAAATAGACAGATCGCTGA<br>GATAGGTGCCTCACTGATTAAGCATTGGTAACTGTCAGACCAAGTTTACTCATATAT<br>ACTTTAGATTGATTTAAAACTTCATTTTTAATTTAAAAGGATCTAGGTGAAGATCCTT<br>TTTGATAATCTCATGACCAAAATCCCTTAACGTGAGTTTTCGTTCCACTGAGCGTCA<br>GACCCCGTAGAAAAGATCAAAGGATCTTCTTGAGATCCTTTTTTTCTGCGCGTAATC<br>TGCTGCTTGCAAACAAAAAAACCACCGCTACCAGCGGTGGTTTGTTTGCCGGATCA<br>AGAGCTACCAACTCTTTTTCCGAAGGTAACTGGCTTCAGCAGAGCGCAGATACCAA<br>ATACTGTTCTTCTAGTGTAGCCGTAGTTAGGCCACCACTTCAAGAACTCTGTAGCA<br>CCGCCTACATACCTCGCTCTGCTAATCCTGTTACCAGTGGCTGCTGCCAGTGGCG<br>ATAAGTCGTGTCTTACCGGGTTGGACTCAAGACGATAGTTACCGGATAAGGCGCA<br>GCGGTCGGGCTGAACGGGGGGTTCGTGCACACAGCCCAGCTTGGAGCGAACGAC<br>CTACACCGAACT
pIV012	GAGATACCTACAGCGTGAGCTATGAGAAAGCGCCACGCTTCCCGAAGGGAGAAAG<br>GCGGACAGGTATCCGGTAAGCGGCAGGGTCGGAACAGGAGAGCGCACGAGGGA<br>GCTTCCAGGGGGAAACGCCTGGTATCTTTATAGTCCTGTCGGGTTTCGCCACCTCT<br>GACTTGAGCGTCGATTTTTGTGATGCTCGTCAGGGGGGCGGAGCCTATGGAAAAA<br>CGCCAGCAACGCGGCCTTTTTACGGTTCCTGGCCTTTTGCTGGCCTTTTGCTCACA<br>TGTTCTTTCCTGCGTTATCCCCTGATTCTGTGGATAACCGTATTACCGCCTTTGAGT<br>GAGCTGATACCGCTCGCCGCAGCCGAACGACCGAGCGCAGCGAGTCAGTGAGCG<br>AGGAAGCGGAAGAGCGCCCAATACGCAAACCGCCTCTCCCCGCGCGTTGGCCGA<br>TTCATTAATGCAGCTGGCACGACAGGTTTCCCGACTGGAAAGCGGGCAGTGAGCG<br>CAACGCAATTAATGTGAGTTAGCTCACTCATTAGGCACCCCAGGCTTTACACTTTAT<br>GCTTCCGGCTCGTATGTTGTGTGGAATTGTGAGCGGATAACAATTTCACACAGGAA<br>ACAGCTATGACCATGATTACGCCAAGCTTGCATGCCTGCAGGTCGACTCTAGAaaag<br>attaaaaaaagtcaaagctacactggcatactttgaactgtttttcaaagctgtctccacgtggaaagtttatttcatcaaaac

tattatcaaaaattgtggttttgttaatacaaaagctgatatatatataatatggcatttagtgattttgctgccatatgctcaaaga
ccccgttgccattatgttcggtaataaagtctaaaacccatctaatactttcgaactcaacaattatacatgatttgatcctttaa
atttgaatgtcggtgtactgccacgctgttatgctcggtcgattgatcttgccaatacagtcatctttgatgtcgggaacgcattc
ataaatattggtgctctaggtgtcattttaatcatacttataacataagacagaagtatactgctattggcaggtctgaatatctc
tactttttccaactaacattgctattgataatatttaccttggtggtagactgtggtgtatctCgacatggaggcccagaataccc
tccttgacagtcttgacgtgcgcagctcaggggcatgatgtgactgtcgcccgtacatttagcccatacatccccatgtataat
catttgcatccatacatttgatggccgcacggcgcgaagcaaaaattacggctcctcgctgcagacctgcgagcaggga
aacgctcccctcacagacgcgttgaattgtccccacgccgcgcccctgtagagaaatataaaaggttaggatttgccactg
aggttcttctttcatatacttccttttaaaatcttgctaggatacagttctcacatcacatccgaacataaacaaccatgggtacc
actcttgacgacacggcttaccggtaccgcaccagtgtcccgggggacgccgaggccatcgaggcactggatgggtcct
tcaccaccgacaccgttttccgcgtcaccAccaccggggacggcttcaccctgcgggaggtgccggtggacccgcccct
gaccaaggtgttccccgacgacgaatcggacgacgaatcggacgacggggaggacggcgacccggactcccggac
gttcgtcgcgtacggggacgacggcgacctggcgggcttcgtggtcgtctcgtactccggctggaaccgccggctgaccg
tcgaggacatcgaggtcgccccggagcaccgggggcacggggtcgggcgcgcgttgatggggctcgcgacggagttc
gcccgcgagcggggcgccgggcacctctggctggaggtcaccaacgtcaacgcaccggcgatccacgcgtaccggc
ggatggggttcaccctctgcggcctggacaccgccctgtacgacggcaccgcctcggacggcgagcaggcgctctacat
gagcatgccctgcccctaatcagtactgacaataaaaagattcttgttttcaagaacttgtcatttgtatagtttttttatattgtagt
tgttctattttaatcaaatgttagcgtgatttatattttttttcgcctcgacatcatctgcccagatgcgaagttaagtgcgcagaaa
gtaatatcatgcgtcaatcgtatgtgaatgctggtcgctatactgCCATGGGCTAGCCTCGAGAGATCCCA
TAGCTTCAAAATGTTTCTACTCCTTTTTTACTCTTCCAGATTTTCTCGGACTCCGCGC
ATCGCCGTACCACTTCAAAACACCCAAGCACAGCATACTAAATTTCCCCTCTTTCTT
CCTCTAGGGTGTCGTTAATTACCCGTACTAAAGGTTTGGAAAAGAAAAAAGAGACC
GCCTCGTTTCTTTTTCTTCGTCGAAAAAGGCAATAAAAATTTTTATCACGTTTCTTTT
TCTTGAAAATTTTTTTTTTGATTTTTTTCTCTTTCGATGACCTCCCATTGATATTTAAG
TTAATAAACGGTCTTCAATTTCTCAAGTTTCAGTTTCATTTTTCTTGTTCTATTACAAC
TTTTTTTACTTCTTGCTCATTAGAAAGAAAGCATAGCAATCTAATCTAAGCCAAGCTA
GATCTACCGGTCAAAATGTCTAAAGGTGAAGAATTATTCACTGGTGTTGTCCCAATT
TTGGTTGAATTAGATGGTGATGTTAATGGTCACAAATTTTCTGTCTCCGGTGAAGGT
GAAGGTGATGCTACTTACGGTAAATTGACCTTAAAATTTATTTGTACTACTGGTAAA
TTGCCAGTTCCATGGCCAACCTTAGTCACTACTTTCGGTTATGGTGTTCAATGTTTT
GCGAGATACCCAGATCATATGAAACAACATGACTTTTTCAAGTCTGCCATGCCAGA
AGGTTATGTTCAAGAAAGAACTATTTTTTTCAAAGATGACGGTAACTACAAGACCAG
AGCTGAAGTCAAGTTTGAAGGTGATACCTTAGTTAATAGAATCGAATTAAAAGGTAT
TGATTTTAAAGAAGATGGTAACATTTTAGGTCACAAATTGGAATACAACTATAACTCT
CACAATGTTTACATCATGGCTGACAAACAAAAGAATGGTATCAAAGTTAACTTCAAA
ATTAGACACAACATTGAAGATGGTTCTGTTCAATTAGCTGACCATTATCAACAAAAT
ACTCCAATTGGTGATGGTCCAGTCTTGTTACCAGACAACCATTACTTATCCACTCAA
TCTGCCTTATCCAAAGATCCAAACGAAAAGAGAGACCACATGGTCTTGTTAGAATTT
GTTACTGCTGCTGGTATTACCCATGGTATGGATGAATTGTACAAATAACTCGAGTAA
GCTTGGTACCGCGGCTAGCTAAGATCCGCTCTAACCGAAAAGGAAGGAGTTAGAC
AACCTGAAGTCTAGGTCCCTATTTATTTTTTTATAGTTATGTTAGTATTAAGAACGTT
ATTTATATTTCAAATTTTTCTTTTTTTTCTGTACAGACGCGTGTACGCATGTAACATTA
TACTGAAAACCTTGCTTGAGAAGGTTTTGGGACGCTCGAAGGCGGCCGCcgggtgcat
gttgctgggccttattgataatcgggtttttaggtttcaatttatgggaagatgggactacaaagtccatgctgttggtccgtgga
acgtccatgctaggattcatagccaatttttagcctctatttaaccttcaaagcatggatcaccgaccataaagtagcaaca
atgaacgcttcagggatgattgtcgtcgtttacataataaacgccattttcttattcgttttcgttatttgtcaattactggtatcccta
ttggtagttcgaaacttatgggtcacaggagctatctttttggggctatttttctttgtagcaggccaggtattggtttatgccttctct
acacaaatttgtgaagggttcaagcactacttagatggcctctttttggaagcatctgtaatgtgttcacattaatgatggtttac
aagacttgggatatgactaccgacgacgacttggaatttggtgtaagtgttagcGGATCCCCGGGTACCGAG
CTCGAATTCACTGGCCGTCGTTTTACAACGTCGTGACTGGGAAAACCCTGGCGTTA
CCCAACTTAATCGCCTTGCAGCACATCCCCCTTTCGCCAGCTGGCGTAATAGCGAA
GAGGCCCGCACCGATCGCCCTTCCCAACAGTTGCGCAGCCTGAATGGCGAATGG
CGCCTGATGCGGTATTTTCTCCTTACGCATCTGTGCGGTATTTCACACCGCATATG
GTGCACTCTCAGTACAATCTGCTCTGATGCCGCATAGTTAAGCCAGCCCCGACACC
CGCCAACACCCGCTGACGCGCCCTGACGGGCTTGTCTGCTCCCGGCATCCGCTTA
CAGACAAGCTGTGACCGTCTCCGGGAGCTGCATGTGTCAGAGGTTTTCACCGTCA
TCACCGAAACGCGCGAGACGAAAGGGCCTCGTGATACGCCTATTTTTATAGGTTAA
TGTCATGATAATAATGGTTTCTTAGACGTCAGGTGGCACTTTTCGGGGAAATGTGC
GCGGAACCCCTATTTGTTTATTTTTCTAAATACATTCAAATATGTATCCGCTCATGAG
ACAATAACCCTGATAAATGCTTCAATAATATTGAAAAAGGAAGAGTATGAGTATTCA
ACATTTCCGTGTCGCCCTTATTCCCTTTTTTGCGGCATTTTGCCTTCCTGTTTTTGCT

	CACCCAGAAACGCTGGTGAAAGTAAAAGATGCTGAAGATCAGTTGGGTGCACGAG<br>TGGGTTACATCGAACTGGATCTCAACAGCGGTAAGATCCTTGAGAGTTTTCGCCCC<br>GAAGAACGTTTTCCAATGATGAGCACTTTTAAAGTTCTGCTATGTGGCGCGGTATTA<br>TCCCGTATTGACGCCGGGCAAGAGCAACTCGGTCGCCGCATACACTATTCTCAGA<br>ATGACTTGGTTGAGTACTCACCAGTCACAGAAAAGCATCTTACGGATGGCATGACA<br>GTAAGAGAATTATGCAGTGCTGCCATAACCATGAGTGATAACACTGCGGCCAACTT<br>ACTTCTGACAACGATCGGAGGACCGAAGGAGCTAACCGCTTTTTTGCACAACATGG<br>GGGATCATGTAACTCGCCTTGATCGTTGGGAACCGGAGCTGAATGAAGCCATACC<br>AAACGACGAGCGTGACACCACGATGCCTGTAGCAATGGCAACAACGTTGCGCAAA<br>CTATTAACTGGCGAACTACTTACTCTAGCTTCCCGGCAACAATTAATAGACTGGATG<br>GAGGCGGATAAAGTTGCAGGACCACTTCTGCGCTCGGCCCTTCCGGCTGGCTGGT<br>TTATTGCTGATAAATCTGGAGCCGGTGAGCGTGGGTCTCGCGGTATCATTGCAGCA<br>CTGGGGCCAGATGGTAAGCCCTCCCGTATCGTAGTTATCTACACGACGGGGAGTC<br>AGGCAACTATGGATGAACGAAATAGACAGATCGCTGAGATAGGTGCCTCACTGATT<br>AAGCATTGGTAACTGTCAGACCAAGTTTACTCATATATACTTTAGATTGATTTAAAAC<br>TTCATTTTTAATTTAAAAGGATCTAGGTGAAGATCCTTTTTGATAATCTCATGACCAA<br>AATCCCTTAACGTGAGTTTTCGTTCCACTGAGCGTCAGACCCCGTAGAAAAGATCA<br>AAGGATCTTCTTGAGATCCTTTTTTTCTGCGCGTAATCTGCTGCTTGCAAACAAAAA<br>AACCACCGCTACCAGCGGTGGTTTGTTTGCCGGATCAAGAGCTACCAACTCTTTTT<br>CCGAAGGTAACTGGCTTCAGCAGAGCGCAGATACCAAATACTGTTCTTCTAGTGTA<br>GCCGTAGTTAGGCCACCACTTCAAGAACTCTGTAGCACCGCCTACATACCTCGCTC<br>TGCTAATCCTGTTACCAGTGGCTGCTGCCAGTGGCGATAAGTCGTGTCTTACCGG<br>GTTGGACTCAAGACGATAGTTACCGGATAAGGCGCAGCGGTCGGGCTGAACGGG<br>GGGTTCGTGCACACAGCCCAGCTTGGAGCGAACGACCTACACCGAACT
pIV013	GAGATACCTACAGCGTGAGCTATGAGAAAGCGCCACGCTTCCCGAAGGGAGAAAG<br>GCGGACAGGTATCCGGTAAGCGGCAGGGTCGGAACAGGAGAGCGCACGAGGGA<br>GCTTCCAGGGGGAAACGCCTGGTATCTTTATAGTCCTGTCGGGTTTCGCCACCTCT<br>GACTTGAGCGTCGATTTTTGTGATGCTCGTCAGGGGGGCGGAGCCTATGGAAAAA<br>CGCCAGCAACGCGGCCTTTTTACGGTTCCTGGCCTTTTGCTGGCCTTTTGCTCACA<br>TGTTCTTTCCTGCGTTATCCCCTGATTCTGTGGATAACCGTATTACCGCCTTTGAGT<br>GAGCTGATACCGCTCGCCGCAGCCGAACGACCGAGCGCAGCGAGTCAGTGAGCG<br>AGGAAGCGGAAGAGCGCCCAATACGCAAACCGCCTCTCCCCGCGCGTTGGCCGA<br>TTCATTAATGCAGCTGGCACGACAGGTTTCCCGACTGGAAAGCGGGCAGTGAGCG<br>CAACGCAATTAATGTGAGTTAGCTCACTCATTAGGCACCCCAGGCTTTACACTTTAT<br>GCTTCCGGCTCGTATGTTGTGTGGAATTGTGAGCGGATAACAATTTCACACAGGAA<br>ACAGCTATGACCATGATTACGCCAAGCTTGCATGCCTGCAGGTCGACTCTAGACAA<br>CGCAAAATAAATAATTAATATGTAAAATATCTGTTTACTTGAAATTTACCCTTAACAG<br>AGGTACTAACGGGAGGAAGGACTGGCCTTGCTCCGGTAGACCTCGATGCCTGAAC<br>CTTCTCCCTCATCTCGAAACGGTACGACCTCTGAGAAAACGATATCAATCAATGCG<br>CGTCTTCCAGATGAAATTCTTCGGAATGCCCATAATACGAAAAACTAACGATGGAAA<br>AAATACTATAAATGTATGGGTGTAATTTTAACCGAATGTATGGATGTTACAGGGAGC<br>TTTATATATGTAGAAAATTCCAGACGTTCCAAACAAATAAGTGAAAATCAACACGATT<br>GGGAAAGTACATGCCAGAGTTCGATGTTTAGGTTAATGATAAAAATAATTTTTCTCT<br>AATCACCCTCCATTTTCGATAAAATATCAGCGTAGTCTGAAAATATATACATTAAGCA<br>AAAAGAATGTCAAACAAGTTTCACTGTGACGTTTGTTCAGCCGCTTAACTATGCGG<br>CATCAGAGCAGATTGTACTGAGAGTGCACCATAGATCAACGACATTACTATATATAT<br>AATATAGGAAGCATTTAATAGAACAGCATCGTAATATATGTGTACTTTGCAGTTATG<br>ACGCCAGATGGCAGTAGTGGAAGATATTCTTTATTGAAAAATAGCTTGTCACCTTAC<br>GTACAATCTTGATCCGGAGCTTTTCTTTTTTTGCCGATTAAGAATTAATTCGGTCGA<br>AAAAAGAAAAGGAGAGGGCCAAGAGGGAGGGCATTGGTGACTATTGAGCACGTGA<br>GTATACGTGATTAAGCACACAAAGGCAGCTTGGAGTATGTCTGTTATTAATTTCACA<br>GGTAGTTCTGGTCCATTGGTGAAAGTTTGCGGCTTGCAGAGCACAGAGGCCGCAG<br>AATGTGCTCTAGATTCCGATGCTGACTTGCTGGGTATTATATGTGTGCCCAATAGAA<br>AGAGAACAATTGACCCGGTTATTGCAAGGAAAATTTCAAGTCTTGTAAAAGCATATA<br>AAAATAGTTCAGGCACTCCGAAATACTTGGTTGGCGTGTTTCGTAATCAACCTAAG<br>GAGGATGTTTTGGCTCTGGTCAATGATTACGGCATTGATATCGTCCAACTGCATGG<br>AGATGAGTCGTGGCAAGAATACCAAGAGTTCCTCGGTTTGCCAGTTATTAAAAGAC<br>TCGTATTTCCAAAAGACTGCAACATACTACTCAGTGCAGCTTCACAGAAACCTCATT<br>CGTTTATTCCCTTGTTTGATTCAGAAGCAGGTGGGACAGGTGAACTTTTGGATTGG<br>AACTCGATTTCTGACTGGGTTGGAAGGCAAGAGAGCCCCGAAAGCTTACATTTTAT<br>GTTAGCTGGTGGACTGACGCCAGAAAATGTTGGTGATGCGCTTAGATTAAATGGCG

	TTATTGGTGTTGATGTAAGCGGAGGTGTGGAGACAAATGGTGTAAAAGACTCTAAC<br>AAAATAGCAAATTTCGTCAAAAATGCTAAGAAATAGGTTATTACTGAGTAGTATTTAT<br>TTAAGTATTGTTTGTGCACTTGCCGATCTATGCGGTGTGAAATACCGCACAGATGC<br>GTAAGGAGAAAATACCGCATCAGGAAATTGTAAACGTTAATCCATGGGCTAGCCGT<br>AGTTTAGTGTTTTTCTTCCAGTGCGAGTATCTTTCTTGCAAATCGATGGGTATACTG<br>GAGGCTTCATGAGTTATGTCCTTCGCGCACTGATTCATCTTTGAAACTAAATTTGTA<br>TATTGTTTGTCAGAAATAGGACCTGATATCCTCCTTTTAAACAGTTGATGAGAACCT<br>TTTTCGCAAGTTCAAGGTGCTCTAATTTTTAAAATTTTTACTTTTCGCGACACAATAA<br>AGTCTTCACGACGCTAAACTATTAGTGCACATAATGTAGTTACTTGGACGCTGTTCA<br>ATAATGTATAAAATTTATTTCCTTTGCATTACGTACATTATATAACCAAATCTTAAAAA<br>TATAGAAATATGATATGTGTATAATAATATAAGCAAAATTTACGTATCTTTGCTTATAA<br>TATAGCTTTAATGTTCTTTAGGTATATATTTAAGAGCGATTTGTCTACACACCGATAA<br>CAGGTATTGTGGACTTTTCGGTTTCTGGTTTGCCGAACACTGAGGCCTCTTCAGCA<br>TAGCTCTTTTTTAACAATTCTGCTACTCTCTCGGCAACAGAGCATTTGGCTGCAGGA<br>CTCACACCATGCTTTCCAATAATGGATTCAAGGACGGCTTCTGATAGTTGTTTGTTC<br>TCGATTACAGCATTTGCAGTACCGGTAACTGAATCGTCCTTGGCAAAGCTGATGGT<br>TAACAAGCCTAATGGACTCTGAGTAAACATAATGGAAGCACCAGGTGGGAATGTTT<br>CTGATCTAAACACATCCAAGAATTTCTCGATTGCCCTTCCTTCGTCATCAGTGTAGG<br>TACCTATGCCCTTCCAGTGAGCAACACAGTTTTCGGCGACTTTCTCAGAATATTGCT<br>TTCCTGTAAGAGGTAGAATCATAGTTACTCTGGTAAACTTTTCGAATGGACCTGTAA<br>CCACGTCCCTAAAGAACTCGACGCTATCAGTTAACTCCTGAGGGGTCTTACCTTTC<br>CACTTTTCTGCCAAAAATGGGATAGCACTTTCCTCAAGGTATACGCCTATGGCTGT<br>GAATTTCACAAACTTTCCTTCAATCTCTAGACCTCTATGGCCTGCTCCAGCTAAGAA<br>CAAAGTATTGGTGGAACCGGCAGGGTTCACTGTTGGTGCAAAAGCATAATTTTCGA<br>CTTGCATTTTTGTAACAGAAACTGGTGGAGACATTTTTAGGCTGGTATCTTGATTCT<br>AAATCGATTTTGCAAGACTAGAGGAATATGTATTAAAATTCACTGAATGCAAGTAAA<br>ATATTAAGTAAAAGAAAGCATTGATTATGAAGAAAGGACCTTTTTAAGATATGAGAA<br>CAATGAGAAAAGGAAAAATGGGACGAAACAAATAGGGAAAATTCCGTCTGTAAAAC<br>CAATTGTAGTAACAAAAAACGCTCATTTCAATAGCAAAAAAATAGAATACCGTCAAT<br>GGATATATATAGACACGGGACATTATAACAACGTGCAGATGGTGATGATTGCAAAC<br>CCAAAAAAATTACCAGCTTCCTGTAATTCCAGGTTGAGCGCGTGGCTTCCTATAATC<br>CAATACACCCACACCCACCCAGATCGTGATTTTTGTTACATAACAATACTGACAGTA<br>CTAAATAATTGCCTACTTGGCTTCACATACGTTGCATACGTCGATATAGATAATAAT<br>GATAATGACAGCAGGATTATCGTAATACGTAATAGTTGAAAATCTCAAAAATGTGTG<br>GGTCATTACGTAAATAATGATAGGAATGGGATTCTTCTATTTTTCCTTTTTCCATTCT<br>AGCAGCCGTCGGGAAAACGTGGCATCCTCTCTTTCGGGCTCAATTGGAGTCACGC<br>TGCCGTGAGCATCCTCTCTTTCCATATCTAACAACTGAGCACGTAACCAATGGAAA<br>AGCATGAGCTTAGCGTTGCTCCAAAAAAGTATTGGATGGTTAATACCATTTGTCTGT<br>TCTCTTCTGACTTTGACTCCTCAAAAAAAAAAATCTACAATCAACAGATCGCTTCAAT<br>TACGCCCTCACAAAAACTTTTTTCCTTCTTCTTCGCCCACGTTAAATTTTATCCCTCA<br>TGTTGTCTAACGGATTTCTGCACTTGATTTATTATAAAAAGACAAAGACATAATACTT<br>CTCTATCAATTTCAGTTATTGTTCTTCCTTGCGTTATTCTTCTGTTCTTCTTTTTCTTT<br>TGTCATATATAACCATAACCAAGTAATACATATTCAAAATGGATTTGTTATTGCTGGA<br>AAAGTCACTTATTGCTGTATTTGTGGCAGTTATTCTAGCCACGGTTATTTCTAAATTA<br>AGAGGTAAGAAACTAAAACTACCTCCTGGTCCCATCCCCATACCAATTTTTGGTAAT<br>TGGTTGCAAGTGGGCGATGATTTGAATCACAGAAATTTGGTAGACTATGCTAAGAA<br>GTTCGGTGACCTTTTCTTGCTTAGAATGGGTCAAAGGAATTTGGTAGTGGTTAGCT<br>CACCTGATTTGACTAAGGAGGTCTTATTAACGCAAGGCGTTGAGTTTGGCTCCAGA<br>ACTAGAAATGTTGTGTTTGATATTTTCACTGGTAAAGGTCAAGATATGGTTTTTACA<br>GTTTACGGTGAGCACTGGAGAAAAATGAGAAGAATCATGACCGTACCATTCTTTAC<br>TAACAAGGTTGTTCAACAAAATAGAGAAGGTTGGGAGTTTGAGGCAGCTTCCGTAG<br>TGGAAGACGTAAAGAAAAATCCAGATTCGGCCACAAAGGGTATAGTACTAAGAAAA<br>AGACTACAATTGATGATGTACAACAATATGTTCAGAATTATGTTTGACAGAAGATTT<br>GAAAGTGAAGATGACCCTTTGTTCCTGAGACTTAAGGCTTTGAATGGTGAAAGATC<br>GAGATTGGCTCAAAGTTTCGAATATAATTACGGTGACTTTATTCCAATCTTAAGACC<br>ATTTTTGAGAGGCTATTTGAAAATTTGCCAAGACGTCAAGGATAGGAGGATCGCTC<br>TTTTCAAGAAGTACTTTGTGGACGAGAGAAAGCAAATAGCTTCTTCCAAGCCCACA<br>GGTTCGGAAGGTTTAAAATGTGCAATTGATCATATTTTAGAAGCTGAACAAAAAGGT<br>GAAATTAACGAAGATAATGTTTTGTACATTGTAGAAAATATCAATGTGGCTGCAATA<br>GAAACAACCTTATGGTCAATAGAATGGGGTATTGCTGAATTGGTGAATCACCCAGA<br>AATACAATCTAAACTGAGAAACGAGCTAGATACCGTTTTAGGTCCAGGTGTCCAAG

	TTACAGAACCTGATTTGCATAAGTTACCCTACTTGCAAGCTGTGGTTAAAGAAACCT<br>TGAGATTGAGAATGGCTATTCCTCTTCTAGTTCCTCATATGAACCTACATGATGCTA<br>AACTGGCCGGTTATGATATTCCAGCAGAAAGTAAGATTTTAGTAAATGCATGGTGG<br>TTGGCCAACAATCCAAACAGTTGGAAAAAGCCTGAAGAATTCAGACCTGAAAGATT<br>CTTCGAAGAGGAATCTCATGTTGAAGCCAACGGAAATGACTTCAGATATGTACCTTT<br>TGGCGTTGGCAGAAGATCGTGTCCAGGAATAATACTAGCCTTACCAATATTGGGTA<br>TCACAATTGGTAGGATGGTTCAAAATTTTGAGTTGCTACCACCACCCGGACAATCG<br>AAAGTCGATACTTCAGAGAAAGGAGGACAATTCTCATTGCATATTTTGAATCATTCC<br>ATTATAGTCATGAAACCCAGAAATTGTAGCGCTGAAGCTGCAGCAAAAGAAGCTGC<br>AGCTAAAGAAGCTGCAGCAAAAGCTGGTAGGAGGAGCGGTTCGGGCAATTCAAAG<br>AGGGTTGAACCACTAAAGCCATTAGTTATCAAACCTAGAGAAGAGGAAATTGACGA<br>TGGAAGGAAGAAAGTCACTATATTCTTCGGCACCCAAACAGGTACAGCTGAAGGTT<br>TTGCTAAGGCTCTAGGAGAAGAAGCAAAAGCTAGATATGAAAAGACGAGATTCAAA<br>ATTGTCGATCTGGATGACTATGCCGCCGATGATGACGAATACGAAGAAAAATTGAA<br>GAAAGAAGATGTCGCATTTTTCTTCCTTGCCACCTACGGCGACGGTGAACCAACAG<br>ATAATGCCGCAAGGTTTTACAAGTGGTTTACTGAAGGTAATGACAGAGGAGAATGG<br>CTGAAGAATTTGAAATATGGTGTGTTCGGCCTTGGTAACAGACAGTACGAGCATTT<br>TAATAAGGTCGCTAAGGTTGTAGATGATATACTTGTTGAACAAGGTGCTCAAAGGTT<br>AGTGCAGGTGGGCTTGGGTGACGATGATCAATGTATTGAAGATGACTTTACTGCTT<br>GGAGAGAAGCCTTGTGGCCTGAATTAGATACTATCCTTAGAGAAGAAGGTGACACT<br>GCTGTTGCTACCCCCTACACTGCAGCAGTCCTAGAATATAGAGTCTCAATCCATGA<br>TTCAGAAGACGCCAAATTCAATGATATTAACATGGCCAACGGTAACGGTTACACCG<br>TTTTTGACGCACAACATCCATACAAAGCTAATGTTGCTGTTAAAAGGGAACTTCACA<br>CCCCAGAAAGTGACAGGTCATGTATACATTTGGAATTTGATATCGCTGGTAGTGGT<br>TTGACTTACGAAACAGGTGACCATGTCGGAGTACTTTGCGATAATTTGTCAGAAACT<br>GTTGATGAAGCTTTGAGGTTATTGGATATGTCACCAGATACTTACTTCTCATTGCAT<br>GCAGAAAAAGAAGACGGAACTCCAATATCAAGCTCGCTTCCCCCTCCATTCCCTCC<br>CTGTAACTTAAGAACAGCCCTAACTAGATATGCTTGTTTACTGTCTTCTCCAAAGAA<br>AAGTGCTTTGGTTGCATTGGCAGCCCACGCATCCGATCCTACCGAAGCTGAGAGA<br>TTAAAGCATTTGGCTTCACCAGCCGGTAAAGATGAATACAGTAAGTGGGTAGTGGA<br>GAGCCAAAGATCGCTTTTAGAAGTGATGGCTGAGTTTCCAAGTGCTAAACCTCCTC<br>TGGGTGTATTTTTCGCTGGTGTGGCCCCAAGATTGCAGCCTAGATTTTATTCCATAT<br>CCTCATCTCCAAAAATTGCCGAAACCAGAATTCACGTGACATGTGCTCTGGTCTAC<br>GAAAAGATGCCAACAGGTAGGATTCACAAGGGTGTCTGTTCTACCTGGATGAAAAA<br>TGCTGTACCCTATGAAAAATCCGAAAATTGTTCTAGTGCACCAATTTTCGTAAGACA<br>ATCTAATTTCAAGTTACCAAGCGATTCTAAAGTACCCATTATTATGATCGGTCCAGG<br>TACTGGTTTGGCCCCATTCAGAGGCTTCTTGCAAGAAAGATTGGCTTTAGTGGAGA<br>GTGGAGTTGAATTGGGTCCTTCAGTTTTATTCTTTGGTTGTAGAAACAGAAGAATGG<br>ACTTTATCTACGAAGAAGAATTGCAGAGATTTGTTGAAAGTGGTGCATTGGCCGAA<br>TTGAGTGTTGCATTCAGCAGGGAAGGTCCAACCAAAGAATACGTTCAACACAAGAT<br>GATGGACAAGGCTTCTGATATCTGGAATATGATTTCCCAAGGTGCTTATTTGTATGT<br>TTGTGGTGACGCTAAAGGAATGGCTAGAGATGTTCATAGATCACTGCATACAATCG<br>CACAAGAACAAGGTAGCATGGATTCAACAAAAGCAGAGGGCTTTGTAAAGAATCTT<br>CAGACAAGCGGTAGATATCTGAGAGATGTATGGTAAATCGCGTGGTTAATTCAAAT<br>TAATTGATATAGTTTTTTAATGAGTATTGAATCTGTTTAGAAATAATGGAATATTATTT<br>TTATTTATTTATTTATATTATTGGTCGGCTCTTTTCTTCTGAAGGTCAATGACAAAAT<br>GATATGAAGGAAATAATGATTTCTAAAATTTTACAACGTAAGATATTTTTACAAAAGC<br>CTAGCTCATCTTTTGTCATGCACTATTTTACTCACGCTTGAAATTAACGGCCAGTCC<br>ACTTTGGAGTCATTTCAAAGTCATCCTAATCGATCTATCGTTTTTGATAGCTCGAGA<br>GATCTGCGGCCGCTACAGGAAAGCATCGCCCTTACCATGATTATAGGATAATAGAG<br>ACCAATTCATACCCCATTCTCTGTCCTGATTGGGGTGCCGATGAAGAATTGCAGTT<br>GATTAAAGGCGCACAGACTTTAGGGCTCGGTAATTGGCAGGATATTGCTGACCATA<br>TAGGCAGCAGAGGCAAAGAAGAAGTTAAGGAACATTACCTAAAATATTATCTGGAA<br>AGCAAATACTATCCAATACCTGATATTACCCAAAATATACATGTCCCACAAGATGAA<br>TTTTTGGAACAGCGAAGGCATAGAATCGAGTCCTTCCGGGAGAGGCCGCTAGAGC<br>CTCCAAGAAAGCCCATGGCATCGGTTCCTAGCTGCCATGAAGTACAGGGGTTTATG<br>CCGGGCAGATTAGAATTTGAAACAGAATTCGAAAATGAAGCTGAAGGTCCCGTAAA<br>GGATATGGTTTTTGAACCTGATGACCAGCCCTTAGACATTGAATTGAAGTTTGCCAT<br>CTTGGACAGGATCCCCGGGTACCGAGCTCGAATTCACTGGCCGTCGTTTTACAAC<br>GTCGTGACTGGGAAAACCCTGGCGTTACCCAACTTAATCGCCTTGCAGCACATCCC<br>CCTTTCGCCAGCTGGCGTAATAGCGAAGAGGCCCGCACCGATCGCCCTTCCCAAC

	AGTTGCGCAGCCTGAATGGCGAATGGCGCCTGATGCGGTATTTTCTCCTTACGCAT<br>CTGTGCGGTATTTCACACCGCATATGGTGCACTCTCAGTACAATCTGCTCTGATGC<br>CGCATAGTTAAGCCAGCCCCGACACCCGCCAACACCCGCTGACGCGCCCTGACG<br>GGCTTGTCTGCTCCCGGCATCCGCTTACAGACAAGCTGTGACCGTCTCCGGGAGC<br>TGCATGTGTCAGAGGTTTTCACCGTCATCACCGAAACGCGCGAGACGAAAGGGCC<br>TCGTGATACGCCTATTTTTATAGGTTAATGTCATGATAATAATGGTTTCTTAGACGTC<br>AGGTGGCACTTTTCGGGGAAATGTGCGCGGAACCCCTATTTGTTTATTTTTCTAAAT<br>ACATTCAAATATGTATCCGCTCATGAGACAATAACCCTGATAAATGCTTCAATAATAT<br>TGAAAAAGGAAGAGTATGAGTATTCAACATTTCCGTGTCGCCCTTATTCCCTTTTTT<br>GCGGCATTTTGCCTTCCTGTTTTTGCTCACCCAGAAACGCTGGTGAAAGTAAAAGA<br>TGCTGAAGATCAGTTGGGTGCACGAGTGGGTTACATCGAACTGGATCTCAACAGC<br>GGTAAGATCCTTGAGAGTTTTCGCCCCGAAGAACGTTTTCCAATGATGAGCACTTT<br>TAAAGTTCTGCTATGTGGCGCGGTATTATCCCGTATTGACGCCGGGCAAGAGCAAC<br>TCGGTCGCCGCATACACTATTCTCAGAATGACTTGGTTGAGTACTCACCAGTCACA<br>GAAAAGCATCTTACGGATGGCATGACAGTAAGAGAATTATGCAGTGCTGCCATAAC<br>CATGAGTGATAACACTGCGGCCAACTTACTTCTGACAACGATCGGAGGACCGAAG<br>GAGCTAACCGCTTTTTTGCACAACATGGGGGATCATGTAACTCGCCTTGATCGTTG<br>GGAACCGGAGCTGAATGAAGCCATACCAAACGACGAGCGTGACACCACGATGCCT<br>GTAGCAATGGCAACAACGTTGCGCAAACTATTAACTGGCGAACTACTTACTCTAGC<br>TTCCCGGCAACAATTAATAGACTGGATGGAGGCGGATAAAGTTGCAGGACCACTTC<br>TGCGCTCGGCCCTTCCGGCTGGCTGGTTTATTGCTGATAAATCTGGAGCCGGTGA<br>GCGTGGGTCTCGCGGTATCATTGCAGCACTGGGGCCAGATGGTAAGCCCTCCCGT<br>ATCGTAGTTATCTACACGACGGGGAGTCAGGCAACTATGGATGAACGAAATAGACA<br>GATCGCTGAGATAGGTGCCTCACTGATTAAGCATTGGTAACTGTCAGACCAAGTTT<br>ACTCATATATACTTTAGATTGATTTAAAACTTCATTTTTAATTTAAAAGGATCTAGGTG<br>AAGATCCTTTTTGATAATCTCATGACCAAAATCCCTTAACGTGAGTTTTCGTTCCACT<br>GAGCGTCAGACCCCGTAGAAAAGATCAAAGGATCTTCTTGAGATCCTTTTTTTCTG<br>CGCGTAATCTGCTGCTTGCAAACAAAAAAACCACCGCTACCAGCGGTGGTTTGTTT<br>GCCGGATCAAGAGCTACCAACTCTTTTTCCGAAGGTAACTGGCTTCAGCAGAGCG<br>CAGATACCAAATACTGTTCTTCTAGTGTAGCCGTAGTTAGGCCACCACTTCAAGAA<br>CTCTGTAGCACCGCCTACATACCTCGCTCTGCTAATCCTGTTACCAGTGGCTGCTG<br>CCAGTGGCGATAAGTCGTGTCTTACCGGGTTGGACTCAAGACGATAGTTACCGGA<br>TAAGGCGCAGCGGTCGGGCTGAACGGGGGGTTCGTGCACACAGCCCAGCTTGGA<br>GCGAACGACCTACACCGAACT
pIV014	GAGATACCTACAGCGTGAGCTATGAGAAAGCGCCACGCTTCCCGAAGGGAGAAAG<br>GCGGACAGGTATCCGGTAAGCGGCAGGGTCGGAACAGGAGAGCGCACGAGGGA<br>GCTTCCAGGGGGAAACGCCTGGTATCTTTATAGTCCTGTCGGGTTTCGCCACCTCT<br>GACTTGAGCGTCGATTTTTGTGATGCTCGTCAGGGGGGCGGAGCCTATGGAAAAA<br>CGCCAGCAACGCGGCCTTTTTACGGTTCCTGGCCTTTTGCTGGCCTTTTGCTCACA<br>TGTTCTTTCCTGCGTTATCCCCTGATTCTGTGGATAACCGTATTACCGCCTTTGAGT<br>GAGCTGATACCGCTCGCCGCAGCCGAACGACCGAGCGCAGCGAGTCAGTGAGCG<br>AGGAAGCGGAAGAGCGCCCAATACGCAAACCGCCTCTCCCCGCGCGTTGGCCGA<br>TTCATTAATGCAGCTGGCACGACAGGTTTCCCGACTGGAAAGCGGGCAGTGAGCG<br>CAACGCAATTAATGTGAGTTAGCTCACTCATTAGGCACCCCAGGCTTTACACTTTAT<br>GCTTCCGGCTCGTATGTTGTGTGGAATTGTGAGCGGATAACAATTTCACACAGGAA<br>ACAGCTATGACCATGATTACGCCAAGCTTGCATGCCTGCAGGTCGACTCTAGAAAA<br>GATTAAAAAAAGTCAAAGCTACACTGGCATACTTTGAACTGTTTTTTCAAAGCTGTC<br>TCCACGTGGAAAGTTTATTTCATCAAAACTATTATCAAAAATTGTGGTTTTGTTAATA<br>CAAAAGCTGATATATATATAATATGGCATTTAGTGATTTTGCTGCCATATGCTCAAA<br>GACCCCGTTGCCATTATGTTCGGTAATAAAGTCTAAAACCCATCTAATACTTTCGAA<br>CTCAACAATTATACATGATTTTGATCCTTTAAATTTGAATGTCGGTGTACTGCCACG<br>CTGTTATGCTCGGTCGATTGATCTTGCCAATACAGTCATCTTTGATGTCGGGAACG<br>CATTCATAAATATTGGTGCTCTAGGTGTCATTTTAATCATACTTTATAACATAAGACA<br>GAAGTATACTGCTATTGGCAGGTCTGAATATCTCTACTTTTTCCAACTAACATTGCT<br>ATTGATAATATTTACCTTGGTGGTAGACTGTGGTGTATCTCGACATGGAGGCCCAG<br>AATACCCTCCTTGACAGTCTTGACGTGCGCAGCTCAGGGGCATGATGTGACTGTC<br>GCCCGTACATTTAGCCCATACATCCCCATGTATAATCATTTGCATCCATACATTTTG<br>ATGGCCGCACGGCGCGAAGCAAAAATTACGGCTCCTCGCTGCAGACCTGCGAGCA<br>GGGAAACGCTCCCCTCACAGACGCGTTGAATTGTCCCCACGCCGCGCCCCTGTAG<br>AGAAATATAAAAGGTTAGGATTTGCCACTGAGGTTCTTCTTTCATATACTTCCTTTTA<br>AAATCTTGCTAGGATACAGTTCTCACATCACATCCGAACATAAACAACCATGGGTAC

	CACTCTTGACGACACGGCTTACCGGTACCGCACCAGTGTCCCGGGGGACGCCGA<br>GGCCATCGAGGCACTGGATGGGTCCTTCACCACCGACACCGTTTTCCGCGTCACC<br>ACCACCGGGGACGGCTTCACCCTGCGGGAGGTGCCGGTGGACCCGCCCCTGACC<br>AAGGTGTTCCCCGACGACGAATCGGACGACGAATCGGACGACGGGGAGGACGGC<br>GACCCGGACTCCCGGACGTTCGTCGCGTACGGGGACGACGGCGACCTGGCGGG<br>CTTCGTGGTCGTCTCGTACTCCGGCTGGAACCGCCGGCTGACCGTCGAGGACATC<br>GAGGTCGCCCCGGAGCACCGGGGGCACGGGGTCGGGCGCGCGTTGATGGGGCT<br>CGCGACGGAGTTCGCCCGCGAGCGGGGCGCCGGGCACCTCTGGCTGGAGGTCA<br>CCAACGTCAACGCACCGGCGATCCACGCGTACCGGCGGATGGGGTTCACCCTCT<br>GCGGCCTGGACACCGCCCTGTACGACGGCACCGCCTCGGACGGCGAGCAGGCG<br>CTCTACATGAGCATGCCCTGCCCCTAATCAGTACTGACAATAAAAAGATTCTTGTTT<br>TCAAGAACTTGTCATTTGTATAGTTTTTTTATATTGTAGTTGTTCTATTTTAATCAAAT<br>GTTAGCGTGATTTATATTTTTTTTCGCCTCGACATCATCTGCCCAGATGCGAAGTTA<br>AGTGCGCAGAAAGTAATATCATGCGTCAATCGTATGTGAATGCTGGTCGCTATACT<br>GCCATGGGCTAGCCATCCTCTAGAGAGGCATTAAAAGAGGAGCGAATTTTTTTTTA<br>ATAAAAATCTTAATAATCATTAAAAGATAAATAATAGTCTATATATACGTATATAAATA<br>AAAAATATTCAAAAAATAAAATAAACTATTATTTTAGCGTAAAGGATGGGGAAAGAG<br>AAAAGAAAAAAATTGATCTATCGATTTCAATTCAATTCAATCTAGTTCATTAATCCAT<br>TTGCTAGTCTTGCTCTTAGATCCTTCCTCAATATCTTCCCTGATGGAGCTTTAGGAA<br>TAGAGTCAGTGAAGAACACTTTGTTGATTCTCTTATAAAACACAACCTGTTTTGACA<br>CGAATTGCTTGATTTCATCTTCGGATATATTTGAATCTTTCGATCTCACCACAAACG<br>CAACAGGAACCTCACCAGCATCTTCTTCCTTCATGGCGACGACAGCAACATCATTG<br>ATTTCTGGATGACCTATGAGGAGAGACTCTAGCTCAGCTGGAGCCACTTGAAATCC<br>TTTGTACTTGATGAGTTCTTTCAATCTATCCACAATGAAAAGCTCGTCGTCATCATC<br>GATAAATCCGACGTCTCCAGTGTGAAGCCAACCATCTTTATCGATCGTCGATGCCG<br>TGGCCAAGGGGTCATTGAGATAGCCTTTCATGATTTGGTTGCCACGGATGCATATT<br>TCGCCGGGTTTGTTCCTAGGCAAAGAATCTCCTGTGTCTGGATCAAGTATCTTCAT<br>CTCGGCGTTCCTCACCACCGTACCACATGCTCCTGACTTCACTGGAAACGGCTCTT<br>TAGCAAACCCTAACGACATTGCTAGCACCGGACCTGCTTCTGTCATCCCATAGCCC<br>TGACCAAGCTTGGCGTTAGGAAACTTAGCACTAATAGCATCTTCAAGCTCCTTACC<br>AAGAGGAGCTGCTCCAGACTTAACCATCCTAACCGAGCTCAGATCATACTTCTCCG<br>TCTCCGGCGACTTCGCGATAGCTAAAACGATCGGTGGCACGACCATAGCCACCGT<br>GACTTTACACCTTTGTATCTGCTCTAACAAGAGAGTGATTTCGAACTTAGGCATTAT<br>CAAGATCGTGGCACCAACTCTGAGACTACAGAGCATGATGGAGTTGAGAGCGTAT<br>ATATGGAACATAGGCAAGACACAGAGGATCACGTCGTCTCTGTTGAAGTAAAGATT<br>CGGATTCTCGCCGTCGACTTGCTGCGCCACGCTCGTGACTAGACCTTTGTGTGTTA<br>GCATCACTCCTTTGGGGAGACCCGTCGTGCCGGATGAGAAAGGAAGCGCCACGA<br>CGTCTTCTGGCGAAATCTTCTCCGGTATTGAGTCCACTCGTGGTTCTTCGGACTGA<br>GTTAACTCGGAGAAACGGAGGCAGTTTTCGGGGATGGCGTCGGAGTCGGTGGTG<br>ACGATCAAAACGCCGTCGTTTTGGAGGTTCTTGATTTTATCGACGTAACGGGATTG<br>AGTGACGATGAGTTTCGCCGCGGAGGCTTTGGCTTGTTTAGAAATCTCCGCCGGA<br>GTGAAGAACGGGTTCGCGGAGGTGGTGATTGCGCCGATGAAGGAGGCGGCAAGG<br>AAAGTGAGGACTACTTCAGGAGAGTTCGGGAGGAGGATCATTACAACGTCGTGTT<br>GCTTCACGCCGAGGTTATGAAGACCGGCGGCGAGTTTCCGAGATGTTACGTGGAC<br>ATCGGCGTAGGTGTATACTTCGCCGGTGGGACCGTTGATCAAGCATGGCTTAGCG<br>GCGAACTCTGAGATATTTTCGAAGATGTAGTCGTGGAGTGGGAGGTGGTTAGGGA<br>TGTATATATCAGGCAATCTCGATCGGAAAATGACGTCATTACTACACTGTTTCTGAT<br>CATTCTGATCATTGACTATCACATCTTGTGTCGTCATTGTTTTATATTTGTTGTAAAA<br>AGTAGATAATTACTTCCTTGATGATCTGTAAAAAAGAGAAAAAGAAAGCATCTAAGA<br>ACTTGAAAAACTACGAATTAGAAAAGACCAAATATGTATTTCTTGCATTGACCAATTT<br>ATGCAAGTTTATATATATGTAAATGTAAGTTTCACGAGGTTCTACTAAACTAAACCAC<br>CCCCTTGGTTAGAAGAAAAGAGTGTGTGAGAACAGGCTGTTGTTGTCACACGATTC<br>GGACAATTCTGTTTGAAAGAGAGAGAGTAACAGTACGATCGAACGAACTTTGCTCT<br>GGAGATCACAGTGGGCATCATAGCATGTGGTACTAAACCCTTTCCCGCCATTCCAG<br>AACCTTCGATTGCTTGTTACAAAACCTGTGAGCCGTCGCTAGGACCTTGTTGTGTG<br>ACGAAATTGGAAGCTGCAATCAATAGGAAGACAGGAAGTCGAGCGTGTCTGGGTT<br>TTTTCAGTTTTGTTCTTTTTGCAAACAAATCACGAGCGACGGTAATTTCTTTCTCGAT<br>AAGAGGCCACGTGCTTTATGAGGGTAACATCAATTCAAGAAGGAGGGAAACACTTC<br>CTTTTTCTGGCCCTGATAATAGTATGAGGGTGAAGCCAAAATAAAGGATTCGCGCC<br>CAAATCGGCATCTTTAAATGCAGGTATGCGATAGTTCCTCACTCTTTCCTTACTCAC<br>GAGTAATTCTTGCAAATGCCTATTATGCAGATGTTATAATATCTGTGCGTAGTTTATC

	ATTATCAATACTGCCATTTCAAAGAATACGTAAATAATTAATAGTAGTGATTTTCCTA<br>ACTTTATTTAGTCAAAAAATTAGCCTTTTAATTCTGCTGTAACCCGTACATGCCCAAA<br>ATAGGGGGCGGGTTACACAGAATATATAACATCGTAGGTGTCTGGGTGAACAGTTT<br>ATTCCTGGCATCCACTAAATATAATGGAGCCCGCTTTTTAAGCTGGCATCCAGAAAA<br>AAAAAGAATCCCAGCACCAAAATATTGTTTTCTTCACCAACCATCAGTTCATAGGTC<br>CATTCTCTTAGCGCAACTACAGAGAACAGGGGCACAAACAGGCAAAAAACGGGCA<br>CAACCTCAATGGAGTGATGCAACCTGCCTGGAGTAAATGATGACACAAGGCAATTG<br>ACCCACGCATGTATCTATCTCATTTTCTTACACCTTCTATTACCTTCTGCTCTCTCTG<br>ATTTGGAAAAAGCTGAAAAAAAAGGTTGAAACCAGTTCCCTGAAATTATTCCCCTAC<br>TTGACTAATAAGTATATAAAGACGGTAGGTATTGATTGTAATTCTGTAAATCTATTTC<br>TTAAACTTCTTAAATTCTACTTTTATAGTTAGTCTTTTTTTTAGTTTTAAAACACCAAG<br>AACTTAGTTTCGAATAAACACACATAAACAAACAAAATGGTTACTGTTGAAGAAGTT<br>AGAAAAGCTCAAAGGGCAGAAGGTCCAGCCACAGTGATGGCTATTGGAACCGCAG<br>TTCCTCCAAATTGTGTAGATCAGGCCACTTATCCTGACTACTACTTTAGAATAACAA<br>ACTCTGAGCATAAGGCTGAATTGAAAGAAAAGTTCCAAAGGATGTGCGACAAATCA<br>CAGATCAAGAAAAGATACATGTACCTTAATGAGGAAGTCCTAAAGGAAAACCCAAA<br>TATGTGTGCATACATGGCCCCTTCCCTTGACGCTAGACAAGATATTGTGGTTGTAG<br>AGGTCCCAAAATTGGGCAAGGAAGCAGCTGTTAAAGCCATAAAGGAATGGGGTCA<br>ACCTAAGAGCAAAATCACCCACCTTGTGTTTTGCACTACAAGCGGAGTTGACATGC<br>CAGGCGCAGATTATCAGCTAACCAAACTTTTGGGTTTAAGGCCTTCTGTAAAAAGAT<br>TGATGATGTACCAACAAGGTTGTTTCGCTGGAGGCACTGTCTTAAGACTAGCCAAG<br>GATCTTGCAGAGAACAACAAAGGTGCTAGGGTGTTGGTTGTATGCTCAGAAATTAC<br>AGCCGTCACCTTTAGAGGACCAACTGACACTCACTTAGATTCCCTAGTTGGTCAGG<br>CATTGTTTGGCGACGGTGCTGCCGCAATAATCATTGGAAGTGATCCTATTCCAGAG<br>GTGGAAAAGCCTCTTTTTGAACTTGTTAGCGCTGCCCAAACTATATTGCCAGATTCT<br>GAGGGTGCAATCGACGGCCACTTAAGGGAAGTAGGTCTAACCTTCCATCTTTTGAA<br>AGATGTCCCTGGTTTAATTTCAAAGAACGTGGAAAAATCCCTAACAGAGGCTTTTAA<br>ACCATTGGGTATAAGTGACTGGAATAGCTTATTCTGGATCGCTCACCCAGGCGGCC<br>CTGCCATACTTGACCAGGTTGAAGCAAAATTGAGCTTAAAGCCAGAAAAACTAAGA<br>GCTACTAGACATGTATTGTCAGAGTATGGTAACATGTCCAGTGCCTGTGTCCTTTTC<br>ATTTTGGATGAAATGAGGAGAAAAAGCAAGGAGGACGGCCTAAAAACCACAGGTG<br>AGGGAATCGAATGGGGTGTTCTATTCGGCTTTGGTCCAGGCCTTACTGTGGAGAC<br>AGTTGTACTTCATTCAGTCGCAATTAATTAAGTGAATTTACTTTAAATCTTGCATTTA<br>AATAAATTTTCTTTTTATAGCTTTATGACTTAGTTTCAATTTATATACTATTTTAATGAC<br>ATTTTCGATTCATTGATTGAAAGCTTTGTGTTTTTTCTTGATGCGCTATTGCATTGTT<br>CTTGTCTTTTTCGCCACATGTAATATCTGTAGTAGATACCTGATACATTGTGGATGC<br>TGAGTGAAATTTTAGTTAATAATGGAGGCGCTCTTAATAATTTTGGGGATATTGGCT<br>TTTTTTTTTAAAGTTTACAAATGAATTTTTTCCGCCAGGATAACGATTCTGAAGTTAC<br>TCTTAGCGTTCCTATCGGTACAGCCATCAAATCATGCCTATAAATCATGCCTATATT<br>TGCGTGCAGTCAGTATCATCTACATGAAAAAAACTCCCGCAATTTCTTATAGAATAC<br>GTTGAAAATTAAATGTACGCGCCAAGATAAGATAACATATATCTAGATGCAGTAATA<br>TACACAGATTCCTATTGATCCGTACTAATATTGTTAGATGAGCTGCCGAAATTGTTC<br>GCATTGCCATCCTTCAGGAAGAATCCTAATTGTATGCCATTAATGAAAAGTGTGAAA<br>ATTAACAAAGAATCCAGAGTTGTTATATAGACTCTGTGAGACTCGCTTTGTACCGAT<br>ATTGGGCTCCTCAAGGTATTATGAAAATAGTCCACTGTGCAGCCCATGGTGATCAG<br>TGGCAATGCAAAATTAAGAACGTTGTAAAAGTATCCAATATGAAACTTGCTAGGCGC<br>AAAAACAGTCTCTGTGATAAACGATATCAGTGACGGTATTCCATATAACAATGTCGA<br>GCATACAATAACTATCAGTACCGCTTCGTGGTCGTGGAACTTGCATAGCAATGCGA<br>TAGCAATGGCACCTGTGGCAATTGAGAGAAGAGCACTTCCGAACTTGATCCCCAAA<br>AAACCAGCCTTATTTCCGCTCCTAACAGAATTATGGCACATAAGAGAGTATGATTCC<br>TTTCTTAGTATATATATACTGCTCAAGGGCAAATGCAATTACTTTGGAAATGCTTTCT<br>TGTTCTGATACGTAAGAAGACAAGTAATCTCTGGACTAAAAGTGGAGCCGGAATAG<br>AGACTCTCTTAGTATCAGTATCCACCTCTTCAAAGGATAATGCGTTCTTTGCCAATA<br>GTCACATATTGTGGGTATGTGCGTTTTGCAAAGGCAGCAATATATTGTTTACTTATT<br>TCCCAAAAAGGAAAAATCTTTAAACCATCCTAGAACTTCAATTCACCACGTTCAGGG<br>TAATATATTTTAACCGCCGACGTTCTCGCCATAACTTTTAAACAGTGATAACTAGTAT<br>TGAAACTTAGAAGAGAAAAGGGTAAAGTTAATGCTTAATCTTGTCTTGGCTTAAAAA<br>GTAATATGTACTTATTTACGTCTTCACAAAATCTAGCAGAAAAACGGTAGTATTTATG<br>TATATTCAAAAAAAAATCATTATCCTCATCAAGATTGCTTTATCTCGAGAGATCTGCG<br>GCCGCCGGGTGCATGTTGCTGGGCCTTATTGATAATCGGGTTTTTAGGTTTCAATT<br>TATGGGAAGATGGGACTACAAAGTCCATGCTGTTGGTCCGTGGAACGTCCATGCTA

	GGATTCATAGCCAATTTTTTAGCCTCTATTTTAACCTTCAAAGCATGGATCACCGAC CATAAAGTAGCAACAATGAACGCTTCAGGGATGATTGTCGTCGTTTACATAATAAAC GCCATTTTCTTATTCGTTTTCGTTATTTGTCAATTACTGGTATCCCTATTGGTAGTTC GAAACTTATGGGTCACAGGAGCTATCTTTTTGGGGCTATTTTTCTTTGTAGCAGGCC AGGTATTGGTTTATGCCTTCTCTACACAAATTTGTGAAGGGTTCAAGCACTACTTAG ATGGCCTCTTTTTTGGAAGCATCTGTAATGTGTTCACATTAATGATGGTTTACAAGA CTTGGGATATGACTACCGACGACGACTTGGAATTTGGTGTAAGTGTTAGCGGATCC CCGGGTACCGAGCTCGAATTCACTGGCCGTCGTTTTACAACGTCGTGACTGGGAA AACCCTGGCGTTACCCAACTTAATCGCCTTGCAGCACATCCCCCTTTCGCCAGCTG GCGTAATAGCGAAGAGGCCCGCACCGATCGCCCTTCCCAACAGTTGCGCAGCCTG AATGGCGAATGGCGCCTGATGCGGTATTTTCTCCTTACGCATCTGTGCGGTATTTC ACACCGCATATGGTGCACTCTCAGTACAATCTGCTCTGATGCCGCATAGTTAAGCC AGCCCCGACACCCGCCAACACCCGCTGACGCGCCCTGACGGGCTTGTCTGCTCC CGGCATCCGCTTACAGACAAGCTGTGACCGTCTCCGGGAGCTGCATGTGTCAGAG GTTTTCACCGTCATCACCGAAACGCGCGAGACGAAAGGGCCTCGTGATACGCCTA TTTTTATAGGTTAATGTCATGATAATAATGGTTTCTTAGACGTCAGGTGGCACTTTTC GGGGAAATGTGCGCGGAACCCCTATTTGTTTATTTTTCTAAATACATTCAAATATGT ATCCGCTCATGAGACAATAACCCTGATAAATGCTTCAATAATATTGAAAAAGGAAGA GTATGAGTATTCAACATTTCCGTGTCGCCCTTATTCCCTTTTTTGCGGCATTTTGCC TTCCTGTTTTTGCTCACCCAGAAACGCTGGTGAAAGTAAAAGATGCTGAAGATCAG TTGGGTGCACGAGTGGGTTACATCGAACTGGATCTCAACAGCGGTAAGATCCTTGA GAGTTTTCGCCCCGAAGAACGTTTTCCAATGATGAGCACTTTTAAAGTTCTGCTATG TGGCGCGGTATTATCCCGTATTGACGCCGGGCAAGAGCAACTCGGTCGCCGCATA CACTATTCTCAGAATGACTTGGTTGAGTACTCACCAGTCACAGAAAAGCATCTTACG GATGGCATGACAGTAAGAGAATTATGCAGTGCTGCCATAACCATGAGTGATAACAC TGCGGCCAACTTACTTCTGACAACGATCGGAGGACCGAAGGAGCTAACCGCTTTTT TGCACAACATGGGGGATCATGTAACTCGCCTTGATCGTTGGGAACCGGAGCTGAA TGAAGCCATACCAAACGACGAGCGTGACACCACGATGCCTGTAGCAATGGCAACA ACGTTGCGCAAACTATTAACTGGCGAACTACTTACTCTAGCTTCCCGGCAACAATTA ATAGACTGGATGGAGGCGGATAAAGTTGCAGGACCACTTCTGCGCTCGGCCCTTC CGGCTGGCTGGTTTATTGCTGATAAATCTGGAGCCGGTGAGCGTGGGTCTCGCGG TATCATTGCAGCACTGGGGCCAGATGGTAAGCCCTCCCGTATCGTAGTTATCTACA CGACGGGGAGTCAGGCAACTATGGATGAACGAAATAGACAGATCGCTGAGATAGG TGCCTCACTGATTAAGCATTGGTAACTGTCAGACCAAGTTTACTCATATATACTTTA GATTGATTTAAAACTTCATTTTTAATTTAAAAGGATCTAGGTGAAGATCCTTTTTGAT AATCTCATGACCAAAATCCCTTAACGTGAGTTTTCGTTCCACTGAGCGTCAGACCC CGTAGAAAAGATCAAAGGATCTTCTTGAGATCCTTTTTTTCTGCGCGTAATCTGCTG CTTGCAAACAAAAAAACCACCGCTACCAGCGGTGGTTTGTTTGCCGGATCAAGAGC TACCAACTCTTTTTCCGAAGGTAACTGGCTTCAGCAGAGCGCAGATACCAAATACT GTTCTTCTAGTGTAGCCGTAGTTAGGCCACCACTTCAAGAACTCTGTAGCACCGCC TACATACCTCGCTCTGCTAATCCTGTTACCAGTGGCTGCTGCCAGTGGCGATAAGT CGTGTCTTACCGGGTTGGACTCAAGACGATAGTTACCGGATAAGGCGCAGCGGTC GGGCTGAACGGGGGGTTCGTGCACACAGCCCAGCTTGGAGCGAACGACCTACAC CGAACT
pIV015	GAGATACCTACAGCGTGAGCTATGAGAAAGCGCCACGCTTCCCGAAGGGAGAAAG GCGGACAGGTATCCGGTAAGCGGCAGGGTCGGAACAGGAGAGCGCACGAGGGA GCTTCCAGGGGGAAACGCCTGGTATCTTTATAGTCCTGTCGGGTTTCGCCACCTCT GACTTGAGCGTCGATTTTTGTGATGCTCGTCAGGGGGGCGGAGCCTATGGAAAAA CGCCAGCAACGCGGCCTTTTTACGGTTCCTGGCCTTTTGCTGGCCTTTTGCTCACA TGTTCTTTCCTGCGTTATCCCCTGATTCTGTGGATAACCGTATTACCGCCTTTGAGT GAGCTGATACCGCTCGCCGCAGCCGAACGACCGAGCGCAGCGAGTCAGTGAGCG AGGAAGCGGAAGAGCGCCCAATACGCAAACCGCCTCTCCCCGCGCGTTGGCCGA TTCATTAATGCAGCTGGCACGACAGGTTTCCCGACTGGAAAGCGGGCAGTGAGCG CAACGCAATTAATGTGAGTTAGCTCACTCATTAGGCACCCCAGGCTTTACACTTTAT GCTTCCGGCTCGTATGTTGTGTGGAATTGTGAGCGGATAACAATTTCACACAGGAA ACAGCTATGACCATGATTACGCCAAGCTTGCATGCCTGCAGGTCGACTCTAGAGC GCTAATTACATTTGATGAAAGGTTATCTTTTAAAGCCGCTAACGATCAAGATATGGT GGATTGGATAATAAATTTCAAGAGTGGAATTTTGATAAGAAAAAAATTAAAGGCCGA AAATATATAAGCAATGCCAATGATGAATATGTATTGACTTTTTTTTTCTTTGTACTATT AAATAATACGTGTAATTGTTTTCCTTACGTCTAGTTAAAAAACTATTTATTTATTTGTA TTTTAATGATAATAAGCACTTTTTTCCTCGCTGAAAATTAAGGGAAAGGACCAAATA

	CAAAAGTGATTAGAAGGATAACGAGTAGAGAAAAAGGGAAGTAAAAGTTAAAAACT<br>AGAATCCTAGTATGACATGCATTGCCCAATTTGCCACCATAGAGCGCATGTAGTGT<br>ACTGTGCACATTGTATCAATACGAGCCCAAGTCTGCTACTGAAGCTAAAACTAGATT<br>TAATTTTATTAAAGGATGAGAATAAAGAACTTAACGGGAACAACTGTGGGAATACTC<br>AGGTATCGTAAGATGCAAGAGTTCGAATCTCTTAGCAACCATTATTTTTTTCCTCAA<br>CATAACGAGAACACACAGGGGCGCTATCGCACAGAATCAAATTCGATGACTGGAAA<br>TTTTTTGTTAATTTCAGAGGTCGCCTGACGCATATACCTTTTTCAACTGAAAAATTGG<br>GAGAAAAAGGAAAGGTGAGAGCGCCGGAACCGGCTTTTCATATAGAATAGAGAAG<br>CGTTCATGACTAAATGCTTGCATCACAATACTTGAAGTTGACAATATTATTTAAGGA<br>CCTATTGTTTTTTCCAATAGGTGGTTAGCAATCGTCTTACTTTCTAACTTTTCTTACC<br>TTTTACATTTCAGCAATATATATATATATATTTCAAGGATATACCATTCTAATGTCTGC<br>CCCTAAGAAGATCGTCGTTTTGCCAGGTGACCACGTTGGTCAAGAAATCACAGCCG<br>AAGCCATTAAGGTTCTTAAAGCTATTTCTGATGTTCGTTCCAATGTCAAGTTCGATTT<br>CGAAAATCATTTAATTGGTGGTGCTGCTATCGATGCTGCAGGTATTCCACTTCCAG<br>ATGAGGCGCTGGAAGCCTCCAAGAAGGCTGATGCCGTTTTGTTAGGTGCTGTGGG<br>TGGTCCTAAATGGGGTACCGGTAGTGTTAGACCTGAACAAGGTTTACTAAAAATCC<br>GTAAAGAACTTCAATTGTACGCCAACTTAAGACCATGTAACTTTGCATCCGACTCTC<br>TTTTAGACTTATCTCCAATCAAGCCACAATTTGCTAAAGGTACTGACTTCGTTGTTG<br>TCAGAGAATTAGTGGGAGGTATTTACTTTGGTAAGAGAAAGGAAGACGATGGTGAT<br>GGTGTCGCTTGGGATAGTGAACAATACACCGTTCCAGAAGTGCAAAGAATCACAAG<br>AATGGCCGCTTTCATGGCCCTACAACATGAGCCACCATTGCCTATTTGGTCCTTGG<br>ATAAAGCTAATGTTTTGGCCTCTTCAAGATTATGGAGAAAAACTGTGGAGGAAACCA<br>TCAAGAACGAATTCCCTACATTGAAGGTTCAACATCAATTGATTGATTCTGCCGCCA<br>TGATCCTAGTTAAGAACCCAACCCACCTAAATGGTATTATAATCACCAGCAACATGT<br>TTGGTGATATCATCTCCGATGAAGCCTCCGTTATCCCAGGTTCCTTGGGTTTGTTG<br>CCATCTGCGTCCTTGGCCTCTTTGCCAGACAAGAACACCGCATTTGGTTTGTACGA<br>ACCATGCCACGGTTCTGCTCCAGATTTGCCAAAGAATAAGGTCAACCCTATCGCCA<br>CTATCTTGTCTGCTGCAATGATGTTGAAATTGTCATTGAACTTGCCTGAAGAAGGTA<br>AGGCCATTGAAGATGCAGTTAAAAAGGTTTTGGATGCAGGTATCAGAACTGGTGAT<br>TTAGGTGGTTCCAACAGTACCACCGAAGTCGGTGATGCTGTCGCCGAAGAAGTTAA<br>GAAAATCCTTGCTTAAAAAGATTCTCTTTTTTTATGATATTTGTACATAAACTTTATAA<br>ATGAAATTCATAATAGAAACGACACGAAATTACAAAATGGAATATGTTCATAGGGTA<br>GACGAAACTATATACGCAATCTACATACATTTATCAAGAAGGAGAAAAAGGAGGAT<br>GTAAAGGAATACAGGTAAGCAAATTGATACTAATGGCTCAACGTGATAAGGAAAAA<br>GAATTGCACTTTAACATTAATATTGACAAGGAGGAGGGCACCACACAAAAAGTTAG<br>GTGTAACAGAAAATCATGAAACTATGATTCCTAATTTATATATTGGAGGATTTTCTCT<br>AAAAAAAAAAAAATACAACAAATAAAAAACACTCAATGACCTGACCATTTGATGGAG<br>TTTAAGTCAATACCTTCTTGAACCATTTCCCATAATGGTGAAAGTTCCCTCAAGAATT<br>TTACTCTGTCAGAAACGGCCTTAACGACGTAGTCGATATGGTGCACTCTCAGTACA<br>ATCTGCTCTGATGCCGCATAGTTAAGCCAGCCCCGACACCCGCCAACACCCGCTG<br>ACGCGCCCTGACGGGCTTGTCTGCTCCCGGCATCCCCATGGGCTAGCCTTAGTAA<br>AAATGTGCGCACCACAAGCCTACATGACTCCACGTCACATGAAACCACACCGTGG<br>GGCCTTGTTGCGCTAGGAATAGGATATGCGACGAAGACGCTTCTGCTTAGTAACCA<br>CACCACATTTTCAGGGGGTCGATCTGCTTGCTTCCTTTACTGTCACGAGCGGCCCA<br>TAATCGCGCTTTTTTTTTAAAAGGCGCGAGACAGCAAACAGGAAGCTCGGGTTTCA<br>ACCTTCGGAGTGGTCGCAGATCTGGAGACTGGATCTTTACAATACAGTAAGGCAAG<br>CCACCATCTGCTTCTTAGGTGCATGCGACGGTATCCACGTGCAGAACAACATAGTC<br>TGAAGAAGGGGGGGAGGAGCATGTTCATTCTCTGTAGCAGTAAGAGCTTGGTGAT<br>AATGACCAAAACTGGAGTCTCGAAATCATATAAATAGACAATATATTTTCACACAAT<br>GAGATTTGTAGTACAGTTCTATTCTCTCTCTTGCATAAATAAGAAATTCATCAAGAAC<br>TTGGTTTGATATTTCACCAACACACACAAAAAACAGTACTTCACTAAATTTACACACA<br>AAACAAAATGGACCAAATTGAAGCAATGCTATGCGGTGGTGGTGAAAAGACCAAGG<br>TGGCCGTAACGACAAAAACTCTTGCAGATCCTTTGAATTGGGGTCTGGCAGCTGAC<br>CAGATGAAAGGTAGCCATCTGGATGAAGTTAAGAAGATGGTTGAGGAATACAGAAG<br>ACCAGTCGTAAATCTAGGCGGCGAGACATTGACGATAGGACAGGTAGCTGCTATTT<br>CGACCGTTGGCGGTTCAGTGAAGGTAGAACTTGCAGAAACAAGTAGAGCCGGAGT<br>TAAGGCTTCATCAGATTGGGTCATGGAAAGTATGAACAAGGGCACAGATTCCTATG<br>GCGTTACCACAGGCTTTGGTGCTACCTCTCATAGAAGAACTAAAAATGGCACTGCT<br>TTGCAAACAGAACTGATCAGATTCCTTAACGCCGGTATTTTCGGTAATACAAAGGAA<br>ACTTGCCATACATTACCCCAATCGGCAACAAGAGCTGCTATGCTTGTTAGGGTGAA<br>CACTTTGTTGCAAGGTTACTCTGGAATAAGGTTTGAAATTCTTGAGGCCATCACTTC

ACTATTGAACCACAACATTTCTCCTTCGTTGCCCTTAAGAGGAACAATAACTGCCAG
CGGTGATTTGGTTCCCCTTTCATATATCGCAGGCTTATTAACGGGAAGACCTAATTC
AAAGGCCACTGGTCCAGACGGAGAATCCTTAACCGCTAAGGAAGCATTTGAGAAA
GCTGGTATTTCAACTGGTTTCTTTGATTTGCAACCCAAGGAAGGTTTAGCCCTGGT
GAATGGCACCGCTGTCGGCAGCGGTATGGCATCCATGGTGTTGTTTGAAGCTAAC
GTACAAGCAGTTTTGGCCGAAGTTTTGTCCGCAATTTTTGCCGAAGTCATGAGTGG
AAAACCTGAGTTTACTGATCACTTGACCCACAGGTTAAAACATCACCCAGGACAAAT
TGAAGCAGCAGCTATCATGGAGCACATTTTGGACGGCTCTAGCTACATGAAGTTAG
CCCAGAAGGTTCATGAAATGGACCCTTTGCAAAAACCCAAACAAGATAGATATGCT
TTAAGGACATCCCCACAATGGCTTGGCCCTCAAATTGAAGTAATTAGACAAGCTAC
AAAGTCTATAGAAAGAGAGATCAACTCTGTTAACGATAATCCACTTATTGATGTGTC
GAGGAATAAGGCAATACATGGAGGCAATTTCCAGGGTACACCCATAGGAGTCAGT
ATGGATAATACCAGGCTTGCCATAGCCGCAATTGGCAAATTAATGTTTGCCCAATTT
TCTGAATTGGTCAATGACTTCTACAATAACGGTTTGCCTTCGAATCTGACCGCATCT
TCTAACCCTAGTCTTGATTATGGTTTCAAAGGTGCTGAGATAGCAATGGCAAGCTAT
TGTTCAGAGCTGCAATATCTAGCCAACCCAGTAACCTCTCATGTACAATCAGCCGA
ACAACACAATCAGGATGTTAATTCTTTGGGCCTGATTTCATCAAGAAAAACAAGCGA
GGCCGTTGATATCCTTAAATTAATGTCCACAACATTTTTAGTGGGTATATGCCAGGC
CGTAGATTTGAGACACTTGGAAGAGAATTTGAGACAGACAGTGAAAAATACCGTAT
CACAGGTTGCAAAAAAGGTTCTAACTACAGGTATCAATGGTGAATTGCACCCATCA
AGATTCTGTGAAAAAGATTTATTAAAAGTTGTAGATAGAGAACAAGTATTTACTTACG
TTGACGATCCATGTAGCGCTACTTATCCATTGATGCAGAGATTGAGACAAGTTATTG
TAGATCACGCTTTATCCAATGGTGAAACTGAGAAAAATGCCGTTACTTCAATATTCC
AAAAGATAGGTGCCTTTGAAGAAGAACTGAAGGCAGTTTTACCAAAGGAAGTCGAA
GCTGCTAGAGCCGCATACGGAAATGGTACTGCCCCTATACCAAATAGAATCAAAGA
GTGTAGGTCGTACCCTTTGTACAGATTCGTTAGAGAAGAGTTGGGAACCAAATTAC
TAACTGGTGAAAAAGTCGTTAGCCCAGGTGAAGAATTTGACAAGGTATTCACAGCT
ATGTGCGAGGGAAAGTTGATAGATCCACTTATGGATTGCTTGAAAGAGTGGAATGG
TGCACCTATTCCAATCTGCTAAATAAAGCAATCTTGATGAGGATAATGATTTTTTTT
GAATATACATAAATACTACCGTTTTTCTGCTAGATTTTGTGAAGACGTAAATAAGTAC
ATATTACTTTTTAAGCCAAGACAAGATTAAGCATTAACTTTACCCTTTTCTCTTCTAA
GTTTCAATACTAGTTATCACTGTTTAAAAGTTATGGCGAGAACGTCGGCGGTTAAAA
TATATTACCCTGAACGTGGTGAATTGAAGTTCTAGGATGGTTTAAAGATTTTTCCTTT
TTGGGAAATAAGTAAACAATATATTGCTGCCTTTGCAAAACGCACATACCCACAATA
TGTGACTATTGGCAAAGAACGCATTATCCTTTGAAGAGGTGGATACTGATACTAAG
AGAGTCTCTATTCCGGCTCCACTTTTAGTCCAGAGATTACTTGTCTTCTTACGTATC
AGAACAAGAAAGCATTTCCAAAGTAATTGCATTTGCCCTTGAGCAGTATATATATAC
TAAGAAAGGAATCATACTCTCTTATGTGCCATAATTCTGTTAGGAGCGGAAATAAGG
CTGGTTTTTTGGGGATCAAGTTCGGAAGTGCTCTTCTCTCAATTGCCACAGGTGCC
ATTGCTATCGCATTGCTATGCAAGTTCCACGACCACGAAGCGGTACTGATAGTTAT
TGTATGCTCGACATTGTTATATGGAATACCGTCACTGATATCGTTTATCACAGAGAC
TGTTTTTGCGCCTAGCAAGTTTCATATTGGATACTTTTACAACGTTCTTAATTTTGCA
TTGCCACTGATCACCATGGGCTGCACAGTGGACTATTTTCATAATACCTTGAGGAG
CCCAATATCGGTACAAAGCGAGTCTCACAGAGTCTATATAACAACTCTGGATTCTTT
GTTAATTTTCACACTTTTCATTAATGGCATACAATTAGGATTCTTCCTGAAGGATGG
CAATGCGAACAATTTCGGCAGCTCATCTAACAATATTAGTACGGATCAATATCGAGA
GATCTGCGGCCGCATGGAGAAAAAGAAAGATCCCCTGATGAATAGTCTCATGAAGT
TAGATGTTTTACGAATGAAAAAGAATAATAACCTGATTAGGCACAGGATAGAACAGC
TAAATGAGCGGATTTATAGTAAAAGGAATCACATCAGCGAATTGAAAGTAGAAATTG
ACAATTATAAATGTTATAAGGTGGGTACTGGTACGGACAAATTAAGAGAGCAAGTG
GAAATTAGTGATGCAAAAAATAAGCTGGCACAAGTATCGAAGATTTGTGAATCGGC
TAGAGATTACAAGCTCAATCTACTAAATAATTGGTTTGTGATCCAGAAGCTGCAAGA
TAATTTCCAAATACCTTTCGCTATAGCTTTTCAGCCACTAATATCTTTGAAAAACTTC
CGTATTTTACCTCTTGCTATAACAAACGATTCCATCAACATCATGTGGAAGTATATTA
GCTTCTTCTCAGACATTCTTATGATTAAACTTCCCTACACAAATAAAATCTGTGAACA
GGATCCCCGGGTACCGAGCTCGAATTCACTGGCCGTCGTTTTACAACGTCGTGAC
TGGGAAAACCCTGGCGTTACCCAACTTAATCGCCTTGCAGCACATCCCCCTTTCGC
CAGCTGGCGTAATAGCGAAGAGGCCCGCACCGATCGCCCTTCCCAACAGTTGCGC
AGCCTGAATGGCGAATGGCGCCTGATGCGGTATTTTCTCCTTACGCATCTGTGCG
GTATTTCACACCGCATATGGTGCACTCTCAGTACAATCTGCTCTGATGCCGCATAG
TTAAGCCAGCCCCGACACCCGCCAACACCCGCTGACGCGCCCTGACGGGCTTGT

	CTGCTCCCGGCATCCGCTTACAGACAAGCTGTGACCGTCTCCGGGAGCTGCATGT<br>GTCAGAGGTTTTCACCGTCATCACCGAAACGCGCGAGACGAAAGGGCCTCGTGAT<br>ACGCCTATTTTTATAGGTTAATGTCATGATAATAATGGTTTCTTAGACGTCAGGTGG<br>CACTTTTCGGGGAAATGTGCGCGGAACCCCTATTTGTTTATTTTTCTAAATACATTC<br>AAATATGTATCCGCTCATGAGACAATAACCCTGATAAATGCTTCAATAATATTGAAA<br>AAGGAAGAGTATGAGTATTCAACATTTCCGTGTCGCCCTTATTCCCTTTTTTGCGGC<br>ATTTTGCCTTCCTGTTTTTGCTCACCCAGAAACGCTGGTGAAAGTAAAAGATGCTGA<br>AGATCAGTTGGGTGCACGAGTGGGTTACATCGAACTGGATCTCAACAGCGGTAAG<br>ATCCTTGAGAGTTTTCGCCCCGAAGAACGTTTTCCAATGATGAGCACTTTTAAAGTT<br>CTGCTATGTGGCGCGGTATTATCCCGTATTGACGCCGGGCAAGAGCAACTCGGTC<br>GCCGCATACACTATTCTCAGAATGACTTGGTTGAGTACTCACCAGTCACAGAAAAG<br>CATCTTACGGATGGCATGACAGTAAGAGAATTATGCAGTGCTGCCATAACCATGAG<br>TGATAACACTGCGGCCAACTTACTTCTGACAACGATCGGAGGACCGAAGGAGCTA<br>ACCGCTTTTTTGCACAACATGGGGGATCATGTAACTCGCCTTGATCGTTGGGAACC<br>GGAGCTGAATGAAGCCATACCAAACGACGAGCGTGACACCACGATGCCTGTAGCA<br>ATGGCAACAACGTTGCGCAAACTATTAACTGGCGAACTACTTACTCTAGCTTCCCG<br>GCAACAATTAATAGACTGGATGGAGGCGGATAAAGTTGCAGGACCACTTCTGCGCT<br>CGGCCCTTCCGGCTGGCTGGTTTATTGCTGATAAATCTGGAGCCGGTGAGCGTGG<br>GTCTCGCGGTATCATTGCAGCACTGGGGCCAGATGGTAAGCCCTCCCGTATCGTA<br>GTTATCTACACGACGGGGAGTCAGGCAACTATGGATGAACGAAATAGACAGATCG<br>CTGAGATAGGTGCCTCACTGATTAAGCATTGGTAACTGTCAGACCAAGTTTACTCAT<br>ATATACTTTAGATTGATTTAAAACTTCATTTTTAATTTAAAAGGATCTAGGTGAAGAT<br>CCTTTTTGATAATCTCATGACCAAAATCCCTTAACGTGAGTTTTCGTTCCACTGAGC<br>GTCAGACCCCGTAGAAAAGATCAAAGGATCTTCTTGAGATCCTTTTTTTCTGCGCGT<br>AATCTGCTGCTTGCAAACAAAAAAACCACCGCTACCAGCGGTGGTTTGTTTGCCGG<br>ATCAAGAGCTACCAACTCTTTTTCCGAAGGTAACTGGCTTCAGCAGAGCGCAGATA<br>CCAAATACTGTTCTTCTAGTGTAGCCGTAGTTAGGCCACCACTTCAAGAACTCTGTA<br>GCACCGCCTACATACCTCGCTCTGCTAATCCTGTTACCAGTGGCTGCTGCCAGTG<br>GCGATAAGTCGTGTCTTACCGGGTTGGACTCAAGACGATAGTTACCGGATAAGGC<br>GCAGCGGTCGGGCTGAACGGGGGGTTCGTGCACACAGCCCAGCTTGGAGCGAAC<br>GACCTACACCGAACT
pIV016	GGTAGCAACAGCAGTGTCACCTTCTTCTCTAAGGATAGTATCTAATTCAGGCCACA<br>AGGCTTCTCTCCAAGCAGTAAAGTCATCTTCAATACATTGATCATCGTCACCCAAGC<br>CCACCTGCACTAACCTTTGAGCACCTTGTTCAACAAGTATATCATCTACAACCTTAG<br>CGACCTTATTAAAATGCTCGTACTGTCTGTTACCAAGGCCGAACACACCATATTTCA<br>AATTCTTCAGCCATTCTCCTCTGTCATTACCTTCAGTAAACCACTTGTAAAACCTTG<br>CGGCATTATCTGTTGGTTCACCGTCGCCGTAGGTGGCAAGGAAGAAAAATGCGAC<br>ATCTTCTTTCTTCAATTTTTCTTCGTATTCGTCATCATCGGCGGCATAGTCATCCAGA<br>TCGACAATTTTGAATCTCGTCTTTTCATATCTAGCTTTTGCTTCTTCTCCTAGAGCCT<br>TAGCAAAACCTTCAGCTGTACCTGTTTGGGTGCCGAAGAATATAGTGACTTTCTTCC<br>TTCCATCGTCAATTTCCTCTTCTCTAGGTTTGATAACTAATGGCTTTAGTGGTTCAAC<br>CCTCTTTGAATTGCCCGAACCGCTCCTCCTACCAGCTTTTGCTGCAGCTTCTTTAG<br>CTGCAGCTTCTTTTGCTGCAGCTTCAGCGCTACAATTTCTGGGTTTCATGACTATAA<br>TGGAATGATTCAAAATATGCAATGAGAATTGTCCTCCTTTCTCTGAAGTATCGACTT<br>TCGATTGTCCGGGTGGTGGTAGCAACTCAAAATTTTGAACCATCCTACCAATTGTG<br>ATACCCAATATTGGTAAGGCTAGTATTATTCCTGGACACGATCTTCTGCCAACGCCA<br>AAAGGTACATATCTGAAGTCATTTCCGTTGGCTTCAACATGAGATTCCTCTTCGAAG<br>AATCTTTCAGGTCTGAATTCTTCAGGCTTTTTCCAACTGTTTGGATTGTTGGCCAAC<br>CACCATGCATTTACTAAAATCTTACTTTCTGCTGGAATATCATAACCGGCCAGTTTA<br>GCATCATGTAGGTTCATATGAGGAACTAGAAGAGGAATAGCCATTCTCAATCTCAA<br>GGTTTCTTTAACCACAGCTTGCAAGTAGGGTAACTTATGCAAATCAGGTTCTGTAAC<br>TTGGACACCTGGACCTAAAACGGTATCTAGCTCGTTTCTCAGTTTAGATTGTATTTC<br>TGGGTGATTCACCAATTCAGCAATACCCCATTCTATTGACCATAAGGTTGTTTCTAT<br>TGCAGCCACATTGATATTTTCTACAATGTACAAAACATTATCTTCGTTAATTTCACCT<br>TTTTGTTCAGCTTCTAAAATATGATCAATTGCACATTTTAAACCTTCCGAACCTGTGG<br>GCTTGGAAGAAGCTATTTGCTTTCTCTCGTCCACAAAGTACTTCTTGAAAAGAGCGA<br>TCCTCCTATCCTTGACGTCTTGGCAAATTTTCAAATAGCCTCTCAAAAATGGTCTTA<br>AGATTGGAATAAAGTCACCGTAATTATATTCGAAACTTTGAGCCAATCTCGATCTTT<br>CACCATTCAAAGCCTTAAGTCTCAGGAACAAAGGGTCATCTTCACTTTCAAATCTTC<br>TGTCAAACATAATTCTGAACATATTGTTGTACATCATCAATTGTAGTCTTTTTCTTAG<br>TACTATACCCTTTGTGGCCGAATCTGGATTTTTCTTTACGTCTTCCACTACGGAAGC

	TGCCTCAAACTCCCAACCTTCTCTATTTTGTTGAACAACCTTGTTAGTAAAGAATGG<br>TACGGTCATGATTCTTCTCATTTTTCTCCAGTGCTCACCGTAAACTGTAAAAACCAT<br>ATCTTGACCTTTACCAGTGAAAATATCAAACACAACATTTCTAGTTCTGGAGCCAAA<br>CTCAACGCCTTGCGTTAATAAGACCTCCTTAGTCAAATCAGGTGAGCTAACCACTA<br>CCAAATTCCTTTGACCCATTCTAAGCAAGAAAAGGTCACCGAACTTCTTAGCATAGT<br>CTACCAAATTTCTGTGATTCAAATCATCGCCCACTTGCAACCAATTACCAAAAATTG<br>GTATGGGGATGGGACCAGGAGGTAGTTTTAGTTTCTTACCTCTTAATTTAGAAATAA<br>CCGTGGCTAGAATAACTGCCACAAATACAGCAATAAGTGACTTTTCCAGCAATAAC<br>AAATCCATTTTGAATATGTATTACTTGGTTATGGTTATATATGACAAAAGAAAAAGAA<br>GAACAGAAGAATAACGCAAGGAAGAACAATAACTGAAATTGATAGAGAAGTATTAT<br>GTCTTTGTCTTTTTATAATAAATCAAGTGCAGAAATCCGTTAGACAACATGAGGGAT<br>AAAATTTAACGTGGGCGAAGAAGAAGGAAAAAAGTTTTTGTGAGGGCGTAATTGAA<br>GCGATCTGTTGATTGTAGATTTTTTTTTTTTGAGGAGTCAAAGTCAGAAGAGAACAG<br>ACAAATGGTATTAACCATCCAATACTTTTTTGGAGCAACGCTAAGCTCATGCTTTTC<br>CATTGGTTACGTGCTCAGTTGTTAGATATGGAAAGAGAGGATGCTCACGGCAGCGT<br>GACTCCAATTGAGCCCGAAAGAGAGGATGCCACGTTTTCCCGACGGCTGCTAGAA<br>TGGAAAAAGGAAAAATAGAAGAATCCCATTCCTATCATTATTTACGTAATGACCCAC<br>ACATTTTTGAGATTTTCAACTATTACGTATTACGATAATCCTGCTGTCATTATCATTA<br>TTATCTATATCGACGTATGCAACGTATGTGAAGCCAAGTAGGCAATTATTTAGTACT<br>GTCAGTATTGTTATGTAACAAAAATCACGATCTGGGTGGGTGTGGGTGTATTGGAT<br>TATAGGAAGCCACGCGCTCAACCTGGAATTACAGGAAGCTGGTAATTTTTTGGGTT<br>TGCAATCATCACCATCTGCACGTTGTTATAATGTCCCGTGTCTATATATATCCATTG<br>ACGGTATTCTATTTTTTTGCTATTGAAATGAGCGTTTTTTGTTACTACAATTGGTTTT<br>ACAGACGGAATTTTCCCTATTTGTTTCGTCCCATTTTTCCTTTTCTCATTGTTCTCAT<br>ATCTTAAAAAGGTCCTTTCTTCATAATCAATGCTTTCTTTTACTTAATATTTTACTTGC<br>ATTCAGTGAATTTTAATACATATTCCTCTAGTCTTGCAAAATCGATTTAGAATCAAGA<br>TACCAGCCTAAAAATGTCTCCACCAGTTTCTGTTACAAAAATGCAAGTCGAAAATTA<br>TGCTTTTGCACCAACAGTGAACCCTGCCGGTTCCACCAATACTTTGTTCTTAGCTG<br>GAGCAGGCCATAGAGGTCTAGAGATTGAAGGAAAGTTTGTGAAATTCACAGCCATA<br>GGCGTATACCTTGAGGAAAGTGCTATCCCATTTTTGGCAGAAAAGTGGAAAGGTAA<br>GACCCCTCAGGAGTTAACTGATAGCGTCGAGTTCTTTAGGGACGTGGTTACAGGTC<br>CATTCGAAAAGTTTACCAGAGTAACTATGATTCTACCTCTTACAGGAAAGCAATATT<br>CTGAGAAAGTCGCCGAAAACTGTGTTGCTCACTGGAAGGGCATAGGTACCTACACT<br>GATGACGAAGGAAGGGCAATCGAGAAATTCTTGGATGTGTTTAGATCAGAAACATT<br>CCCACCTGGTGCTTCCATTATGTTTACTCAGAGTCCATTAGGCTTGTTAACCATCAG<br>CTTTGCCAAGGACGATTCAGTTACCGGTACTGCAAATGCTGTAATCGAGAACAAAC<br>AACTATCAGAAGCCGTCCTTGAATCCATTATTGGAAAGCATGGTGTGAGTCCTGCA<br>GCCAAATGCTCTGTTGCCGAGAGAGTAGCAGAATTGTTAAAAAGAGCTATGCTGA<br>AGAGGCCTCAGTGTTCGGCAAACCAGAAACCGAAAAGTCCACAATACCTGTTATCG<br>GTGTGTAGACAAATCGCTCTTAAATATATACCTAAAGAACATTAAAGCTATATTATAA<br>GCAAAGATACGTAAATTTTGCTTATATTATTATACACATATCATATTTCTATATTTTTA<br>AGATTTGGTTATATAATGTACGTAATGCAAAGGAAATAAATTTTATACATTATTGAAC<br>AGCGTCCAAGTAACTACATTATGTGCACTAATAGTTTAGCGTCGTGAAGACTTTATT<br>GTGTCGCGAAAAGTAAAAATTTTAAAAATTAGAGCACCTTGAACTTGCGAAAAAGGT<br>TCTCATCAACTGTTTAAAAGGAGGATATCAGGTCCTATTTCTGACAAACAATATACA<br>AATTTAGTTTCAAAGATGAATCAGTGCGCGAAGGACATAACTCATGAAGCCTCCAG<br>TATACCCATCGATTTGCAAGAAAGATACTCGCACTGGAAGAAAAACACTAAACTAC<br>GGCTAGCCCATGGATTAACGTTTACAATTTCCTGATGCGGTATTTTCTCCTTACGCA<br>TCTGTGCGGTATTTCACACCGCATAGATCGGCAAGTGCACAAACAATACTTAAATAA<br>ATACTACTCAGTAATAACCTATTTCTTAGCATTTTTGACGAAATTTGCTATTTTGTTA<br>GAGTCTTTTACACCATTTGTCTCCACACCTCCGCTTACATCAACACCAATAACGCCA<br>TTTAATCTAAGCGCATCACCAACATTTTCTGGCGTCAGTCCACCAGCTAACATAAAA<br>TGTAAGCTTTCGGGGCTCTCTTGCCTTCCAACCCAGTCAGAAATCGAGTTCCAATC<br>CAAAAGTTCACCTGTCCCACCTGCTTCTGAATCAAACAAGGGAATAAACGAATGAG<br>GTTTCTGTGAAGCTGCACTGAGTAGTATGTTGCAGTCTTTTGGAAATACGAGTCTTT<br>TAATAACTGGCAAACCGAGGAACTCTTGGTATTCTTGCCACGACTCATCTCCATGC<br>AGTTGGACGATATCAATGCCGTAATCATTGACCAGAGCCAAAACATCCTCCTTAGG<br>TTGATTACGAAACACGCCAACCAAGTATTTCGGAGTGCCTGAACTATTTTTATATGC<br>TTTTACAAGACTTGAAATTTTCCTTGCAATAACCGGGTCAATTGTTCTCTTTCTATTG<br>GGCACACATATAATACCCAGCAAGTCAGCATCGGAATCTAGAGCACATTCTGCGGC<br>CTCTGTGCTCTGCAAGCCGCAAACTTTCACCAATGGACCAGAACTACCTGTGAAAT

	TAATAACAGACATACTCCAAGCTGCCTTTGTGTGCTTAATCACGTATACTCACGTGC<br>TCAATAGTCACCAATGCCCTCCCTCTTGGCCCTCTCCTTTTCTTTTTTCGACCGAAT<br>TAATTCTTAATCGGCAAAAAAAGAAAAGCTCCGGATCAAGATTGTACGTAAGGTGA<br>CAAGCTATTTTTCAATAAAGAATATCTTCCACTACTGCCATCTGGCGTCATAACTGC<br>AAAGTACACATATATTACGATGCTGTTCTATTAAATGCTTCCTATATTATATATATAG<br>TAATGTCGTTGATCTATGGTGCACTCTCAGTACAATCTGCTCTGATGCCGCATAGTT<br>AAGCGGTGCCACCTGGGTCCTTTTCATCACGTGCTATAAAAATAATTATAATTTAAA<br>TTTTTTAATATAAATATATAAATTAAAAATAGAAAGTAAAAAAAGAAATTAAAGAAAAA<br>ATAGTTTTTGTTTTCCGAAGATGTAAAAGACTCTAGGGGGATCGCCAACAAATACTA<br>CCTTTTATCTTGCTCTTCCTGCTCTCAGGTATTAATGCCGAATTGTTTCATCTTGTCT<br>GTGTAGAAGACCACACACGAAAATCCTGTGATTTTACATTTTACTTATCGTTAATCG<br>AATGTATATCTATTTAATCTGCTTTTCTTGTCTAATAAATATATATGTAAAGTACGCTT<br>TTTGTTGAAATTTTTTAAACCTTTGTTTATTTTTTTTTCTTCATTCCGTAACTCTTCTAC<br>CTTCTTTATTTACTTTCTAAAATCCAAATACAAAACATAAAAATAAATAAACACAGAG<br>TAAATTCCCAAATTATTCCATCATTAAAAGATACGAGGCGCGTGTAAGTTACAGGCA<br>AGCGATCTCTAGAGTCGACCTGCAGGCATGCAAGCTTGGCGTAATCATGGTCATA<br>GCTGTTTCCTGTGTGAAATTGTTATCCGCTCACAATTCCACACAACATACGAGCCG<br>GAAGCATAAAGTGTAAAGCCTGGGGTGCCTAATGAGTGAGCTAACTCACATTAATT<br>GCGTTGCGCTCACTGCCCGCTTTCCAGTCGGGAAACCTGTCGTGCCAGCTGCATT<br>AATGAATCGGCCAACGCGCGGGGAGAGGCGGTTTGCGTATTGGGCGCTCTTCCG<br>CTTCCTCGCTCACTGACTCGCTGCGCTCGGTCGTTCGGCTGCGGCGAGCGGTATC<br>AGCTCACTCAAAGGCGGTAATACGGTTATCCACAGAATCAGGGGATAACGCAGGA<br>AAGAACATGTGAGCAAAAGGCCAGCAAAAGGCCAGGAACCGTAAAAAGGCCGCGT<br>TGCTGGCGTTTTTCCATAGGCTCCGCCCCCCTGACGAGCATCACAAAAATCGACG<br>CTCAAGTCAGAGGTGGCGAAACCCGACAGGACTATAAAGATACCAGGCGTTTCCC<br>CCTGGAAGCTCCCTCGTGCGCTCTCCTGTTCCGACCCTGCCGCTTACCGGATACC<br>TGTCCGCCTTTCTCCCTTCGGGAAGCGTGGCGCTTTCTCATAGCTCACGCTGTAGG<br>TATCTCAGTTCGGTGTAGGTCGTTCGCTCCAAGCTGGGCTGTGTGCACGAACCCC<br>CCGTTCAGCCCGACCGCTGCGCCTTATCCGGTAACTATCGTCTTGAGTCCAACCC<br>GGTAAGACACGACTTATCGCCACTGGCAGCAGCCACTGGTAACAGGATTAGCAGA<br>GCGAGGTATGTAGGCGGTGCTACAGAGTTCTTGAAGTGGTGGCCTAACTACGGCT<br>ACACTAGAAGAACAGTATTTGGTATCTGCGCTCTGCTGAAGCCAGTTACCTTCGGA<br>AAAAGAGTTGGTAGCTCTTGATCCGGCAAACAAACCACCGCTGGTAGCGGTGGTTT<br>TTTTGTTTGCAAGCAGCAGATTACGCGCAGAAAAAAAGGATCTCAAGAAGATCCTTT<br>GATCTTTTCTACGGGGTCTGACGCTCAGTGGAACGAAAACTCACGTTAAGGGATTT<br>TGGTCATGAGATTATCAAAAAGGATCTTCACCTAGATCCTTTTAAATTAAAAATGAA<br>GTTTTAAATCAATCTAAAGTATATATGAGTAAACTTGGTCTGACAGTTACCAATGCTT<br>AATCAGTGAGGCACCTATCTCAGCGATCTGTCTATTTCGTTCATCCATAGTTGCCTG<br>ACTCCCCGTCGTGTAGATAACTACGATACGGGAGGGCTTACCATCTGGCCCCAGT<br>GCTGCAATGATACCGCGAGACCCACGCTCACCGGCTCCAGATTTATCAGCAATAAA<br>CCAGCCAGCCGGAAGGGCCGAGCGCAGAAGTGGTCCTGCAACTTTATCCGCCTC<br>CATCCAGTCTATTAATTGTTGCCGGGAAGCTAGAGTAAGTAGTTCGCCAGTTAATA<br>GTTTGCGCAACGTTGTTGCCATTGCTACAGGCATCGTGGTGTCACGCTCGTCGTTT<br>GGTATGGCTTCATTCAGCTCCGGTTCCCAACGATCAAGGCGAGTTACATGATCCCC<br>CATGTTGTGCAAAAAAGCGGTTAGCTCCTTCGGTCCTCCGATCGTTGTCAGAAGTA<br>AGTTGGCCGCAGTGTTATCACTCATGGTTATGGCAGCACTGCATAATTCTCTTACT<br>GTCATGCCATCCGTAAGATGCTTTTCTGTGACTGGTGAGTACTCAACCAAGTCATT<br>CTGAGAATAGTGTATGCGGCGACCGAGTTGCTCTTGCCCGGCGTCAATACGGGAT<br>AATACCGCGCCACATAGCAGAACTTTAAAAGTGCTCATCATTGGAAAACGTTCTTC<br>GGGGCGAAAACTCTCAAGGATCTTACCGCTGTTGAGATCCAGTTCGATGTAACCCA<br>CTCGTGCACCCAACTGATCTTCAGCATCTTTTACTTTCACCAGCGTTTCTGGGTGA<br>GCAAAAACAGGAAGGCAAAATGCCGCAAAAAAGGGAATAAGGGCGACACGGAAAT<br>GTTGAATACTCATACTCTTCCTTTTTCAATATTATTGAAGCATTTATCAGGGTTATTG<br>TCTCATGAGCGGATACATATTTGAATGTATTTAGAAAAATAAACAAATAGGGGTTCC<br>GCGCACATTTCCCCGAAAAGTGCCACCTGACGTCTAAGAAACCATTATTATCATGA<br>CATTAACCTATAAAAATAGGCGTATCACGAGGCCCTTTCGTCTCGCGCGTTTCGGT<br>GATGACGGTGAAAACCTCTGACACATGCAGCTCCCGGAGACGGTCACAGCTTGTC<br>TGTAAGCGGATGCCGGGAGCAGACAAGCCCGTCAGGGCGCGTCAGCGGGTGTTG<br>GCGGGTGTCGGGGCTGGCTTAACTATGCGGCATCAGAGCAGATTGTACTGAGAGT<br>GCACCATATGCGGTGTGAAATACCGCACAGATGCGTAAGGAGAAAATACCGCATC<br>AGGCGCCATTCGCCATTCAGGCTGCGCAACTGTTGGGAAGGGCGATCGGTGCGG

	GCCTCTTCGCTATTACGCCAGCTGGCGAAAGGGGGATGTGCTGCAAGGCGATTAA<br>GTTGGGTAACGCCAGGGTTTTCCCAGTCACGACGTTGTAAAACGACGGCCAGTGA<br>ATTCGAGCTCGGTACCCGGGGATCCTGTCCAAGATGGCAAACTTCAATTCAATGTC<br>TAAGGGCTGGTCATCAGGTTCAAAAACCATATCCTTTACGGGACCTTCAGCTTCATT<br>TTCGAATTCTGTTTCAAATTCTAATCTGCCCGGCATAAACCCCTGTACTTCATGGCA<br>GCTAGGAACCGATGCCATGGGCTTTCTTGGAGGCTCTAGCGGCCTCTCCCGGAAG<br>GACTCGATTCTATGCCTTCGCTGTTCCAAAAATTCATCTTGTGGGACATGTATATTT<br>TGGGTAATATCAGGTATTGGATAGTATTTGCTTTCCAGATAATATTTTAGGTAATGTT<br>CCTTAACTTCTTCTTTGCCTCTGCTGCCTATATGGTCAGCAATATCCTGCCAATTAC<br>CGAGCCCTAAAGTCTGTGCGCCTTTAATCAACTGCAATTCTTCATCGGCACCCCAA<br>TCAGGACAGAGAATGGGGTATGAATTGGTCTCTATTATCCTATAATCATGGTAAGG<br>GCGATGCTTTCCTGTAGCGGCCGCAGATCTCTCGAGCTATCAAAAACGATAGATCG<br>ATTAGGATGACTTTGAAATGACTCCAAAGTGGACTGGCCGTTAATTTCAAGCGTGA<br>GTAAAATAGTGCATGACAAAAGATGAGCTAGGCTTTTGTAAAAATATCTTACGTTGT<br>AAAATTTTAGAAATCATTATTTCCTTCATATCATTTTGTCATTGACCTTCAGAAGAAA<br>AGAGCCGACCAATAATATAAATAAATAAATAAAAATAATATTCCATTATTTCTAAACA<br>GATTCAATACTCATTAAAAAACTATATCAATTAATTTGAATTAACCACGCGATTTACC<br>ATACATCTCTCAGATATCTACCGCTTGTCTGAAGATTCTTTACAAAGCCCTCTGCTT<br>TTGTTGAATCCATGCTACCTTGTTCTTGTGCGATTGTATGCAGTGATCTATGAACAT<br>CTCTAGCCATTCCTTTAGCGTCACCACAAACATACAAATAAGCACCTTGGGAAATCA<br>TATTCCAGATATCAGAAGCCTTGTCCATCATCTTGTGTTGAACGTATTCTTTGGTTG<br>GACCTTCCCTGCTGAATGCAACACTCAATTCGGCCAATGCACCACTTTCAACAAAT<br>CTCTGCAATTCTTCTTCGTAGATAAAGTCCATTCTTCTGTTTCTACAACCAAAGAATA<br>AAACTGAAGGACCCAATTCAACTCCACTCTCCACTAAAGCCAATCTTTCTTGCAAGA<br>AGCCTCTGAATGGGGCCAAACCAGTACCTGGACCGATCATAATAATGGGTACTTTA<br>GAATCGCTTGGTAACTTGAAATTAGATTGTCTTACGAAAATTGGTGCACTAGAACAA<br>TTTTCGGATTTTTCATAGGGTACAGCATTTTTCATCCAGGTAGAACAGACACCCTTG<br>TGAATCCTACCTGTTGGCATCTTTTCGTAGACCAGAGCACATGTCACGTGAATTCT<br>GGTTTCGGCAATTTTTGGAGATGAGGATATGGAATAAAATCTAGGCTGCAATCTTG<br>GGGCCACACCAGCGAAAAATACACCCAGAGGAGGTTTAGCACTTGGAAACTCAGC<br>CATCACTTCTAAAAGCGATCTTTGGCTCTCCACTACCCACTTACTGTATTCATCTTTA<br>CCGGCTGGTGAAGCCAAATGCTTTAATCTCTCAGCTTCGGTAGGATCGGATGCGT<br>GGGCTGCCAATGCAACCAAAGCACTTTTCTTTGGAGAAGACAGTAAACAAGCATAT<br>CTAGTTAGGGCTGTTCTTAAGTTACAGGGAGGGAATGGAGGGGGAAGCGAGCTTG<br>ATATTGGAGTTCCGTCTTCTTTTTCTGCATGCAATGAGAAGTAAGTATCTGGTGACA<br>TATCCAATAACCTCAAAGCTTCATCAACAGTTTCTGACAAATTATCGCAAAGTACTC<br>CGACATGGTCACCTGTTTCGTAAGTCAAACCACTACCAGCGATATCAAATTCCAAAT<br>GTATACATGACCTGTCACTTTCTGGGGTGTGAAGTTCCCTTTTAACAGCAACATTAG<br>CTTTGTATGGATGTTGTGCGTCAAAAACGGTGTAACCGTTACCGTTGGCCATGTTA<br>ATATCATTGAATTTGGCGTCTTCTGAATCATGGATTGAGACTCTATATTCTAGGACT<br>GCTGCAGTGTAGGG
pIV017	AGAATCACAAGAATGGCCGCTTTCATGGCCCTACAACATGAGCCACCATTGCCTAT<br>TTGGTCCTTGGATAAAGCTAATGTTTTGGCCTCTTCAAGATTATGGAGAAAAACTGT<br>GGAGGAAACCATCAAGAACGAATTCCCTACATTGAAGGTTCAACATCAATTGATTG<br>ATTCTGCCGCCATGATCCTAGTTAAGAACCCAACCCACCTAAATGGTATTATAATCA<br>CCAGCAACATGTTTGGTGATATCATCTCCGATGAAGCCTCCGTTATCCCAGGTTCC<br>TTGGGTTTGTTGCCATCTGCGTCCTTGGCCTCTTTGCCAGACAAGAACACCGCATT<br>TGGTTTGTACGAACCATGCCACGGTTCTGCTCCAGATTTGCCAAAGAATAAGGTCA<br>ACCCTATCGCCACTATCTTGTCTGCTGCAATGATGTTGAAATTGTCATTGAACTTGC<br>CTGAAGAAGGTAAGGCCATTGAAGATGCAGTTAAAAAGGTTTTGGATGCAGGTATC<br>AGAACTGGTGATTTAGGTGGTTCCAACAGTACCACCGAAGTCGGTGATGCTGTCG<br>CCGAAGAAGTTAAGAAAATCCTTGCTTAAAAAGATTCTCTTTTTTTATGATATTTGTA<br>CATAAACTTTATAAATGAAATTCATAATAGAAACGACACGAAATTACAAAATGGAATA<br>TGTTCATAGGGTAGACGAAACTATATACGCAATCTACATACATTTATCAAGAAGGAG<br>AAAAAGGAGGATGTAAAGGAATACAGGTAAGCAAATTGATACTAATGGCTCAACGT<br>GATAAGGAAAAAGAATTGCACTTTAACATTAATATTGACAAGGAGGAGGGCACCAC<br>ACAAAAAGTTAGGTGTAACAGAAAATCATGAAACTATGATTCCTAATTTATATATTGG<br>AGGATTTTCTCTAAAAAAAAAAAAAATACAACAAATAAAAAACACTCAATGACCTGAC<br>CATTTGATGGAGTTTAAGTCAATACCTTCTTGAACCATTTCCCATAATGGTGAAAGT<br>TCCCTCAAGAATTTTACTCTGTCAGAAACGGCCTTAACGACGTAGTCGATATGGTG<br>CACTCTCAGTACAATCTGCTCTGATGCCGCATAGTTAAGCCAGCCCCGACACCCGC

	CAACACCCGCTGACGCGCCCTGACGGGCTTGTCTGCTCCCGGCATCCCCATGGG<br>CTAGCCAGGCATTAAAAGAGGAGCGAATTTTTTTTAATAAAAATCTTAATAATCATT<br>AAAAGATAAATAATAGTCTATATATACGTATATAAATAAAAAATATTCAAAAAATAAAA<br>TAAACTATTATTTTAGCGTAAAGGATGGGGAAAGAGAAAAGAAAAAAATTGATCTAT<br>CGATTTCAATTCAATTCAATCTAGTTCATTAATCCATTTGCTAGTCTTGCTCTTAGAT<br>CCTTCCTCAATATCTTCCCTGATGGAGCTTTAGGAATAGAGTCAGTGAAGAACACTT<br>TGTTGATTCTCTTATAAAACACAACCTGTTTTGACACGAATTGCTTGATTTCATCTTC<br>GGATATATTTGAATCTTTCGATCTCACCACAAACGCAACAGGAACCTCACCAGCAT<br>CTTCTTCCTTCATGGCGACGACAGCAACATCATTGATTTCTGGATGACCTATGAGG<br>AGAGACTCTAGCTCAGCTGGAGCCACTTGAAATCCTTTGTACTTGATGAGTTCTTTC<br>AATCTATCCACAATGAAAAGCTCGTCGTCATCATCGATAAATCCGACGTCTCCAGT<br>GTGAAGCCAACCATCTTTATCGATCGTCGATGCCGTGGCCAAGGGGTCATTGAGAT<br>AGCCTTTCATGATTTGGTTGCCACGGATGCATATTTCGCCGGGTTTGTTCCTAGGC<br>AAAGAATCTCCTGTGTCTGGATCAAGTATCTTCATCTCGGCGTTCCTCACCACCGT<br>ACCACATGCTCCTGACTTCACTGGAAACGGCTCTTTAGCAAACCCTAACGACATTG<br>CTAGCACCGGACCTGCTTCTGTCATCCCATAGCCCTGACCAAGCTTGGCGTTAGG<br>AAACTTAGCACTAATAGCATCTTCAAGCTCCTTACCAAGAGGAGCTGCTCCAGACT<br>TAACCATCCTAACCGAGCTCAGATCATACTTCTCCGTCTCCGGCGACTTCGCGATA<br>GCTAAAACGATCGGTGGCACGACCATAGCCACCGTGACTTTACACCTTTGTATCTG<br>CTCTAACAAGAGAGTGATTTCGAACTTAGGCATTATCAAGATCGTGGCACCAACTC<br>TGAGACTACAGAGCATGATGGAGTTGAGAGCGTATATATGGAACATAGGCAAGACA<br>CAGAGGATCACGTCGTCTCTGTTGAAGTAAAGATTCGGATTCTCGCCGTCGACTTG<br>CTGCGCCACGCTCGTGACTAGACCTTTGTGTGTTAGCATCACTCCTTTGGGGAGAC<br>CCGTCGTGCCGGATGAGAAAGGAAGCGCCACGACGTCTTCTGGCGAAATCTTCTC<br>CGGTATTGAGTCCACTCGTGGTTCTTCGGACTGAGTTAACTCGGAGAAACGGAGG<br>CAGTTTTCGGGGATGGCGTCGGAGTCGGTGGTGACGATCAAAACGCCGTCGTTTT<br>GGAGGTTCTTGATTTTATCGACGTAACGGGATTGAGTGACGATGAGTTTCGCCGCG<br>GAGGCTTTGGCTTGTTTAGAAATCTCCGCCGGAGTGAAGAACGGGTTCGCGGAGG<br>TGGTGATTGCGCCGATGAAGGAGGCGGCAAGGAAAGTGAGGACTACTTCAGGAGA<br>GTTCGGGAGGAGGATCATTACAACGTCGTGTTGCTTCACGCCGAGGTTATGAAGA<br>CCGGCGGCGAGTTTCCGAGATGTTACGTGGACATCGGCGTAGGTGTATACTTCGC<br>CGGTGGGACCGTTGATCAAGCATGGCTTAGCGGCGAACTCTGAGATATTTTCGAA<br>GATGTAGTCGTGGAGTGGGAGGTGGTTAGGGATGTATATATCAGGCAATCTCGAT<br>CGGAAAATGACGTCATTACTACACTGTTTCTGATCATTCTGATCATTGACTATCACA<br>TCTTGTGTCGTCATTGTTTTATATTTGTTGTAAAAAGTAGATAATTACTTCCTTGATG<br>ATCTGTAAAAAAGAGAAAAAGAAAGCATCTAAGAACTTGAAAAACTACGAATTAGAA<br>AAGACCAAATATGTATTTCTTGCATTGACCAATTTATGCAAGTTTATATATATGTAAA<br>TGTAAGTTTCACGAGGTTCTACTAAACTAAACCACCCCCTTGGTTAGAAGAAAAGA<br>GTGTGTGAGAACAGGCTGTTGTTGTCACACGATTCGGACAATTCTGTTTGAAAGAG<br>AGAGAGTAACAGTACGATCGAACGAACTTTGCTCTGGAGATCACAGTGGGCATCAT<br>AGCATGTGGTACTAAACCCTTTCCCGCCATTCCAGAACCTTCGATTGCTTGTTACAA<br>AACCTGTGAGCCGTCGCTAGGACCTTGTTGTGTGACGAAATTGGAAGCTGCAATCA<br>ATAGGAAGACAGGAAGTCGAGCGTGTCTGGGTTTTTTCAGTTTTGTTCTTTTTGCAA<br>ACAAATCACGAGCGACGGTAATTTCTTTCTCGATAAGAGGCCACGTGCTTTATGAG<br>GGTAACATCAATTCAAGAAGGAGGGAAACACTTCCTTTTTCTGGCCCTGATAATAGT<br>ATGAGGGTGAAGCCAAAATAAAGGATTCGCGCCCAAATCGGCATCTTTAAATGCAG<br>GTATGCGATAGTTCCTCACTCTTTCCTTACTCACGAGTAATTCTTGCAAATGCCTAT<br>TATGCAGATGTTATAATATCTGTGCGTAGTTTATCATTATCAATACTGCCATTTCAAA<br>GAATACGTAAATAATTAATAGTAGTGATTTTCCTAACTTTATTTAGTCAAAAAATTAG<br>CCTTTTAATTCTGCTGTAACCCGTACATGCCCAAAATAGGGGGCGGGTTACACAGA<br>ATATATAACATCGTAGGTGTCTGGGTGAACAGTTTATTCCTGGCATCCACTAAATAT<br>AATGGAGCCCGCTTTTTAAGCTGGCATCCAGAAAAAAAAAGAATCCCAGCACCAAA<br>ATATTGTTTTCTTCACCAACCATCAGTTCATAGGTCCATTCTCTTAGCGCAACTACA<br>GAGAACAGGGGCACAAACAGGCAAAAAACGGGCACAACCTCAATGGAGTGATGCA<br>ACCTGCCTGGAGTAAATGATGACACAAGGCAATTGACCCACGCATGTATCTATCTC<br>ATTTTCTTACACCTTCTATTACCTTCTGCTCTCTCTGATTTGGAAAAAGCTGAAAAAA<br>AAGGTTGAAACCAGTTCCCTGAAATTATTCCCCTACTTGACTAATAAGTATATAAAG<br>ACGGTAGGTATTGATTGTAATTCTGTAAATCTATTTCTTAAACTTCTTAAATTCTACTT<br>TTATAGTTAGTCTTTTTTTTAGTTTTAAAACACCAAGAACTTAGTTTCGAATAAACAC<br>ACATAAACAAACAAAATGGTTACTGTTGAAGAAGTTAGAAAAGCTCAAAGGGCAGA<br>AGGTCCAGCCACAGTGATGGCTATTGGAACCGCAGTTCCTCCAAATTGTGTAGATC

	AGGCCACTTATCCTGACTACTACTTTAGAATAACAAACTCTGAGCATAAGGCTGAAT<br>TGAAAGAAAAGTTCCAAAGGATGTGCGACAAATCACAGATCAAGAAAAGATACATG<br>TACCTTAATGAGGAAGTCCTAAAGGAAAACCCAAATATGTGTGCATACATGGCCCC<br>TTCCCTTGACGCTAGACAAGATATTGTGGTTGTAGAGGTCCCAAAATTGGGCAAGG<br>AAGCAGCTGTTAAAGCCATAAAGGAATGGGGTCAACCTAAGAGCAAAATCACCCAC<br>CTTGTGTTTTGCACTACAAGCGGAGTTGACATGCCAGGCGCAGATTATCAGCTAAC<br>CAAACTTTTGGGTTTAAGGCCTTCTGTAAAAAGATTGATGATGTACCAACAAGGTTG<br>TTTCGCTGGAGGCACTGTCTTAAGACTAGCCAAGGATCTTGCAGAGAACAACAAAG<br>GTGCTAGGGTGTTGGTTGTATGCTCAGAAATTACAGCCGTCACCTTTAGAGGACCA<br>ACTGACACTCACTTAGATTCCCTAGTTGGTCAGGCATTGTTTGGCGACGGTGCTGC<br>CGCAATAATCATTGGAAGTGATCCTATTCCAGAGGTGGAAAAGCCTCTTTTTGAACT<br>TGTTAGCGCTGCCCAAACTATATTGCCAGATTCTGAGGGTGCAATCGACGGCCACT<br>TAAGGGAAGTAGGTCTAACCTTCCATCTTTTGAAAGATGTCCCTGGTTTAATTTCAA<br>AGAACGTGGAAAAATCCCTAACAGAGGCTTTTAAACCATTGGGTATAAGTGACTGG<br>AATAGCTTATTCTGGATCGCTCACCCAGGCGGCCCTGCCATACTTGACCAGGTTGA<br>AGCAAAATTGAGCTTAAAGCCAGAAAAACTAAGAGCTACTAGACATGTATTGTCAGA<br>GTATGGTAACATGTCCAGTGCCTGTGTCCTTTTCATTTTGGATGAAATGAGGAGAAA<br>AAGCAAGGAGGACGGCCTAAAAACCACAGGTGAGGGAATCGAATGGGGTGTTCTA<br>TTCGGCTTTGGTCCAGGCCTTACTGTGGAGACAGTTGTACTTCATTCAGTCGCAAT<br>TAATTAAGTGAATTTACTTTAAATCTTGCATTTAAATAAATTTTCTTTTTATAGCTTTAT<br>GACTTAGTTTCAATTTATATACTATTTTAATGACATTTTCGATTCATTGATTGAAAGCT<br>TTGTGTTTTTTCTTGATGCGCTATTGCATTGTTCTTGTCTTTTTCGCCACATGTAATA<br>TCTGTAGTAGATACCTGATACATTGTGGATGCTGAGTGAAATTTTAGTTAATAATGG<br>AGGCGCTCTTAATAATTTTGGGGATATTGGCTTTTTTTTTTAAAGTTTACAAATGAAT<br>TTTTTCCGCCAGGATAACGATTCTGAAGTTACTCTTAGCGTTCCTATCGGTACAGCC<br>ATCAAATCATGCCTATAAATCATGCCTATATTTGCGTGCAGTCAGTATCATCTACAT<br>GAAAAAAACTCCCGCAATTTCTTATAGAATACGTTGAAAATTAAATGTACGCGCCAA<br>GATAAGATAACATATATCTAGATGCAGTAATATACACAGATTCCTATTGATCCGTAC<br>TAATATTGTTAGATGAGCTGCCGAAATTGTTCGCATTGCCATCCTTCAGGAAGAATC<br>CTAATTGTATGCCATTAATGAAAAGTGTGAAAATTAACAAAGAATCCAGAGTTGTTA<br>TATAGACTCTGTGAGACTCGCTTTGTACCGATATTGGGCTCCTCAAGGTATTATGAA<br>AATAGTCCACTGTGCAGCCCATGGTGATCAGTGGCAATGCAAAATTAAGAACGTTG<br>TAAAAGTATCCAATATGAAACTTGCTAGGCGCAAAAACAGTCTCTGTGATAAACGAT<br>ATCAGTGACGGTATTCCATATAACAATGTCGAGCATACAATAACTATCAGTACCGCT<br>TCGTGGTCGTGGAACTTGCATAGCAATGCGATAGCAATGGCACCTGTGGCAATTGA<br>GAGAAGAGCACTTCCGAACTTGATCCCCAAAAAACCAGCCTTATTTCCGCTCCTAA<br>CAGAATTATGGCACATAAGAGAGTATGATTCCTTTCTTAGTATATATATACTGCTCAA<br>GGGCAAATGCAATTACTTTGGAAATGCTTTCTTGTTCTGATACGTAAGAAGACAAGT<br>AATCTCTGGACTAAAAGTGGAGCCGGAATAGAGACTCTCTTAGTATCAGTATCCAC<br>CTCTTCAAAGGATAATGCGTTCTTTGCCAATAGTCACATATTGTGGGTATGTGCGTT<br>TTGCAAAGGCAGCAATATATTGTTTACTTATTTCCCAAAAAGGAAAAATCTTTAAACC<br>ATCCTAGAACTTCAATTCACCACGTTCAGGGTAATATATTTTAACCGCCGACGTTCT<br>CGCCATAACTTTTAAACAGTGATAACTAGTATTGAAACTTAGAAGAGAAAAGGGTAA<br>AGTTAATGCTTAATCTTGTCTTGGCTTAAAAAGTAATATGTACTTATTTACGTCTTCA<br>CAAAATCTAGCAGAAAAACGGTAGTATTTATGTATATTCAAAAAAAAATCATTATCCT<br>CATCAAGATTGCTTTATTTAGCAGATTGGAATAGGTGCACCATTCCACTCTTTCAAG<br>CAATCCATAAGTGGATCTATCAACTTTCCCTCGCACATAGCTGTGAATACCTTGTCA<br>AATTCTTCACCTGGGCTAACGACTTTTTCACCAGTTAGTAATTTGGTTCCCAACTCT<br>TCTCTAACGAATCTGTACAAAGGGTACGACCTACACTCTTTGATTCTATTTGGTATA<br>GGGGCAGTACCATTTCCGTATGCGGCTCTAGCAGCTTCGACTTCCTTTGGTAAAAC<br>TGCCTTCAGTTCTTCTTCAAAGGCACCTATCTTTTGGAATATTGAAGTAACGGCATT<br>TTTCTCAGTTTCACCATTGGATAAAGCGTGATCTACAATAACTTGTCTCAATCTCTG<br>CATCAATGGATAAGTAGCGCTACATGGATCGTCAACGTAAGTAAATACTTGTTCTCT<br>ATCTACAACTTTTAATAAATCTTTTTCACAGAATCTTGATGGGTGCAATTCACCATTG<br>ATACCTGTAGTTAGAACCTTTTTTGCAACCTGTGATACGGTATTTTTCACTGTCTGT<br>CTCAAATTCTCTTCCAAGTGTCTCAAATCTACGGCCTGGCATATACCCACTAAAAAT<br>GTTGTGGACATTAATTTAAGGATATCAACGGCCTCGCTTGTTTTTCTTGATGAAATC<br>AGGCCCAAAGAATTAACATCCTGATTGTGTTGTTCGGCTGATTGTACATGAGAGGT<br>TACTGGGTTGGCTAGATATTGCAGCTCTGAACAATAGCTTGCCATTGCTATCTCAG<br>CACCTTTGAAACCATAATCAAGACTAGGGTTAGAAGATGCGGTCAGATTCGAAGGC<br>AAACCGTTATTGTAGAAGTCATTGACCAATTCAGAAAATTGGGCAAACATTAATTTG

	CCAATTGCGGCTATGGCAAGCCTGGTATTATCCATACTGACTCCTATGGGTGTACC<br>CTGGAAATTGCCTCCATGTATTGCCTTATTCCTCGACACATCAATAAGTGGATTATC<br>GTTAACAGAGTTGATCTCTCTTTCTATAGACTTTGTAGCTTGTCTAATTACTTCAATT<br>TGAGGGCCAAGCCATTGTGGGGATGTCCTTAAAGCATATCTATCTTGTTTGGGTTT<br>TTGCAAAGGGTCCATTTCATGAACCTTCTGGGCTAACTTCATGTAGCTAGAGCCGT<br>CCAAAATGTGCTCCATGATAGCTGCTGCTTCAATTTGTCCTGGGTGATGTTTTAACC<br>TGTGGGTCAAGTGATCAGTAAACTCAGGTTTTCCACTCATGACTTCGGCAAAAATT<br>GCGGACAAAACTTCGGCCAAAACTGCTTGTACGTTAGCTTCAAACAACACCATGGA<br>TGCCATACCGCTGCCGACAGCGGTGCCATTCACCAGGGCTAAACCTTCCTTGGGT<br>TGCAAATCAAAGAAACCAGTTGAAATACCAGCTTTCTCAAATGCTTCCTTAGCGGTT<br>AAGGATTCTCCGTCTGGACCAGTGGCCTTTGAATTAGGTCTTCCCGTTAATAAGCC<br>TGCGATATATGAAAGGGGAACCAAATCACCGCTGGCAGTTATTGTTCCTCTTAAGG<br>GCAACGAAGGAGAAATGTTGTGGTTCAATAGTGAAGTGATGGCCTCAAGAATTTCA<br>AACCTTATTCCAGAGTAACCTTGCAACAAAGTGTTCACCCTAACAAGCATAGCAGCT<br>CTTGTTGCCGATTGGGGTAATGTATGGCAAGTTTCCTTTGTATTACCGAAAATACCG<br>GCGTTAAGGAATCTGATCAGTTCTGTTTGCAAAGCAGTGCCATTTTTAGTTCTTCTA<br>TGAGAGGTAGCACCAAAGCCTGTGGTAACGCCATAGGAATCTGTGCCCTTGTTCAT<br>ACTTTCCATGACCCAATCTGATGAAGCCTTAACTCCGGCTCTACTTGTTTCTGCAAG<br>TTCTACCTTCACTGAACCGCCAACGGTCGAAATAGCAGCTACCTGTCCTATCGTCA<br>ATGTCTCGCCGCCTAGATTTACGACTGGTCTTCTGTATTCCTCAACCATCTTCTTAA<br>CTTCATCCAGATGGCTACCTTTCATCTGGTCAGCTGCCAGACCCCAATTCAAAGGA<br>TCTGCAAGAGTTTTTGTCGTTACGGCCACCTTGGTCTTTTCACCACCACCGCATAG<br>CATTGCTTCAATTTGGTCCATTTTGTTTTGTGTGTAAATTTAGTGAAGTACTGTTTTT<br>TGTGTGTGTTGGTGAAATATCAAACCAAGTTCTTGATGAATTTCTTATTTATGCAAGA<br>GAGAGAATAGAACTGTACTACAAATCTCATTGTGTGAAAATATATTGTCTATTTATAT<br>GATTTCGAGACTCCAGTTTTGGTCATTATCACCAAGCTCTTACTGCTACAGAGAATG<br>AACATGCTCCTCCCCCCCTTCTTCAGACTATGTTGTTCTGCACGTGGATACCGTCG<br>CATGCACCTAAGAAGCAGATGGTGGCTTGCCTTACTGTATTGTAAAGATCCAGTCT<br>CCAGATCTGCGACCACTCCGAAGGTTGAAACCCGAGCTTCCTGTTTGCTGTCTCGC<br>GCCTTTTAAAAAAAAAGCGCGATTATGGGCCGCTCGTGACAGTAAAGGAAGCAAGC<br>AGATCGACCCCCTGAAAATGTGGTGTGGTTACTAAGCAGAAGCGTCTTCGTCGCAT<br>ATCCTATTCCTAGCGCAACAAGGCCCCACGGTGTGGTTTCATGTGACGTGGAGTCA<br>TGTAGGCTTGTGGTGCGCACATTTTTACTAATCGAGAGATCTGCGGCCGCATGGAG<br>AAAAAGAAAGATCCCCTGATGAATAGTCTCATGAAGTTAGATGTTTTACGAATGAAA<br>AAGAATAATAACCTGATTAGGCACAGGATAGAACAGCTAAATGAGCGGATTTATAG<br>TAAAAGGAATCACATCAGCGAATTGAAAGTAGAAATTGACAATTATAAATGTTATAA<br>GGTGGGTACTGGTACGGACAAATTAAGAGAGCAAGTGGAAATTAGTGATGCAAAAA<br>ATAAGCTGGCACAAGTATCGAAGATTTGTGAATCGGCTAGAGATTACAAGCTCAAT<br>CTACTAAATAATTGGTTTGTGATCCAGAAGCTGCAAGATAATTTCCAAATACCTTTC<br>GCTATAGCTTTTCAGCCACTAATATCTTTGAAAAACTTCCGTATTTTACCTCTTGCTA<br>TAACAAACGATTCCATCAACATCATGTGGAAGTATATTAGCTTCTTCTCAGACATTC<br>TTATGATTAAACTTCCCTACACAAATAAAATCTGTGAACAGGATCCCCGGGTACCGA<br>GCTCGAATTCACTGGCCGTCGTTTTACAACGTCGTGACTGGGAAAACCCTGGCGTT<br>ACCCAACTTAATCGCCTTGCAGCACATCCCCCTTTCGCCAGCTGGCGTAATAGCGA<br>AGAGGCCCGCACCGATCGCCCTTCCCAACAGTTGCGCAGCCTGAATGGCGAATGG<br>CGCCTGATGCGGTATTTTCTCCTTACGCATCTGTGCGGTATTTCACACCGCATATG<br>GTGCACTCTCAGTACAATCTGCTCTGATGCCGCATAGTTAAGCCAGCCCCGACACC<br>CGCCAACACCCGCTGACGCGCCCTGACGGGCTTGTCTGCTCCCGGCATCCGCTTA<br>CAGACAAGCTGTGACCGTCTCCGGGAGCTGCATGTGTCAGAGGTTTTCACCGTCA<br>TCACCGAAACGCGCGAGACGAAAGGGCCTCGTGATACGCCTATTTTTATAGGTTAA<br>TGTCATGATAATAATGGTTTCTTAGACGTCAGGTGGCACTTTTCGGGGAAATGTGC<br>GCGGAACCCCTATTTGTTTATTTTTCTAAATACATTCAAATATGTATCCGCTCATGAG<br>ACAATAACCCTGATAAATGCTTCAATAATATTGAAAAAGGAAGAGTATGAGTATTCA<br>ACATTTCCGTGTCGCCCTTATTCCCTTTTTTGCGGCATTTTGCCTTCCTGTTTTTGCT<br>CACCCAGAAACGCTGGTGAAAGTAAAAGATGCTGAAGATCAGTTGGGTGCACGAG<br>TGGGTTACATCGAACTGGATCTCAACAGCGGTAAGATCCTTGAGAGTTTTCGCCCC<br>GAAGAACGTTTTCCAATGATGAGCACTTTTAAAGTTCTGCTATGTGGCGCGGTATTA<br>TCCCGTATTGACGCCGGGCAAGAGCAACTCGGTCGCCGCATACACTATTCTCAGA<br>ATGACTTGGTTGAGTACTCACCAGTCACAGAAAAGCATCTTACGGATGGCATGACA<br>GTAAGAGAATTATGCAGTGCTGCCATAACCATGAGTGATAACACTGCGGCCAACTT<br>ACTTCTGACAACGATCGGAGGACCGAAGGAGCTAACCGCTTTTTTGCACAACATGG

	GGGATCATGTAACTCGCCTTGATCGTTGGGAACCGGAGCTGAATGAAGCCATACC<br>AAACGACGAGCGTGACACCACGATGCCTGTAGCAATGGCAACAACGTTGCGCAAA<br>CTATTAACTGGCGAACTACTTACTCTAGCTTCCCGGCAACAATTAATAGACTGGATG<br>GAGGCGGATAAAGTTGCAGGACCACTTCTGCGCTCGGCCCTTCCGGCTGGCTGGT<br>TTATTGCTGATAAATCTGGAGCCGGTGAGCGTGGGTCTCGCGGTATCATTGCAGCA<br>CTGGGGCCAGATGGTAAGCCCTCCCGTATCGTAGTTATCTACACGACGGGGAGTC<br>AGGCAACTATGGATGAACGAAATAGACAGATCGCTGAGATAGGTGCCTCACTGATT<br>AAGCATTGGTAACTGTCAGACCAAGTTTACTCATATATACTTTAGATTGATTTAAAAC<br>TTCATTTTTAATTTAAAAGGATCTAGGTGAAGATCCTTTTTGATAATCTCATGACCAA<br>AATCCCTTAACGTGAGTTTTCGTTCCACTGAGCGTCAGACCCCGTAGAAAAGATCA<br>AAGGATCTTCTTGAGATCCTTTTTTTCTGCGCGTAATCTGCTGCTTGCAAACAAAAA<br>AACCACCGCTACCAGCGGTGGTTTGTTTGCCGGATCAAGAGCTACCAACTCTTTTT<br>CCGAAGGTAACTGGCTTCAGCAGAGCGCAGATACCAAATACTGTTCTTCTAGTGTA<br>GCCGTAGTTAGGCCACCACTTCAAGAACTCTGTAGCACCGCCTACATACCTCGCTC<br>TGCTAATCCTGTTACCAGTGGCTGCTGCCAGTGGCGATAAGTCGTGTCTTACCGG<br>GTTGGACTCAAGACGATAGTTACCGGATAAGGCGCAGCGGTCGGGCTGAACGGG<br>GGGTTCGTGCACACAGCCCAGCTTGGAGCGAACGACCTACACCGAACTGAGATAC<br>CTACAGCGTGAGCTATGAGAAAGCGCCACGCTTCCCGAAGGGAGAAAGGCGGAC<br>AGGTATCCGGTAAGCGGCAGGGTCGGAACAGGAGAGCGCACGAGGGAGCTTCCA<br>GGGGGAAACGCCTGGTATCTTTATAGTCCTGTCGGGTTTCGCCACCTCTGACTTGA<br>GCGTCGATTTTTGTGATGCTCGTCAGGGGGGCGGAGCCTATGGAAAAACGCCAGC<br>AACGCGGCCTTTTTACGGTTCCTGGCCTTTTGCTGGCCTTTTGCTCACATGTTCTTT<br>CCTGCGTTATCCCCTGATTCTGTGGATAACCGTATTACCGCCTTTGAGTGAGCTGA<br>TACCGCTCGCCGCAGCCGAACGACCGAGCGCAGCGAGTCAGTGAGCGAGGAAGC<br>GGAAGAGCGCCCAATACGCAAACCGCCTCTCCCCGCGCGTTGGCCGATTCATTAA<br>TGCAGCTGGCACGACAGGTTTCCCGACTGGAAAGCGGGCAGTGAGCGCAACGCA<br>ATTAATGTGAGTTAGCTCACTCATTAGGCACCCCAGGCTTTACACTTTATGCTTCCG<br>GCTCGTATGTTGTGTGGAATTGTGAGCGGATAACAATTTCACACAGGAAACAGCTA<br>TGACCATGATTACGCCAAGCTTGCATGCCTGCAGGTCGACTCTAGAGATCGCTTGC<br>CTGTAACTTACACGCGCCTCGTATCTTTTAATGATGGAATAATTTGGGAATTTACTC<br>TGTGTTTATTTATTTTTATGTTTTGTATTTGGATTTTAGAAAGTAAATAAAGAAGGTAG<br>AAGAGTTACGGAATGAAGAAAAAAAAATAAACAAAGGTTTAAAAAATTTCAACAAAA<br>AGCGTACTTTACATATATATTTATTAGACAAGAAAAGCAGATTAAATAGATATACATT<br>CGATTAACGATAAGTAAAATGTAAAATCACAGGATTTTCGTGTGTGGTCTTCTACAC<br>AGACAAGATGAAACAATTCGGCATTAATACCTGAGAGCAGGAAGAGCAAGATAAAA<br>GGTAGTATTTGTTGGCGATCCCCCTAGAGTCTTTTACATCTTCGGAAAACAAAAACT<br>ATTTTTTCTTTAATTTCTTTTTTTACTTTCTATTTTTAATTTATATATTTATATTAAAAAA<br>TTTAAATTATAATTATTTTTATAGCACGTGATGAAAAGGACCCAGGTGGCACCAACT<br>GTGGGAATACTCAGGTATCGTAAGATGCAAGAGTTCGAATCTCTTAGCAACCATTA<br>TTTTTTTCCTCAACATAACGAGAACACACAGGGGCGCTATCGCACAGAATCAAATTC<br>GATGACTGGAAATTTTTTGTTAATTTCAGAGGTCGCCTGACGCATATACCTTTTTCA<br>ACTGAAAAATTGGGAGAAAAAGGAAAGGTGAGAGCGCCGGAACCGGCTTTTCATA<br>TAGAATAGAGAAGCGTTCATGACTAAATGCTTGCATCACAATACTTGAAGTTGACAA<br>TATTATTTAAGGACCTATTGTTTTTTCCAATAGGTGGTTAGCAATCGTCTTACTTTCT<br>AACTTTTCTTACCTTTTACATTTCAGCAATATATATATATATATATTTCAAGGATATACCA<br>TTCTAATGTCTGCCCCTAAGAAGATCGTCGTTTTGCCAGGTGACCACGTTGGTCAA<br>GAAATCACAGCCGAAGCCATTAAGGTTCTTAAAGCTATTTCTGATGTTCGTTCCAAT<br>GTCAAGTTCGATTTCGAAAATCATTTAATTGGTGGTGCTGCTATCGATGCTATAAGT<br>GTTGCACGTCCACTTTAGCGGCGCTGGAAGCCTCGCAAGAAGGCGCTGATGCCGT<br>TTTGTTAGGTGCTGTGGGTGGTCCTAAATGGGGTACCGGTAGTGTTAGACCTGAAC<br>AAGGTTTACTAAAAATCCGTAAAGAACTTCAATTGTACGCCAACTTAAGACCATGTA<br>ACTTTGCATCCGACTCTCTTTTAGACTTATCTCCAATCAAGCCACAATTTGCTAAAG<br>GTACTGACTTCGTTGTTGTCAGAGAATTAGTGGGAGGTATTTACTTTGGTAAGAGAA<br>AGGAAGACGATGGTGATGGTGTCGCTTGGGATAGTGAACAATACACCGTTCCAGA<br>AGTGCAA

**Table S2. Sequence of primers used in present study.**	
**Primer name**	**Primer sequence (5'- 3')**
MA01	CCAAACCAATGCTAGTAGAGAAGGG

MA02	CTCTCCCTTCTCTACTAGCATTGGT
MA03	CCAAAGAGATAGGTAAATAAACGCG
MA04	CTCTCGCGTTTATTTACCTATCTCT
MA05	CCAAATGGCCCTGATAATAGTATGA
MA06	CTCTTCATACTATTATCAGGGCCAT
MA07	CCAAAGTATCACAACCGACGATCCG
MA08	CTCTCGGATCGTCGGTTGTGATACT
MA09	CCAAAAGTCGAACAAATATTAAACG
MA10	CTCTCGTTTAATATTTGTTCGACTT
MA11	CCAAAATGACCAGTTAGATATCAAG
MA12	CTCTCTTGATATCTAACTGGTCATT
MA13	CCAAATGATTGCACGAACAGAGTGA
MA14	CTCTTCACTCTGTTCGTGCAATCAT
MA15	CCAAATACGAGCCTTGTGAAAAGCA
MA16	CTCTTGCTTTTCACAAGGCTCGTAT
MA17	CCAAAATGGCTTGGGAAGATGTCAG
MA18	CTCTCTGACATCTTCCCAAGCCATT
MA19	CCAAAGCTGTTTACGAAGGTTACGA
MA20	CTCTTCGTAACCTTCGTAAACAGCT
MA21	CCAAAGGATATGACCCAGAGCCGGG
MA22	CTCTCCCGGCTCTGGGTCATATCCT
MA23	CCAAACGTGGCTATACAAATAGGAC
MA24	CTCTGTCCTATTTGTATAGCCACGT
MA25	ATTAACGTTTACAATTTCCTGATGC
MA26	GCATCAGGAAATTGTAAACGTTAATAAAACTGTATTATAAGTAAATGCATG
MA27	CTGCTCTGATGCCGCATAGTTAAGCGATTTATCTTCGTTTCCTG
MA28	TTGCATGCCTGCAGGTCGACTCTAGATGGAACAACGACATCTGTTGGTG
MA29	GCGGCCGCAGATCTCTCGAGGCTAGCCCATGGTAACACCCCTCCGCGCTC
MA30	TTGCATGCCTGCAGGTCGACTCTAGACAACTATGAAAGAAGATCAAGCG
	GCGGCCGCAGATCTCTCGAGGCTAGCCCATGGGGGTGTGTTTATTACATGAAG

MA31	
MA32	TTGCATGCCTGCAGGTCGACTCTAGACACTCACATCTACCAGTG
MA33	GCGGCCGCAGATCTCTCGAGGCTAGCCCATGGGGGTGAAGCCAAAATAAA
MA34	TTGCATGCCTGCAGGTCGACTCTAGAAAAGAGGGTCTGTCCAGC
MA35	GGAGGGTATTCTGGGCCTCCATGTCGGGGTCATGGCGGCTATTT
MA36	TTGCATGCCTGCAGGTCGACTCTAGAGCGCTAATTACATTTGATGAAAG
MA37	CGATACCTGAGTATTCCCACAGTTGTTCCCGTTAAGTTCTTTATTC
MA38	TTGCATGCCTGCAGGTCGACTCTAGACAACGCAAAATAAATAATTAATATGTAAAA TATC
MA39	CTGCTCTGATGCCGCATAGTTAAGCGGCTGAACAAACGTCACAG
MA40	TTGCATGCCTGCAGGTCGACTCTAGAAGGTTGATGCTTCTGGGTTC
MA41	CTCGCTAAAACTGTGCGATGCCTCTAGAAAAACATCACAGCCGAAATG
MA42	TTGCATGCCTGCAGGTCGACTCTAGAAAAGATTAAAAAAAGTCAAAGCTAC
MA43	GGAGGGTATTCTGGGCCTCCATGTCGAGATACACCACAGTCTAC
MA44	CGGAAAAGAGCGCGGAGGGGTGTTACCTAGTACACTCTATATTTTTTATGCCTC G
MA45	CGGCCGCAGATCTCTCGAGGCTAGCCCATGGCGCATAGATCCGTCGAGTTC
MA46	TCTTCTTCATGTAATAAACACACCCCGTATCACGAGGCCCTTTCG
MA47	CGCAGATCTCTCGAGGCTAGCCCATGGCGAGCACAGGGTAATAACTG
MA48	CGAATCCTTTATTTTGGCTTCACCCCGACATGGAGGCCCAGAATACC
MA49	CCGCAGATCTCTCGAGGCTAGCCCATGGCAGTATAGCGACCAGCATTCAC
MA50	GACATGGAGGCCCAGAATAC
MA51	CAGTATAGCGACCAGCATTC
MA52	AACTGTGGGAATACTCAGGTATCG
MA53	CGGCCGCAGATCTCTCGAGGCTAGCCCATGGGGATGCCGGGAGCAGACAAG
MA54	GCTTAACTATGCGGCATC
MA55	GCAGATCTCTCGAGGCTAGCCCATGGATTAACGTTTACAATTTCCTGATG
MA56	TAGAGGCATCGCACAGTTTTAG
MA57	GCATGCCTGATTTACAGTTCTTATTC
MA58	GACATGGAGGCCCAGAATAC
MA59	CAGTATAGCGACCAGCATTCAC
MA60	CCATGGGCTAGCCTCGAGAGATCTGCGGCCGCAATATATGTGCATAGGAGAAGT GTAAAGTTC
MA61	AATTCGAGCTCGGTACCCGGGGATCCCTGACTCTGTGGCGGACC
MA62	CCATGGGCTAGCCTCGAGAGATCTGCGGCCGCTAAACTTCAACACCTTATATCAT AAC
MA63	AATTCGAGCTCGGTACCCGGGGATCCAAAATGTGCCATCACCATAAG
MA64	CCATGGGCTAGCCTCGAGAGATCTGCGGCCGCGAAAAAGGAAGTGTTTCCC
MA65	AATTCGAGCTCGGTACCCGGGGATCCATTAGAAAAGACCAAATATGTATTTC
MA66	TATGTGAATGCTGGTCGCTATACTGCCATGGGCTAGCCTCGAGAGATCTGCGGCC GCAGCGCCCTGGTCAAACTTC
MA67	GAATTCGAGCTCGGTACCCGGGGATCCCATCACAAGCATTCATTTACCAAGAG
MA68	GGCTAGCCTCGAGAGATCTGCGGCCGCATGGAGAAAAAGAAAGATCCC

MA69	AATTCGAGCTCGGTACCCGGGGATCCTGTTCACAGATTTTATTTGTGTAG
MA70	GGCTAGCCTCGAGAGATCTGCGGCCGCTACAGGAAAGCATCGCCC
MA71	AATTCGAGCTCGGTACCCGGGGATCCTGTCCAAGATGGCAAACTTC
MA72	GAATAAGAACTGTAAATCAGGCATGCCCATGGGCTAGCCTCGAGAGATCTGCGGCCGCTTGGTTAAGTGAAGGTGGTAC
MA73	AATTCGAGCTCGGTACCCGGGGATCCCCTGCCATTTTCCGTGAG
MA74	TATGTGAATGCTGGTCGCTATACTGCCATGGGCTAGCCTCGAGAGATCTGCGGCCGCCGGGTGCATGTTGCTGGG
MA75	AATTCGAGCTCGGTACCCGGGGATCCGCTAACACTTACACCAAATTCCAAGTCGTC
MA76	TACCGGGATCCCATAGCTTCAAAATGTTTCTACTC
MA77	TATTAGCGGCCGCCTTCGAGCGTCCCAAAAC
MA78	GTAAACGTTAATCCATGGGCTAGCCGTAGTTTAGTGTTTTTCTTCCA
MA79	ACAAATCGCTCTTAAATATATACC
MA80	AGGTATATATTTAAGAGCGATTTGTCTACACACCGATAACAGGTATTG
MA81	TTAGAATCAAGATACCAGCCTAAAAATGTCTCCACCAGTTTCTG
MA82	TTTTAGGCTGGTATCTTG
MA83	TATTTAGTACTGTCAGTATTGTTATGTAACAAAAATCACGATCTGGGT
MA84	ATAACAATACTGACAGTACTAAATAATTG
MA85	TTTGAATATGTATTACTTGGTTATGG
MA86	CATAACCAAGTAATACATATTCAAAATGGATTTGTTATTGCTGG
MA87	ACTATATCAATTAATTTGAATTAACCACGCGATTTACCATACATC
MA88	GTTAATTCAAATTAATTGATATAGTTTTTTAATG
MA89	CCTGTAGCGGCCGCAGATCTCTCGAGCTATCAAAAACGATAGATC
MA90	AATGCTGGTCGCTATACTGCCATGGGCTAGCCATCCTCTAGAG
MA91	ATTGAATTGAATTGAAATCGATAGATC
MA92	TCTATCGATTTCAATTCAATTCAATCTAGTTCATTAATCCATTTGCTAGTC
MA93	TACTTTTTACAACAAATATAAAACAATGACGACACAAGATGTG
MA94	TGTTTTATATTTGTTGTAAAAAGTAG
MA95	ATGGCAGTATTGATAATGATAAACTACGCACAGATATTATAACATCTGC
MA96	AGTTTATCATTATCAATACTGCCATTTC

MA97	TTTGTTTGTTTATGTGTGTTTATTC
MA98	GAATAAACACACATAAACAAACAAAATGGTTACTGTTGAAGAAG
MA99	AATGCAAGATTTAAAGTAAATTCACTTAATTAATTGCGACTGAATGAAG
MA100	GTGAATTTACTTTAAATCTTGCATTTAAATAAATTTTC
MA101	CACCCGGCGGCCGCAGATCTCTCGAGATAAAGCAATCTTGATGAGGATAATG
MA102	CTCCCGGCATCCCCATGGGCTAGCCTTAGTAAAAATGTGCGCAC
MA103	TTTGTTTTGTGTGTAAATTTAGTG
MA104	TCACTAAATTTACACACAAAACAAAATGGACCAAATTGAAGCAATG
MA105	ATTATCCTCATCAAGATTGCTTTATTTAGCAGATTGGAATAGGTG
MA106	ATAAAGCAATCTTGATGAGGATAATG
MA107	CTCCATGCGGCCGCAGATCTCTCGATATTGATCCGTACTAATATTGTTAGATG
MA108	TGCATGCCTGCAGGTCGACTCTAGAGATCGCTTGCCTGTAACTTACACG
MA109	CTGCTCTGATGCCGCATAGTTAAGCGGTGCCACCTGGGTCCTTTTC
MA110	TCTAGAGTCGACCTGCAG
MA111	CTCCCGGCATCCCCATGGGCTAGCCAGGCATTAAAAGAGGAGC
MA112	CTAACAATATTAGTACGGATCAATAGGAATCTGTGTATATTACTGCATC
MA113	ACGATACCTGAGTATTCCCACAGTTGGTGCCACCTGGGTCCTTTTC
MA114	GCTAGCCCATGGGGATGC
MA115	TATTGATCCGTACTAATATTGTTAGATG

Table S3. List of fragments and respective primers used in construction of plasmids

Plasmid	Fragment	Primer name	Primer sequence (5'- 3')
pHV 001	H1 gRNA	MA01	CCAAACCAATGCTAGTAGAGAAGGG
		MA02	CTCTCCCTTCTCTACTAGCATTGGT
pHV 002	H2 gRNA	MA03	CCAAAGAGATAGGTAAATAAACGCG
		MA04	CTCTCGCGTTTATTTACCTATCTCT
pHV 003	H5 gRNA	MA05	CCAAATGGCCCTGATAATAGTATGA
		MA06	CTCTTCATACTATTATCAGGGCCAT
pHV 004	H7 gRNA	MA07	CCAAAGTATCACAACCGACGATCCG

		MA08	CTCTCGGATCGTCGGTTGTGATACT
pHV 005	YBR1 28C gRNA -1	MA09	CCAAAAGTCGAACAAATATTAAACG
		MA10	CTCTCGTTTAATATTTGTTCGACTT
pHV 006	YBR1 28C gRNA -2	MA11	CCAAAATGACCAGTTAGATATCAAG
		MA12	CTCTCTTGATATCTAACTGGTCATT
pHV 007	YDR4 48W gRNA -1	MA13	CCAAATGATTGCACGAACAGAGTGA
		MA14	CTCTTCACTCTGTTCGTGCAATCAT
pHV 008	YDR4 48W gRNA -2	MA15	CCAAATACGAGCCTTGTGAAAAGCA
		MA16	CTCTTGCTTTTCACAAGGCTCGTAT
pHV 009	YGR2 40C gRNA -1	MA17	CCAAAATGGCTTGGGAAGATGTCAG
		MA18	CTCTCTGACATCTTCCCAAGCCATT
pHV 010	YGR2 40C gRNA -2	MA19	CCAAAGCTGTTTACGAAGGTTACGA
		MA20	CTCTTCGTAACCTTCGTAAACAGCT
pHV 011	YHR1 42W gRNA -1	MA21	CCAAAGGATATGACCCAGAGCCGGG
		MA22	CTCTCCCGGCTCTGGGTCATATCCT
pHV 012	YHR1 42W gRNA -2	MA23	CCAAACGTGGCTATACAAATAGGAC
		MA24	CTCTGTCCTATTTGTATAGCCACGT
pIV0 01	Left homol ogous arm	MA28	TTGCATGCCTGCAGGTCGACTCTAGATGGAACAACGACATCTGT TGGTG
		MA29	GCGGCCGCAGATCTCTCGAGGCTAGCCCATGGTAACACCCCTC CGCGCTC
	HIS3	MA44	CGGAAAAGAGCGCGGAGGGGTGTTACCTAGTACACTCTATATTT TTTTATGCCTCG
		MA45	CGGCCGCAGATCTCTCGAGGCTAGCCCATGGCGCATAGATCCG TCGAGTTC
	Right homol ogous arm	MA60	CCATGGGCTAGCCTCGAGAGATCTGCGGCCGCAATATATGTGC ATAGGAGAAGTGTAAAGTTC
		MA61	AATTCGAGCTCGGTACCCGGGGATCCCTGACTCTGTGGCGGAC C
pIV0 02	Left homol ogous arm	MA30	TTGCATGCCTGCAGGTCGACTCTAGACAACTATGAAAGAAGATC AAGCG
		MA31	GCGGCCGCAGATCTCTCGAGGCTAGCCCATGGGGGTGTGTTTA TTACATGAAG
	URA3	MA46	TCTTCTTCATGTAATAAACACACCCCGTATCACGAGGCCCTTTCG
		MA47	CGCAGATCTCTCGAGGCTAGCCCATGGCGAGCACAGGGTAATA ACTG
	Right homol		CCATGGGCTAGCCTCGAGAGATCTGCGGCCGCTAAACTTCAAC ACCTTATATCATAAC

	ogous arm	MA62	
		MA63	AATTCGAGCTCGGTACCCGGGGATCCAAAATGTGCCATCACCATAAG
pIV003	Left homologous arm	MA32	TTGCATGCCTGCAGGTCGACTCTAGACACTCACATCTACCAGTG
		MA33	GCGGCCGCAGATCTCTCGAGGCTAGCCCATGGGGGTGAAGCCAAAATAAA
	KanMX	MA48	CGAATCCTTTATTTTGGCTTCACCCCGACATGGAGGCCCAGAATACC
		MA49	CCGCAGATCTCTCGAGGCTAGCCCATGGCAGTATAGCGACCAGCATTCAC
	Right homologous arm	MA64	CCATGGGCTAGCCTCGAGAGATCTGCGGCCGCGAAAAAGGAAGTGTTTCCC
		MA65	AATTCGAGCTCGGTACCCGGGGATCCATTAGAAAAGACCAAATATGTATTTC
pIV004	Left homologous arm	MA34	TTGCATGCCTGCAGGTCGACTCTAGAAAAGAGGGTCTGTCCAGC
		MA35	GGAGGGTATTCTGGGCCTCCATGTCGGGGTCATGGCGGCTATTT
	hphMX6	MA50	GACATGGAGGCCCAGAATAC
		MA51	CAGTATAGCGACCAGCATTC
	Right homologous arm	MA66	TATGTGAATGCTGGTCGCTATACTGCCATGGGCTAGCCTCGAGAGATCTGCGGCCGCAGCGCCCTGGTCAAACTTC
		MA67	GAATTCGAGCTCGGTACCCGGGGATCCCATCACAAGCATTCATTTACCAAGAG
pIV005	Left homologous arm	MA36	TTGCATGCCTGCAGGTCGACTCTAGAGCGCTAATTACATTTGATGAAAG
		MA37	CGATACCTGAGTATTCCCACAGTTGTTCCCGTTAAGTTCTTTATTC
	LEU2	MA52	AACTGTGGGAATACTCAGGTATCG
		MA53	CGGCCGCAGATCTCTCGAGGCTAGCCCATGGGGATGCCGGGAGCAGACAAG
	Right homologous arm	MA68	GGCTAGCCTCGAGAGATCTGCGGCCGCATGGAGAAAAAGAAAGATCCC
		MA69	AATTCGAGCTCGGTACCCGGGGATCCTGTTCACAGATTTTATTTGTGTAG
pIV006	Left homologous arm	MA38	TTGCATGCCTGCAGGTCGACTCTAGACAACGCAAAATAAATAATTAATATGTAAAATATC
		MA39	CTGCTCTGATGCCGCATAGTTAAGCGGCTGAACAAACGTCACAG
	TRP1	MA54	GCTTAACTATGCGGCATC
		MA55	GCAGATCTCTCGAGGCTAGCCCATGGATTAACGTTTACAATTTCCTGATG
	Right homologous arm	MA70	GGCTAGCCTCGAGAGATCTGCGGCCGCTACAGGAAAGCATCGCCC
			AATTCGAGCTCGGTACCCGGGGATCCTGTCCAAGATGGCAAACTTC

		MA71	
pIV007	Left homologous arm	MA40	TTGCATGCCTGCAGGTCGACTCTAGAAGGTTGATGCTTCTGGGTTC
		MA41	CTCGCTAAAACTGTGCGATGCCTCTAGAAAAACATCACAGCCGAAATG
	LYS	MA56	TAGAGGCATCGCACAGTTTTAG
		MA57	GCATGCCTGATTTACAGTTCTTATTC
	Right homologous arm	MA72	GAATAAGAACTGTAAATCAGGCATGCCCATGGGCTAGCCTCGAGAGATCTGCGGCCGCTTGGTTAAGTGAAGGTGGTAC
		MA73	AATTCGAGCTCGGTACCCGGGGATCCCCTGCCATTTTCCGTGAG
pIV008	Left homologous arm	MA42	TTGCATGCCTGCAGGTCGACTCTAGAAAAGATTAAAAAAAGTCAAAGCTAC
		MA43	GGAGGGTATTCTGGGCCTCCATGTCGAGATACACCACAGTCTAC
	natMX	MA58	GACATGGAGGCCCAGAATAC
		MA59	CAGTATAGCGACCAGCATTCAC
	Right homologous arm	MA74	TATGTGAATGCTGGTCGCTATACTGCCATGGGCTAGCCTCGAGAGATCTGCGGCCGCCGGGTGCATGTTGCTGGG
		MA75	AATTCGAGCTCGGTACCCGGGGATCCGCTAACACTTACACCAAATTCCAAGTCGTC

Supplementary Table S4. Summary of expression cassettes and corresponding regulatory elements and primers used in this study.

Expression cassette	Promoter	Promoter primers	Gene/ CDS	CDS primers	Terminator	Terminator primers	Plasmid	Integration locus
yEGFP	*TEF1*	*	*yEGFP*	MA76/MA77*	*CYC1*	*	pIV009<br>pIV010<br>pIV011<br>pIV012	*YBR128C*<br>*YDR448W*<br>*YGR240C*<br>*YHR142W*
PhCHI	*PGI1*	MA82/MA83	*PhCHI*	MA80/MA81	*PGI1*	MA78/MA79	pIV013	*YDR448W*
AtC4H:L5:AtATR2	*FBA1*	MA84/MA85	*AtC4H:L5:AtATR2*	MA86/MA87	*FBA1*	MA88/MA89	pIV013	*YDR448W*
At4CL-2	*PGK1*	MA94/MA95	*At4CL-2*	MA92/MA93	*PGK1*	MA90/MA91	pIV014	*YHR142W*
HaCHS	*TDH3*	MA96/MA97	*HaCHS*	MA98/MA99	*TDH3*	MA100/MA101	pIV014	*YHR142W*
AtPAL-2	*TDH1*	MA102/MA103	*AtPAL-2*	MA104/MA105	*TDH1*	MA106/MA107	pIV015	*YBR128C*

* The complete PTEF1-yEGFP-TCYC1 cassette was amplified from an IDT gBlock using primers MA76/MA77; separate promoter- and terminator-specific primer pairs were therefore not used. Plasmid sequences are included in **Supplementary Table 1**. Primer identities and cassette architecture are described, receptively, in **Supplementary Table 2** and Methods).

Supplementary Table 5. Comparison of reported naringenin production in *Saccharomyces cerevisiae* using different pathway, host-engineering, and cultivation strategies.

Study	Pathway genes	Pathway expression strategy	Additional host engineering	Cultivation / substrate	Time	Titer (mg L⁻¹)	Engineering category
Jiang et al., 2005[1]	*RtPAL, At4CL, HaCHS*	Episomal expression	Δ*PAD1*; increased *PAL* expression	Glucose, shake flask	40	~7	Relatively simple pathway / limited host modification
Koopman et al., 2012[2]	*AtPAL1, AtC4H, AtCPR1, At4CL3, AtCHS3, AtCHI1; RcTAL, additional AtCHS3 copies*	Mixed genomic integration and episomal expression	*ARO4*$_{G226S}$; Δ*ARO3*, Δ*ARO10*, Δ*PDC5*, Δ*PDC6* to increase aromatic precursor supply	Glucose;controlled batch bioreactor (pH 5)	38	~109–113	Precursor engineering
Lyu et al., 2017[3]	*FjTAL, At4CL, HaCHS, MsCHI*	Single copy genomic integration	Δ*PHA2*, Δ*ARO10*, Δ*PDC5*; *ARO4*$_{G226S}$	Sucrose/glycerol; shake flask	72	~84–90	Precursor engineering
Rodriguez et al., 2017[4]	*FjTAL, Pc4CL, PhCHS, MsCHI*	Single copy genomic integration	Δ*ARO10*, Δ*PDC5*; *ARO4*$_{K229L}$, *ARO7*$_{G141S}$	Synthetic fed-batch medium; microscale cultivation	72	1.55	Precursor engineering
Mark et al., 2019[5]	*FjTAL, MsCHI, Mt4CL, VvCHS*	Episomal expression	Modified *GAL* regulatory system; *IDH1* silencing; *ACS1* overexpression; *YlACL* expression; *PHA2* downregulation; Δ*PDC5*, Δ*ARO10*	Sucrose/glycerol; shake flask	72	28.68	Enzyme/source + host engineering
Lyu et al., 2019[6]	*FjTAL, At4CL1, HaCHS, PhCHI*	Episomal expression	Aromatic amino-acid-overproducing background; elimination of competing phenylethanol pathway; core flavonoid pathway and PEP/E4P precursor optimization	Glucose; shake flask / Tween 80 supplementation	72	220	Precursor/pathway engineering
Du et al., 2020[7]	*FjTAL, At4CL1, AtCHS, AtCHI*	Single copy genomic integration	*ARO4*$_{K229L}$, *ARO7*$_{G141S}$; Δ*PDC5*, Δ*ARO10*, increased aromatic amino-acid and malonyl-CoA supply	Glucose	72	144.1	Precursor + malonyl-CoA engineering
Gao et al., 2020[8]	*Pc4CL, PhCHS, MsCHI*	Promoter-library pathway optimization	ΔGAL80 chassis; extensive promoter optimization of *Pc4CL*, *PhCHS*, and *MsCHI*	p-Coumaric acid-fed, 5-L bioreactor	120	1,210	Precursor feeding + pathway/process optimization
Li et al., 2021[9]	*FjTAL, Pc4CL, PhCHS, MsCHI*	Multicopy genomic integration / copy-number optimization	*ARO4*$_{K229L}$, *ARO7*$_{G141S}$; Δ*PDC5*, Δ*ARO10*	Glucose	96	149.8	Precursor + copy-number engineering
Zhang et al., 2021[10]	FjTAL, Pc4CL, PhCHS, MsCHI	Multicopy genomic pathway integration + episomal	ΔGAL80; ARO4$_{fbr}$, ARO7$_{fbr;}$ Δ*PDC5*, Δ*ARO10*; *PEX11* overexpression	Fed-batch fermentation	NR	1,129.44	Acetyl-/malonyl-CoA + fed-batch engineering

Study	Pathway genes	Pathway expression strategy	Additional host engineering	Cultivation / substrate	Time	Titer (mg L⁻¹)	Engineering category
		PEX11 overexpression	to enhance peroxisomal fatty-acid β-oxidation and cytosolic acetyl-CoA supply				
FASTOP	*AtPAL-2, AtC4H:L5:AtATR2, At4CL-2, HaCHS, PhCHI*	Single-copy chromosomal integration of each pathway gene/module at YBR128C, YDR448W and YHR142W	No additional precursor or malonyl-CoA host engineering	YPDA; shake flask, 30 °C	16 h	505.7	Pathway integration without additional host metabolic engineering

References

1. Jiang, H.; Wood, K. V.; Morgan, J. A. Metabolic engineering of the phenylpropanoid pathway in *Saccharomyces cerevisiae*. Appl. Environ. Microbiol. 2005, 71 (6), 2962–2969. DOI: 10.1128/AEM.71.6.2962-2969.2005.
2. Koopman, F.; Beekwilder, J.; Crimi, B.; van Houwelingen, A.; Hall, R. D.; Bosch, D.; van Maris, A. J. A.; Pronk, J. T.; Daran, J.-M. De novo production of the flavonoid naringenin in engineered *Saccharomyces cerevisiae*. Microb. Cell Fact. 2012, 11, 155. DOI: 10.1186/1475-2859-11-155.
3. Lyu, X.; Ng, K. R.; Lee, J. L.; Mark, R.; Chen, W. N. Enhancement of naringenin biosynthesis from tyrosine by metabolic engineering of *Saccharomyces cerevisiae*. J. Agric. Food Chem. 2017, 65 (31), 6638–6646. DOI: 10.1021/acs.jafc.7b02507.
4. Rodriguez, A.; Strucko, T.; Stahlhut, S. G.; Kristensen, M.; Svenssen, D. K.; Forster, J.; Nielsen, J.; Borodina, I. Metabolic engineering of yeast for fermentative production of flavonoids. Bioresour. Technol. 2017, 245, 1645–1654. DOI: 10.1016/j.biortech.2017.06.043.
5. Lyu, X.; Zhao, G.; Ng, K. R.; Mark, R.; Chen, W. N. Metabolic engineering of *Saccharomyces cerevisiae* for de novo production of kaempferol. J. Agric. Food Chem. 2019, 67 (19), 5596–5606. DOI: 10.1021/acs.jafc.9b01329.
6. Mark, R.; Lyu, X.; Ng, K. R.; Chen, W. N. Gene source screening as a tool for naringenin production in engineered *Saccharomyces cerevisiae*. ACS Omega 2019, 4 (7), 12872–12879. DOI: 10.1021/acsomega.9b00364.
7. Du, Y.; Yang, B.; Yi, Z.; Hu, L.; Li, M. Engineering *Saccharomyces cerevisiae* coculture platform for the production of flavonoids. J. Agric. Food Chem. 2020, 68 (7), 2146–2154. DOI: 10.1021/acs.jafc.9b07916.
8. Gao, S.; Zhou, H.; Zhou, J.; Chen, J. Promoter-library-based pathway optimization for efficient (2s)-naringenin production from p-coumaric acid in *Saccharomyces cerevisiae*. J. Agric. Food Chem. 2020, 68 (25), 6884–6891. DOI: 10.1021/acs.jafc.0c01130.
9. Li, H.; Gao, S.; Zhang, S.; Zeng, W.; Zhou, J. Effects of metabolic pathway gene copy numbers on the biosynthesis of (2s)-naringenin in *Saccharomyces cerevisiae*. J. Biotechnol. 2021, 325, 119–127. DOI: 10.1016/j.jbiotec.2020.11.009.
10. Zhang, Q.; Yu, S.; Lyu, Y.; Zeng, W.; Zhou, J. Systematically engineered fatty acid catabolite pathway for the production of (2s)-naringenin in *Saccharomyces cerevisiae*. ACS Synth. Biol. 2021, 10 (5), 1166–1175. DOI: 10.1021/acssynbio.1c00002.
